# Reshuffle Bonds by Ball Milling: A Mechanochemical Protocol for Charge-Accelerated Aza-Claisen Rearrangements

**DOI:** 10.3390/molecules28020807

**Published:** 2023-01-13

**Authors:** Christian Schumacher, Lieselotte Fritz, Lena M. Hanek, Vitali Sidorin, Daniel Brüx, Carsten Bolm

**Affiliations:** Institute of Organic Chemistry, RWTH Aachen University, Landoltweg 1, D-52074 Aachen, Germany

**Keywords:** Claisen rearrangement, ball milling, mechanochemistry, amide, lactam, ring expansion

## Abstract

This study presents the development of a mechanochemical protocol for a charge-accelerated aza-Claisen rearrangement. The protocol waives the use of commonly applied transition metals, ligands, or pyrophoric Lewis acids, e.g., AlMe_3_. Based on (heterocyclic) tertiary allylamines and acyl chlorides, the desired tertiary amides were prepared in yields ranging from 17% to 84%. Moreover, the same protocol was applied for a Belluš–Claisen-type rearrangement resulting in the synthesis of a 9-membered lactam without further optimization.

## 1. Introduction

Our modern society is still built on a fade-away foundation—the usage of fossil resources. These hydrocarbon deposits, especially petroleum, are a treasured feedstock since they are used to manufacture a plethora of products placing the chemical industry in competition with the energy sector [1,2]. As a result, an unambiguous goal of modern chemistry is the design of efficient chemical transformations with high atom economies that take account of the valuable raw material base. Thus, reactions increasing the chemical space and product complexity while offering high atom economies are of particular interest [3,4,5]. These requirements are matched by sigmatropic rearrangement reactions [6,7,8,9,10,11,12,13,14], that are represented by famous transformations, such as the Fischer indole syntheses [15,16,17,18,19,20], Cope [21,22,23,24,25,26,27], or Claisen rearrangements [28,29,30,31,32,33,34,35,36], that are all valuable C–C and C–X bond formation reactions. Of note is the synthetic value of the Claisen rearrangement, which is demonstrated by the development of numerous descendants such as Belluš– [37,38,39,40], Eschenmoser– [41,42,43,44], Ireland– [45,46,47,48,49,50], or Johnson–Claisen variants [51,52,53,54]. Despite their synthetic relevance as bond forming reactions, all of these original protocols have one disadvantage in common: a high energy demand [21,30,31]. Consequently, catalytic protocols that make use of transition metal complexes [55,56,57,58,59,60,61], Lewis or Brønsted acids [62,63,64,65,66,67,68,69,70,71,72,73,74], or organocatalysts [75,76,77,78,79,80], were developed to shape the (energy) efficiency of the Claisen rearrangement. Along these lines, the ‘on-water’-effect was explored to reduce the reaction temperatures, too [81,82,83,84,85,86,87,88,89,90,91,92,93,94]. In addition, the use of alternative energy inputs, such as photochemistry [95,96,97,98], or microwave irradiation [99,100,101,102,103,104], that do not solely rely on a thermal activation led to further improvements.

However, mechanochemistry [105], which is the induction of chemical transformations by mechanical forces such as grinding, milling, pulling, shearing, or cavitation [106], has for long not been considered as alternative activation mode for rearrangement reactions. This idle potential is surprising, since mechanochemical reactions are known to offer unique advantages such as an altering product selectivity [107], the use of insoluble compounds [108,109,110], or fast and energy efficient reactions due to the absence of solvent [111,112,113]. Recently, the situation changed, as mechanochemical protocols started to enhance the toolbox for rearrangement reactions. For instance, the first protocols for main group, Lossen, or Beckmann rearrangements were reported in the last ten years [114,115,116,117,118]. However, the extension to sigmatropic rearrangement reaction was still missing until Yan and coworkers recently reported about a mechanochemical diaza-Cope rearrangement that significantly outstrips the reaction rate of other protocols that are conducted under solution, neat or sonication conditions [119,120].

Motivated by this result and the absence of a mechanochemical Claisen-type rearrangement, we started to explore this classic reaction under ball milling conditions. The first idea was to use aromatic allyl ethers to perform the original aromatic Claisen rearrangement [28]. However, Lamaty, Métro, and coworkers demonstrated in a control experiment that the reaction does not take place in a ball mill [121]. Therefore, our next idea was the investigation of a charge-accelerated amide enolate aza-Claisen rearrangement under ball milling conditions. Such a transformation is presented by the reaction between acyl chlorides and tertiary allylamines in the presence of a base which results in the formation of γ,δ-unsaturated amides [122]. In the literature, two mechanistic proposals are provided for these kinds of reactions. The first one describes the acylation of the tertiary amine followed by a deprotonation of the formed acylammonium salt to yield the required [3,3]-sigmatropic rearrangement framework as a zwitterion [123]. The second possibility is an in situ formation of a ketene by a base-mediated dehydrohalogation of the corresponding acyl chloride and the subsequent nucleophilic attack on the carbonyl carbon by the tertiary amine resulting in the same zwitterionic intermediate [62]. The described zwitterionic reactions are driven by charge neutralization that results in a serious cut of the reaction temperature from 200–350 °C to 80–140 °C [122]. Furthermore, a temperature reduction was demonstrated for solution protocols but they required additional adjustments such as high catalyst loadings [63], the use of pyrophoric AlMe_3_ [122,123], a consequently increased effort in the reaction setup, or extended reaction times [124].

In this context, we describe the proof-of-concept design of a mechanochemical charge-accelerated amide enolate aza-Claisen rearrangement protocol that grants access to a range of γ,δ-unsaturated amides within 30 min, while not relying on additional additives and keeping a plain synthetical procedure. After an optimization, including reaction time, stochiometric amounts, and the reaction setup, we were able to synthesize nine γ,δ-unsaturated amides of diverse substitution patterns in low to good yields. The protocol was extended to a 1 mmol scale resulting in a slightly increased yield. Moreover, it was transferred to a Belluš–Claisen-type rearrangement that gave access to an azonine derivative by ring enlargement. Thus, a potentially universal use in similar Claisen-type reactions under ball milling conditions is demonstrated.

## 2. Results and Discussion

### 2.1. Optimization 

For our investigation, we focused on the synthesis of γ,δ-unsaturated amide **3aa** accessible by a [3,3]-sigmatropic bond reorganization event between propionyl chloride (**1a**) and *N*-allylmorpholine (**2a**). As described before [62,122,123], a base is needed to form the required [3,3]-sigmatropic core system. Therefore, we started the optimization by a base screening [125]. Using stainless steel as milling material and one ball (10 mm in Ø), amine **2a** and 1.2 equiv. of propionyl chloride (**1a**) were milled for 1 h at 25 Hz in the presence of 1 equivalent of the chosen base. First, the reaction was performed in the absent of base, which resulted in only 3% of amide **3aa** (Table 1, entry 1). Next, potassium and cesium carbonate were tested as frequently used bases in organic transformations; however, no product formation was observed (Table 1, entry 2 and 3). A first improvement was achieved when LiOH or NEt_3_ were applied. Their use resulted in the formation of **3aa** in yields of 9% and 11%, respectively (Table 1, entry 4 and 5). As the yield was still low, the strong, non-nucleophilic base DBU was used, expecting to either ease the formation of the amide enolate or favor the dehydrohalogenation [62,122,123]. However, with DBU, no product was formed (Table 1, entry 6). 

Then, a breakthrough was achieved when diisopropylethylamine (DIPEA, Hünig’s base) was used as the mediator. Its usage resulted in an increased yield of 35% of amide **3aa** (Table 1, entry 7). A similar yield of 40% was obtained when the reaction was repeated in a ZrO_2_-Y milling container (Table 1, entry 7). As this showed only a small effect on the reaction, we out ruled a crucial effect of the used stainless steel as milling material or a potential ‘mechanocatalysis’ [126]. Of the plethora of tested bases (see Supplementary Material, Table S1), no further improvement was made. Hence, a potential influence of the used amount of DIPEA was investigated. Therefore, 0.5, 1.0, and 1.5 equivalents of DIPEA were tested and yields of 34%, 33%, and 18%, respectively (Table 1, entry 8–10; see also Supplementary Material, Table S2), were obtained for amide **3aa**. However, repeating the reactions resulted in surprisingly low yields of 1%, 6%, and 4% of **3aa**. This observation was a first indication that the reaction might proceed by an in situ formation of a volatile ketene species and would be crucial for the reaction setup (vide infra). The collected data so far revealed a diminished yield when an excess of base is used. As a result, we decided to use a stochiometric amount of DIPEA (Table 1, entry 9 vs. 10). Next, we concentrated on the milling time (see Supplementary Material, Table S3). A relatively short milling period of 15 min resulted only in 27% of **3aa** (Table 1, entry 11). A significant increase in the formation of amide **3aa** was achieved, when the substrates were subjected to a milling time of 30 min which resulted in a yield of 58% (Table 1, entry 12). A further extension to 120 min of milling decreased the yield of **3aa** to 42% (Table 1, entry 13). A yield reduction upon an increased milling time (Table 1, entry 12 vs. 9 and 13) might be the result of competing reactions such as a von Braun degradation or a fragmentation/*N*-dealkylation reaction that are described by Nubbemeyer and Vedejs [123,127]. However, we were not able to isolate any of the expected side products after column chromatography. We continued our study by varying the number of milling balls. Using three smaller balls (7 mm in Ø) instead of one ball (10 mm in Ø) we obtained **3aa** in a yield of only 24% (Table 1, entry 14). Thus, we decided to continue the optimization by using a milling time of 30 min and one ball (10 mm in Ø). As we assumed the reaction to proceed by a volatile ketene, we wondered if an increased amount of propionyl chloride (**1a**) would be beneficial for the reaction outcome. When a stochiometric amount of **1a** was used, amide **3aa** was obtained in 20% only (Table 1, entry 15). On the other hand, an excess of **1a** seemed to be beneficial for the reaction and good yields of 69% and 67% were obtained when 1.5 or 2.0 equivalents of propionyl chloride were deployed (Table 1, entry 16 and 17; see also Supplementary Material, Table S4). As these results would be in accordance with our hypotheses of a volatile ketene, we wondered how the reaction could be improved further. As MacMillan and coworkers demonstrated in their asymmetric acyl-Claisen reaction protocol that similar reactions can proceed well in sub-zero temperatures [63], we wondered if cryogenic temperatures during the setup of the reaction could tame the formation of the ketene. Therefore, the milling container was cooled in liquid nitrogen before propionyl chloride (**1a**) was added and then subjected to milling. By this procedure product **3aa** was obtained in a yield of 51% (Table 1, entry 18). As the use of cryogenic temperatures did not show the wanted improvement, we decided to test a final approach. Before, the substances were added to one half of the milling container and then closed. Now, the idea was to add amine **2a** and DIPEA first and attach the other half of the milling container leaving a small gap. Then, using a syringe propionyl chloride was added through the gap and the jar was closed immediately. By this method we were able to prepare **3aa** in a good yield of 77% (Table 1, entry 19). To confirm the reproducibility of the new operation, it was repeated twice. The repetition resulted in similar yields of 76% and 80%, respectively (Table 1, entry 20). In addition, the method allowed us to double the size of the approach, which resulted in an even better yield of 84% (Table 1, entry 20). In parallel, we also investigated the use of several additives, such as Lewis acids or Schreiner’s thiourea catalyst; however, no improvement compared to the developed additive-free protocol was achieved (Supplementary Material, Table S5). 

Having identified suitable conditions for a mechanochemical charge-accelerated aza-Claisen rearrangement, we decided to transfer our protocol to solution for comparison. When the reaction is performed in DCM using a concentration of 0.1 M, no product formation was observed after 30 min and only 6% of product **3aa** were formed after 120 min (Table 1, entry 21). These results clearly indicate a faster reaction under ball-milling conditions. Next, the reaction was performed under neat conditions and amide **3aa** was obtained in a good yield of 82% (Table 1, entry 22). As was recently demonstrated, that a mixing process in a ball mill can facilitate reactions better compared with sole mixing under neat conditions using a magnetic stirring bar [128], and gaseous substrates are no limitation for mechanochemical reactions [129]; thus, we decided to stick to the mechanochemical protocol. In addition, due to the zwitterionic character of the reaction, the mixture started to solidify. Hence, mixing in a ball mill will be more effective than using a stirring bar. 

### 2.2. Synthesis of Additional γ,δ-Unsaturated Amides 3

**Scheme 1:**
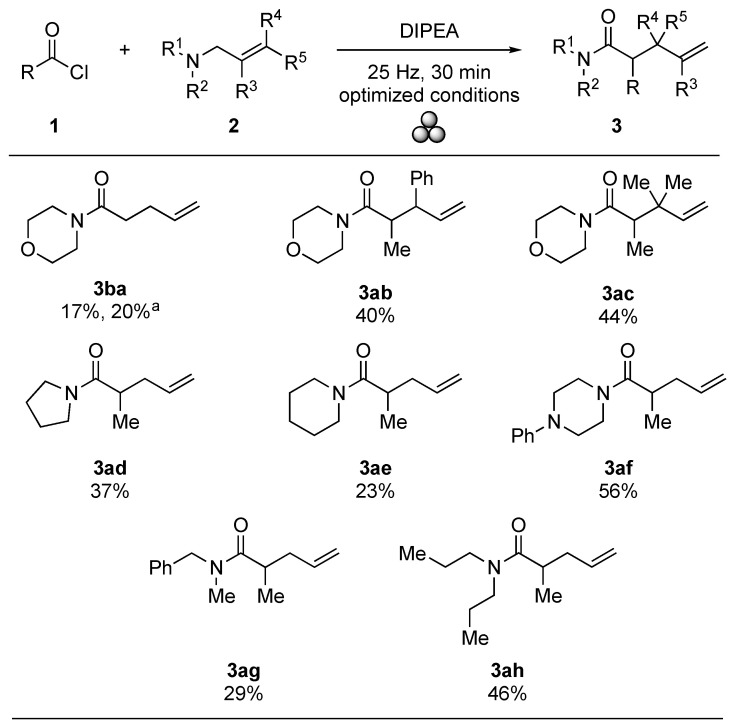
Synthesis of additional γ,δ-unsaturated amides **3** under optimized mechanochemical conditions, see General Procedure 2. ^a^ Acetyl bromide was used instead of acetyl chloride.

With the optimized conditions in hand, we investigated the limitations of the developed mechanochemical charge-accelerated aza-Claisen rearrangement protocol (Scheme 1). First, the acyl chloride **1** was varied. When propionyl chloride was substituted by acetyl chloride the corresponding amide **3ba** was obtained in a low yield of 17%. The use of acetyl bromide increased the yield of **3ba** slightly to 20%. The reduced yield might be attributed to the even more volatile ethenone, but this result also showed the possible use of acyl bromides as potential ketene precursors. In general, the use of other acyl chlorides proved difficult under the applied conditions (Supplementary Material, Scheme S1). Most likely, the combination of Hünig’s base and acyl halides **1** other than propionyl chloride (**1a**) is not suitable for the desired dehydrohalogenation. 

After these experiments, we varied the tertiary allylamines **2**. First, we investigated different substituents on the double bond while keeping the morpholine core. Using amine **2b** with an installed cinnamyl group the corresponding amide **3ab** was obtained as a mixture of diastereomers in 40% yield. Next, amine **2c** having a prenyl group was tested and a similar yield of 44% was obtained for amide **3ac**. As the substituents were located on the double bond, it was most likely that they hampered the formation of the chair-like transition state that is required for the [3,3]-sigmatropic rearrangement [123]. Then, we kept the allyl group but tested different aliphatic nitrogen containing heterocycles. The combination of pyrrolidine derivative **2d** and **1a** gave access to product **3ad** with a yield of 37%. In a similar way, starting from piperidine derivative **2e** the corresponding amide **3ae** was obtained in a yield of 23%. The use of a more complex phenyl-substituted piperazine derivative **2f** resulted in a yield of 56% of the γ,δ-unsaturated amide **3af**. As the tested amine derivatives **2a–f** were cyclic tertiary amines, we tested more conformationally flexible molecules such as **2g** and **2h**. The unsymmetrically substituted amine **2g** allowed the preparation of amide **3ag** with a yield of 29%. The use of the symmetric dipropyl substrate **2h** resulted in the formation of amide **3ah** in a yield of 46%. All these results show that a range of tertiary allyl amines **2** can be subjected to the developed protocol. However, every change made on acyl chloride **1** or amine **2** influenced the reaction and resulted always in reduced yields compared with the yield of amide **3aa**. In particular, sterically more demanding substrates proved difficult under the tested conditions (Supplementary Material, Scheme S2). 

### 2.3. Extension towards a Mechanochemical Belluš–Claisen-Type Rearrangement

**Scheme 2:**
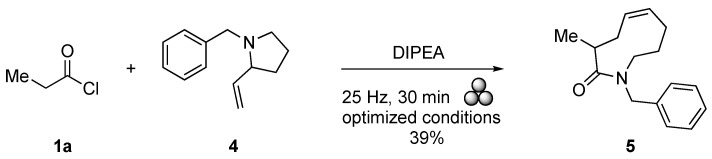
Extension of the optimized protocol to a Belluš–Claisen-type reaction between propionyl chloride (**1a**) and 2-vinylpyrrolidine **4**.

As we found the protocol to be applicable but offering some limitations with respect to the substrate choice, we were curious if an extension towards related aza-Claisen rearrangements without further optimization would be possible. Therefore, a Belluš–Claisen-type rearrangement was investigated. This reaction type is of high synthetic value since it offers the convenience of a ring expansion. Thus, Belluš–Claisen rearrangements are a popular choice for the synthesis of complex lactams as the ring size can be easily varied and the synthesis starts from relatively simple starting materials [130,131]. Motivated by the offered advantages of a transformation, we chose the reaction between propionyl chloride (**1a**) and 2-vinylpyrrolidine **4** as proof-of-concept reaction (Scheme 2). 

In order to gain access to a variety of derivatives of **4**, we evaluated different synthetic routes including the *N*-alkylation and vinylation of pyrrolidine [132,133,134], the reductive amination of allylamine, followed by a *N*-alkylation and cyclization approach (Supplementary Material, Scheme S3). However, at least one step in the tested strategies was difficult or not reproducible. Therefore, we focused on the preparation of starting material **4** by a reported decarbonylative vinylation of proline derivatives for our proof-of-concept reaction [135,136]. Having access to sufficient quantities of 2-vinylpyrrolidine **4**, the starting materials **1a** and **4** were milled under the optimized ball milling conditions using 1.0 equivalent of DIPEA. To our delight, 9-membered lactam **5** could be isolated in a yield of 39% without further optimization. 

This result demonstrates that the developed protocol holds the potential to be used for several aza-Claisen-type rearrangements under mechanochemical conditions. As it offers access to a variety of products and does not rely on (pyrophoric) additives, we hope the presented protocol will stimulate the scientific community to further investigate sigmatropic rearrangement reactions under mechanochemical conditions. 

## 3. Materials and Methods

### 3.1. General Information

#### 3.1.1. Chemicals

Unless otherwise mentioned, all chemicals used were commercially available and used as received. 

#### 3.1.2. Chromatography

Solvents for column chromatography were of technical grade and were distilled prior to use. The stated eluents are always understood as volumetric ratios *v*/*v*. The stationary phase used was always silica gel [Silica 60 M (0.04–0.063 mm), purchased from MACHERY-NAGEL].

Thin layer chromatography (TLC) was performed with silica coated alumina plates [TLC Silica gel 60 F254 from Merck] and the products were visualized using UV-light (*λ* = 254 nm). As many of the substances prepared in this study are UV-inactive, they were visualized either by dipping the TLC plate in an aqueous solution of KMnO_4_ (1.5 g of KMnO_4_, 10 g of K_2_CO_3_, and 1.25 mL of 10% NaOH_(aq.)_ were dissolved in 200 mL of water) and heating of the stained TLC plate with a heat gun until dryness, if necessary, or by putting the TLC plates in an iodine chamber (1 g of I_2_ and 100 g of SiO_2_ were shaken until a homogenous powder was observed). Retention factors (R_f_) are defined as the distance traveled by the compound divided by the distance of the eluent in relation to the baseline. 

#### 3.1.3. Melting Point

Melting points (m.p.) were determined as melting range (range between solidus and liquidus temperature) using a Büchi melting point apparatus M-560, open-end capillaries, a heating rate of 5 °C·min^–1^, and are uncorrected.

#### 3.1.4. Nuclear Magnetic Resonance (NMR) Spectroscopy

NMR measurements were performed either on a Varian VNMRS 600 or Bruker Avance Neo 400 spectrometer. If not stated otherwise, all NMR spectra were recorded at room temperature (25 °C). ^13^C NMR measurements were conducted with proton broad band decoupling indicated as ^13^C{^1^H}. The spectra were processed and analyzed using the program MestReNova [137]. Proton and carbon NMR spectra were referenced to the non-deuterated residual solvent signal (CHCl_3_: ^1^H NMR: *δ* = 7.26 ppm, CDCl_3_: ^13^C{^1^H} NMR: *δ* = 77.16 ppm; DMSO: ^1^H NMR: *δ* = 2.50 ppm, DMSO-d_6_: ^13^C{^1^H} NMR: *δ* = 39.52 ppm; CH_3_CN: *δ* = 1.94 ppm) [138]. ^19^F spectra were referenced using the absolute frequency of the lock signal of the ^2^H resonance signal of the used deuterated solvent. Chemical shifts (*δ*) are reported in ppm (parts per million), and the signals are reported from low to high field. The multiplicity of the peaks is reported as br (broad), s (singlet), d (doublet), t (triplet), q (quartet), p (pentet), m (multiplet) and/or combinations thereof. The spin-spin coupling constants (*J*) are reported in Hz (Hertz). The NMR spectra are depicted in the Supplementary Material, Figures S1–S66.

#### 3.1.5. Infrared (IR) Spectroscopy

IR spectra were recorded neat on a PerkinElmer Spectrum 100 FT-IR spectrometer with an attached UATR device with a KRS-5 crystal. IR bands are reported with their corresponding wavenumber 1/*λ* given in cm^−1^ (in decreasing order) and the relative intensity of transmission (strong (s), medium(m), weak (w)).

#### 3.1.6. Mass Spectrometry (MS)

Mass spectra were recorded on a Finnigan SSQ 7000 mass spectrometer (electron ionization (El), 70 eV; chemical ionization (CI), methane, 100 eV). The signals are given according to their *m*/*z* values and their relative intensity is reported in parenthesis. High resolution mass (HRMS) spectra were recorded as ESI (electrospray ionization, positive mode) spectra on a ThermoFisher Scientific LTQ Orbitrap XL mass spectrometer.

#### 3.1.7. Elemental Analysis (CHN)

CHN analysis was performed either on a Elementar varioEL or Elementar varioEL cube apparatus. The percentage of carbon (C), hydrogen (H), and nitrogen (N) was calculated for a defined compound and compared with the determined amount of the sample.

#### 3.1.8. Mechanochemical Reactions

All mechanochemical reactions were performed using a Retsch mixer mill MM400. The milling containers and balls used were always of the same material. For this purpose, stainless steel, or yttrium-stabilized ZrO_2_ were used. The milling containers used explicitly had a volume of *V* = 10 mL. 

### 3.2. General Procedures

#### 3.2.1. General Procedure 1 (GP1)—Optimization

A milling container of a chosen material equipped with the chosen number of milling balls and chosen diameter was charged with *N*-allylmorpholine (**2a**, 63.6 mg, 0.50 mmol, 1.00 equiv.). Next, the chosen base and chosen additive (10 mol%) were added, if used in the chosen amount. Then, the to be tested amount of propionyl chloride (**1a**) was added volumetrically. The milling container was closed and subjected to milling for a defined time at a chosen frequency. If successful, the product was purified by running a dry-loaded column chromatography.

#### 3.2.2. General Procedure 2 (GP2)—Optimized Conditions

A stainless-steel milling container equipped with one milling ball (10 mm in Ø) was charged with the chosen allylic amine (**2**, 0.50 mmol, 1.00 equiv.). Next, Hünig’s base was added (87 µL, 0.50 mmol, 1.00 equiv.) using an appropriate syringe. Then, the two parts of the container were almost closed leaving a small gap. Using a suitable syringe, the chosen acyl chloride (**1**, 0.75 mmol, 1.50 equiv.) was added through the gap and the jar immediately closed. (Note: This is essential for a successful transformation as most likely a volatile ketene intermediate is formed.) Then, the reaction mixture was placed in the mixer mill and milled for 30 min at a frequency of 25 Hz. After milling, the container was filled with EtOAc, shaken, and the obtained reaction mixture was transferred to a flask. The procedure was repeated (3–5×) to ensure a complete transfer. Finally, product **3** was purified by running a dry-loaded column chromatography. Therefore, a suitable amount of silica gel was added to the flask, and the volatiles were removed under reduced pressure to obtain a free-floating powder, which was placed on top of the column.

#### 3.2.3. General Procedure 3 (GP3)—Solution/Neat Conditions

A 10 mL reaction tube equipped with a magnetic stirring bar was charged with *N*-allylmorpholine (**2a**, 63.6 mg, 0.50 mmol, 1.00 equiv.), which was dissolved in DCM (5 mL), when used. Then, Hünig’s base (87 µL, 0.50 mmol, 1.00 equiv.) and propionyl chloride (**1a**, 66 µL, 0.75 mmol, 1.50 equiv.) were added, the tube closed and stirred at room temperature. After a chosen reaction time, the reaction mixture was transferred to a round bottom flask, and the reaction tube was rinsed with EtOAc (3 × 5 mL) to complete the transfer. Then, volatiles were removed, and product **3** was purified by running a dry-loaded column chromatography.

### 3.3. Charge-Accelerated Aza-Claisen Rearrangement

#### 3.3.1. Synthesis and Characterization of Additives

*N*,*N*′−Bis [3,5−bis(trifluoromethyl)phenyl]−thiourea (Schreiner’s thiourea catalyst). The title compound was prepared according to a modified literature procedure [139]. A 4 mL-GC vial was charged with 3,5-bis(trifluoromethyl)aniline (573 mg, 2.50 mmol, 1.00 equiv.) and was dissolved in 0.25 mL of MeOH. A second 4 mL-GC vial was charged with 3,5-bis-(trifluoromethyl)phenyl isothiocyanate (678 mg, 2.50 mmol, 1.00 equiv.) and dissolved in 0.25 mL of MeOH. Both solutions were combined in a 50 mL round bottom flask, each vial rinsed with additional MeOH (0.25 mL), and the solution was stirred for 1 h at room temperature. Then, the solvent was evaporated to yield the product as a colorless solid (1.17 g, 2.33 mmol, 93%) without further purification. The NMR data closely match the ones previously reported in the literature [140]. m.p.: 159–161 °C. ^1^H NMR (DMSO-d_6_, 600 MHz): *δ* = 10.64 (s, 2H), 8.20 (s, 4H), 7.85 (s, 2H) ppm. ^13^C{^1^H} NMR (DMSO-d_6_, 151 MHz): *δ* = 180.7, 141.2 (2C), 130.4 (q, *J*_C–F_ = 33.1 Hz, 4C), 124.2 (d, *J*_C–F_ = 2.5 Hz, 4C), 123.2 (q, *J*_C–F_ = 272.6 Hz, 4C), 117.8 (t, *J*_C–F_ = 3.8 Hz, 2C) ppm. ^19^F NMR (DMSO-d_6_, 564 MHz): *δ* = –61.6 (s, 12F) ppm. IR (ATR): 1/*λ* = 3169 (w), 3047 (w), 2986 (w), 2201 (w), 2046 (w), 1800 (w), 1625 (w), 1552 (m), 1464 (m), 1371 (s), 1324 (m), 1277 (s), 1171 (s), 1123 (s), 1003 (m), 926 (m), 890 (m), 848 (m), 764 (w), 706 (s), 679 (s) cm^−1^. CI-MS (100 eV, Methane): *m*/*z* (%): 501 (100) [*M*+H]^+^, 500 (9) [*M*]^+^. EI-MS (70 eV): *m*/*z* (%): 501 (28) [*M*+H]^+^, 500 (81) [*M*]^+^, 481 (17), 272 (27), 252 (16), 229 (100), 213 (16), 163 (15), 69 (17). CHN: calcd (%) for C_17_H_8_F_12_N_2_S: C 40.81, H 1.61, N 5.60; found: C 40.80, H 2.07, N 5.53.

#### 3.3.2. Synthesis and Characterizations of the Starting Materials 2

*N*-Allylmorpholine (**2a**). A 50 mL round bottom flask equipped with a magnetic stirring bar was charged with morpholine (5.25 mL, 60.0 mmol, 3.00 equiv.) and cooled to 0 °C using an ice bath. At this temperature, allyl bromide (1.73 mL, 20.0 mmol, 1.00 equiv.) was added dropwise (**ATTENTION**: The cooling bath is mandatory as the reaction is highly exothermic). After addition, the reaction mixture was kept in the cooling bath and allowed to warm up to room temperature over the course of 21 h. Then, the reaction mixture was suspended between water and distilled Et_2_O (each 25 mL), and the organic phase was washed with water (2 × 25 mL). The organic phase was discarded as it contained impurities. The aqueous phases were combined and extracted with distilled Et_2_O (2 × 100 mL). The organic phases were combined and concentrated under reduced pressure to give the title compound as yellow liquid (559 mg, 4.4 mmol, 22%). The NMR data closely match the ones previously reported in the literature [141]. ^1^H NMR (CDCl_3_, 600 MHz): *δ* = 5.84 (ddtd, *J* = 16.8, 10.2, 6.6, 1.0 Hz, 1H), 5.20 (dq, *J* = 17.1, 1.5 Hz, 1H), 5.16 (ddq, *J* = 10.1, 2.1, 1.1 Hz, 1H), 3.72 (t, *J* = 4.7 Hz, 4H), 2.99 (dq, *J* = 6.6, 1.2 Hz, 2H), 2.44 (br s, 4H) ppm. ^13^C{^1^H} NMR (CDCl_3_, 151 MHz): *δ* = 134.7, 118.5, 67.1 (2C), 62.3, 53.7 (2C) ppm. IR (ATR): 1/*λ* = 3076 (w), 2957 (s), 2907 (s), 2854 (s), 2799 (s), 2333 (m), 2100 (m), 1992 (w), 1840 (w), 1643 (m), 1451 (m), 1422 (m), 1332 (m), 1291 (s), 1270 (m), 1239 (w), 1206 (w), 1117 (s), 1071 (m), 1034 (w), 1002 (s), 921 (s), 862 (s), 803 (m), 701 (w), 660 (w) cm^−1^. CI-MS (100 eV, Methane): *m*/*z* (%): 255 (76) [2*M*+H]^+^, 254 (13) [2*M*]^+^, 128 (55) [*M*+H]^+^, 127 (10) [*M*]^+^. EI-MS (70 eV): *m*/*z* (%): 128 (3) [*M*+H]^+^, 127 (5) [*M*]^+^, 126 (24), 114 (22), 113 (10), 100 (100), 57 (12), 56 (16).

*N*-Cinnamylmorpholine (**2b**). The title compound was prepared according to a modified literature procedure [142]. A 25 mL round bottom flask equipped with a magnetic stirring bar was charged with morpholine (440 µL, 5.00 mmol, 1.00 equiv.), MeCN (10 mL), and K_2_CO_3_ (0.76 g, 5.50 mmol, 1.10 equiv.) in the given order. Then, (*E*)-cinnamyl chloride (777 µL, 5.50 mmol, 1.10 equiv.) was added dropwise and the reaction mixture stirred at room temperature for 21 h. The reaction mixture was filtered over a plug of cotton and rinsed with MeCN (3 × 5 mL). The solvent was removed under reduced pressure and the crude product was purified by running a dry-loaded column chromatography (SiO_2_, pentane:EtOAc 1:0 → 9:1 → MeOH) to obtain the title compound as yellow oil (196 mg, 0.96 mmol, 19%). The NMR data closely match the ones previously reported in the literature [143]. R_f_ = 0.75 (MeOH), UV-active. ^1^H NMR (CDCl_3_, 600 MHz): *δ* = 7.39–7.35 (m, 2H), 7.33–7.29 (m, 2H), 7.25–7.21 (m, 1H), 6.54 (d, *J* = 15.8 Hz, 1H), 6.26 (dt, *J* = 15.9, 6.8 Hz, 1H), 3.74 (t, *J* = 4.7 Hz, 4H), 3.16 (dd, *J* = 6.8 Hz, 2H), 2.51 (br s, 4H) ppm. ^13^C{^1^H} NMR (CDCl_3_, 151 MHz): *δ* = 136.9, 133.6, 128.7 (2C), 127.7, 126.5 (2C), 126.2, 67.1 (2C), 61.6, 53.8 (2C) ppm. IR (ATR): 1/*λ* = 3872 (w), 3403 (w), 3026 (m), 2955 (m), 2912 (m), 2854 (s), 2804 (s), 2762 (m), 2326 (w), 2092 (w), 2009 (w), 1805 (w), 1679 (w), 1598 (w), 1494 (m), 1450 (s), 1393 (w), 1350 (m), 1325 (m), 1279 (m), 1205 (w), 1116 (s), 1070 (m), 1032 (m), 1003 (s), 969 (s), 904 (m), 866 (s), 788 (m), 740 (s), 692 (s) cm^−1^. CI-MS (100 eV, Methane): *m*/*z* (%): 204 (100) [*M*+H]^+^, 203 (29) [*M*]^+^. EI-MS (70 eV): *m*/*z* (%): 204 (33) [*M*+H]^+^, 203 (100) [*M*]^+^, 202 (28), 144 (13), 118 (10), 117 (57), 115 (33), 112 (79), 91 (16), 56 (17).

*N*-Prenylmorpholine (**2c**). The title compound was prepared according to a modified literature procedure [142]. A 100 mL round bottom flask equipped with a magnetic stirring bar was charged with morpholine (1.75 mL, 20.0 mmol, 1.00 equiv.), MeCN (50 mL), and K_2_CO_3_ (4.15 g, 30.0 mmol, 1.50 equiv.) in the given order. Then, 3,3-dimethylallyl bromide (2.31 mL, 20.0 mmol, 1.00 equiv.) was added dropwise and the reaction mixture stirred at room temperature for 21 h. The reaction mixture was filtered over a plug of cotton and rinsed with MeCN (5 × 5 mL). The solvent was removed under reduced pressure and the crude product was purified by vacuum distillation to yield the title compound at a head temperature of 87 °C as yellow oil (1.88 g, 12.1 mmol, 60%). The NMR data closely match the ones previously reported in the literature [144]. ^1^H NMR (CDCl_3_, 600 MHz): *δ* = 5.24 (tp, *J* = 7.1, 1.4 Hz, 1H), 3.71 (t, *J* = 4.7 HZ, 4H), 2.94 (d, *J* = 7.1 Hz, 2H), 2.44 (br s, 4H), 1.73 (d, *J* = 1.2 Hz, 3H), 1.65 (d, *J* = 1.4 Hz, 3H) ppm. ^13^C{^1^H} NMR (CDCl_3_, 151 MHz): *δ* = 135.9, 120.6, 67.2 (2C), 56.7, 53.8 (2C), 26.1, 18.2 ppm. IR (ATR): 1/*λ* = 3432 (w), 2960 (m), 2918 (m), 2855 (s), 2805 (s), 2684 (w), 2323 (w), 2087 (w), 1988 (w), 1805 (w), 1675 (w), 1449 (s), 1376 (m), 1320 (m), 1289 (m), 1242 (w), 1201 (w), 1116 (s), 1071 (m), 1033 (m), 1002 (s), 907 (m), 865 (s), 784 (m) cm^−1^. CI-MS (100 eV, Methane): *m*/*z* (%): not detectable. EI-MS (70 eV): *m*/*z* (%): 156 (2) [*M*+H]^+^, 155 (28) [*M*]^+^, 154 (10), 140 (14), 110 (13), 97 (10), 87 (83), 86 (32), 85 (15), 83 (23), 82 (19), 73 (12), 71 (15), 70 (12), 69 (100), 68 (12), 67 (17), 60 (12), 57 (81), 56 (55), 55 (55), 53 (11), 45 (16).

*N*-Allylpyrrolidine (**2d**). The title compound was prepared following an adjusted literature procedure [145]. A 100 mL round bottom flask equipped with a magnetic stirring bar was charged with pyrrolidine (3.34 mL, 40.0 mmol, 1.82 equiv.), which was dissolved in distilled Et_2_O (10 mL) and cooled to 0 °C using an ice bath. Then, allyl bromide (1.90 mL, 22.0 mmol, 1.00 equiv.) was added dropwise at 0 °C and stirred for 30 min at this temperature. Next, the ice bath was removed, and the reaction mixture was stirred for 21 h at room temperature. The mixture was filtered over a pad of Celite, which was rinsed with distilled Et_2_O. The organic phase was concentrated under reduced pressure and the crude product was purified by vacuum distillation. At a head temperature of 26 °C (oil bath 35 °C) the product was obtained as colorless oil and as a single fraction (152 mg, 1.36 mmol, 7%). The NMR data closely match the ones previously reported in the literature [145]. ^1^H NMR (CDCl_3_, 600 MHz): *δ* = 5.93 (ddtd, *J* = 16.8, 10.2, 6.6, 1.1 Hz, 1H), 5.18 (ddt, *J* = 17.1, 2.8, 1.4 Hz, 1H), 5.08 (ddq, *J* = 10.1, 2.1, 1.1 Hz, 1H), 3.09 (dq, *J* = 6.5, 1.3 Hz, 2H), 2.50 (tdd, *J* = 5.4, 2.6, 1.2 Hz, 4H), 1.78 (dddd, *J* = 6.7, 4.0, 2.9, 1.1 Hz, 4H) ppm. ^13^C{^1^H} NMR (CDCl_3_, 151 MHz): *δ* = 136.4, 116.7, 59.4, 54.2 (2C), 23.6 (2C) ppm. IR (ATR): 1/*λ* = 3435 (w), 3077 (w), 2963 (s), 2910 (s), 2876 (m), 2776 (s), 2324 (w), 2177 (w), 2085 (w), 2024 (w), 1989 (w), 1643 (m), 1460 (m), 1420 (m), 1347 (m), 1317 (m), 1263 (m), 1197 (m), 1143 (s), 1032 (w), 994 (s), 915 (s), 877 (s), 673 (w) cm^−1^. CI-MS (100 eV, Methane): *m*/*z* (%): not detectable. EI-MS (70 eV): *m*/*z* (%): 112 (1) [*M*+H]^+^, 111 (15) [*M*]+, 110 (19), 85 (61), 84 (28), 83 (100), 48 (12), 47 (25).

*N*-Allylpiperidine (**2e**). The title compound was prepared following an adjusted literature procedure [145]. A 100 mL round bottom flask equipped with a magnetic stirring bar was charged with piperidine (3.95 mL, 40.0 mmol, 1.82 equiv.), which was dissolved in distilled Et_2_O (10 mL) and cooled to 0 °C using an ice bath. Then, allyl bromide (1.90 mL, 22.0 mmol, 1.00 equiv.) was added dropwise at 0 °C and stirred for 30 min at this temperature; an additional 10 mL distilled Et_2_O was added as the reaction mixture was very viscous. Next, the ice bath was removed, and the reaction mixture was stirred for 21 h at room temperature. The mixture was filtered over a pad of Celite, which was rinsed with distilled Et_2_O. The organic phase was concentrated (rotary evaporator bath: 35 °C) under reduced pressure and the crude product was purified by vacuum distillation. At a head temperature of 35 °C the product was obtained as colorless oil and as a single fraction (1.38 g, 11.0 mmol, 55%). The NMR data closely match the ones previously reported in the literature [146]. ^1^H NMR (CDCl_3_, 600 MHz): *δ* = 5.88 (ddt, *J* = 16.9, 10.2, 6.6 Hz, 1H), 5.15 (dq, *J* = 17.1, 1.5 Hz, 1H), 5.11 (ddt, *J* = 10.1, 2.1, 1.1 Hz, 1H), 2.95 (dt, *J* = 6.6, 1.3 Hz, 2H), 2.36 (br s, 4H), 1.58 (m, 4H), 1.42 (m, 2H) ppm. ^13^C{^1^H} NMR (CDCl_3_, 151 MHz): *δ* = 135.8, 117.6, 62.8, 54.6 (2C), 26.1 (2C), 24.5 ppm. IR (ATR): 1/*λ* = 3877 (w), 3399 (w), 3077 (w), 3007 (w), 2932 (s), 2855 (s), 2783 (s), 2749 (s), 2323 (w), 2116 (w), 2000 (w), 1838 (w), 1643 (m), 1444 (m), 1385 (m), 1334 (m), 1299 (m), 1274 (m), 1202 (w), 1155 (m), 1111 (s), 1039 (m), 994 (s), 916 (s), 859 (m), 788 (m), 687 (w) cm^−1^. CI-MS (100 eV, Methane): *m*/*z* (%): not detectable. EI-MS (70 eV): *m*/*z* (%): 126 (4) [*M*+H]^+^, 125 (44) [*M*]^+^, 124 (61), 110 (23), 98 (100), 85 (35), 84 (21), 83 (47), 82 (15), 73 (15), 69 (14), 57 (18), 56 (11), 55 (26).

1-Allyl-4-phenylpiperazine (**2f**). The title compound was prepared according to a modified literature procedure [142]. A 25 mL round bottom flask equipped with a magnetic stirring bar was charged with 1-phenylpiperazine (822 mg, 5.00 mmol, 1.00 equiv.), MeCN (10 mL), and K_2_CO_3_ (760 g, 5.50 mmol, 1.10 equiv.) in the given order. Then, allyl bromide (0.47 mL, 5.50 mmol, 1.10 equiv.) was added dropwise and the reaction mixture stirred at room temperature for 4 days. The reaction mixture was filtered over a plug of cotton and rinsed with MeCN (3 × 5 mL). The solvent was removed under reduced pressure and the crude product was purified by running a dry-loaded column chromatography (SiO_2_, pentane:EtOAc 9:1 → 4:1) to obtain the title compound as yellow oil (677 mg, 3.35 mmol, 67%). The NMR data closely match the ones previously reported in the literature [147]. R_f_ = 0.22 (pentane:EtOAc 4:1), UV-active, smears. ^1^H NMR (CDCl_3_, 600 MHz): *δ* = 7.29–7.23 (m, 2H), 6.94 (m, 2H), 6.86 (m, 1H), 5.91 (ddt, *J* = 16.9, 10.2, 6.6 Hz, 1H), 5.23 (dq, *J* = 17.1, 1.5 Hz, 1H), 5.19 (ddt, *J* = 10.1, 2.1, 1.1 Hz, 1H), 3.22 (m, 4H), 3.07 (dt, *J* = 6.6, 1.3 Hz, 2H), 2.62 (m, 4H) ppm. ^13^C{^1^H} NMR (CDCl_3_, 151 MHz): *δ* = 151.5, 135.0, 129.2 (2C), 119.8, 118.4, 116.2 (2C), 62.0, 53.3 (2C), 49.3 (2C) ppm. IR (ATR): 1/*λ* = 3886 (w), 3416 (w), 3196 (w), 3066 (m), 3030 (w), 2941 (m), 2907 (m), 2882 (m), 2813 (s), 2327 (m), 2082 (m), 1995 (w), 1915 (w), 1821 (m), 1642 (w), 1597 (s), 1498 (s), 1451 (s), 1422 (m), 1382 (m), 1337 (s), 1299 (m), 1230 (s), 1139 (s), 1060 (w), 1003 (s), 922 (s), 878 (w), 814 (m), 756 (s), 690 (s) cm^−1^. CI-MS (100 eV, Methane): *m*/*z* (%): 203 (100) [*M*+H]^+^, 202 (77) [*M*]^+^. EI-MS (70 eV): *m*/*z* (%): 203 (57) [*M*+H]^+^, 202 (100) [*M*]^+^, 161 (12), 106 (14), 96 (12).

*N*-Benzyl-*N*-methylprop-2-en-1-amine (**2g**). The title compound was prepared following an adjusted literature procedure [145]. A 100 mL round bottom flask equipped with a magnetic stirring bar was charged with *N*-benzylmethylamine (4.85 g, 40.0 mmol, 1.82 equiv.), which was dissolved in distilled Et_2_O (10 mL) and cooled to 0 °C using an ice bath. Then, allyl bromide (1.90 mL, 22.0 mmol, 1.00 equiv.) was added dropwise at 0 °C and stirred for 30 min at this temperature. Next, the ice bath was removed, and the reaction mixture was stirred for 21 h at room temperature. The mixture was filtered over a pad of Celite, which was rinsed with distilled Et_2_O. The organic phase was concentrated (rotary evaporator bath: 35 °C) under reduced pressure and the crude product was purified by vacuum distillation. At a head temperature of 85 °C, the product was obtained as colorless oil (2.31 g, 14.3 mmol, 72%). The NMR data closely match the ones previously reported in the literature [141]. ^1^H NMR (CDCl_3_, 600 MHz): *δ* = 7.34–7.29 (m, 4H), 7.27–7.22 (m, 1H), 5.92 (ddt, *J* = 16.8, 10.2, 6.5 Hz, 1H), 5.20 (dq, *J* = 17.2, 1.5 Hz, 1H), 5.15 (ddt, *J* = 10.2, 2.2, 1.2 Hz, 1H), 3.50 (s, 2H), 3.03 (dt, *J* = 6.5, 1.3 Hz, 2H), 2.19 (s, 3H) ppm. ^13^C{^1^H} NMR (CDCl_3_, 151 MHz): *δ* = 139.2, 136.1, 129.2 (2C), 128.4 (2C), 127.1, 117.6, 61.8, 60.7, 42.2 ppm. IR (ATR): 1/*λ* = 3876 (w), 3418 (w), 3068 (m), 3028 (m), 2978 (m), 2920 (m), 2877 (m), 2834 (m), 2782 (s), 2323 (w), 2095 (w), 1999 (w), 1810 (m), 1643 (m), 1601 (w), 1493 (m), 1451 (s), 1365 (m), 1274 (m), 1251 (m), 1200 (m), 1132 (m), 1075 (m), 1026 (s), 993 (s), 917 (s), 859 (s), 821 (m), 737 (s), 697 (s) cm^−1^. CI-MS (100 eV, Methane): *m*/*z* (%): 162 (100) [*M*+H]^+^, 161 (32) [*M*]^+^. EI-MS (70 eV): *m*/*z* (%): 162 (100) [*M*+H]^+^, 161 (48) [*M*]^+^, 160 (44), 134 (15).

*N*,*N*-Dipropylprop-2-en-1-amine (**2h**). The title compound was prepared following an adjusted literature procedure [145]. A 100 mL round bottom flask equipped with a magnetic stirring bar was charged with dipropylamine (4.05 mL, 40.0 mmol, 1.82 equiv.), which was dissolved in distilled Et_2_O (10 mL) and cooled to 0 °C using an ice bath. Then, allyl bromide (1.90 mL, 22.0 mmol, 1.00 equiv.) was added dropwise at 0 °C and stirred for 30 min at this temperature. Next, the ice bath was removed, and the reaction mixture was stirred for 21 h at room temperature. The mixture was filtered over a pad of Celite, which was rinsed with distilled Et_2_O. The organic phase was concentrated (rotary evaporator bath: 35 °C) under reduced pressure and the crude product was purified by vacuum distillation. At a head temperature of 35 °C, the product was obtained as colorless oil and as a single fraction (1.44 g, 10.2 mmol, 51%). The NMR data closely match the ones previously reported in the literature [148]. ^1^H NMR (CDCl_3_, 600 MHz): *δ* = 5.87 (ddt, *J* = 16.8, 10.0, 6.5 Hz, 1H), 5.15 (dq, *J* = 17.1, 1.6 Hz, 1H), 5.09 (ddt, *J* = 10.1, 2.2, 1.2 Hz, 1H), 3.08 (m, 2H), 2.37 (m, 4H), 1.46 (m, 4H), 0.87 (t, *J* = 7.4 Hz, 6H) ppm. ^13^C{^1^H} NMR (CDCl_3_, 151 MHz): *δ* = 136.5, 116.9, 57.5, 56.0 (2C), 20.3 (2C), 12.1 (2C) ppm. IR (ATR): 1/*λ* = 3446 (w), 3077 (m), 2960 (s), 2874 (s), 2798 (s), 1985 (w), 1836 (w), 1688 (w), 1642 (w), 1461 (s), 1419 (m), 1381 (m), 1272 (m), 1188 (m), 1168 (m), 1075 (s), 1027 (m), 995 (m), 958 (m), 916 (s), 841 (w), 749 (w), 632 (w), 509 (w) cm^−1^. CI-MS (100 eV, Methane): *m*/*z* (%): 142 (9) [*M*+H]^+^, 141 (1) [*M*]^+^. EI-MS (70 eV): *m*/*z* (%): 142 (1) [*M*+H]^+^, 141 (6) [*M*]^+^, 119 (11), 112 (33).

For detailed preparative protocols and characterizing data for compounds **2i–n**, [141,142,145,149,150,151,152] see the Supporting Material.

#### 3.3.3. Synthesis and Characterization of the Products 3

2-Methyl-1-morpholinopent-4-en-1-one (**3aa**). The title compound was prepared following the GP2 using *N*-allylmorpholine (**2a**, 63.6 mg, 0.50 mmol, 1.00 equiv.), propionyl chloride (**1a**, 68.0 µL, 0.75 mmol, 1.50 equiv.), and Hünig’s base (76.0 µL, 0.50 mmol, 1.00 equiv.). After a dry-loaded column chromatography (SiO_2_, EtOAc) product **3aa** was obtained as yellow viscous oil (71 mg, 0.39 mmol, 77%). Repeating the reaction twice yielded 80% and 76%, respectively. Performing the same reaction on a 1 mmol scale yielded the product in 84% (153 mg, 0.84 mmol). The NMR data reported closely match the ones previously reported in the literature [62]. R_f_ = 0.51 (EtOAc), stains with KMnO4. ^1^H NMR (CDCl_3_, 600 MHz): *δ* = 5.71 (ddt, *J* = 17.1, 10.2, 7.0 Hz, 1H), 5.02 (dq, *J* = 17.2, 1.7 Hz, 1H), 4.98 (ddt, *J* = 10.2, 2.1, 1.1 Hz, 1H), 3.65–3.42 (m, 8H), 2.68 (sextet, *J* = 6.9 Hz, 1H), 2.38 (dtt, *J* = 13.6, 6.7, 1.4 Hz, 1H), 2.09 (m, 1H), 1.08 (d, *J* = 6.9 Hz, 3H) ppm. ^13^C{^1^H} NMR (CDCl_3_, 151 MHz): *δ* = 174.5, 136.0, 116.7, 67.1, 66.9, 46.1, 42.1, 38.1, 35.1, 17.3 ppm. IR (ATR): 1/*λ* = 3489 (w), 3272 (w), 3075 (w), 2970 (m), 2920 (m), 2856 (m), 2331 (w), 2078 (w), 1734 (w), 1638 (s), 1432 (s), 1364 (m), 1300 (w), 1268 (m), 1222 (s), 1154 (w), 1114 (s), 1068 (m), 1028 (s), 913 (s), 844 (m), 724 (w) cm^−1^. CI-MS (100 eV, Methane): *m*/*z* (%): 367 (14) [2*M*+H]^+^, 184 (100) [*M*+H]^+^, 183 (10) [*M*]^+^. EI-MS (70 eV): m/z (%): 367 (5) [2*M*+H]^+^, 184 (100) [*M*+H]^+^, 183 (37) [*M*]^+^, 114 (11), 86 (11).

2-Methyl-1-morpholino-3-phenylpent-4-en-1-one (**3ab**). The title compound was prepared following the GP2 using *N*-cinnamylmorpholine (**2b**, 102 mg, 0.5 mmol, 1.00 equiv.), propionyl chloride (**1a**, 66.0 µL, 0.75 mmol, 1.50 equiv.), and Hünig’s base (87.0 µL, 0.50 mmol, 1.00 equiv.). After a dry-loaded column chromatography (SiO_2_, pentane:EtOAc 1:1), product **3ab** was obtained as yellow, viscous oil (51.3 mg, 0.20 mmol, 40%) and as a mixture of diastereomers (4:1 determined by ^1^H NMR spectroscopy). The NMR data (for the major diastereomer) closely match the ones previously reported in the literature [124]. R_f_ = 0.33 (pentane:EtOAc 1:1), UV-active, stains with KMnO_4_. ^1^H NMR (CDCl_3_, 600 MHz): *δ* (mixture of diastereomers 4:1) = 7.34–7.15 (m, 5H), 6.04–5.94 {m, 1H; [6.01 (ddd, *J* = 17.1, 10.4, 7.8 Hz, 1H, major diastereomer)] + [6.01–5.95 (m, 1H, minor diastereomer)]}, 5.19–4.96 {m, 2H; [5.19–5.12 (m, 2H, minor diastereomer)] + [5.02 (dt, *J* = 10.4, 1.3 Hz, 1H) and 4.99 (dt, *J* = 17.1, 1.4 Hz, 1H), major diastereomer]}, 3.70–3.47 (m, 8H), 3.46–3.10 (m, 1H), 3.09–2.97 {m, 1H; [3.06 (dq, *J* = 9.9, 6.8 Hz, 1H, major diastereomer)] + [3.00 (dq, *J* = 10.3, 6.6 Hz, 1H, minor diastereomer)]}, 1.20–0.88 {m, 3H; [1.19 (d, *J* = 6.7 Hz, 3H, minor diastereomer)] + [0.92 (d, *J* = 6.7 Hz, 3H, major diastereomer)]} ppm. ^13^C{^1^H} NMR (CDCl_3_, 151 MHz): *δ* (major diastereomer) = 174.1, 141.8, 139.8, 127.7 (2C), 128.4 (2C), 126.8, 115.7, 67.1, 66.8, 53.4, 46.3, 42.2, 39.8, 16.8 ppm; *δ* (minor diastereomer) = 174.0, 143.1, 138.8, 128.6 (2C), 127.8 (2C), 126.7, 117.2, 66.4, 54.2, 46.1, 42.0, 40.2, 16.9 ppm. Note: For the minor diastereomer only 15 C were detected. Most likely the missing signal is overlayed by the signals of the major diastereomer. IR (ATR): 1/*λ* = 3481 (w), 3063 (w), 2972 (m), 2922 (m), 2858 (m), 2329 (w), 2076 (w), 1885 (w), 1757 (w), 1626 (s), 1436 (s), 1363 (w), 1300 (w), 1241 (s), 1150 (w), 1113 (s), 1070 (m), 1028 (s), 912 (m), 845 (m), 766 (m), 736 (m), 701 (s) cm^−1^. CI-MS (100 eV, Methane): *m*/*z* (%): 260 (100) [*M*+H]^+^, 259 (5) [*M*]^+^. EI-MS (70 eV): *m*/*z* (%): 519 (9) [2*M*+H]^+^, 260 (100) [*M*+H]^+^, 259 (46) [*M*]^+^, 258 (41), 245 (14), 244 (84), 118 (10), 117 (34), 115 (15), 114 (10).

2,3,3-Trimethyl-1-morpholinopent-4-en-1-one (**3ac**). The title compound was prepared following the GP2 using *N*-prenylmorpholine (**2c**, 77.6 mg, 0.50 mmol, 1.00 equiv.), propionyl chloride (**1a**, 66.0 µL, 0.75 mmol, 1.50 equiv.), and Hünig’s base (87.0 µL, 0.50 mmol, 1.00 equiv.). After a dry-loaded column chromatography (SiO_2_, pentane:EtOAc 1:1) product **3ac** was obtained as yellow viscous oil (46.3 mg, 0.22 mmol, 44%). R_f_ = 0.26 (pentane:EtOAc 1:1), stains with KMnO_4_. ^1^H NMR (CDCl_3_, 600 MHz): *δ* = 5.90 (dd, *J* = 17.4, 10.9 Hz, 1H), 4.97 (m, 1H), 4.95 (dd, *J* = 10.9, 1.3 Hz, 1H), 3.68–3.48 (m, 8H), 2.62 (q, *J* = 6.9 Hz, 1H), 1.07 (s, 3H), 1.06 (s, 3H), 1.05 (s, 3H) ppm. ^13^C{^1^H} NMR (CDCl_3_, 151 MHz): *δ* = 174.2, 146.6, 111.7, 67.2, 66.9, 47.0, 42.5, 42.1, 39.5, 24.8, 24.2, 13.7 ppm. IR (ATR): 1/*λ* = 3491 (w), 3263 (w), 3081 (w), 2966 (m), 2858 (m), 2325 (w), 2161 (w), 1934 (w), 1731 (w), 1635 (s), 1427 (s), 1363 (m), 1300 (w), 1265 (m), 1234 (s), 1115 (s), 1073 (m), 1025 (s), 946 (w), 911 (s), 843 (m), 779 (w), 684 (w) cm^−1^. CI-MS (100 eV, Methane): *m*/*z* (%): 212 (100) [*M*+H]^+^, 211 (6) [*M*]^+^. EI-MS (70 eV): *m*/*z* (%): 212 (100) [*M*+H]^+^, 211 (19) [*M*]^+^, 196 (19), 143 (17), 142 (30), 114 (12), 87 (10), 69 (13), 55 (11). HRMS (ESI): *m*/*z* calcd for C_12_H_21_O_2_N+H^+^ [*M*+H]^+^: 212.1645; found: 212.1641. 

2-Methyl-1-(pyrrolidin-1-yl)pent-4-en-1-one (**3ad**). The title compound was prepared following the GP2 using *N*-allylpyrrolidine (**2d**, 55.6 mg, 0.51 mmol, 1.00 equiv.), propionyl chloride (**1a**, 66.0 µL, 0.76 mmol, 1.50 equiv.), and Hünig’s base (88.0 µL, 0.51 mmol, 1.00 equiv.). After a dry-loaded column chromatography (SiO_2_, EtOAc) product **3ad** was obtained as yellow viscous oil (56.5 mg, 0.34 mmol, 68%). However, ^1^H NMR analysis showed the presence of propionic acid. Therefore, the crude product was dissolved in distilled Et_2_O (20 mL) and washed with 1 M NaOH_(aq.)_ (3 × 10 mL) to yield the pure product as yellow viscous oil (31.2 mg, 0.19 mmol, 37%) after the solvent was removed under reduced pressure. The NMR data closely match the ones previously reported in the literature [153]. R_f_ = 0.29 (EtOAc), stains with KMnO_4_. ^1^H NMR (CDCl_3_, 600 MHz): *δ* = 5.75 (dddd, *J* = 16.8, 10.1, 7.6, 6.5 Hz, 1H), 5.04 (dq, *J* = 17.0, 1.6 Hz, 1H), 4.98 (ddt, *J* = 10.1, 2.1, 1.1 Hz, 1H), 3.52–3.33 (m, 4H), 2.57 (sextet, *J* = 6.9 Hz, 1H), 2.41 (dddt, *J* = 13.9, 7.6, 6.5, 1.4 Hz, 1H), 2.17–2.05 (m, 1H), 1.96–1.89 (m, 2H), 1.88–1.77 (m, 2H), 1.10 (d, *J* = 6.8 Hz, 3H) ppm. ^13^C{^1^H} NMR (CDCl_3_, 151 MHz): *δ* = 174.6, 136.5, 116.5, 46.5, 45.8, 38.2, 38.1, 26.3, 24.4, 17.0 ppm. IR (ATR): 1/*λ* = 3477 (w), 3074 (w), 2970 (m), 2874 (m), 2328 (w), 2091 (w), 2004 (w), 1890 (w), 1756 (w), 1633 (s), 1430 (s), 1373 (w), 1334 (m), 1256 (w), 1225 (w), 1188 (w), 1114 (w), 1034 (w), 994 (w), 912 (m), 866 (w), 804 (w), 746 (w), 692 (w), 668 (w) cm^−1^. CI-MS (100 eV, Methane): *m*/*z* (%): 168 (100) [*M*+H]^+^, 167 (6) [*M*]+. EI-MS (70 eV): *m*/*z* (%): 168 (85) [*M*+H]^+^, 167 (100) [*M*]^+^, 166 (14), 152 (65), 138 (10), 126 (29), 125 (37), 124 (23), 98 (70), 97 (37), 72 (18), 71 (15), 70 (49), 69 (31), 68 (13), 56 (28), 55 (51), 53 (10).

2-Methyl-1-(piperidin-1-yl)pent-4-en-1-one (**3ae**). The title compound was prepared following the GP2 using *N*-allylpiperidine (**2e**, 63.3 mg, 0.51 mmol, 1.00 equiv.), propionyl chloride (**1a**, 66.0 µL, 0.76 mmol, 1.50 equiv.), and Hünig’s base (88.0 µL, 0.51 mmol, 1.00 equiv.). After a dry-loaded column chromatography (SiO_2_, pentane:EtOAc 1:1) product **3ae** was obtained as yellow oil (21.0 mg, 0.12 mmol, 23%). R_f_ = 0.55 (pentane:EtOAc 1:1), UV-active, stains with KMnO_4_. ^1^H NMR (CDCl_3_, 600 MHz): *δ* = 5.76 (dddd, *J* = 16.8, 10.1, 7.6, 6.4 Hz, 1H), 5.04 (dq, *J* = 17.1, 1.6 Hz, 1H), 4.99 (ddt, *J* = 10.2, 2.1, 1.1 Hz, 1H), 3.55 (dddd, *J* = 40.4, 13.1, 6.8, 4.4 Hz, 2H), 3.50–3.37 (m, 2H), 2.75 (sextet, *J* = 6.9 Hz, 1H), 2.42 (dtt, *J* = 14.4, 6.6, 1.4 Hz, 1H), 2.22–2.03 (m, 1H), 1.64 (pd, *J* = 5.7, 1.8 Hz, 2H), 1.59–1.45 (m, 4H), 1.10 (d, *J* = 6.8 Hz, 3H) ppm. ^13^C{^1^H} NMR (CDCl_3_, 151 MHz): *δ* = 174.2, 136.6, 116.4, 46.7, 43.0, 38.3, 35.4, 26.9, 25.9, 24.8, 17.5 ppm. IR (ATR): 1/*λ* = 3485 (w), 3075 (w), 2931 (s), 2856 (m), 2166 (m), 2010 (w), 1757 (w), 1635 (s), 1436 (s), 1366 (m), 1243 (s), 1215 (s), 1122 (m), 1007 (s), 952 (w), 910 (m), 852 (w), 803 (w), 719 (w) cm^−1^. CI-MS (100 eV, Methane): *m*/*z* (%): 363 (7) [2*M*+H]^+^, 182 (100) [*M*+H]^+^, 181 (7) [*M*]^+^. EI-MS (70 eV): *m*/*z* (%): 182 (100) [*M*+H]^+^, 181 (73) [*M*]^+^, 166 (33), 140 (24), 139 (28), 138 (20), 112 (33), 111 (26), 86 (13), 84 (35), 69 (31), 56 (11). HRMS (ESI): *m*/*z* calcd for C_11_H_19_NO+Na^+^ [*M*+Na]^+^: 204.1359; found: 204.1358. 

2-Methyl-1-(4-phenylpiperazin-1-yl)pent-4-en-1-one (**3af**). The title compound was prepared following the GP2 using 1-allyl-4-phenylpiperazine (**2f**, 101 mg, 0.50 mmol, 1.00 equiv.), propionyl chloride (**1a**, 66.0 µL, 0.75 mmol, 1.50 equiv.), and Hünig’s base (87.0 µL, 0.50 mmol, 1.00 equiv.). After a dry-loaded column chromatography (SiO_2_, pentane:EtOAc 4:1 → 1:1) product **3af** was obtained as yellow oil (72.2 mg, 0.28 mmol, 56%). R_f_ = 0.54 (pentane:EtOAc 1:1), stains with KMnO_4_. ^1^H NMR (CDCl_3_, 600 MHz): *δ* = 7.31–7.26 (m, 2H), 6.96–6.89 (m, 3H), 5.78 (dddd, *J* = 16.8, 10.2, 7.6, 6.5 Hz, 1H), 5.07 (dq, *J* = 17.1, 1.6 Hz, 1H), 5.03 (ddt, *J* = 10.2, 2.1, 1.1 Hz, 1H), 3.86–3.74 (m, 2H), 3.73–3.63 (m, 2H), 3.22–3.10 (m, 4H), 2.80 (sextet, *J* = 6.9 Hz, 1H), 2.46 (dtt, *J* = 14.9, 6.7, 1.4 Hz, 1H), 2.16 (m, 1H), 1.16 (d, *J* = 6.8 Hz, 3H) ppm. ^13^C{^1^H} NMR (CDCl_3_, 151 MHz): *δ* = 174.5, 151.1, 136.2, 129.4 (2C), 120.7, 116.8, 116.7 (2C), 50.1, 49.7, 45.6, 41.8, 38.3, 35.5, 17.5 ppm. IR (ATR): 1/*λ* = 3478 (w), 3275 (w), 3066 (w), 2972 (m), 2910 (m), 2819 (m), 2329 (w), 2084 (w), 1922 (w), 1732 (w), 1639 (s), 1598 (s), 1497 (s), 1435 (s), 1375 (m), 1336 (m), 1276 (m), 1225 (s), 1154 (m), 1095 (w), 1021 (s), 909 (s), 757 (s), 693 (m) cm^−1^. CI-MS (100 eV, Methane): *m*/*z* (%): 259 (100) [*M*+H]^+^, 258 (11) [*M*]^+^. EI-MS (70 eV): *m*/*z* (%): 259 (47) [*M*+H]^+^, 258 (100) [*M*]^+^, 161 (15), 132 (52), 120 (14), 56 (11). HRMS (ESI): *m*/*z* calcd for C_16_H_22_ON_2_+Na^+^ [*M*+Na]^+^: 281.1624; found: 281.1621. 

*N*-Benzyl-*N*,2-dimethylpent-4-enamide (**3ag**). The title compound was prepared following the GP2 using *N*-benzyl-*N*-methylprop-2-en-1-amine (**2g**, 80.3 mg, 0.50 mmol, 1.00 equiv.), propionyl chloride (**1a**, 66.0 µL, 0.75 mmol, 1.50 equiv.), and Hünig’s base (87.0 µL, 0.50 mmol, 1.00 equiv.). After two dry-loaded column chromatographies (SiO_2_, 1st: pentane:EtOAc 2:1 → 1:1, 2nd: 9:1 → 6:1 → 4:1 → 2:1) product was obtained as yellow oil (31.6 mg, 0.15 mmol, 29%). *Note*: The NMR spectra were recorded at an elevated temperature as product **3ag** was observed to be a mixture of rotamers at room temperature. R_f_ = 0.26 (pentane:EtOAc 4:1), stains with KMnO_4_. ^1^H NMR (100 °C, DMSO-d_6_, 400 MHz): *δ* = 7.37–7.30 (m, 2H), 7.29–7.18 (m, 3H), 5.78 (dq, *J* = 16.9 Hz, 7.8 Hz, 1H), 5.07–4.94 (m, 2H), 4.63–4.48 (m, 2H), 2.95–2.83 (m, 4H), 2.35 (m, 1H), 2.08 (m, 1H), 1.06 (d, *J* = 6.8 Hz, 3H) ppm. ^13^C{^1^H} NMR (100 °C, DMSO-d_6_, 101 MHz): *δ* = 174.7, 137.5, 135.9, 127.9 (2C), 126.6, 126.4 (2C), 115.5, 37.3, 34.2, 33.8, 16.5 ppm. IR (ATR): 1/*λ* = 3486 (w), 3276 (w), 3068 (w), 3029 (w), 2972 (m), 2929 (m), 2328 (w), 2092 (w), 1883 (w), 1759 (w), 1721 (w), 1639 (s), 1450 (s), 1407 (m), 1355 (w), 1256 (w), 1202 (w), 1086 (m), 1027 (w), 994 (m), 913 (m), 810 (w), 732 (s), 699 (s) cm^−1^. CI-MS (100 eV, Methane): *m*/*z* (%): 435 (22) [2*M*+H]^+^, 434 (1) [2*M*]^+^, 218 (100) [*M*+H]^+^, 217 (7) [*M*]^+^. EI-MS (70 eV): *m*/*z* (%): 218 (46) [*M*+H]^+^, 217 (85) [*M*]^+^, 216 (28), 202 (30), 176 (10), 175 (15), 174 (48), 126 (20), 120 (21), 118 (19), 92 (11), 91 (100), 69 (21), 65 (14). HRMS (ESI): *m*/*z* calcd for C_14_H_19_NO+Na^+^ [*M*+Na]^+^: 240.1359; found: 240.1355. 

2-Methyl-*N*,*N*-dipropylpent-4-enamide (**3ah**). The title compound was prepared following the GP2 using *N*,*N*-diprop-2-en-1-amine (**2h**, 86.7 mg, 0.61 mmol, 1.00 equiv.), propionyl chloride (**1a**, 80.0 µL, 0.92 mmol, 1.50 equiv.), and Hünig’s base (107 µL, 0.61 mmol, 1.00 equiv.). After a dry-loaded column chromatography (SiO_2_, pentane:EtOAc 9:1) product **3ah** was obtained as yellow oil (55.2 mg, 0.28 mmol, 46%). R_f_ = 0.34 (pentane:EtOAc 9:1), stains with KMnO_4_. ^1^H NMR (CDCl_3_, 600 MHz): *δ* = 5.72 (dddd, *J* = 16.9, 10.1, 7.7, 6.5 Hz, 1H), 5.02 (dq, *J* = 17.0, 1.5 Hz, 1H), 4.95 (ddt, *J* = 10.2, 2.1, 1.0 Hz, 1H), 3.29 (m, 1H), 3.23–3.09 (m, 4H), 2.65 (sextet, *J* = 6.9 Hz, 1H), 2.39 (dddt, *J* = 14.0, 7.7, 6.5, 1.4 Hz, 1H), 2.08 (dddt, *J* = 14.0, 7.7, 6.6, 1.1 Hz, 1H), 1.60–1.45 (m, 4H), 1.08 (dd, *J* = 6.8, 0.7 Hz, 3H), 0.88 (t, *J* = 7.4 Hz, 3H), 0.84 (t, *J* = 7.4 Hz, 3H) ppm. ^13^C{^1^H} NMR (CDCl_3_, 151 MHz): *δ* = 175.8, 136.4, 116.5, 49.6, 47.9, 38.8, 35.8, 22.9, 21.1, 17.9, 11.4, 11.3 ppm. IR (ATR): 1/*λ* = 3481 (w), 3266 (w), 3076 (w), 2964 (s), 2933 (m), 2875 (m), 2326 (w), 2087 (w), 1999 (w), 1838 (w), 1761 (w), 1637 (s), 1429 (s), 1374 (m), 1301 (w), 1234 (m), 1216 (m), 1098 (m), 998 (m), 910 (m), 749 (m), 671 (w) cm^−1^. CI-MS (100 eV, Methane): *m*/*z* (%): 198 (100) [*M*+H]^+^, 197 (13) [*M*]^+^. EI-MS (70 eV): *m*/*z* (%): 395 (2) [2*M*+H]^+^, 198 (100) [*M*+H]^+^, 197 (37) [*M*]^+^, 168 (14), 126 (11), 72 (27), 69 (15). HRMS (ESI): *m*/*z* calcd for C_12_H_23_NO+Na^+^ [*M*+Na]^+^: 220.1672; found: 220.1668. 

1-Morpholinopent-4-en-1-one (**3ba**). The title compound was prepared following the GP2 using *N*-allylmorpholine (**2a**, 63.3 mg, 0.50 mmol, 1.00 equiv.), acetyl chloride (**1b**, 54.0 µL, 0.75 mmol, 1.50 equiv.), and Hünig’s base (87.0 µL, 0.50 mmol, 1.00 equiv.). After a dry-loaded column chromatography (SiO_2_, pentane:EtOAc 1:1) product **3ba** was obtained as yellow oil (14.0 mg, 0.08 mmol, 17%). Keeping everything the same but using acetyl bromide (55.0 µL, 0.75 mmol, 1.50 equiv.) instead of acetyl chloride increased the yield slightly (17.0 mg, 0.10 mmol, 20%). The NMR data closely match the ones previously reported in the literature [154]. R_f_ = 0.33 (pentane:EtOAc 1:1), stains with KMnO_4_. ^1^H NMR (CDCl_3_, MHz): *δ* = 5.85 (m, 1H), 5.06 (m, 1H), 5.00 (m, 1H), 3.69–3.64 (m, 4H), 3.63–3.59 (m, 2H), 3.49–3.43 (m, 2H), 2.40 (m, 4H) ppm. ^13^C{^1^H} NMR (CDCl_3_, 151 MHz): *δ* = 171.1, 137.4, 115.5, 67.1, 66.8, 46.1, 42.1, 32.4, 29.3 ppm. IR (ATR): 1/*λ* = 3489 (w), 3273 (w), 3075 (w), 2966 (m), 2916 (m), 2856 (m), 2326 (w), 2225 (w), 2110 (w), 1761 (w), 1639 (s), 1431 (s), 1365 (w), 1269 (m), 1224 (s), 1113 (s), 1067 (m), 1025 (m), 961 (m), 913 (s), 848 (m), 799 (w), 737 (w) cm^−1^. CI-MS (100 eV, Methane): *m*/*z* (%): 339 (5) [2*M*+H]^+^, 170 (100) [*M*+H]^+^, 169 (9) [*M*]^+^. EI-MS (70 eV): *m*/*z* (%): 339 (1) [2*M*+H]^+^, 170 (100) [*M*+H]^+^, 169 (92) [*M*]^+^, 140 (20), 126 (19), 114 (62), 88 (15), 87 (12), 86 (39), 70 (16), 57 (44), 56 (33), 55 (52).

### 3.4. Belluš–Claisen-Type Rearrangement

#### 3.4.1. Synthesis and Characterization of the Starting Materials

*N*-Benzylproline (**S4a**). The title compound was prepared following a modified literature procedure [155]. A detailed preparative protocol and the characterizing data can be found in the Supplementary Material. Preparation and characterization data of additional starting materials **S1**, **S2**, **S3a** and isolated side products **S3b** can be found in the Supplementary Material [156,157,158,159,160].

*N*-Benzyl-2-vinylpyrrolidine (**4**). The title compound was prepared according to a modified literature procedure [136]. A detailed preparative protocol and the characterizing data can be found in the Supporting Information.

#### 3.4.2. Synthesis and Characterization of the Product

1-Benzyl-3-methyl-1,3,4,7,8,9-hexahydro-2H-azonin-2-one (**5**). The title compound was prepared following the GP2 using *N*-benzyl-2-vinylpyrrolidine (**4**, 97.1 mg, 0.52 mmol, 1.00 equiv.), propionyl chloride (68.0 µL, 0.78 mmol, 1.50 equiv.), and Hünig’s base (90.0 µL, 0.52 mmol, 1.00 equiv.). After a dry-loaded column chromatography (SiO_2_, pentane:EtOAc 9:1) product **5** was obtained as yellow oil (48.7 mg, 0.20 mmol, 39%). R_f_ = 0.23 (pentane:EtOAc 9:1), stains with I_2_@SiO_2_. ^1^H NMR (CDCl_3_, 600 MHz): *δ* = 7.32–7.27 (m, 2H), 7.23 (m, 1H), 7.20–7.16 (m, 2H), 5.65 (ddd, *J* = 15.8, 10.7, 5.2 Hz, 1H), 5.44–5.34 (m, 2H), 3.91 (d, *J* = 15.0 Hz, 1H), 3.57 (dd, *J* = 14.6, 10.2 Hz, 1H), 2.99 (dd, *J* = 14.6, 5.3 Hz, 1H), 2.71 (dtd, *J* = 13.2, 7.7, 5.4 Hz, 1H), 2.36 (ddd, *J* = 10.6, 6.5, 3.3 Hz, 1H), 2.19 (q, *J* = 11.4 Hz, 1H), 2.11 (ddd, *J* = 12.3, 5.2, 2.2 Hz, 1H), 2.09–1.94 (m, 2H), 1.71 (m, 1H), 1.22 (dd, *J* = 6.6, 1.0 Hz, 3H) ppm. ^13^C{^1^H} NMR (CDCl_3_, 151 MHz): *δ* = 176.4, 138.1, 132.9, 131.2, 128.6 (2C), 128.1 (2C), 127.2, 47.2, 44.7, 41.0, 37.9, 31.9, 27.9, 19.0 ppm. IR (ATR): 1/*λ* = 3473 (w), 2930 (s), 2863 (m), 2327 (w), 2237 (w), 2160 (w), 2117 (w), 1891 (w), 1759 (w), 1621 (s), 1493 (m), 1452 (s), 1418 (s), 1361 (m), 1269 (w), 1235 (m), 1186 (s), 1142 (m), 1079 (m), 1030 (w), 982 (s), 919 (w), 872 (w), 802 (m), 731 (s), 700 (s) cm^−1^. CI-MS (100 eV, Methane): *m*/*z* (%): 244 (100) [*M*+H]^+^, 243 (7) [*M*]^+^. EI-MS (70 eV): *m*/*z* (%): 244 (24) [*M*+H]^+^, 243 (12) [*M*]+, 242 (8), 174 (10), 152 (79), 151 (17), 124 (16), 91 (100), 65 (10), 55 (11). HRMS (ESI): *m*/*z* calcd for C_16_H_21_NO [*M*]^+^: 243.1623; found: 243.1624.

## Figures and Tables

**Table 1:** Optimization of the mechanochemical synthesis of amide **3aa**.

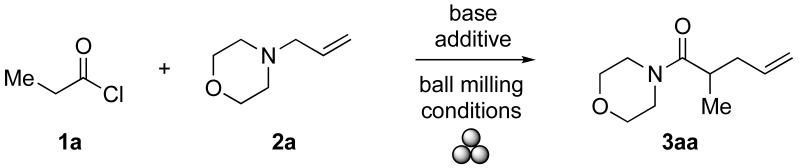				
Entry ^a^	Equiv. (1a)	Base (equiv.)	*t* [min]	*Y* (3aa) [%] ^b^
1	1.2	-	60	3
2	1.2	K_2_CO_3_ (1.0)	60	-
3	1.2	Cs_2_CO_3_ (1.0)	60	-
4	1.2	LiOH	60	9
5	1.2	NEt_3_	60	11
6	1.2	DBU	60	-
7	1.2	DIPEA (1.0)	60	35 (40) ^c^
8	1.2	DIPEA (0.5)	60	34 (1) ^d^
9	1.2	DIPEA (1.0)	60	33 (6) ^d^
10	1.2	DIPEA (1.5)	60	18 (4) ^d^
11	1.2	DIPEA (1.0)	15	27
12	1.2	DIPEA (1.0)	30	58
13	1.2	DIPEA (1.0)	120	42
14	1.2	DIPEA (1.0)	60	24 ^e^
15	1.0	DIPEA (1.0)	30	20
16	1.5	DIPEA (1.0)	30	69
17	2.0	DIPEA (1.0)	30	67
18	1.5	DIPEA (1.0)	30	51 ^f^
**19**	**1.5**	**DIPEA (1.0)**	**30**	**77 ^g^**
**20**	**1.5**	**DIPEA (1.0)**	**30**	**76 ^h^ 80 ^h^ (84) ^i^**
21 ^j^	1.5	DIPEA (1.0)	30	– (6) ^k^
22 ^l^	1.5	DIPEA (1.0)	30	82

^a^ Standard conditions: stainless steel jar, 10 mL, 1 ball (10 mm in Ø), 0.5 mmol amine **2a**, base, and propionyl chloride (**1a**) added in the given order, 25 Hz, 60 min; ^b^ determined after column chromatography; ^c^ ZrO_2_-Y jar; ^d^ reaction performed twice; ^e^ 3 balls (7 mm in Ø); ^f^ after amine **2a** and DIPEA first cooled in liquid nitrogen, then add **1a**; ^g^ keeping the parts of the milling container as close as possible together, add **1a** through the gap, and close immediately to reduce loss of the volatile ketene formed; ^h^ double repetition of entry 19; ^i^ repetition of entry 19 on a 1 mmol scale; ^j^ following GP3: amine **2a** dissolved in 5 mL DCM, then addition of DIPEA and propionyl chloride, rt, 30 min; ^k^ following GP3, 120 min; ^l^ following GP3, neat.

## Data Availability

The data presented in this study are available on request from the corresponding author.

## Supplementary Materials

The following supporting information can be downloaded at: https://www.mdpi.com/article/10.3390/molecules28020807/s1, general procedures; extended optimization tables (Tables S1–S5); schemes of unsuccessful reactions (Schemes S1 and S2); tested synthetic routes for the starting material synthesis in the Belluš–Claisen-type rearrangement (Scheme S3); synthesis of tested starting materials for the Belluš–Claisen-type rearrangement but not essential to the study; NMR copies of the prepared compounds (Figures S1–S66).

## Sample Availability

If required small quantities for (further) analysis from the prepared compounds are available from the authors upon request.
