# Supplementary material for: Reshuffle Bonds by Ball Milling: A Mechanochemical Protocol for Charge-Accelerated Aza-Claisen Rearrangements
Source: Molecules. 2023 Jan 13;28(2):807. doi: 10.3390/molecules28020807 (PMC9860570; doi:10.3390/molecules28020807)
Supplement: Supplementary file 1 [file molecules-28-00807-s001.zip › molecules-2120629-supplementary.pdf]

Supplementary material

for

Reshuffle bonds by ball milling: a mechanochemical protocol for charge-accelerated aza-Claisen rearrangements

Christian Schumacher [1], Lieselotte Fritz [1,†], Lena M. Hanek [1,†], Vitali Sidorin [1,†], Daniel Brüx, [1,†] and Carsten Bolm [1,*]

[1] Institute of Organic Chemistry, RWTH Aachen University, Landoltweg 1, D-52074 Aachen, Germany

* Correspondence: carsten.bolm@oc.rwth-aachen.de

[†] These authors contributed equally to this work and are listed in chronological order.

Table of Contents

1 General Information **S2**
2 General Procedures **S4**
3 Charge-accelerated Aza-Claisen Rearrangement **S5**
3.1 Optimization Tables **S5**
3.1.1 Base Screening S5
3.1.2 Optimization of Hünig's Base Amount S6
3.1.3 Optimization of Ball Milling Conditions S6
3.1.4 Optimization of Propionyl Chloride Amount S7
3.1.5 Additional Additive Screening S8
3.2 Synthesis and Characterization of Additives **S9**
3.3 Synthesis and Characterization of the Starting Materials **S10**
3.4 Synthesis and Characterization of the Products **S21**
3.5 Unsuccessful Reactions **S28**
3.5.1 Varying the Acyl Chloride S28
3.5.2 Varying the Allylamine S29
4 Extension towards a Charge-accelerated Belluš-Claisen Type Rearrangement **S30**
4.1 Considered and Tested Synthetic Routes **S30**
4.2 Synthesis and Characterization of the Starting Materials **S31**
4.3 Synthesis and Characterization of the Product **S37**
5 References **S38**
6 Copy of NMR Spectra **S40**

1 General Information

Chemicals

Unless otherwise stated all chemicals used were commercially available and were used as received.

Chromatography

Solvents for column chromatography were of technical grade and distilled prior to use. The stated eluents are always understood as volumetric ratios *v*/*v*. The used stationary phase was always silica gel [Silica 60 M (0.04–0.063 mm), purchased from MACHERY-NAGEL].

Thin layer chromatography (TLC) was performed with silica coated alumina plates [TLC Silica gel 60 F254 from Merck] and the products were visualized using UV-light (λ = 254 nm). As many of the substances prepared in this study are UV-inactive they were visualized either by dipping the TLC plate in an aqueous solution of $KMnO_4$ [1.5 g $KMnO_4$, 10 g K_2CO_3, and 1.25 mL 10% $NaOH_{(aq.)}$ were dissolved in 200 mL water] and heating of the stained TLC plate with a heat gun until dryness, if necessary, or by putting the TLC plates in an iodine chamber (1 g I_2 and 100 g SiO_2 were shaken until a homogenous powder was observed).

Melting point

Melting points (m.p.) were determined as melting range (range between solidus and liquidus temperature) using a Büchi melting point apparatus M-560, open-end capillaries, a heating rate of 5 °C·min^{-1} and are uncorrected.

Nuclear Magnetic Resonance (NMR) Spectroscopy

NMR measurements were performed either on a Varian VNMRS 600 or Bruker Avance Neo 400 spectrometer. If not stated otherwise, all NMR spectra were recorded at room temperature (25 °C). ^{13}C NMR measurements were conducted with proton broad band decoupling indicated as ^{13}C{^{1}H}. The spectra were processed and analyzed using the program MestReNova.[1] Proton and carbon NMR spectra were referenced to the non-deuterated residual solvent signal ($CHCl_3$: ^{1}H NMR: δ = 7.26 ppm, $CDCl_3$: ^{13}C{^{1}H} NMR: δ = 77.16 ppm; DMSO: ^{1}H NMR: δ = 2.50 ppm, DMSO-d$_6$: ^{13}C{^{1}H} NMR: δ = 39.52 ppm; CH_3CN: δ = 1.94 ppm).[2] ^{19}F spectra were referenced using the absolute frequency of the lock signal of the ^{2}H resonance signal of the used deuterated solvent. Chemical shifts (δ) are reported in ppm (parts per million) and the signals are reported from low to high field. The multiplicity of the peaks is reported as br (broad), s (singlet), d (doublet), t (triplet), q (quartet), p (pentet), m (multiplet) and/or combinations thereof. The spin-spin coupling constants (*J*) are reported in Hz (hertz).

Infrared (IR) spectroscopy

IR spectra were recorded neat on a PerkinElmer Spectrum 100 FT-IR spectrometer with an attached UATR device with a KRS-5 crystal. IR bands are reported with their corresponding wavenumber 1/λ given in cm^{-1} (in decreasing order) and the relative intensity of transmission [strong (s), medium(m), weak (w)].

Mass spectrometry (MS)

Mass spectra were recorded on a Finnigan SSQ 7000 mass spectrometer [electron ionization (EI), 70 eV; chemical ionization (CI), methane, 100 eV]. The signals are given according to their *m*/*z* values and their relative intensity is reported in parenthesis. High resolution mass (HRMS) spectra were recorded

as ESI (electrospray ionization, positive mode) spectra on a ThermoFisher Scientific LTQ Orbitrap XL mass spectrometer.

Elemental analysis (CHN)

CHN analysis was performed either on a Elementar varioEL or Elementar varioEL cube apparatus. The percentage of carbon (C), hydrogen (H), and nitrogen (N) was calculated for a defined compound and compared with the determined amount of the sample.

Mechanochemical reactions

All mechanochemical reactions were performed on a Retsch mixer mill MM400. The milling containers and balls used were always of the same material. For this purpose, stainless steel, or yttrium stabilized ZrO_2 were used. The milling containers used explicitly had a volume of V = 10 mL.

2 General Procedures

General Procedure 1 (GP1) – Optimization

A milling container of a chosen material equipped with the chosen number of milling balls and chosen diameter was charged with *N*-allylmorpholine (**2a**, 63.6 mg, 0.50 mmol, 1.00 equiv.). Next, the chosen base and chosen additive (10 mol%) were added, if used in the chosen amount. Then, the to be tested amount of propionyl chloride (**1a**) was added volumetrically. The milling container was closed and subjected to milling for a defined time at a chosen frequency. If successful, the product was purified by running a dry loaded column chromatography.

General Procedure 2 (GP2) – Optimized Conditions

A stainless-steel milling container equipped with one milling ball (10 mm in Ø) was charged with the chosen allylic amine (**2**, 0.50 mmol, 1.00 equiv.). Next, Hünig's base was added (87 µL, 0.50 mmol, 1.00 equiv.) using an appropriate syringe. Then, the two parts of the container were almost closed leaving a small gap. Using a suitable syringe, the chosen acyl chloride (**1**, 0.75 mmol, 1.50 equiv.) was added through the gap and the jar immediately closed. (***Note***: This is essential for a successful transformation as most likely a volatile ketene intermediate is formed.) Then, the reaction mixture was placed in the mixer mill and milled for 30 minutes at a frequency of 25 Hz. After milling, the container was filled with EtOAc, shaken, and the obtained reaction mixture was transferred to a flask. The procedure was repeated (3–5×) to ensure a complete transfer. Finally, the product **3** was purified by running a dry loaded column chromatography. Therefore, a suitable amount of silica gel was added to the flask, the volatiles removed under reduced pressure to obtain a free-floating powder which was placed on top of the column.

General Procedure 3 (GP3) – Solution/neat

A 10 mL-reaction tube equipped with a magnetic stirring bar was charged with *N*-allylmorpholine (**2a**, 63.6 mg, 0.50 mmol, 1.00 equiv.), which was dissolved in DCM (5 mL), when used. Then, Hünig's base (76.0 µL, 0.50 mmol, 1.00 equiv.) and propionyl chloride (**1a**, 68.0 µL, 0.75 mmol, 1.50 equiv.) were added, the tube closed and stirred at room temperature. After a chosen reaction time, the reaction mixture was transferred to a round bottom flask, and the reaction tube was rinsed with EtOAc (3×5 mL) to complete the transfer. Then, the volatiles were removed, and the product **3** was purified by running a dry loaded column chromatography.

3 Charge-accelerated Aza-Claisen Rearrangement

3.1 Optimization Tables

3.1.1 Base Screening

Table S1. Tested bases in the mechanochemical charge-accelerated aza-Claisen rearrangement. The reaction was performed following **GP1** [stainless steel, t = 1 h, f = 25 Hz, 1 ball (10 mm in Ø), propionyl chloride (1.20 equiv.)].

Entry	Base (1.00 equiv.)	*Y* (3aa) [%]
1	–	3
2	K_2CO_3	–
3	Cs_2CO_3	–
4	Ag_2CO_3	–
5	K_3PO_4	7
6	Al_2O_3 (neutral)	0
7	CaO	4
8	$LiOH{\cdot}H_2O$	4
9	LiOH	9
10	NaOH	>1
11	KOH	6
12	DABCO	–
13	NEt_3	11
14	DIPA	–
15	DBU	–
16	KO^tBu	–
17	Trioctylamine	>1
18	Pyridine	–
19	NaOAc	–
20	*N*,*N*-Di-*iso*-propyl-*iso*-butylamine	–
21	Hünig’s base ($^iPr_2NEt$)	35

3.1.2 Optimization of Hünig's Base Amount

Table S2. Varying the amount of Hünig's base in the mechanochemical charge-accelerated aza-Claisen rearrangement. The reaction was performed following **GP1** [stainless steel, *t* = 1 h, *f* = 25 Hz, 1 ball (10 mm in Ø), propionyl chloride (1.20 equiv.)].

Entry	Equiv.	*Y* (3aa) [%]
1	0.1	24
2	0.5	1
3	0.5	34
4	1.0	6
5	1.0	33
6	1.5	4
7	1.5	18
8	2.0	5
9	2.0	12

3.1.3 Optimization of Ball Milling Conditions

Table S3. Varying the ball milling conditions in the mechanochemical charge-accelerated aza-Claisen rearrangement. The reaction was performed following **GP1** [Hünig's base (1.00 equiv.), propionyl chloride (1.20 equiv.), *f* = 25 Hz].

Entry	Material	Balls (⌀)	*t* [min]	Other changes	*Y* (3aa) [%]
1	ZrO_2–Y	1 (10)	60	No base	22
2	ZrO_2–Y	1 (10)	60	–	40
3	stainless steel	1 (10)	120	–	42
4	stainless steel	1 (10)	60	–	33
5	stainless steel	1 (10)	30	–	58
6	stainless steel	1 (10)	15	–	27
7	stainless steel	1 (10)	5	–	34
8	stainless steel	3 (7)	60	–	24
9	stainless steel	2 (10)	30	1.50 equiv. of propionyl chloride	65
10	stainless steel	3 (7)	30	20 Hz, instead of 25 Hz	55
11	stainless steel	3 (7)	30	–	63
12	stainless steel	3 (7)	30	30 Hz, instead of 25 Hz	59
13	stainless steel	1 (10)	30	1.50 equiv. of propionyl chloride	69

3.1.4 Optimization of Propionyl Chloride Amount

Table S4. Varying the amount of propionyl chloride used in the mechanochemical charge-accelerated aza-Claisen rearrangement. The reaction was performed following **GP1** [stainless steel, *t* = 30 min, *f* = 25 Hz, 1 ball (10 mm in Ø), Hünig's base (1.00 equiv.)].

Entry	Equiv. of 1a	Comments	*Y* (3aa) [%]
1	1.0	–	20
2	1.2	–	63
3	1.5	–	69
4	2.0	–	67
5	1.5	Milling container cooled in liquid nitrogen after the reagents were added, then milling	63
6	1.5	Add substrate and base first, cooled in liquid nitrogen, addition of propionyl chloride, cooled again, milling	51
7	1.5	Keeping the parts of the milling container as close as possible while adding propionyl chloride and close immediately	77
8	1.2	Vessels flushed with argon and reagents added under a "argon shower"	50

3.1.5 Additional Additive Screening

Table S5. Tested additives in the mechanochemical charge-accelerated aza-Claisen rearrangement. The reaction was performed following **GP1** [stainless steel, *t* = 30 min, *f* = 25 Hz, 1 ball (10 mm in Ø), Hünig's base (1.00 equiv.), propionyl chloride (1.50 equiv.)].

Entry	Additive	*Y* (3aa) [%]
1	$Al(OTf)_3$	22
2	$AlCl_3$	37
3	$Fe(OTf)_3$	20
4	$La(OTf)_3$	19
5	$Zn(OTf)_2$	29
6	$PdCl_2$	47
7	$ZnCl_2 \cdot x\ H_2O$	41
8	$ZnCl_2$ (freshly dried)	67
9	$Cu(OTf)_2$	17
10	$CuCl_2$	31
11	CuCl	51
12	CuBr	38
13	CuI	42
14	Covellite	55
15	Bornite	55
16	Montmorillonite K10	25
17	Montmorillonite K30	20
18	Schreiner's thiourea catalyst	–
19	I_2	68
20	PIDA	50
21	NIS	63
22	Iodopentafluorobenzene	57
23	Silica gel	–
24	1,1'-Binaphthyl-2,2'-diyl hydrogenphosphate	53

3.2 Synthesis and Characterization of Additives

***N,N'*–Bis[3,5–bis(trifluoromethyl)phenyl]–thiourea (Schreiner's Thiourea Catalyst)**

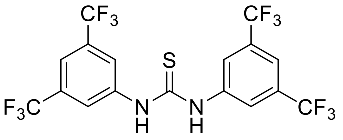

$C_{17}H_8F_{12}N_2S$
m/z: 500.0217
M_W: 500.31 g·mol^{-1}

The title compound was prepared according to a modified literature procedure.[3]

A 4 mL-GC vial was charged with 3,5-bis(trifluoromethyl)aniline (573 mg, 2.50 mmol, 1.00 equiv.) and was dissolved in 0.25 mL of MeOH. A second 4 mL-GC vial was charged with 3,5-bis-(trifluoromethyl)phenyl isothiocyanate (678 mg, 2.50 mmol, 1.00 equiv.) and was dissolved in 0.25 mL MeOH. Both solutions were combined in a 50 mL-round bottom flask, each vial rinsed with additional MeOH (0.25 mL) and the solution was stirred for 1 h at room temperature. Then, the solvent was evaporated to yield the product as colorless solid (1.17 g, 2.33 mmol, 93%) without further purification. The NMR data closely match the ones previously reported in the literature.[4]

m.p.: 159–161 °C.

^{1}H NMR (DMSO-d$_6$, 600 MHz): δ = 10.64 (s, 2H), 8.20 (s, 4H), 7.85 (s, 2H) ppm.

^{13}C{^{1}H} NMR (DMSO-d$_6$, 151 MHz): δ = 180.7, 141.2 (2C), 130.4 (q, $J_{C\text{–}F}$ = 33.1 Hz, 4C), 124.2 (d, $J_{C\text{–}F}$ = 2.5 Hz, 4C), 123.2 (q, $J_{C\text{–}F}$ = 272.6 Hz, 4C), 117.8 (t, $J_{C\text{–}F}$ = 3.8 Hz, 2C) ppm.

^{19}F NMR (DMSO-d$_6$, 564 MHz): δ = –61.6 (s, 12F) ppm.

IR (ATR): 1/λ = 3169 (w), 3047 (w), 2986 (w), 2201 (w), 2046 (w), 1800 (w), 1625 (w), 1552 (m), 1464 (m), 1371 (s), 1324 (m), 1277 (s), 1171 (s), 1123 (s), 1003 (m), 926 (m), 890 (m), 848 (m), 764 (w), 706 (s), 679 (s) cm^{-1}.

CI-MS (100 eV, Methane): *m/z* (%): 501 (100) [*M*+H]$^+$, 500 (9) [*M*]$^+$.

EI-MS (70 eV): *m/z* (%): 501 (28) [*M*+H]$^+$, 500 (81) [*M*]$^+$, 481 (17), 272 (27), 252 (16), 229 (100), 213 (16), 163 (15), 69 (17).

CHN: calcd (%) for $C_{17}H_8F_{12}N_2S$: C 40.81, H 1.61, N 5.60; found: C 40.80, H 2.07, N 5.53.

3.3 Synthesis and Characterization of the Starting Materials

***N*-Allylmorpholine (2a)**

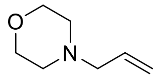

$C_7H_{13}NO$
m/*z*: 127.0997
M_W: 127.19 g·mol^{-1}

A 50 mL-round bottom flask equipped with a magnetic stirring bar was charged with morpholine (5.25 μΛ, ρ = 0.996 g·mL^{-1}, 60.0 mmol, 3.00 equiv.) and cooled to 0 °C using an ice bath. At this temperature, allyl bromide (1.73 mL, ρ = 1.40 g·mL^{-1}, 20.0 mmol, 1.00 equiv.) was added dropwise (**ATTENTION**: The cooling bath is mandatory as the reaction is highly exothermic). After addition, the reaction mixture was kept in the cooling bath and allowed to warm up to room temperature over the course of 21 h. Then, the reaction mixture was suspended between water and distilled Et_2O (each 25 mL), and the organic phase was washed with water (2×25 mL). The organic phase was discarded as it contained impurities. The aqueous phases were combined and extracted with distilled Et_2O (2×100 mL). The organic phases were combined and concentrated under reduced pressure to give the title compound as yellow liquid (559 mg, 4.4 mmol, 22%). The NMR data closely match the ones previously reported in the literature.[5]

^{1}H NMR ($CDCl_3$, 600 MHz): δ = 5.84 (ddtd, *J* = 16.8, 10.2, 6.6, 1.0 Hz, 1H), 5.20 (dq, *J* = 17.1, 1.5 Hz, 1H), 5.16 (ddq, *J* = 10.1, 2.1, 1.1 Hz, 1H), 3.72 (t, *J* = 4.7 Hz, 4H), 2.99 (dq, J = 6.6, 1.2 Hz, 2H), 2.44 (br s, 4H) ppm.

^{13}C{^{1}H} NMR ($CDCl_3$, 151 MHz): δ = 134.7, 118.5, 67.1 (2C), 62.3, 53.7 (2C) ppm.

IR (ATR): 1/λ = 3076 (w), 2957 (s), 2907 (s), 2854 (s), 2799 (s), 2333 (m), 2100 (m), 1992 (w), 1840 (w), 1643 (m), 1451 (m), 1422 (m), 1332 (m), 1291 (s), 1270 (m), 1239 (w), 1206 (w), 1117 (s), 1071 (m), 1034 (w), 1002 (s), 921 (s), 862 (s), 803 (m), 701 (w), 660 (w) cm^{-1}.

CI-MS (100 eV, Methane): *m/z* (%): 255 (76) [2*M*+H]$^+$, 254 (13) [2*M*]$^+$, 128 (55) [*M*+H]$^+$, 127 (10) [*M*]$^+$.

EI-MS (70 eV): *m/z* (%): 128 (3) [*M*+H]$^+$, 127 (5) [*M*]$^+$, 126 (24), 114 (22), 113 (10), 100 (100), 57 (12), 56 (16).

***N*-Cinnamylmorpholine (2b)**

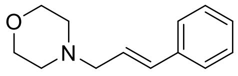

$C_{13}H_{17}NO$
m/*z*: 203.1310
M_W: 203.29 g·mol^{-1}

The title compound was prepared according to a modified literature procedure.[6]

A 25 mL-round bottom flask equipped with a magnetic stirring bar was charged with morpholine (440 µl, ρ = 0.996 g·mL^{-1}, 5.00 mmol, 1.00 equiv.), MeCN (10 mL), and K_2CO_3 (0.76 g, 5.50 mmol, 1.10 equiv.) in the given order. Then, (*E*)-cinnamyl chloride (777 µl, ρ = 1.08 g·mL^{-1}, 5.50 mmol, 1.10 equiv.) was added dropwise and the reaction mixture stirred at room temperature for 21 h. The reaction

mixture was filtered over a plug of cotton and rinsed with MeCN (3×5 mL). The solvent was removed under reduced pressure and the crude product was purified by running a dry loaded column chromatography (SiO_2, pentane:EtOAc 1:0 → 9:1 → MeOH) to obtain the title compound as yellow oil (196 mg, 0.96 mmol, 19%). The NMR data closely match the ones previously reported in the literature.[7]

R$_f$ = 0.75 (MeOH), UV-active.

^{1}H NMR (CDCl$_3$, 600 MHz): δ = 7.39–7.35 (m, 2H), 7.33–7.29 (m, 2H), 7.25–7.21 (m, 1H), 6.54 (d, J = 15.8 Hz, 1H), 6.26 (dt, J = 15.9, 6.8 Hz, 1H), 3.74 (t, J = 4.7 Hz, 4H), 3.16 (dd, J = 6.8 Hz, 2H), 2.51 (br s, 4H) ppm.

^{13}C{^{1}H} NMR (CDCl$_3$, 151 MHz): δ = 136.9, 133.6, 128.7 (2C), 127.7, 126.5 (2C), 126.2, 67.1 (2C), 61.6, 53.8 (2C) ppm.

IR (ATR): $1/\lambda$ = 3872 (w), 3403 (w), 3026 (m), 2955 (m), 2912 (m), 2854 (s), 2804 (s), 2762 (m), 2326 (w), 2092 (w), 2009 (w), 1805 (w), 1679 (w), 1598 (w), 1494 (m), 1450 (s), 1393 (w), 1350 (m), 1325 (m), 1279 (m), 1205 (w), 1116 (s), 1070 (m), 1032 (m), 1003 (s), 969 (s), 904 (m), 866 (s), 788 (m), 740 (s), 692 (s) cm^{-1}.

CI-MS (100 eV, Methane): m/z (%): 204 (100) [M+H]$^+$, 203 (29) [M]$^+$.

EI-MS (70 eV): m/z (%): 204 (33) [M+H]$^+$, 203 (100) [M]$^+$, 202 (28), 144 (13), 118 (10), 117 (57), 115 (33), 112 (79), 91 (16), 56 (17).

***N*-Prenylmorpholine (2c)**

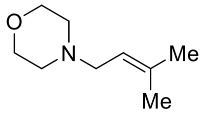

$C_9H_{17}NO$
m/z: 155.1310
M_W: 155.24 g·mol^{-1}

The title compound was prepared according to a modified literature procedure.[6]

A 100 mL-round bottom flask equipped with a magnetic stirring bar was charged with morpholine (1.75 mL, ρ = 0.996 g·mL^{-1}, 20.0 mmol, 1.00 equiv.), MeCN (50 mL), and K_2CO_3 (4.15 g, 30.0 mmol, 1.50 equiv.) in the given order. Then, 3,3-dimethylallyl bromide (2.31 mL, ρ = 1.29 g·mL^{-1}, 20.0 mmol, 1.00 equiv.) was added dropwise and the reaction mixture stirred at room temperature for 21 h. The reaction mixture was filtered over a plug of cotton and rinsed with MeCN (5×5 mL). The solvent was removed under reduced pressure and the crude product was purified by vacuum distillation to yield the title compound at a head temperature of 87 °C as yellow oil (1.88 g, 12.1 mmol, 60%). The NMR data closely match the ones previously reported in the literature.[8]

^{1}H NMR (CDCl$_3$, 600 MHz): δ = 5.24 (tp, J = 7.1, 1.4 Hz, 1H), 3.71 (t, J = 4.7 HZ, 4H), 2.94 (d, J = 7.1 Hz, 2H), 2.44 (br s, 4H), 1.73 (d, J = 1.2 Hz, 3H), 1.65 (d, J = 1.4 Hz, 3H) ppm.

^{13}C{^{1}H} NMR (CDCl$_3$, 151 MHz): δ = 135.9, 120.6, 67.2 (2C), 56.7, 53.8 (2C), 26.1, 18.2 ppm.

IR (ATR): $1/\lambda$ = 3432 (w), 2960 (m), 2918 (m), 2855 (s), 2805 (s), 2684 (w), 2323 (w), 2087 (w), 1988 (w), 1805 (w), 1675 (w), 1449 (s), 1376 (m), 1320 (m), 1289 (m), 1242 (w), 1201 (w), 1116 (s), 1071 (m), 1033 (m), 1002 (s), 907 (m), 865 (s), 784 (m) cm^{-1}.

CI-MS (100 eV, Methane): *m/z* (%): not detectable.

EI-MS (70 eV): *m/z* (%): 156 (2) [*M*+H]$^+$, 155 (28) [*M*]$^+$, 154 (10), 140 (14), 110 (13), 97 (10), 87 (83), 86 (32), 85 (15), 83 (23), 82 (19), 73 (12), 71 (15), 70 (12), 69 (100), 68 (12), 67 (17), 60 (12), 57 (81), 56 (55), 55 (55), 53 (11), 45 (16).

N-Allylpyrrolidine (2d)

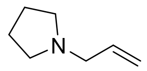

$C_7H_{13}N$
m/z: 111.1048
M_W: 111.19 g·mol^{-1}

The product was prepared following an adjusted literature procedure.[9]

A 100 mL-round bottom flask equipped with a magnetic stirring bar was charged with pyrrolidine (3.34 mL, ρ = 852 mg·mL^{-1}, 40.0 mmol, 1.82 equiv.), which was dissolved in distilled Et_2O (10 mL) and cooled to 0 °C using an ice bath. Then, allyl bromide (1.90 mL, ρ = 1.40 g·mL^{-1}, 22.0 mmol, 1.00 equiv.) was added dropwise at 0 °C and stirred for 30 minutes at this temperature. Next, the ice bath was removed, and the reaction mixture was stirred for 21 h at room temperature. The mixture was filtered over a pad of Celite, which was rinsed with distilled Et_2O. The organic phase was concentrated under reduced pressure and the crude product was purified by vacuum distillation. At a head temperature of 26 °C (oil bath 35 °C) the product was obtained as colorless oil and as a single fraction (152 mg, 1.36 mmol, 7%). The NMR data closely match the ones previously reported in the literature.[9]

^{1}H NMR ($CDCl_3$, 600 MHz): δ = 5.93 (ddtd, *J* = 16.8, 10.2, 6.6, 1.1 Hz, 1H), 5.18 (ddt, *J* = 17.1, 2.8, 1.4 Hz, 1H), 5.08 (ddq, *J* = 10.1, 2.1, 1.1 Hz, 1H), 3.09 (dq, *J* = 6.5, 1.3 Hz, 2H), 2.50 (tdd, *J* = 5.4, 2.6, 1.2 Hz, 4H), 1.78 (dddd, *J* = 6.7, 4.0, 2.9, 1.1 Hz, 4H) ppm.

^{13}C{^{1}H} NMR ($CDCl_3$, 151 MHz): δ = 136.4, 116.7, 59.4, 54.2 (2C), 23.6 (2C) ppm.

IR (ATR): 1/λ = 3435 (w), 3077 (w), 2963 (s), 2910 (s), 2876 (m), 2776 (s), 2324 (w), 2177 (w), 2085 (w), 2024 (w), 1989 (w), 1643 (m), 1460 (m), 1420 (m), 1347 (m), 1317 (m), 1263 (m), 1197 (m), 1143 (s), 1032 (w), 994 (s), 915 (s), 877 (s), 673 (w) cm^{-1}.

CI-MS (100 eV, Methane): *m/z* (%): not detectable.

EI-MS (70 eV): *m/z* (%): 112 (1) [*M*+H]$^+$, 111 (15) [*M*]$^+$, 110 (19), 85 (61), 84 (28), 83 (100), 48 (12), 47 (25).

N-Allylpiperidine (2e)

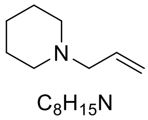

$C_8H_{15}N$
m/z: 125.1204
M_W: 125.22 g·mol^{-1}

The product was prepared following an adjusted literature procedure.[9]

A 100 mL-round bottom flask equipped with a magnetic stirring bar was charged with piperidine (3.95 mL, ρ = 862 mg·mL^{-1}, 40.0 mmol, 1.82 equiv.), which was dissolved in distilled Et_2O (10 mL) and cooled to 0 °C using an ice bath. Then, allyl bromide (1.90 mL, ρ = 1.40 g·mL^{-1}, 22.0 mmol, 1.00 equiv.) was added dropwise at 0 °C and stirred for 30 minutes at this temperature and additional 10 mL distilled Et_2O were added as the reaction mixture was very viscous. Next, the ice bath was removed, and the reaction mixture was stirred for 21 h at room temperature. The mixture was filtered over a pad of Celite, which was rinsed with distilled Et_2O. The organic phase was concentrated (rotary evaporator bath: 35 °C) under reduced pressure and the crude product was purified by vacuum distillation. At a head temperature of 35 °C the product was obtained as colorless oil and as a single fraction (1.38 g, 11.0 mmol, 55%). The NMR data closely match the ones previously reported in the literature.[10]

^{1}H NMR (CDCl$_3$, 600 MHz): δ = 5.88 (ddt, *J* = 16.9, 10.2, 6.6 Hz, 1H), 5.15 (dq, *J* = 17.1, 1.5 Hz, 1H), 5.11 (ddt, *J* = 10.1, 2.1, 1.1 Hz, 1H), 2.95 (dt, *J* = 6.6, 1.3 Hz, 2H), 2.36 (br s, 4H), 1.58 (m, 4H), 1.42 (m, 2H) ppm.

^{13}C{^{1}H} NMR (CDCl$_3$, 151 MHz): δ = 135.8, 117.6, 62.8, 54.6 (2C), 26.1 (2C), 24.5 ppm.

IR (ATR): 1/λ = 3877 (w), 3399 (w), 3077 (w), 3007 (w), 2932 (s), 2855 (s), 2783 (s), 2749 (s), 2323 (w), 2116 (w), 2000 (w), 1838 (w), 1643 (m), 1444 (m), 1385 (m), 1334 (m), 1299 (m), 1274 (m), 1202 (w), 1155 (m), 1111 (s), 1039 (m), 994 (s), 916 (s), 859 (m), 788 (m), 687 (w) cm^{-1}.

CI-MS (100 eV, Methane): *m/z* (%): not detectable.

EI-MS (70 eV): *m/z* (%): 126 (4) [*M*+H]$^+$, 125 (44) [*M*]$^+$, 124 (61), 110 (23), 98 (100), 85 (35), 84 (21), 83 (47), 82 (15), 73 (15), 69 (14), 57 (18), 56 (11), 55 (26).

1-Allyl-4-phenylpiperazine (2f)

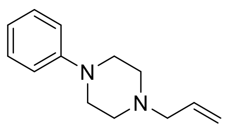

$C_{13}H_{18}N_2$
m/z: 202.1470
M_W: 202.30 g·mol^{-1}

The title compound was prepared according to a modified literature procedure.[6]

A 25 mL-round bottom flask equipped with a magnetic stirring bar was charged with 1-phenylpiperazine (822 mg, 5.00 mmol, 1.00 equiv.), MeCN (10 mL), and K_2CO_3 (760 g, 5.50 mmol, 1.10 equiv.) in the given order. Then, allyl bromide (0.47 mL, ρ = 1.40 g·mL^{-1}, 5.50 mmol, 1.10 equiv.) was added dropwise and the reaction mixture stirred at room temperature for 4 days. The reaction mixture was filtered over a plug of cotton and rinsed with MeCN (3×5 mL). The solvent was removed under reduced pressure and the crude product was purified by running a dry loaded column chromatography (SiO_2, pentane:EtOAc 9:1 → 4:1) to obtain the title compound as yellow oil (677 mg, 3.35 mmol, 67%). The NMR data closely match the ones previously reported in the literature.[11]

R$_f$ = 0.22 (pentane:EtOAc 4:1), UV-active, smears.

^{1}H NMR (CDCl$_3$, 600 MHz): δ = 7.29–7.23 (m, 2H), 6.94 (m, 2H), 6.86 (m, 1H), 5.91 (ddt, *J* = 16.9, 10.2, 6.6 Hz, 1H), 5.23 (dq, *J* = 17.1, 1.5 Hz, 1H), 5.19 (ddt, *J* = 10.1, 2.1, 1.1 Hz, 1H), 3.22 (m, 4H), 3.07 (dt, *J* = 6.6, 1.3 Hz, 2H), 2.62 (m, 4H) ppm.

$^{13}C\{^1H\}$ NMR ($CDCl_3$, 151 MHz): δ = 151.5, 135.0, 129.2 (2C), 119.8, 118.4, 116.2 (2C), 62.0, 53.3 (2C), 49.3 (2C) ppm.

IR (ATR): $1/\lambda$ = 3886 (w), 3416 (w), 3196 (w), 3066 (m), 3030 (w), 2941 (m), 2907 (m), 2882 (m), 2813 (s), 2327 (m), 2082 (m), 1995 (w), 1915 (w), 1821 (m), 1642 (w), 1597 (s), 1498 (s), 1451 (s), 1422 (m), 1382 (m), 1337 (s), 1299 (m), 1230 (s), 1139 (s), 1060 (w), 1003 (s), 922 (s), 878 (w), 814 (m), 756 (s), 690 (s) cm^{-1}.

CI-MS (100 eV, Methane): *m/z* (%): 203 (100) $[M+H]^+$, 202 (77) $[M]^+$.

EI-MS (70 eV): *m/z* (%): 203 (57) $[M+H]^+$, 202 (100) $[M]^+$, 161 (12), 106 (14), 96 (12).

N-Benzyl-*N*-methylprop-2-en-1-amine (2g)

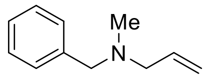

$C_{11}H_{15}N$
m/z: 161.1204
M_W: 161.25 g·mol^{-1}

The product was prepared following an adjusted literature procedure.[9]

A 100 mL-round bottom flask equipped with a magnetic stirring bar was charged with *N*-benzylmethylamine (4.85 g, 40.0 mmol, 1.82 equiv.), which was dissolved in distilled Et_2O (10 mL) and cooled to 0 °C using an ice bath. Then, allyl bromide (1.90 mL, ρ = 1.40 g·mL^{-1}, 22.0 mmol, 1.00 equiv.) was added dropwise at 0 °C and stirred for 30 minutes at this temperature. Next, the ice bath was removed, and the reaction mixture was stirred for 21 h at room temperature. The mixture was filtered over a pad of Celite, which was rinsed with distilled Et_2O. The organic phase was concentrated (rotary evaporator bath: 35 °C) under reduced pressure and the crude product was purified by vacuum distillation. At a head temperature of 85 °C the product was obtained as colorless oil (2.31 g, 14.3 mmol, 72%). The NMR data closely match the ones previously reported in the literature.[5]

^1H NMR ($CDCl_3$, 600 MHz): δ = 7.34–7.29 (m, 4H), 7.27–7.22 (m, 1H), 5.92 (ddt, *J* = 16.8, 10.2, 6.5 Hz, 1H), 5.20 (dq, *J* = 17.2, 1.5 Hz, 1H), 5.15 (ddt, *J* = 10.2, 2.2, 1.2 Hz, 1H), 3.50 (s, 2H), 3.03 (dt, *J* = 6.5, 1.3 Hz, 2H), 2.19 (s, 3H) ppm.

$^{13}C\{^1H\}$ NMR ($CDCl_3$, 151 MHz): δ = 139.2, 136.1, 129.2 (2C), 128.4 (2C), 127.1, 117.6, 61.8, 60.7, 42.2 ppm.

IR (ATR): $1/\lambda$ = 3876 (w), 3418 (w), 3068 (m), 3028 (m), 2978 (m), 2920 (m), 2877 (m), 2834 (m), 2782 (s), 2323 (w), 2095 (w), 1999 (w), 1810 (m), 1643 (m), 1601 (w), 1493 (m), 1451 (s), 1365 (m), 1274 (m), 1251 (m), 1200 (m), 1132 (m), 1075 (m), 1026 (s), 993 (s), 917 (s), 859 (s), 821 (m), 737 (s), 697 (s) cm^{-1}.

CI-MS (100 eV, Methane): *m/z* (%): 162 (100) $[M+H]^+$, 161 (32) $[M]^+$.

EI-MS (70 eV): *m/z* (%): 162 (100) $[M+H]^+$, 161 (48) $[M]^+$, 160 (44), 134 (15).

_N,N_-Dipropylprop-2-en-1-amine (2h)

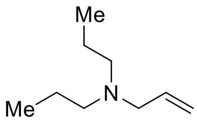

$C_9H_{19}N$
m/*z*: 141.1517
M_W: 141.26 g·mol^{-1}

The product was prepared following an adjusted literature procedure.[9]

A 100 mL-round bottom flask equipped with a magnetic stirring bar was charged with dipropylamine (4.05 mL, 40.0 mmol, 1.82 equiv.), which was dissolved in distilled Et_2O (10 mL) and cooled to 0 °C using an ice bath. Then, allyl bromide (1.90 mL, ρ = 1.40 g·mL^{-1}, 22.0 mmol, 1.00 equiv.) was added dropwise at 0 °C and stirred for 30 minutes at this temperature. Next, the ice bath was removed, and the reaction mixture was stirred for 21 h at room temperature. The mixture was filtered over a pad of Celite, which was rinsed with distilled Et_2O. The organic phase was concentrated (rotary evaporator bath: 35 °C) under reduced pressure and the crude product was purified by vacuum distillation. At a head temperature of 35 °C the product was obtained as colorless oil and as a single fraction (1.44 g, 10.2 mmol, 51%). The NMR data closely match the ones previously reported in the literature.[12]

^{1}H NMR ($CDCl_3$, 600 MHz): δ = 5.87 (ddt, *J* = 16.8, 10.0, 6.5 Hz, 1H), 5.15 (dq, *J* = 17.1, 1.6 Hz, 1H), 5.09 (ddt, *J* = 10.1, 2.2, 1.2 Hz, 1H), 3.08 (m, 2H), 2.37 (m, 4H), 1.46 (m, 4H), 0.87 (t, *J* = 7.4 Hz, 6H) ppm.

$^{13}C\{^1H\}$ NMR ($CDCl_3$, 151 MHz): δ = 136.5, 116.9, 57.5, 56.0 (2C), 20.3 (2C), 12.1 (2C) ppm.

IR (ATR): 1/λ = 3446 (w), 3077 (m), 2960 (s), 2874 (s), 2798 (s), 1985 (w), 1836 (w), 1688 (w), 1642 (w), 1461 (s), 1419 (m), 1381 (m), 1272 (m), 1188 (m), 1168 (m), 1075 (s), 1027 (m), 995 (m), 958 (m), 916 (s), 841 (w), 749 (w), 632 (w), 509 (w) cm^{-1}.

CI-MS (100 eV, Methane): *m*/*z* (%): 142 (9) [*M*+H]$^+$, 141 (1) [*M*]$^+$.

EI-MS (70 eV): *m*/*z* (%): 142 (1) [*M*+H]$^+$, 141 (6) [*M*]$^+$, 119 (11), 112 (33).

_N_-(2-Methylallyl)morpholine (2i)

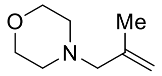

$C_8H_{15}NO$
m/*z*: 141.1154
M_W: 141.21 g·mol^{-1}

The title compound was prepared according to a modified literature procedure.[6]

A 100 mL-round bottom flask equipped with a magnetic stirring bar was charged with morpholine (1.75 mL, ρ = 0.996 g·mL^{-1}, 20.0 mmol, 1.00 equiv.), MeCN (50 mL), and K_2CO_3 (4.15 g, 30.0 mmol, 1.50 equiv.) in the given order. Then, 3-chloro-2-methylpropene (1.97 mL, ρ = 0.92 g·mL^{-1}, 20.0 mmol, 1.00 equiv.) was added dropwise and the reaction mixture stirred at room temperature for 21 h. The reaction mixture was filtered over a plug of cotton and rinsed with MeCN (4×5 mL). The solvent was removed under reduced pressure and the crude product was purified by vacuum distillation (2×) to yield the title compound at a head temperature of 62 °C as yellow oil (1.71 g, 12.1 mmol, 60%).

^{1}H NMR (CDCl$_3$, 600 MHz): δ = 4.89–4.83 (m, 2H), 3.70 (t, *J* = 4.7 Hz, 4H), 2.86 (s, 2H), 2.37 (br s, 4H), 1.74 (s, 3H) ppm.

^{13}C{^{1}H} NMR (CDCl$_3$, 151 MHz): δ = 142.4, 113.4, 67.3 (2C), 65.9, 53.8 (2C), 21.0 ppm.

IR (ATR): 1/λ = 3075 (w), 2959 (m), 2854 (s), 2803 (s), 2329 (w), 2208 (w), 2118 (w), 1984 (w), 1935 (w), 1801 (w), 1651 (w), 1451 (m), 1371 (w), 1345 (w), 1292 (m), 1267 (m), 1204 (w), 1117 (s), 1072 (m), 1010 (s), 974 (w), 899 (s), 866 (s), 807 (m), 708 (w) cm^{-1}.

CI-MS (100 eV, Methane): *m/z* (%): not detectable.

EI-MS (70 eV): *m/z* (%): 142 (2) [*M*+H]$^+$, 141 (17) [*M*]$^+$, 125 (13), 111 (17), 110 (11), 109 (12), 100 (30), 97 (26), 96 (18), 95 (17), 87 (13), 86 (12), 85 (71), 84 (13), 83 (100), 82 (19), 81 (16), 73 (18), 71 (23), 70 (14), 69 (30), 68 (10), 67 (12), 57 (46), 56 (33), 55 (55), 48 (13), 47 (27), 45 (13).

_N_-Allyl-1,2,3,4-tetrahydroquinoline (2j)

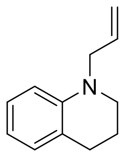

$C_{12}H_{15}N$
m/z: 173.1204
M_W: 173.26 g·mol^{-1}

The title compound was prepared according to a modified literature procedure.[6]

A 25 mL-round bottom flask equipped with a magnetic stirring bar was charged with 1,2,3,4-tetrahydroquinoline (667 mg, 5.00 mmol, 1.00 equiv.), MeCN (10 mL), and K_2CO_3 (760 g, 5.50 mmol, 1.10 equiv.) in the given order. Then, allyl bromide (0.47 mL, ρ = 1.40 g·mL^{-1}, 5.50 mmol, 1.10 equiv.) was added dropwise and the reaction mixture stirred at room temperature for 21 h. The reaction mixture was filtered over a plug of cotton and rinsed with MeCN (3×5 mL). The solvent was removed under reduced pressure and the crude product was purified by running a dry loaded column chromatography (SiO_2, pentane:EtOAc 1:0 → 9:1) to obtain the title compound as dark orange oil (627 mg, 3.62 mmol, 72%). The NMR data closely match the ones previously reported in the literature.[13]

R$_f$ = 0.15 (pentane), UV-active.

^{1}H NMR (CDCl$_3$, 600 MHz): δ = 7.03 (m, 1H), 6.95 (m, 1H), 6.57 (m, 2H), 5.85 (ddtd, *J* = 17.4, 10.1, 5.0, 1.2 Hz, 1H), 5.20 (dp, *J* = 17.2, 1.7 Hz, 1H), 5.15 (dp, *J* = 10.3, 1.6 Hz, 1H), 3.87 (dq, *J* = 4.8, 1.6 Hz, 2H), 3.28 (m, 2H), 2.77 (t, *J* = 6.4 Hz, 2H), 1.97 (dtdd, *J* = 7.1, 5.8, 5.0, 1.2 Hz, 2H) ppm.

^{13}C{^{1}H} NMR (CDCl$_3$, 151 MHz): δ = 145.5, 133.7, 129.1, 127.2, 122.5, 116.0, 115.9, 111.1, 54.0, 49.3, 28.3, 22.5 ppm.

IR (ATR): 1/λ = 3466 (w), 3068 (m), 3017 (m), 2931 (s), 2841 (s), 2634 (w), 2214 (w), 1879 (w), 1844 (w), 1752 (w), 1639 (m), 1602 (s), 1502 (s), 1454 (s), 1401 (m), 1340 (s), 1303 (s), 1244 (s), 1191 (s), 1113 (m), 1057 (m), 991 (m), 965 (m), 918 (s), 871 (w), 834 (w), 800 (w), 744 (s), 717 (m), 655 (w), 554 (w) cm^{-1}.

CI-MS (100 eV, Methane): *m/z* (%): 174 (51) [*M*+H]$^+$, 173 (100) [*M*]$^+$.

EI-MS (70 eV): *m/z* (%): 174 (15) [*M*+H]$^+$, 173 (100) [*M*]$^+$, 172 (13), 146 (46), 144 (10), 132 (10), 131 (10), 130 (21), 118 (11), 117 (21), 91 (13).

***N*-Allyl-*N*-methylaniline (2k)**

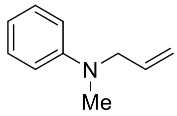

$C_{10}H_{13}N$
m/z: 147.1048
M_W: 147.22 g·mol^{-1}

The title compound was prepared according to a modified literature procedure.[6]

A 25 mL-round bottom flask equipped with a magnetic stirring bar was charged with freshly distilled *N*-methylaniline (537 mg, 5.00 mmol, 1.00 equiv.), MeCN (10 mL), and K_2CO_3 (760 g, 5.50 mmol, 1.10 equiv.) in the given order. Then, allyl bromide (0.47 mL, ρ = 1.40 g·mL^{-1}, 5.50 mmol, 1.10 equiv.) was added dropwise and the reaction mixture stirred at room temperature for 21 h. The reaction mixture was filtered over a plug of cotton and rinsed with MeCN (3×5 mL). The solvent was removed under reduced pressure and the crude product was purified by running a dry loaded column chromatography (SiO_2, pentane:EtOAc 99:1 → 98:2 → 19:1) to obtain the title compound as yellow oil (437 mg, 2.97 mmol, 59%). The NMR data closely match the ones previously reported in the literature.[14]

R$_f$ = 0.13 (pentane), UV-active.

^{1}H NMR (CDCl$_3$, 600 MHz): δ = 7.23 (m, 2H), 6.75–6.68 (m, 3H), 5.85 (m, 1H), 5.20–5.12 (m, 2H), 3.92 (m, 2H), 2.94 (s, 3H) ppm.

^{13}C{^{1}H} NMR (CDCl$_3$, 151 MHz): δ = 149.6, 134.0, 129.2 (2C), 116.5, 116.3, 112.6 (2C), 55.4, 38.1 ppm.

IR (ATR): 1/λ = 3063 (w), 3029 (w), 2979 (w), 2815 (w), 2162 (w), 2032 (w), 1916 (w), 1743 (w), 1640 (w), 1598 (s), 1503 (s), 1450 (w), 1424 (w), 1362 (s), 1244 (s), 1205 (s), 1083 (s), 991 (s), 918 (s), 863 (w), 746 (s), 689 (s) cm^{-1}.

CI-MS (100 eV, Methane): *m/z* (%): 148 (7) [*M*+H]$^+$, 147 (5) [*M*]$^+$.

EI-MS (70 eV): *m/z* (%): 148 (1) [*M*+H]$^+$, 147 (10) [*M*]$^+$, 146 (68), 144 (22), 132 (12), 120 (41), 118 (18), 107 (12), 106 (23), 104 (17), 93 (10), 77 (25).

***N,N*-Dibenzylprop-2-en-1-amine (2l)**

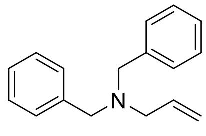

$C_{17}H_{19}N$
m/z: 237.1517
M_W: 237.35 g·mol^{-1}

The product was prepared following an adjusted literature procedure.[9]

A 100 mL-round bottom flask equipped with a magnetic stirring bar was charged with dibenzylamine (7.90 g, 40.0 mmol, 1.82 equiv.), which was dissolved in distilled Et_2O (10 mL) and cooled to 0 °C using

an ice bath. Then, allyl bromide (1.90 mL, ρ = 1.40 g·mL^{-1}, 22.0 mmol, 1.00 equiv.) was added dropwise at 0 °C and stirred for 30 minutes at this temperature. Next, the ice bath was removed, and the reaction mixture was stirred for 21 h at room temperature. The mixture was filtered over a pad of Celite, which was rinsed with distilled Et_2O. The organic phase was concentrated (rotary evaporator bath: 35 °C) under reduced pressure and the crude product was purified by vacuum distillation. At a head temperature range of 140–150 °C the product was obtained together with impurities. Therefore, the crude product was purified by running a dry loaded column chromatography (SiO_2, pentane:EtOAc 99:1). The title compound was obtained as colorless oil (1.57 g, 6.62 mmol, 33%). The NMR data closely match the ones previously reported in the literature.[5]

R$_f$ = 0.57 (pentane:EtOAc 98:2), UV-active.

^{1}H NMR (CDCl$_3$, 600 MHz): δ = 7.40–7.36 (m, 4H), 7.34–7.29 (m, 4H), 7.26–7.20 (m, 2H), 5.92 (m, 1H), 5.92 (m, 1H), 5.22 (m, 1H), 5.15 (m, 1H), 3.58 (s, 4H), 3.07 (dt, *J* = 6.2, 1.4 Hz, 2H) ppm.

^{13}C{^{1}H} NMR (CDCl$_3$, 151 MHz): δ = 139.8 (2C), 136.1, 128.9 (4C), 128.3 (4C), 126.9 (2C), 117.5, 57.9 (2C), 56.5 ppm.

IR (ATR): 1/λ = 3898 (w), 3468 (w), 3064 (s), 3028 (s), 2978 (m), 2922 (s), 2882 (m), 2795 (s), 2712 (m), 2314 (w), 1950 (w), 1872 (w), 1810 (w), 1742 (w), 1642 (w), 1601 (m), 1493 (s), 1449 (s), 1368 (s), 1325 (m), 1251 (m), 1202 (w), 1119 (s), 1071 (m), 1028 (m), 983 (s), 1071 (m), 1028 (m), 983 (s), 918 (s), 867 (w), 820 (w), 741 (s), 698 (s), 642 (w), 613 (w), 561 (w), 507 (w) cm^{-1}.

CI-MS (100 eV, Methane): *m/z* (%): 238 (100) [*M*+H]$^+$, 237 (24) [*M*]$^+$.

EI-MS (70 eV): *m/z* (%): 238 (13) [*M*+H]$^+$, 237 (79) [*M*]$^+$, 236 (13), 235 (45), 211 (18), 210 (18), 209 (19), 181 (10), 161 (27), 160 (20), 259 (26), 146 (51), 145 (15), 144 (10), 91 (100), 89 (49), 65 (28).

1-Allyl-2,2,6,6-tetramethylpiperidine (2m)

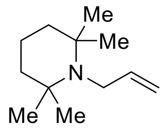

$C_{12}H_{23}N$
m/z: 181.1830
M_W: 181.32 g·mol^{-1}

The title compound was prepared according to a modified literature procedure.[6]

A 25 mL-round bottom flask equipped with a magnetic stirring bar was charged with 2,2,6,6-tetramethylpiperidine (712 mg, 5.04 mmol, 1.00 equiv.), MeCN (10 mL), and K_2CO_3 (766 g, 5.54 mmol, 1.10 equiv.) in the given order. Then, allyl bromide (0.47 mL, ρ = 1.40 g·mL^{-1}, 5.50 mmol, 1.10 equiv.) was added dropwise and the reaction mixture stirred at room temperature for 4 days. The reaction mixture was filtered over a plug of cotton and rinsed with MeCN (3×5 mL). The solvent was removed under reduced pressure and the crude product was purified by running a dry loaded column chromatography (SiO_2, pentane:EtOAc 9:1) to obtain the title compound as yellow oil (349 mg, 1.92 mmol, 38%). The NMR data closely match the ones previously reported in the literature.[15]

R$_f$ = 0.44 (pentane:EtOAc 9:1), stains with $KMnO_4$, smears.

^{1}H NMR (CDCl$_3$, 600 MHz): δ = 5.86 (ddt, *J* = 17.2, 10.3, 5.2 Hz, 1H), 5.13 (dq, *J* = 17.2, 2.0 Hz, 1H), 4.91 (dq, *J* = 10.2, 1.9 Hz, 1H), 3.12 (dt, *J* = 5.2, 1.9 Hz, 2H), 1.56–1.51 (m, 2H), 1.44–1.41 (m, 4H), 1.01 (s, 12H) ppm.

^{13}C{^{1}H} NMR (CDCl$_3$, 151 MHz): δ = 143.7, 112.6, 54.8 (2C), 47.0, 41.4 (2C), 27.6 (4C), 18.0 ppm.

IR (ATR): 1/λ = 3455 (w), 3076 (w), 2966 (s), 2928 (s), 2684 (w), 2161 (w), 2034 (w), 1992 (w), 1818 (w), 1742 (w), 1640 (w), 1463 (s), 1374 (s), 1295 (m), 1259 (s), 1235 (s), 1174 (s), 1128 (s), 1055 (s), 987 (s), 909 (s), 796 (m), 699 (m) cm^{-1}.

CI-MS (100 eV, Methane): *m/z* (%): 181 (31) [*M*]$^+$.

EI-MS (70 eV): *m/z* (%): 182 (28) [*M*+H]$^+$, 181 (53) [*M*]$^+$, 167 (12), 166 (100).

N-Crotylmorpholine (2n)

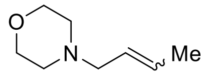

$C_8H_{15}NO$
m/z: 141.1154
M_W: 141.21 g·mol^{-1}

A 250 mL-round bottom flask equipped with a magnetic stirring bar was charged with morpholine (4.37 mL, ρ = 0.996 g·mL^{-1}, 50.0 mmol, 1.00 equiv.), K_2CO_3 (7.60 g, 55.0 mmol, 1.10 equiv.), and distilled Et_2O (200 mL) in the given order. Then, the reaction mixture was cooled to 0 °C using an ice bath and crotyl chloride (predominantly *trans*, 5.64 mL, ρ = 0.929 g·mL^{-1}, 55.0 mmol, 1.10 equiv.) was added dropwise over 15 minutes at 0 °C. After complete addition, the cooling bath was removed, and the reaction mixture stirred for 21 h at room temperature. Then, the resulting mixture was filtered over a pad of Celite, which was rinsed again with distilled Et_2O (20 mL). The organic phase was washed with 1 M $NaOH_{(aq.)}$ and concentrated under reduced pressure. The obtained residue was subjected to a vacuum distillation. The crude product was obtained as a single fraction at a head temperature of 93 °C (oil bath 145 °C). It was further purified by running a dry loaded column chromatography (SiO_2, pentane:EtOAc 1:1 → 0:1) to yield the title compound as yellow liquid (567 mg, 4.01 mmol, 8%) as *cis*:*trans*-mixture (25:75 determined by ^{1}H NMR). The NMR data closely match the ones previously reported in the literature.[16]

R_f = 0.16 (EtOAc), stains with $KMnO_4$ without heating.

^{1}H NMR (CDCl$_3$, 600 MHz): δ (*cis*+*trans*) = 5.69–5.57 (m, 1H), 5.52–5.44 (m, 1H), 3.75–3.67 (m, 4H), 3.04–2.87 {m [*cis*: 3.01 (m)]; *trans*: 2.91 (m), 2H}, 2.51–2.34 (m, 4H}, 1.71–1.61 {m [*cis*: 1.65 (dd, *J* = 6.9, 1.7 Hz); *trans*: 1.69 (dd, *J* = 6.4, 1.3 Hz)], 3H} ppm.

^{13}C{^{1}H} NMR (CDCl$_3$, 151 MHz): δ (*cis*) = 127.9, 126.4, 67.2 (2C), 55.3, 53.8 (2C), 13.3 ppm; δ (*trans*) = 129.7, 127.2, 67.1 (2C), 61.5, 53.7 (2C), 18.0 ppm.

IR (ATR): 1/λ = 3858 (w), 3423 (w), 3018 (w), 2957 (s), 2919 (s), 2855 (s), 2803 (s), 2325 (w), 2101 (w), 1992 (w), 1791 (m), 1671 (m), 1450 (s), 1347 (m), 1283 (m), 1205 (w), 1117 (s), 1071 (m), 1033 (w), 1000 (s), 970 (s), 924 (m), 864 (s), 807 (w), 755 (w), 703 (w) cm^{-1}.

CI-MS (100 eV, Methane): *m/z* (%): not detectable.

EI-MS (70 eV): *m/z* (%): 142 (3) [*M*+H]$^+$, 141 (39) [*M*]$^+$, 140 (14), 127 (12), 126 (17), 111 (17), 105 (32), 100 (14), 97 (34), 96 (21), 95 (15), 87 (23), 85 (30), 83 (38), 81 (14), 71 (38), 70 (13), 69 (46), 67 (10), 57 (95), 56 (32), 55 (100).

3.4 Synthesis and Characterization of the Products

2-Methyl-1-morpholinopent-4-en-1-one (3aa)

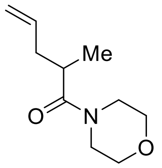

$C_{10}H_{17}NO_2$
m/z: 183.1259
M_W: 183.25 g·mol^{-1}

The title compound was prepared following the **GP2** using *N*-allylmorpholine (**2a**, 63.6 mg, 0.50 mmol, 1.00 equiv.), propionyl chloride (**1a**, 68.0 µL, 0.75 mmol, 1.50 equiv.), and Hünig's base (76.0 µL, 0.50 mmol, 1.00 equiv.). After a dry loaded column chromatography (SiO_2, EtOAc) the product **3aa** was obtained as yellow viscous oil (71 mg, 0.39 mmol, 77%). Repeating the reaction twice yielded 80% and 76%, respectively. Performing the same reaction on a 1 mmol scale yielded the product in 84% (153 mg, 0.84 mmol). The NMR data reported closely match the ones previously reported in the literature.[17]

R_f = 0.51 (EtOAc), stains with $KMnO_4$.

^{1}H NMR ($CDCl_3$, 600 MHz): δ = 5.71 (ddt, *J* = 17.1, 10.2, 7.0 Hz, 1H), 5.02 (dq, *J* = 17.2, 1.7 Hz, 1H), 4.98 (ddt, *J* = 10.2, 2.1, 1.1 Hz, 1H), 3.65–3.42 (m, 8H), 2.68 (sextet, *J* = 6.9 Hz, 1H), 2.38 (dtt, *J* = 13.6, 6.7, 1.4 Hz, 1H), 2.09 (m, 1H), 1.08 (d, *J* = 6.9 Hz, 3H) ppm.

^{13}C{^{1}H} NMR ($CDCl_3$, 151 MHz): δ = 174.5, 136.0, 116.7, 67.1, 66.9, 46.1, 42.1, 38.1, 35.1, 17.3 ppm.

IR (ATR): $1/\lambda$ = 3489 (w), 3272 (w), 3075 (w), 2970 (m), 2920 (m), 2856 (m), 2331 (w), 2078 (w), 1734 (w), 1638 (s), 1432 (s), 1364 (m), 1300 (w), 1268 (m), 1222 (s), 1154 (w), 1114 (s), 1068 (m), 1028 (s), 913 (s), 844 (m), 724 (w) cm^{-1}.

CI-MS (100 eV, Methane): *m/z* (%): 367 (14) [2*M*+H]$^+$, 184 (100) [*M*+H]$^+$, 183 (10) [*M*]$^+$.

EI-MS (70 eV): *m/z* (%): 367 (5) [2*M*+H]$^+$, 184 (100) [*M*+H]$^+$, 183 (37) [*M*]$^+$, 114 (11), 86 (11).

2-Methyl-1-morpholino-3-phenylpent-4-en-1-one (3ab)

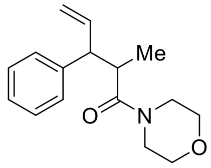

$C_{16}H_{21}NO_2$
m/z: 259.1572
M_W: 259.35 g·mol^{-1}

The title compound was prepared following the **GP2** using *N*-cinnamylmorpholine (**2b**, 102 mg, 0.5 mmol, 1.00 equiv.), propionyl chloride (**1a**, 66.0 µL, 0.75 mmol, 1.50 equiv.), and Hünig's base (87.0 µL, 0.50 mmol, 1.00 equiv.). After a dry loaded column chromatography (SiO_2, pentane:EtOAc 1:1) the product **3ab** was obtained as yellow, viscous oil (51.3 mg, 0.20 mmol, 40%) and as a mixture of diastereomers (4:1 determined by ^{1}H NMR spectroscopy). The NMR data (for the major diastereomer) closely match the ones previously reported in the literature.[18]

R_f = 0.33 (pentane:EtOAc 1:1), UV-active, stains with $KMnO_4$.

^{1}H NMR ($CDCl_3$, 600 MHz): δ (mixture of diastereomers 4:1)= 7.34–7.15 (m, 5H), 6.04–5.94 {m, 1H; [6.01 (ddd, J = 17.1, 10.4, 7.8 Hz, 1H, major diastereomer)] + [6.01–5.95 (m, 1H, minor diastereomer)]}, 5.19–4.96 {m, 2H; [5.19–5.12 (m, 2H, minor diastereomer)] + [5.02 (dt, *J* = 10.4, 1.3 Hz, 1H) & 4.99 (dt, *J* = 17.1, 1.4 Hz, 1H), major diastereomer]}, 3.70–3.47 (m, 8H), 3.46–3.10 (m, 1H), 3.09–2.97 {m, 1H; [3.06 (dq, *J* = 9.9, 6.8 Hz, 1H, major diastereomer)] + [3.00 (dq, *J* = 10.3, 6.6 Hz, 1H, minor diastereomer)]}, 1.20–0.88 {m, 3H; [1.19 (d, *J* = 6.7 Hz, 3H, minor diastereomer)] + [0.92 (d, *J* = 6.7 Hz, 3H, major diastereomer)]} ppm.

^{13}C{^{1}H} NMR ($CDCl_3$, 600 MHz): δ (major diastereomer) = 174.1, 141.8, 139.8, 127.7 (2C), 128.4 (2C), 126.8, 115.7, 67.1, 66.8, 53.4, 46.3, 42.2, 39.8, 16.8 ppm; δ (minor diastereomer) = 174.0, 143.1, 138.8, 128.6 (2C), 127.8 (2C), 126.7, 117.2, 66.4, 54.2, 46.1, 42.0, 40.2, 16.9 ppm. (***Note***: For the minor diastereomer only 15 C were detected. Most likely the missing signal is overlayed by the signals of the major diastereomer.)

IR (ATR): 1/λ = 3481 (w), 3063 (w), 2972 (m), 2922 (m), 2858 (m), 2329 (w), 2076 (w), 1885 (w), 1757 (w), 1626 (s), 1436 (s), 1363 (w), 1300 (w), 1241 (s), 1150 (w), 1113 (s), 1070 (m), 1028 (s), 912 (m), 845 (m), 766 (m), 736 (m), 701 (s) cm^{-1}.

CI-MS (100 eV, Methane): *m/z* (%): 260 (100) [*M*+H]$^+$, 259 (5) [*M*]$^+$.

EI-MS (70 eV): *m/z* (%): 519 (9) [2*M*+H]$^+$, 260 (100) [*M*+H]$^+$, 259 (46) [*M*]$^+$, 258 (41), 245 (14), 244 (84), 118 (10), 117 (34), 115 (15), 114 (10).

2,3,3-Trimethyl-1-morpholinopent-4-en-1-one (3ac)

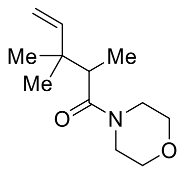

$C_{12}H_{21}NO_2$
m/z: 211.1572
M_W: 211.31 g·mol^{-1}

The title compound was prepared following the **GP2** using *N*-prenylmorpholine (**2c**, 77.6 mg, 0.50 mmol, 1.00 equiv.), propionyl chloride (**1a**, 66.0 µL, 0.75 mmol, 1.50 equiv.), and Hünig's base (87.0 µL, 0.50 mmol, 1.00 equiv.). After a dry loaded column chromatography (SiO_2, pentane:EtOAc 1:1) the product **3ac** was obtained as yellow viscous oil (46.3 mg, 0.22 mmol, 44%).

R_f = 0.26 (pentane:EtOAc 1:1), stains with $KMnO_4$.

^{1}H NMR ($CDCl_3$, 600 MHz): δ = 5.90 (dd, *J* = 17.4, 10.9 Hz, 1H), 4.97 (m, 1H), 4.95 (dd, *J* = 10.9, 1.3 Hz, 1H), 3.68–3.48 (m, 8H), 2.62 (q, *J* = 6.9 Hz, 1H), 1.07 (s, 3H), 1.06 (s, 3H), 1.05 (s, 3H) ppm.

^{13}C{^{1}H} NMR ($CDCl_3$, 151 MHz): δ = 174.2, 146.6, 111.7, 67.2, 66.9, 47.0, 42.5, 42.1, 39.5, 24.8, 24.2, 13.7 ppm.

IR (ATR): 1/λ = 3491 (w), 3263 (w), 3081 (w), 2966 (m), 2858 (m), 2325 (w), 2161 (w), 1934 (w), 1731 (w), 1635 (s), 1427 (s), 1363 (m), 1300 (w), 1265 (m), 1234 (s), 1115 (s), 1073 (m), 1025 (s), 946 (w), 911 (s), 843 (m), 779 (w), 684 (w) cm^{-1}.

CI-MS (100 eV, Methane): *m/z* (%): 212 (100) [*M*+H]$^+$, 211 (6) [*M*]$^+$.

EI-MS (70 eV): *m/z* (%): 212 (100) [*M*+H]$^+$, 211 (19) [*M*]$^+$, 196 (19), 143 (17), 142 (30), 114 (12), 87 (10), 69 (13), 55 (11).

HRMS (ESI): *m/z* calcd for $C_{12}H_{21}O_2N+H^+$ [*M*+H]$^+$: 212.1645; found: 212.1641.

2-Methyl-1-(pyrrolidin-1-yl)pent-4-en-1-one (3ad)

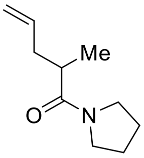

$C_{10}H_{17}NO$
m/z: 167.1310
M_W: 167.25 g·mol^{-1}

The title compound was prepared following the **GP2** using *N*-allylpyrrolidine (**2d**, 55.6 mg, 0.51 mmol, 1.00 equiv.), propionyl chloride (**1a**, 66.0 µL, 0.76 mmol, 1.50 equiv.), and Hünig's base (88.0 µL, 0.51 mmol, 1.00 equiv.). After a dry loaded column chromatography (SiO_2, EtOAc) the product **3ad** was obtained as yellow viscous oil (56.5 mg, 0.34 mmol, 68%). However, ^{1}H NMR analysis showed the presence of propionic acid. Therefore, the crude product was dissolved in distilled Et_2O (20 mL) and washed with 1 M $NaOH_{(aq.)}$ (3×10 mL) to yield the pure product as yellow viscous oil (31.2 mg, 0.19 mmol, 37%) after the solvent was removed under reduced pressure. The NMR data closely match the ones previously reported in the literature.[19]

R$_f$ = 0.29 (EtOAc), stains with $KMnO_4$.

^{1}H NMR ($CDCl_3$, 600 MHz): δ = 5.75 (dddd, *J* = 16.8, 10.1, 7.6, 6.5 Hz, 1H), 5.04 (dq, *J* = 17.0, 1.6 Hz, 1H), 4.98 (ddt, *J* = 10.1, 2.1, 1.1 Hz, 1H), 3.52–3.33 (m, 4H), 2.57 (sextet, *J* = 6.9 Hz, 1H), 2.41 (dddt, *J* = 13.9, 7.6, 6.5, 1.4 Hz, 1H), 2.17–2.05 (m, 1H), 1.96–1.89 (m, 2H), 1.88–1.77 (m, 2H), 1.10 (d, *J* = 6.8 Hz, 3H) ppm.

^{13}C{^{1}H} NMR ($CDCl_3$, 151 MHz): δ = 174.6, 136.5, 116.5, 46.5, 45.8, 38.2, 38.1, 26.3, 24.4, 17.0 ppm.

IR (ATR): 1/λ = 3477 (w), 3074 (w), 2970 (m), 2874 (m), 2328 (w), 2091 (w), 2004 (w), 1890 (w), 1756 (w), 1633 (s), 1430 (s), 1373 (w), 1334 (m), 1256 (w), 1225 (w), 1188 (w), 1114 (w), 1034 (w), 994 (w), 912 (m), 866 (w), 804 (w), 746 (w), 692 (w), 668 (w) cm^{-1}.

CI-MS (100 eV, Methane): *m/z* (%): 168 (100) [*M*+H]$^+$, 167 (6) [*M*]$^+$.

EI-MS (70 eV): *m/z* (%): 168 (85) [*M*+H]$^+$, 167 (100) [*M*]$^+$, 166 (14), 152 (65), 138 (10), 126 (29), 125 (37), 124 (23), 98 (70), 97 (37), 72 (18), 71 (15), 70 (49), 69 (31), 68 (13), 56 (28), 55 (51), 53 (10).

2-Methyl-1-(piperidin-1-yl)pent-4-en-1-one (3ae)

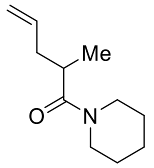

$C_{11}H_{19}NO$
m/z: 181.1467
M_W: 181.28 g·mol^{-1}

The title compound was prepared following the **GP2** using *N*-allylpiperidine (**2e**, 63.3 mg, 0.51 mmol, 1.00 equiv.), propionyl chloride (**1a**, 66.0 µL, 0.76 mmol, 1.50 equiv.), and Hünig's base (88.0 µL, 0.51 mmol, 1.00 equiv.). After a dry loaded column chromatography (SiO_2, pentane:EtOAc 1:1) the product **3ae** was obtained as yellow oil (21.0 mg, 0.12 mmol, 23%).

R$_f$ = 0.55 (pentane:EtOAc 1:1), UV-active, stains with $KMnO_4$.

^{1}H NMR ($CDCl_3$, 600 MHz): δ = 5.76 (dddd, *J* = 16.8, 10.1, 7.6, 6.4 Hz, 1H), 5.04 (dq, *J* = 17.1, 1.6 Hz, 1H), 4.99 (ddt, *J* = 10.2, 2.1, 1.1 Hz, 1H), 3.55 (dddd, *J* = 40.4, 13.1, 6.8, 4.4 Hz, 2H), 3.50–3.37 (m, 2H), 2.75 (sextet, *J* = 6.9 Hz, 1H), 2.42 (dtt, *J* = 14.4, 6.6, 1.4 Hz, 1H), 2.22–2.03 (m, 1H), 1.64 (pd, *J* = 5.7, 1.8 Hz, 2H), 1.59–1.45 (m, 4H), 1.10 (d, *J* = 6.8 Hz, 3H) ppm.

^{13}C{^{1}H} NMR ($CDCl_3$, 151 MHz): δ = 174.2, 136.6, 116.4, 46.7, 43.0, 38.3, 35.4, 26.9, 25.9, 24.8, 17.5 ppm.

IR (ATR): 1/λ = 3485 (w), 3075 (w), 2931 (s), 2856 (m), 2166 (m), 2010 (w), 1757 (w), 1635 (s), 1436 (s), 1366 (m), 1243 (s), 1215 (s), 1122 (m), 1007 (s), 952 (w), 910 (m), 852 (w), 803 (w), 719 (w) cm^{-1}.

CI-MS (100 eV, Methane): *m/z* (%): 363 (7) [2*M*+H]$^+$, 182 (100) [*M*+H]$^+$, 181 (7) [*M*]$^+$.

EI-MS (70 eV): *m/z* (%): 182 (100) [*M*+H]$^+$, 181 (73) [*M*]$^+$, 166 (33), 140 (24), 139 (28), 138 (20), 112 (33), 111 (26), 86 (13), 84 (35), 69 (31), 56 (11).

HRMS (ESI): *m/z* calcd for $C_{11}H_{19}NO+Na^+$ [*M*+Na]$^+$: 204.1359; found: 204.1358.

2-Methyl-1-(4-phenylpiperazin-1-yl)pent-4-en-1-one (3af)

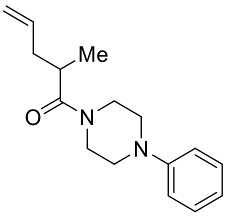

$C_{16}H_{22}N_2O$
m/z: 258.1732
M_W: 258.37 g·mol^{-1}

The title compound was prepared following the **GP2** using 1-allyl-4-phenylpiperazine (**2f**, 101 mg, 0.50 mmol, 1.00 equiv.), propionyl chloride (**1a**, 66.0 µL, 0.75 mmol, 1.50 equiv.), and Hünig's base (87.0 µL, 0.50 mmol, 1.00 equiv.). After a dry loaded column chromatography (SiO_2, pentane:EtOAc 4:1 → 1:1) the product **3af** was obtained as yellow oil (72.2 mg, 0.28 mmol, 56%).

R$_f$ = 0.54 (pentane:EtOAc 1:1), stains with $KMnO_4$.

^{1}H NMR (CDCl$_3$, 600 MHz): δ = 7.31–7.26 (m, 2H), 6.96–6.89 (m, 3H), 5.78 (dddd, *J* = 16.8, 10.2, 7.6, 6.5 Hz, 1H), 5.07 (dq, *J* = 17.1, 1.6 Hz, 1H), 5.03 (ddt, *J* = 10.2, 2.1, 1.1 Hz, 1H), 3.86–3.74 (m, 2H), 3.73–3.63 (m, 2H), 3.22–3.10 (m, 4H), 2.80 (sextet, *J* = 6.9 Hz, 1H), 2.46 (dtt, *J* = 14.9, 6.7, 1.4 Hz, 1H), 2.16 (m, 1H), 1.16 (d, *J* = 6.8 Hz, 3H) ppm.

^{13}C{^{1}H} NMR (CDCl$_3$, 151 MHz): δ = 174.5, 151.1, 136.2, 129.4 (2C), 120.7, 116.8, 116.7 (2C), 50.1, 49.7, 45.6, 41.8, 38.3, 35.5, 17.5 ppm.

IR (ATR): $1/\lambda$ = 3478 (w), 3275 (w), 3066 (w), 2972 (m), 2910 (m), 2819 (m), 2329 (w), 2084 (w), 1922 (w), 1732 (w), 1639 (s), 1598 (s), 1497 (s), 1435 (s), 1375 (m), 1336 (m), 1276 (m), 1225 (s), 1154 (m), 1095 (w), 1021 (s), 909 (s), 757 (s), 693 (m) cm^{-1}.

CI-MS (100 eV, Methane): *m/z* (%): 259 (100) [*M*+H]$^+$, 258 (11) [*M*]$^+$.

EI-MS (70 eV): *m/z* (%): 259 (47) [*M*+H]$^+$, 258 (100) [*M*]$^+$, 161 (15), 132 (52), 120 (14), 56 (11).

HRMS (ESI): *m/z* calcd for $C_{16}H_{22}ON_2$+Na$^+$ [*M*+Na]$^+$: 281.1624; found: 281.1621.

***N*-Benzyl-*N*,2-dimethylpent-4-enamide (3ag)**

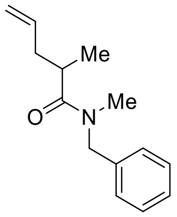

$C_{14}H_{19}NO$
m/z: 217.1467
M_W: 217.31 g·mol^{-1}

The title compound was prepared following the **GP2** using *N*-benzyl-*N*-methylprop-2-en-1-amine (**2g**, 80.3 mg, 0.50 mmol, 1.00 equiv.), propionyl chloride (**1a**, 66.0 µL, 0.75 mmol, 1.50 equiv.), and Hünig's base (87.0 µl, 0.50 mmol, 1.00 equiv.). After two dry loaded column chromatographies (SiO$_2$, 1st: pentane:EtOAc 2:1 → 1:1, 2nd: 9:1 → 6:1 → 4:1 → 2:1) the product was obtained as yellow oil (31.6 mg, 0.15 mmol, 29%). (***Note***: The NMR spectra were recorded at an elevated temperature as the product **3ag** was observed to be a mixture of rotamers at room temperature.)

R$_f$ = 0.26 (pentane:EtOAc 4:1), stains with KMnO$_4$.

^{1}H NMR (100 °C, DMSO-d$_6$, 400 MHz): δ = 7.37–7.30 (m, 2H), 7.29–7.18 (m, 3H), 5.78 (dq, *J* = 16.9 Hz, 7.8 Hz, 1H), 5.07–4.94 (m, 2H), 4.63–4.48 (m, 2H), 2.95–2.83 (m, 4H), 2.35 (m, 1H), 2.08 (m, 1H), 1.06 (d, *J* = 6.8 Hz, 3H) ppm.

^{13}C{^{1}H} NMR (100 °C, DMSO-d$_6$, 101 MHz): δ = 174.7, 137.5, 135.9, 127.9 (2C), 126.6, 126.4 (2C), 115.5, 37.3, 34.2, 33.8, 16.5 ppm.

IR (ATR): $1/\lambda$ = 3486 (w), 3276 (w), 3068 (w), 3029 (w), 2972 (m), 2929 (m), 2328 (w), 2092 (w), 1883 (w), 1759 (w), 1721 (w), 1639 (s), 1450 (s), 1407 (m), 1355 (w), 1256 (w), 1202 (w), 1086 (m), 1027 (w), 994 (m), 913 (m), 810 (w), 732 (s), 699 (s) cm^{-1}.

CI-MS (100 eV, Methane): *m/z* (%): 435 (22) [2*M*+H]$^+$, 434 (1) [2*M*]$^+$, 218 (100) [*M*+H]$^+$, 217 (7) [*M*]$^+$.

EI-MS (70 eV): *m/z* (%): 218 (46) [*M*+H]$^+$, 217 (85) [*M*]$^+$, 216 (28), 202 (30), 176 (10), 175 (15), 174 (48), 126 (20), 120 (21), 118 (19), 92 (11), 91 (100), 69 (21), 65 (14).

HRMS (ESI): *m/z* calcd for $C_{14}H_{19}NO+Na^+$ $[M+Na]^+$: 240.1359; found: 240.1355.

2-Methyl-*N*,*N*-dipropylpent-4-enamide (3ah)

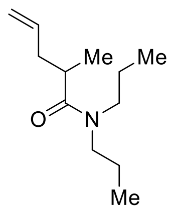

$C_{12}H_{23}NO$
m/z: 197.1780
M_W: 197.32 g·mol^{-1}

The title compound was prepared following the **GP2** using *N*,*N*-diprop-2-en-1-amine (**2h**, 86.7 mg, 0.61 mmol, 1.00 equiv.), propionyl chloride (**1a**, 80.0 µL, 0.92 mmol, 1.50 equiv.), and Hünig's base (107 µL, 0.61 mmol, 1.00 equiv.). After a dry loaded column chromatography (SiO_2, pentane:EtOAc 9:1) the product **3ah** was obtained as yellow oil (55.2 mg, 0.28 mmol, 46%).

$\mathbf{R_f}$ = 0.34 (pentane:EtOAc 9:1), stains with $KMnO_4$.

^{1}H NMR ($CDCl_3$, 600 MHz): δ = 5.72 (dddd, *J* = 16.9, 10.1, 7.7, 6.5 Hz, 1H), 5.02 (dq, *J* = 17.0, 1.5 Hz, 1H), 4.95 (ddt, *J* = 10.2, 2.1, 1.0 Hz, 1H), 3.29 (m, 1H), 3.23–3.09 (m, 4H), 2.65 (sextet, *J* = 6.9 Hz, 1H), 2.39 (dddt, *J* = 14.0, 7.7, 6.5, 1.4 Hz, 1H), 2.08 (dddt, *J* = 14.0, 7.7, 6.6, 1.1 Hz, 1H), 1.60–1.45 (m, 4H), 1.08 (dd, *J* = 6.8, 0.7 Hz, 3H), 0.88 (t, *J* = 7.4 Hz, 3H), 0.84 (t, *J* = 7.4 Hz, 3H) ppm.

^{13}C{^{1}H} NMR ($CDCl_3$, 151 MHz): δ = 175.8, 136.4, 116.5, 49.6, 47.9, 38.8, 35.8, 22.9, 21.1, 17.9, 11.4, 11.3 ppm.

IR (ATR): $1/\lambda$ = 3481 (w), 3266 (w), 3076 (w), 2964 (s), 2933 (m), 2875 (m), 2326 (w), 2087 (w), 1999 (w), 1838 (w), 1761 (w), 1637 (s), 1429 (s), 1374 (m), 1301 (w), 1234 (m), 1216 (m), 1098 (m), 998 (m), 910 (m), 749 (m), 671 (w) cm^{-1}.

CI-MS (100 eV, Methane): *m/z* (%): 198 (100) $[M+H]^+$, 197 (13) $[M]^+$.

EI-MS (70 eV): *m/z* (%): 395 (2) $[2M+H]^+$, 198 (100) $[M+H]^+$, 197 (37) $[M]^+$, 168 (14), 126 (11), 72 (27), 69 (15).

HRMS (ESI): *m/z* calcd for $C_{12}H_{23}NO+Na^+$ $[M+Na]^+$: 220.1672; found: 220.1668.

1-Morpholinopent-4-en-1-one (3ba)

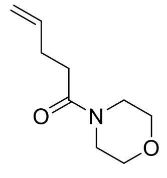

$C_9H_{15}NO_2$
m/z: 169.1103
M_W: 169.22 g·mol^{-1}

The title compound was prepared following the **GP2** using *N*-allylmorpholine (**2a**, 63.3 mg, 0.50 mmol, 1.00 equiv.), acetyl chloride (**1b**, 54.0 µL, 0.75 mmol, 1.50 equiv.), and Hünig's base (87.0 µL, 0.50

mmol, 1.00 equiv.). After a dry loaded column chromatography (SiO_2, pentane:EtOAc 1:1) the product **3ba** was obtained as yellow oil (14.0 mg, 0.08 mmol, 17%). Keeping everything the same but using acetyl bromide (55.0 µL, 0.75 mmol, 1.50 equiv.) instead of acetyl chloride increased the yield slightly (17.0 mg, 0.10 mmol, 20%). The NMR data closely match the ones previously reported in the literature.[20]

R_f = 0.33 (pentane:EtOAc 1:1), stains with $KMnO_4$.

^{1}H NMR ($CDCl_3$, MHz): δ = 5.85 (m, 1H), 5.06 (m, 1H), 5.00 (m, 1H), 3.69–3.64 (m, 4H), 3.63–3.59 (m, 2H), 3.49–3.43 (m, 2H), 2.40 (m, 4H) ppm.

^{13}C{^{1}H} NMR ($CDCl_3$, 151 MHz): δ = 171.1, 137.4, 115.5, 67.1, 66.8, 46.1, 42.1, 32.4, 29.3 ppm.

IR (ATR): $1/\lambda$ = 3489 (w), 3273 (w), 3075 (w), 2966 (m), 2916 (m), 2856 (m), 2326 (w), 2225 (w), 2110 (w), 1761 (w), 1639 (s), 1431 (s), 1365 (w), 1269 (m), 1224 (s), 1113 (s), 1067 (m), 1025 (m), 961 (m), 913 (s), 848 (m), 799 (w), 737 (w) cm^{-1}.

CI-MS (100 eV, Methane): *m/z* (%): 339 (5) $[2M+H]^+$, 170 (100) $[M+H]^+$, 169 (9) $[M]^+$.

EI-MS (70 eV): *m/z* (%): 339 (1) $[2M+H]^+$, 170 (100) $[M+H]^+$, 169 (92) $[M]^+$, 140 (20), 126 (19), 114 (62), 88 (15), 87 (12), 86 (39), 70 (16), 57 (44), 56 (33), 55 (52).

3.5 Unsuccessful Reactions

3.5.1 Varying the Acyl Chloride

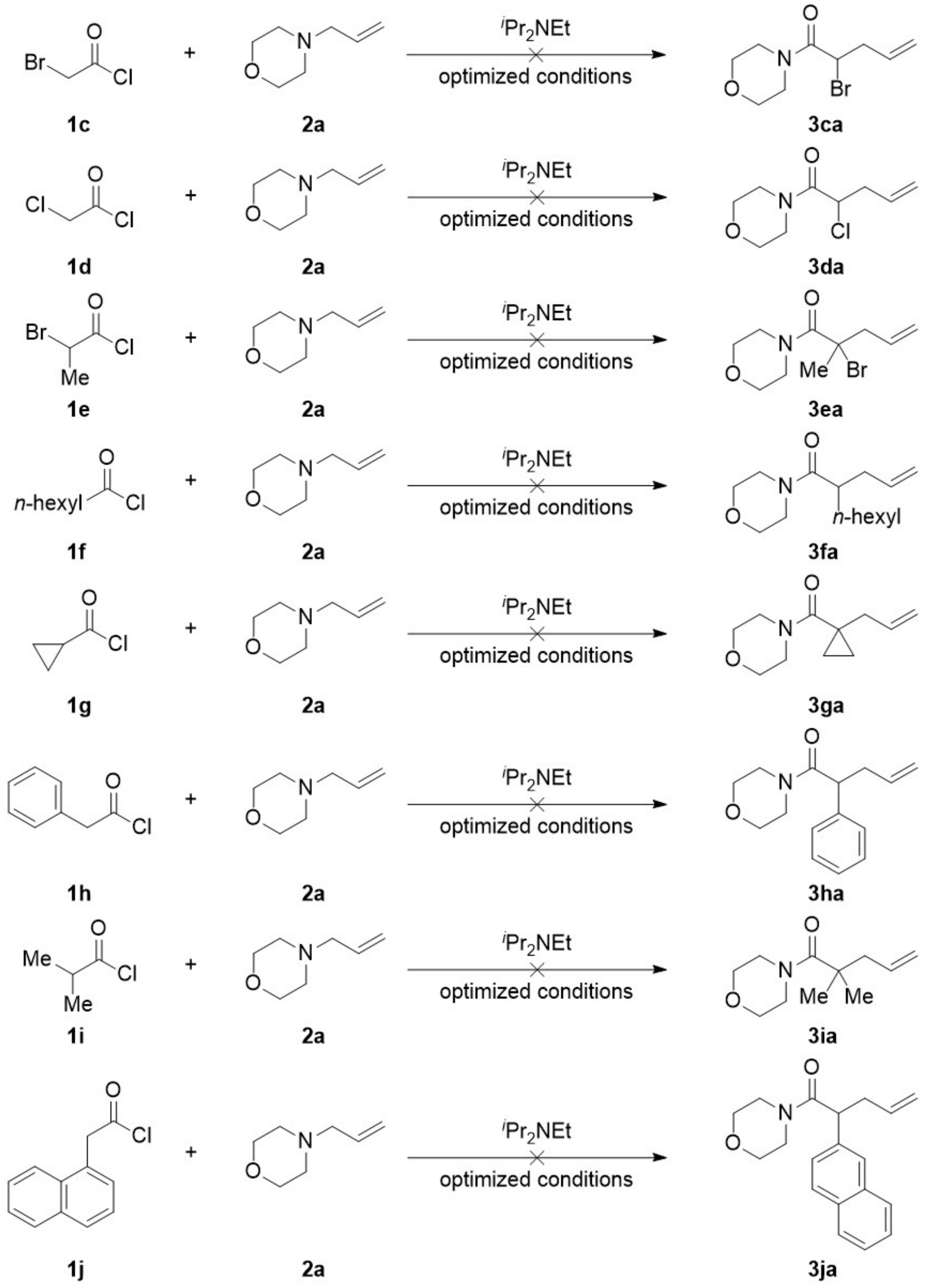

Scheme S1. Unsuccessful acyl chlorides under the optimized conditions.

3.5.2 Varying the Allylamine

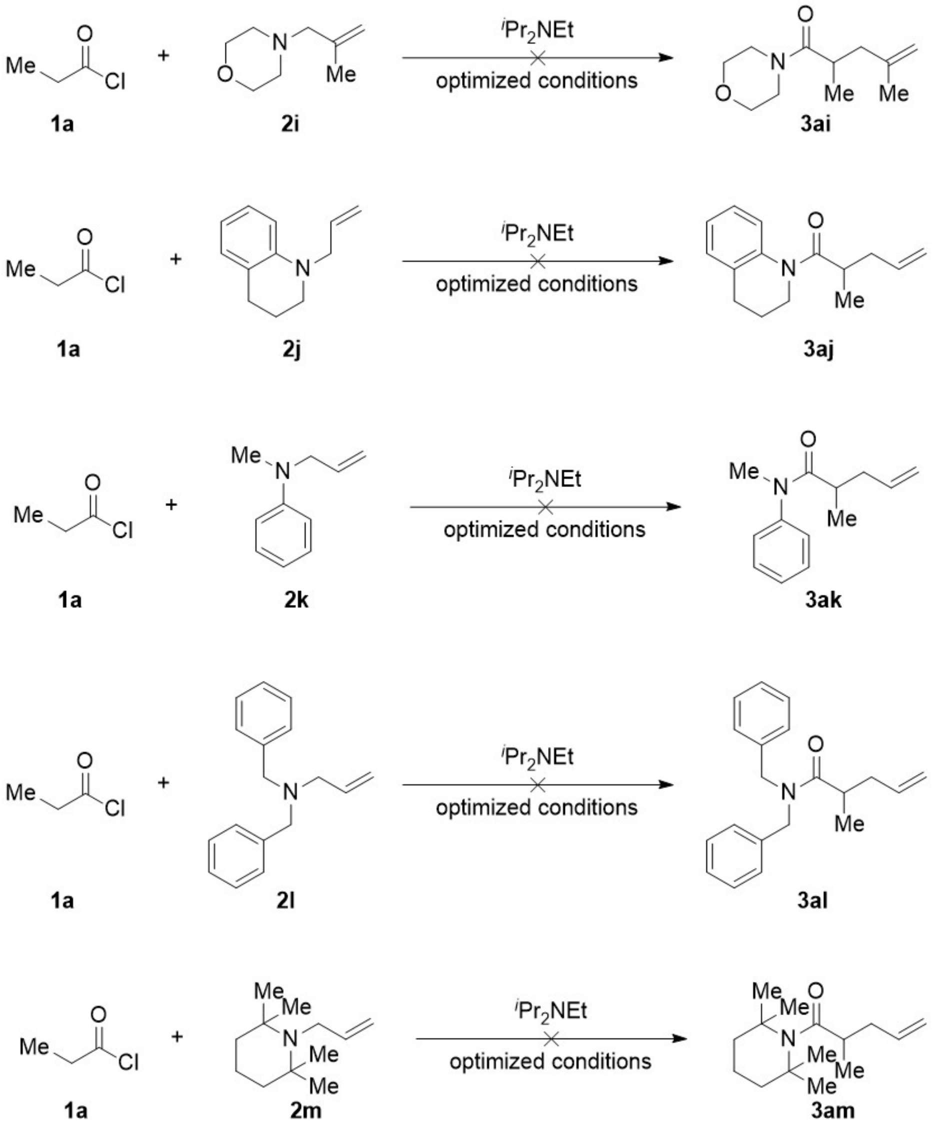

Scheme S2. Unsuccessful substrates under the optimized conditions.

4 Extension towards a Charge-accelerated Belluš-Claisen Type Rearrangement

4.1 Considered and Tested Synthetic Routes

Route 1. *N*-alkylation followed by vinylation.[22]

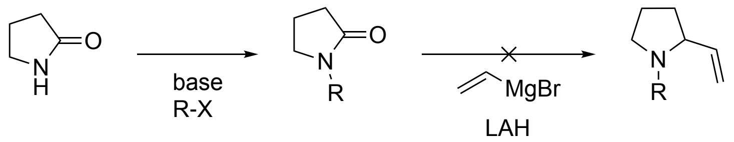

Route 2. *N*-alkylation followed by reductive alkylation by amide activation.[23]

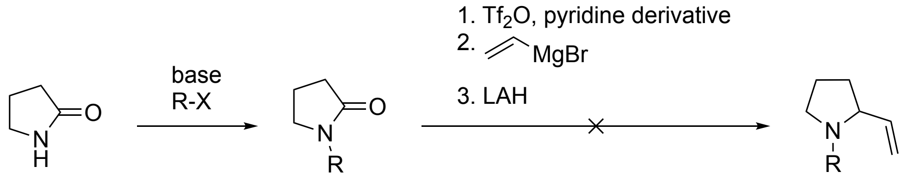

Route 3. Reductive amination, followed by N-alkylation and cyclization.

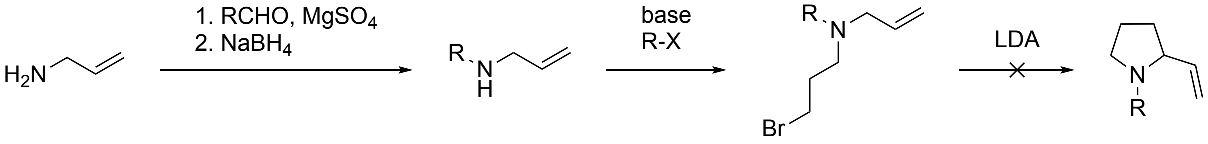

Route 4. *N*-alkylation followed by decarbonylative vinylation.[24]

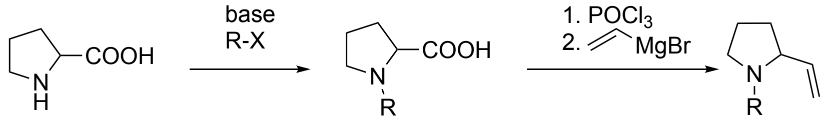

Scheme S3. Tested routes for the synthesis of 2-vinylpyrrolidines.[22–24]

4.2 Synthesis and Characterization of the Starting Materials

***N*-Benzylpyrrolidone (S1)**

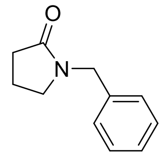

$C_{11}H_{13}NO$
m/*z*: 175.0997
M_W: 175.23 g·mol^{-1}

The title compound was prepared according to a modified literature procedure.[24]

A 250 mL-round bottom flask equipped with a magnetic stirring bar was charged with 2-pyrrolidinone (4.24 g, 49.9 mmol, 1.00 equiv.) which was dissolved in dry THF (100 mL). The solution was cooled to 0 °C using an ice bath. Then, NaH (60% in mineral oil, 2.20 g, 54.9 mmol, 1.10 equiv.) was added at 0 °C, the suspension was stirred for 1 h at this temperature followed by another hour at room temperature. Next, benzyl bromide (6.57 mL, ρ = g·mL^{-1}, 54.9 mmol, 1.10 equiv.) was added dropwise at room temperature and the reaction mixture was stirred for 2 days. The reaction was stopped by adding 50 mL of an aqueous saturated NH_4Cl solution, and was further diluted with 50 mL of water. The aqueous phase was extracted with EtOAc (3×50 mL), the organic phases were combined, dried over MgSO4, and the solvent removed under reduced pressure. The crude product was placed on top of a silica pad and thoroughly washed with pentane to remove remaining mineral oil and benzyl bromide. Then, the collecting flask was changed, and the product was rinsed into the new flask using EtOAc. The solvent was removed under reduced pressure and the title compound was obtained as yellow liquid in quantitative yield. The NMR data closely match the ones previously reported in the literature.[25]

^{1}H NMR ($CDCl_3$, 600 MHz): δ = 7.39–7.30 (m, 2H), 7.27 (m, 1H), 7.25–7.22 (m, 2H), 4.44 (s, 2H), 3.25 (m, 2H), 2.44 (t, *J* = 8.1 Hz, 2H), 1.98 (m, 2H) ppm.

$^{13}C\{^1H\}$ NMR ($CDCl_3$, 151 MHz): δ = 175.1, 136.7, 128.8 (2C), 128.2 (2C), 127.6, 46.71, 46.69, 31.1, 17.8 ppm.

IR (ATR): 1/λ = 3536 (w), 3030 (w), 2916 (w), 2326 (w), 2093 (w), 2005 (w), 1955 (w), 1678 (s), 1494 (m), 1425 (s), 1358 (w), 1284 (m), 1263 (s), 1166 (w), 1081 (w), 1025 (w), 947 (w), 922 (w), 851 (w), 815 (w), 736 (m), 700 (m), 661 (w) cm^{-1}.

CI-MS (100 eV, Methane): *m*/*z* (%): 351 (55) [2*M*+H]$^+$, 350 (1) [2*M*]$^+$, 176 (100) [*M*+H]$^+$, 175 (19) [*M*]$^+$.

EI-MS (70 eV): *m*/*z* (%): 176 (35) [*M*+H]$^+$, 175 (100) [*M*]$^+$, 174 (17), 146 (46), 118 (14), 104 (26), 92 (15), 91 (55), 84 (19), 66 (12), 55 (12).

***N*-Benzylprop-2-en-1-amine (S2)**

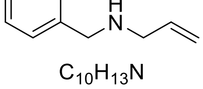

$C_{10}H_{13}N$
m/*z*: 147.1048
M_W: 147.22 g·mol^{-1}

A 250 mL-round bottom flask equipped with a magnetic stirring bar was charged with benzaldehyde (5.30 g, 49.9 mmol, 1.00 equiv.), which was dissolved in DCM (90 mL). Then, $MgSO_4$ (6.01 g, 49.9 mmol, 1.00 equiv.) was added. A 4 mL-GC vial was charged with allylamine (2.85 g, 49.9 mmol, 1.00 equiv.), which was then added to the reaction mixture using a pipette. The GC vial was rinsed with DCM (10 mL) to ensure a complete transfer. The reaction mixture was stirred at room temperature for 18 h, and then filtered using a Buchner funnel to remove the drying agent. The filtrate was concentrated to give a yellow residue that was dissolved in MeOH (200 mL). The solution was cooled in an ice bath to 0 °C. At this temperature, $NaBH_4$ (2.84 g, 75.0 mmol, 1.50 equiv.) was added in portions and the reaction mixture was allowed to warm up to room temperature in the ice bath over 22 h. The solvent was removed and the residue partitioned between water (175 mL) and EtOAc (75 mL). After phase separation, the aqueous phase was extracted with EtOAc (2×75 mL), the organic phases were combined and dried over Na_2SO_4. The solvent was removed to give the product as slightly yellow oil (5.83 g, 39.6 mmol, 79%), which was of sufficient purity for the next step. The NMR data closely match the ones previously reported in the literature.[26]

^{1}H NMR (CDCl$_3$, 600 MHz): δ = 7.39–7.22 (m, 5H), 5.95 (m, 1H), 5.21 (ddq, *J* = 17.1, 3.5, 1.7 Hz, 1H), 5.13 (m, 1H),3.79 (d, *J* = 3.1 Hz, 2H), 3.28 (ddt, *J* = 6.1, 2.3, 1.4 Hz, 2H), 1.63 (br s, 1H) ppm.

^{13}C{^{1}H} NMR (CDCl$_3$, 151 MHz): δ = 140.2, 136.8, 128.3 (2C), 128.1 (2C), 126.9, 115.9, 53.2, 51.7 ppm.

IR (ATR): $1/\lambda$ = 3872 (w), 3651 (w), 3314 (w), 3066 (m), 3027 (m), 2979 (m), 2914 (m), 2811 (s), 2325 (m), 2084 (w), 1989 (w), 1812 (m), 1642 (m), 1603 (m), 1493 (m), 1451 (s), 1359 (m), 1200 (w), 1140 (m), 1105 (s), 1028 (m), 993 (s), 916 (s), 819 (m), 735 (s), 698 (s) cm^{-1}.

CI-MS (100 eV, Methane): *m/z* (%): 148 (23) [*M*+H]$^+$, 147 (3) [*M*]$^+$.

EI-MS (70 eV): *m/z* (%): 148 (1) [*M*+H]$^+$, 147 (9) [*M*]$^+$, 146 (29), 92 (23), 91 (100), 65 (11), 56 (18).

***N*-Benzyl-*N*-(3-bromopropyl)prop-2-en-1-amine (S3a)**

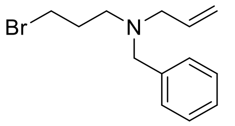

$C_{13}H_{18}BrN$
m/z: 267.0623
M_W: 268.20 g·mol^{-1}

A 25 mL-reaction tube equipped with a magnetic stirring bar was charged with 1,3-dibromopropane (10.1 g, 50.0 mmol, 50.0 equiv.) and K_2CO_3 (138 mg, 1.00 mmol, 1.00 equiv.) in the given order. Then, *N*-benzylprop-2-en-1-amine (147 mg, 1.00 mmol, 1.00 equiv.) was added dropwise with the help of a syringe. The reaction mixture was stirred at room temperature for 19 h. The excess of 1,3-dibromopropane was removed by vacuum distillation and was reused (purity checked by ^{1}H NMR). The remaining crude product was purified by column chromatography (SiO_2, pentane:EtOAc 4:1) to yield the title compound as colorless oil (163 mg, 0.61 mmol, 61%).

R_f = 0.80 (pentane:EtOAc 4:1), slightly UV-active, stains with $KMnO_4$.

^{1}H NMR (CDCl$_3$, 600 MHz): δ = 7.34–7.28 (m, 4H), 7.24 (m, 1H), 5.87 (ddt, *J* = 16.7, 10.2, 6.4 Hz, 1H), 5.19 (dq, *J* = 17.2, 1.6 Hz, 1H), 5.15 (ddt, *J* = 10.2, 2.1, 1.2 Hz, 1H), 3.57 (s, 2H), 3.44 (t, *J* = 6.8 Hz, 2H), 3.07 (dt, *J* = 6.4, 1.4 Hz, 2H), 2.58 (t, *J* = 6.7 Hz, 2H), 2.01 (p, *J* = 6.7 Hz, 2H) ppm.

$^{13}C\{^{1}H\}$ NMR ($CDCl_3$, 151 MHz): δ= 139.6, 135.9, 128.9 (2C), 128.4 (2C), 127.1, 117.6, 58.4, 57.0, 51.7, 32.1, 30.8 ppm.

IR (ATR): $1/\lambda$ = 3881 (w), 3434 (w), 3068 (m), 3027 (m), 2928 (m), 2801 (s), 2323 (w), 2082 (w), 1811 (w), 1743 (w), 1641 (w), 1602 (w), 1493 (w), 1450 (s), 1366 (m), 1256 (m), 1212 (m), 1150 (w), 1118 (m), 1069 (m), 1025 (m), 990 (s), 918 (s), 869 (m), 824 (m), 736 (s), 698 (s), 659 (w) cm^{-1}.

CI-MS (100 eV, Methane): *m/z* (%): 270 (24) $[M+H]^+$ (^{81}Br), 269 (14) $[M]^+$ (^{81}Br), 268 (44) $[M+H]^+$ (^{79}Br), 267 (4) $[M]^+$ (^{79}Br).

EI-MS (70 eV): *m/z* (%): 270 (2) $[M+H]^+$ (^{81}Br), 269 (12) $[M]^+$ (^{81}Br), 268 (3) $[M+H]^+$ (^{79}Br), 267 (6) $[M]^+$ (^{79}Br), 161 (12), 160 (100), 91 (91).

HRMS (ESI): *m/z* calcd for $C_{13}H_{18}{}^{79}BrN+H^+$ $[M+H]^+$: 268.0695; found: 268.0695.

***N*^{1},*N*^{3}-Diallyl-*N*^{1},*N*^{3}-dibenzylpropane-1,3-diamine (S3b)**

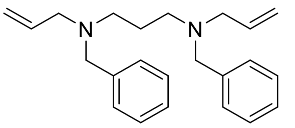

$C_{23}H_{30}N_2$
m/z: 334.2409
M_W: 334.51 g·mol^{-1}

A 25 mL-round bottom flask equipped with a magnetic stirring bar was charged with 1,3-dibromopropane (206 mg, 1.02 mmol, 1.00 equiv.) that was dissolved in MeCN (2.5 mL). Next, KI (16.5 mg, 0.10 mmol, 10 mol%), and Na_2CO_3 (106 mg, 1.00 mmol, 0.98 equiv.) were added in the given order. Then, a 4 mL-GC vial was charged with *N*-benzylprop-2-en-1-amine (150 mg, 1.022 mmol, 1.00 equiv.) that was dissolved in MeCN (2.5 mL). This solution was added dropwise to the reaction flask over the time of 30 minutes using a syringe pump. Next, the reaction mixture was stirred at 70 °C in an oil bath for 17 h until it was cooled down to room temperature. The solid material was filtered off using a filter paper that was washed with MeCN. The solvent was removed and the crude product was purified by running a dry loaded column chromatography (SiO_2, pentane:EtOAc 98:2 → 95:5 → 9:1 → 4:1 → 3:1) to yield the title compound as orange oil (109 mg, 0.33 mmol, 32%).

R_f = 0.25 (pentane:EtOAc 4:1), slightly UV-active, smears, stains with $KMnO_4$ (without heating) and/or I_2@SiO_2.

^1H NMR ($CDCl_3$, 600 MHz): δ= 7.49–7.11 (m, 10 H), 5.91 (ddtd, *J* = 16.8, 10.2, 6.4, 1.5 Hz, 2H), 5.22 (dq, *J* = 17.2, 1.6 Hz, 2H), 5.17 (m, 2H), 3.59 (s, 4H), 3.10 (m, 4H), 2.50 (m, 4H), 1.73 (m, 2H) ppm.

$^{13}C\{^{1}H\}$ NMR ($CDCl_3$, 151 MHz): δ= 139.8 (2C), 136.2 (2C), 129.0 (4C), 128.2 (4C), 126.8 (2C), 117.2 (2C), 58.2 (2C), 56.9 (2C), 51.6 (2C), 24.6 ppm.

IR (ATR): $1/\lambda$ = 3067 (w), 3027 (m), 2932 (m), 2795 (s), 2156 (w), 2013 (w), 1950 (w), 1813 (w), 1642 (w), 1601 (w), 1493 (m), 1450 (s), 1365 (m), 1328 (m), 1253 (m), 1152 (m), 1123 (m), 1072 (w), 1048 (w), 1029 (w), 993 (m), 916 (s), 844 (w), 821 (w), 735 (s), 697 (s) cm^{-1}.

CI-MS (100 eV, Methane): *m/z* (%): 336 (15) $[M+2H]^+$, 335 (86) $[M+H]^+$, 334 (4) $[M]^+$.

EI-MS (70 eV): *m/z* (%): 336 (1) $[M+2H]^+$, 335 (4) $[M+H]^+$, 293 (21), 243 (17), 187 (16), 174 (24), 161 (11), 160 (60), 146 (17), 96 (10), 91 (100).

HRMS (ESI): *m/z* calcd for $C_{23}H_{30}N_2+H^+$ $[M+H]^+$: 335.2482; found: 335.2473.

***N*-Benzylproline (S4a)**

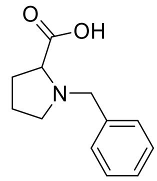

$C_{12}H_{15}NO_2$
m/z: 205.1103
M_W: 205.26 g·mol^{-1}

The title compound was prepared following a modified literature procedure.[27]

A 250 mL-round bottom flask equipped with a magnetic stirring bar was charged with L-proline (2.30 g, 20.0 mmol, 1.00 equiv.), KOH (4.22 g, 75.3 mmol, 3.76 equiv.), and *iso*-propanol (100 mL) in the given order. The solution was heated to 40 °C in an oil bath. At this temperature benzyl bromide (3.80 mL, 31.1 mmol, 1.56 equiv.) was added dropwise. The reaction was allowed to stir at 40 °C for 18 h. After the mixture cooled down to room temperature, the pH was adjusted to 1–2 using conc. $HCl_{(aq.)}$ and chloroform (150 mL) was added. The mixture stirred for additional 23 h. The precipitate was removed by filtration using a Buchner funnel and the filtrate was concentrated under reduced pressure. The remaining deep brownish oil was treated with acetone (40 mL) and placed in the fridge (2 °C). The formed crystals were filtered off and the filtrate was cooled in the fridge again, leading to a new formation of precipitate. The crystallization cycle was repeated one more time and the collected fractions were combined. ^{1}H-NMR analysis revealed that a mixture of different species was obtained by this work-up procedure. Therefore, the combined solids were re-dissolved in a solution of KOH (3.27 g, 58.2 mmol) in methanol (50 mL). The pH was set to 4–5 using conc. $HCl_{(aq.)}$ and chloroform (100 mL) was added. After the reaction mixture stirred for 69 h, the precipitates were filtered of using a Buchner funnel, and a colorless oil was obtained after the filtrate was concentrated under reduced pressure. The crude product was recrystallized from acetone in the fridge (2 °C) to yield a colorless solid (1.68 g, 8.21 mmol, 41 %). The NMR data closely match the ones previously reported in the literature.[28]

m.p.: 169–174 °C.

^{1}H NMR (CD_3OD, 600 MHz): δ = 7.58–7.53 (m, 2H), 7.50–7.44 (m, 3H), 4.58 (d, *J* = 12.8 Hz, 1H), 4.39–4.33 (m, 2H), 3.55 (ddd, *J* = 11.6, 7.7, 4.2 Hz, 1H), 3.39 (ddd, *J* = 11.4, 9.1, 7.9 Hz, 1H), 2.60 (m, 1H), 2.26–2.11 (m, 2H), 2.00 (m, 1H) ppm. (***Note***: The CO_2H is not or observed or masked by the water peak at 4.91 ppm.)

^{13}C{^{1}H} NMR (CD_3OD, 151 MHz): δ = 171.1, 131.9 (2C), 131.6, 131.2, 130.3 (2C), 67.8, 59.7, 55.7, 29.4, 23.4 ppm.

IR (ATR): $1/\lambda$ = 3697 (w), 3460 (w), 3064 (w), 3037 (w), 2984 (w), 2915 (w), 2788 (m), 2658 (m), 2552 (w), 2496 (w), 2392 (w), 2344 (w), 2282 (w), 2203 (w), 2112 (m), 2021 (w), 1922 (w), 1944 (w), 1895 (w), 1723 (s), 1675 (s), 1496 (w), 1455 (m), 1402 (m), 1362 (m), 1308 (w), 1266 (w), 1197 (s), 1108 (w), 1064 (m), 1002 (m), 929 (m), 839 (s), 800 (w), 749 (s), 724 (s), 694 (s) cm^{-1}.

CI-MS (100 eV, Methane): *m/z* (%): 206 (100) $[M+H]^+$, 205 (3) $[M]^+$.

EI-MS (70 eV): *m/z* (%): 206 (1) $[M+H]^+$, 205 (1) $[M]^+$, 160 (55), 91 (100), 70 (11).

Potassium *N*-(4-bromobenzyl)prolinate monohydrate (S4b)

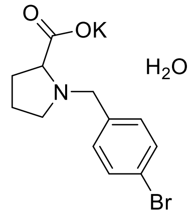

$C_{12}H_{15}BrKNO_3$
m/*z*: 338.9872
M_W: 340.26 g·mol^{-1}

The title compound was synthesized according to a modified literature procedure.[28]

A 250 mL-round bottom flask equipped with a magnetic stirring bar was charged with L-proline (2.30 g, 20.0 mmol, 1.00 equiv.), KOH (3.37 g, 60.0 mmol, 3.00 equiv.), and *iso*-propanol (100 mL) in the given order. The solution was heated in an oil bath to 40 °C. Then, 4-bromobenzyl bromide (5.50 g, 22.0 mmol, 1.10 equiv.) was added in one portion. The reaction mixture was stirred for 20 h at 40 °C before it was cooled down to room temperature. The mixture was acidified by addition of conc. $HCl_{(aq.)}$ to a pH = 3–4. Next, chloroform (100 mL) was added and the reaction mixture stirred for additional 69 h at room temperature. The formed precipitate was filtered off using a Buchner funnel and the filtrate was concentrated under reduced pressure. The crude product was recrystallized from acetone to yield a colorless solid (3.58 g, 10.5 mmol, 53 %).

m.p.: 202–207 °C.

^{1}H NMR (DMSO-d$_6$, 600 MHz): δ = 7.66 (m, 2H), 7.52 (m, 2H), 4.50 (d, *J* = 12.9 Hz, 1H), 4.40–4.34 (m, 2H), 3.47 (ddd, *J* = 11.6, 7.5, 4.3 Hz, 1H), 3.27 (dt, *J* = 11.0, 8.1 Hz, 1H), 2.48–2.40 (m, 1H), 2.10–1.96 (m, 2H), 1.92–1.82 (m, 1H) ppm. (***Note***: The total of 13 H would indicate the free carboxylic acid. But elemental analysis revealed the title compound was obtained as the monohydrate of the potassium salt. The appearance in the ^{1}H NMR spectrum is caused most likely by the water content of the used DMSO-d$_6$.)

^{13}C{^{1}H} NMR (DMSO-d$_6$, 151 MHz): δ = 169.7, 133.1 (2C), 131.7 (2C), 130.1, 123.1, 65.2, 56.5, 54.5, 28.0, 22.0 ppm.

IR (ATR): 1/λ = 3847 (w), 3417 (w), 2968 (m), 2765 (s), 2606 (s), 2479 (s), 2179 (w), 2113 (w), 1969 (w), 1920 (w), 1713 (s), 1593 (m), 1486 (m), 1450 (m), 1353 (s), 1280 (w), 1200 (s), 1090 (w), 1066 (m), 1003 (s), 978 (m), 927 (m), 887 (s), 818 (s), 748 (w), 719 (m), 676 (w) cm^{-1}.

CI-MS (100 eV, Methane): *m/z* (%): 322 (2) [*M*+H]$^+$, 286 (83) [*M* as free carboxylic acid+2H]$^+$, 284 (100) [*M* as free carboxylic acid]$^+$.

EI-MS (70 eV): *m/z* (%): 286 (3) [*M* as free carboxylic acid+2H]$^+$, 285 (4) [*M* as free carboxylic acid+H]$^+$, 284 (5) [*M* as free carboxylic acid]$^+$, 242 (12), 241 (41), 240 (100), 238 (98), 171 (40), 169 (38).

CHN: calcd (%) for $C_{12}H_{13}BrKNO_2·H_2O$: C 42.36, H 4.44, N 4.12; found: C 42.49, H 4.48, N 4.05.

N-Benzyl-2-vinylpyrrolidine (4)

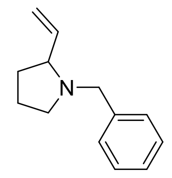

$C_{13}H_{17}N$
m/z: 187.1361
M_W: 187.29 g·mol^{-1}

The title compound was prepared according to a modified literature procedure.[23b]

A 10 mL-Schlenk tube equipped with *N*-benzylproline (**S4a**, 205 mg, 1.00 mmol, 1.00 equiv.). The tube was evacuated and flushed with argon (3×). Then, $POCl_3$ (0.20 mL, ρ = 1.65 g·mL^{-1}, 2.15 mmol, 2.15 equiv.) was added in an argon counterflow (***CAUTION***: gas evolution!). The reaction mixture was placed for 20 minutes in a preheated oil bath (100 °C) until the gas evolution ceased. After the reaction mixture cooled down to room temperature, distilled Et_2O (5 mL) was added, and the biphasic mixture was stirred for 5 minutes. The supernatant solution was taken off using a pipette. The washing procedure was performed thrice. The remaining residue was dried in vacuo. [***Note***: The presence of the desired iminium compound can be checked using ^{1}H NMR (CD_3CN, 300 MHz): δ = 8.52 (m, RN=C*H*R) ppm.] In the meantime, a 100 mL-Schlenk flask equipped with a magnetic stirring bar was evacuated and flushed with argon thrice. Then, vinylmagnesium bromide (0.8 M in THF, 5 mL, 4.00 mmol, 4.00 equiv.) was added and cooled to –78 °C using a cooling bath (dry ice/*iso*-propanol). The dried imine was dissolved in dry THF (1 mL), added dropwise to the cooled Grignard solution, and the resulting solution was stirred for 1 h at –78 °C. Next, the cooling bath was removed, and the reaction mixture stirred for 18 h at room temperature. Then, the reaction was stopped by adding MeOH (1 mL, ***CAUTION***: gas evolution). The reaction mixture was partitioned between water (5 mL), distilled Et_2O (5 mL) and 4 drops of Na_2CO_3. The biphasic reaction mixture was stirred for 5 minutes, the upper ethereal layer taken off using a pipette, and new ether was added. The cycle was repeated 5 times. The organic phases were combined, and the solvent was removed. The crude product was purified by running a dry loaded column chromatography (SiO_2, pentane:EtOAc 1:1) to yield the title compound as yellow oil (80.1 mg, 0.43 mmol, 43%). The NMR data closely match the ones previously reported in the literature.[29]

R_f = 0.66 (pentane:EtOAc 1:1), UV-active, stains with I_2@SiO_2.

^{1}H NMR ($CDCl_3$, 600 MHz): δ = 7.32–7.28 (m, 4H), 7.23 (m, 1H), 5.80 (ddd, *J* = 17.2, 10.1, 8.2 Hz, 1H), 5.22 (ddd, *J* = 17.2, 2.1, 0.7 Hz, 1H), 5.14 (dd, *J* = 10.1, 1.9 Hz, 1H), 4.03 (d, *J* = 13.0 Hz, 1H), 3.08 (d, *J* = 13.0 Hz, 1H), 2.94 (td, *J* = 8.8, 2.5 Hz, 1H), 2.80 (q, *J* = 8.1 Hz, 1H), 2.11 (q, *J* = 8.9 Hz, 1H), 1.96 (dddd, *J* = 12.4, 9.5, 7.1, 4.5 Hz, 1H), 1.82–1.60 (m, 3H) ppm.

^{13}C{^{1}H} NMR ($CDCl_3$, 151 MHz): δ = 141.2, 139.7, 129.2 (2C), 128.3 (2C), 126.9, 116.7, 68.6, 58.2, 53.4, 31.6, 22.2 ppm.

IR (ATR): 1/λ = 3882 (w), 3639 (w), 3433 (w), 3069 (m), 3028 (m), 2964 (s), 2874 (m), 2786 (s), 2717 (m), 2319 (m), 2184 (w), 2089 (m), 1996 (w), 1809 (m), 1748 (m), 1642 (m), 1604 (m), 1493 (m), 1452 (s), 1422 (m), 1368 (s), 1322 (m), 1298 (m), 1219 (m), 1148 (s), 1110 (s), 1074 (m), 1027 (m), 992 (s), 916 (s), 848 (m), 738 (s), 698 (s), 670 (m) cm^{-1}.

CI-MS (100 eV, Methane): *m/z* (%): 188 (100) [*M*+H]$^+$, 187 (17) [*M*]$^+$.

EI-MS (70 eV): *m/z* (%): 188 (1) [*M*+H]$^+$, 187 (10) [*M*]$^+$, 160 (17), 96 (14), 92 (13), 91 (100), 68 (17), 65 (19).

4.3 Synthesis and Characterization of the Product

1-Benzyl-3-methyl-1,3,4,7,8,9-hexahydro-2*H*-azonin-2-one (5)

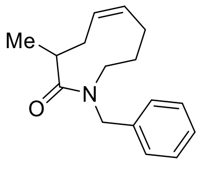

$C_{16}H_{21}NO$
m/z: 243.1623
M_W: 243.35 g·mol^{-1}

The title compound was prepared following the **GP2** using *N*-benzyl-2-vinylpyrrolidine (**4**, 97.1 mg, 0.52 mmol, 1.00 equiv.), propionyl chloride (68.0 µl, 0.78 mmol, 1.50 equiv.), and Hünig's base (90.0 µl, 0.52 mmol, 1.00 equiv.). After a dry loaded column chromatography (SiO_2, pentane:EtOAc 9:1) the product **5** was obtained as yellow oil (48.7 mg, 0.20 mmol, 39%).

$\mathbf{R_f}$ = 0.23 (pentane:EtOAc 9:1), stains with I_2@SiO_2.

^{1}H NMR ($CDCl_3$, 600 MHz): δ = 7.32–7.27 (m, 2H), 7.23 (m, 1H), 7.20–7.16 (m, 2H), 5.65 (ddd, *J* = 15.8, 10.7, 5.2 Hz, 1H), 5.44–5.34 (m, 2H), 3.91 (d, *J* = 15.0 Hz, 1H), 3.57 (dd, *J* = 14.6, 10.2 Hz, 1H), 2.99 (dd, *J* = 14.6, 5.3 Hz, 1H), 2.71 (dtd, *J* = 13.2, 7.7, 5.4 Hz, 1H), 2.36 (ddd, *J* = 10.6, 6.5, 3.3 Hz, 1H), 2.19 (q, *J* = 11.4 Hz, 1H), 2.11 (ddd, *J* = 12.3, 5.2, 2.2 Hz, 1H), 2.09–1.94 (m, 2H), 1.71 (m, 1H), 1.22 (dd, *J* = 6.6, 1.0 Hz, 3H) ppm.

^{13}C{^{1}H} NMR ($CDCl_3$, 151 MHz): δ = 176.4, 138.1, 132.9, 131.2, 128.6 (2C), 128.1 (2C), 127.2, 47.2, 44.7, 41.0, 37.9, 31.9, 27.9, 19.0 ppm.

IR (ATR): 1/λ = 3473 (w), 2930 (s), 2863 (m), 2327 (w), 2237 (w), 2160 (w), 2117 (w), 1891 (w), 1759 (w), 1621 (s), 1493 (m), 1452 (s), 1418 (s), 1361 (m), 1269 (w), 1235 (m), 1186 (s), 1142 (m), 1079 (m), 1030 (w), 982 (s), 919 (w), 872 (w), 802 (m), 731 (s), 700 (s) cm^{-1}.

CI-MS (100 eV, Methane): *m/z* (%): 244 (100) [*M*+H]$^+$, 243 (7) [*M*]$^+$.

EI-MS (70 eV): *m/z* (%): 244 (24) [*M*+H]$^+$, 243 (12) [*M*]$^+$, 242 (8), 174 (10), 152 (79), 151 (17), 124 (16), 91 (100), 65 (10), 55 (11).

HRMS (ESI): *m/z* calcd for $C_{16}H_{21}NO$ [*M*]$^+$: 243.1623; found: 243.1624.

5 References

[1] Mestrelab Research S.L. **2022**, Version 14.2.3-29241.

[2] Fulmer, G. R.; Miller, A. J. M.; Sherden, N. H.; Gottlieb, H. E.; Nudelman, A.; Stoltz, B. M.; Bercaw, J. E.; Goldberg, K. I. NMR Chemical Shifts of Trace Impurities: Common Laboratory Solvents, Organics, and Gases in Deuterated Solvents Relevant to the Organometallic Chemist. *Organometallics* **2010**, *29*, 2176–2179, DOI: 10.1021/om100106e.

[3] Ghosh, T.; Mukherji, A.; Kancharla, P. K. Influence of Anion-Binding Schreiner's Thiourea on DMAP Salts: Synergistic Catalysis toward the Stereoselective Dehydrative Glycosylation from 2-Deoxyhemiacetals. *J. Org. Chem.* **2021**, *86*, 1253–1261, DOI: 10.1021/acs.joc.0c02473.

[4] Štrukil, V.; Igrc, M. D.; Eckert-Maksić, M.; Friščić, T. Click Mechanochemistry: Quantitative Synthesis of "Ready to Use" Chiral Organocatalysts by Efficient Two-Fold Thiourea Coupling to Vicinal Diamines. *Chem. Eur. J.* **2012**, *18*, 8464–8473, DOI: 10.1002/chem.201200632.

[5] Kon, Y.; Nakashima, T.; Fujitani, T.; Murayama, T.; Ueda, W. Dehydrative Allylation of Amine with Allyl Alcohol by Titanium Oxide Supported Molybdenum Oxide Catalyst. *Synlett* **2019**, *30*, 287–292, DOI: 10.1055/s-0037-1612010.

[6] Wu, X.; Ding, G.; Lu, W.; Yang, L.; Wang, J.; Zhang, Y.; Xie, X.; Zhang, Z. Nickel-Catalyzed Hydrosilylation of Terminal Alkenes with Primary Silanes via Electrophilic Silicon–Hydrogen Bond Activation. *Org. Lett.* **2021**, *23*, 1434–1439, DOI: 10.1021/acs.orglett.1c00111.

[7] Xie, Y.; Hu, J.; Wang, Y.; Xia, C.; Huang, H. Palladium-Catalyzed Vinylation of Aminals with Simple Alkenes: A New Strategy To Construct Allylamines. *J. Am. Chem. Soc.* **2012**, *134*, 20613–20616, DOI: 10.1021/ja310848x.

[8] Tafazolian, H.; Schmidt, J. A. R. Cationic [(Iminophosphine)Nickel(Allyl)]+ Complexes as the First Example of Nickel Catalysts for Direct Hydroamination of Allenes. *Chem. Eur. J.* **2017**, *23*, 1507–1511, DOI: 10.1002/chem.201605611.

[9] Gui, J.; Xie, H.; Jiang, H.; Zeng, W. Visible-Light-Mediated Sulfonylimination of Tertiary Amines with Sulfonylazides Involving C_{sp}^3–C_{sp}^3 Bond Cleavage. *Org. Lett.* **2019**, *21*, 2804–2807, DOI: 10.1021/acs.orglett.9b00786.

[10] Henderson, M. A.; Luo, J.; Oliver, A.; McIndoe, J. S. The Pauson-Khand Reaction: A Gas-Phase and Solution-Phase. *Organometallics* **2011**, *30*, 5471–5479, DOI: 10.1021/om200717r.

[11] Bell, L.; Brookings, D. C.; Dawson, G. J.; Whitby, Asymmetric Ethylmagnesiation of Alkenes Using a Novel Zirconium Catalyst. *Tetrahedron* **1998**, *54*, 14617–14634, DOI: 10.1016/S0040-4020(98)00920-X.

[12] Radlauer, M. R.; Buckley, A. K.; Henling, L. M.; Agapie, T. Bimetallic Coordination Insertion Polymerization of Unprotected Polar Monomers: Copolymerization of Amino Olefins and Ethylene by Dinickel Bisphenoxyiminato Catalysts. *J. Am. Chem. Soc.* **2013**, *135*, 3784–3787, DOI: 10.1021/ja4004816.

[13] Douglas, G. E.; Raw, S. A.; Marsden, S. P. Iron-Catalysed Direct Aromatic Amination with *N*-Chloroamines. *Eur. J. Org. Chem.* **2019**, 5508–5514, DOI: 10.1002/ejoc.201900614.

[14] Zhou, J.; Li, L.; Wang, S.; Yan, M.; Wei, W. Catalyst-free photodecarbonylation of ortho-amino benzaldehyde. *Green Chem.* **2020**, *22*, 3421–3426, DOI: 10.1039/D0GC01256D.

[15] Wang, T.; Kehr, G.; Liu, L.; Grimme, S.; Daniliuc C. G.; Erker, G. Selective Oxidation of an Active Intramolecular Amine/Borane Frustrated Lewis Pair with Dioxygen. *J. Am. Chem. Soc.* **2016**, *138*, 4302–4305, DOI: 10.1021/jacs.6b00325.

[16] Krishnan, P.; Wu, M.; Chiang, M.; Li, Y.; Leung, P.-H.; Pullarkat, S. A. N-Heterocyclic Carbene C,S Palladium(II) π-Allyl Complexes: Synthesis, Characterization, and Catalytic Application In Allylic Amination Reactions. *Organometallics* **2013**, *32*, 2389–2397, DOI: 10.1021/om400110t.

[17] Yoon, T. P.; Dong, V. M.; MacMillan, D. W. C. Development of a New Lewis Acid-Catalyzed Claisen Rearrangement. *J. Am. Chem. Soc.* **1999**, *121*, 9726–9727, DOI: 10.1021/ja9919884.

[18] Dittrich, N.; Jung, E.-K.; Davidson, S. J.; Barker, D. An acyl-Claisen/Paal-Knorr approach to fully substituted pyrroles. *Tetrahedron* **2016**, *72*, 4676–7689, DOI: 10.1016/j.tet.2016.06.049.

[19] Niu, Z.-J.; Li, L.-H.; Li, X.-S.; Liu, H.-C.; Shi, W.-Y.; Liang, Y.-M. Formation of *o*-Allyl- and Allenyl-Modified Amides via Intermolecular Claisen Rearrangement. *Org. Lett.* **2021**, *23*, 1315–1320, DOI: 10.1021/acs.orglett.0c04300.

[20] Seastram, A. C; Hareram, M. D.; Knight, T. M. B.; Morrill, L. C. Electrochemical alkene azidocyanation *via* 1,4-nitrile migration. *Chem. Commun.* **2022**, *58*, 8658–8661, DOI: 10.1039/D2CC02958H.

[21] a) Bremner, J. B.; Perkins, D. F. Synthesis of functionalised azecine and azonine derivatives via an enolate assisted aza Claisen rearrangement. *Tetrahedron* **2005**, *61*, 2659–2665, DOI: 10.1016/j.tet.2005.01.061; b) Suh, Y.-G.; Kim, S.-A.; Jung, J.-K.; Shin, D.-Y.; Min, K.-H.; Koo, B.-A.; Kim; H.-S. Asymmetric Total Synthesis of Fluvirucinine A_1. *Angew. Chem. Int. Ed.* **1999**, *38*, 3545–3547, DOI: 10.1002/(SICI)1521-3773(19991203)38:23<3545::AID-ANIE3545>3.0.CO;2-0.

[22] Xiao, K.-J.; Wang, Y.; Ye, K.-Y.; Huang, P.-Q. Versatile One-Pot Reductive Alkylation of Lactams/Amides via Amide Activation: Application to the Concise Syntheses of Bioactive Alkaloids (±)-Bgugaine, (±)-Coniine, (+)-Preussin, and (−)-Cassine. *Chem. Eur. J.* **2010**, *16*, 12792–12796, DOI: 10.1002/chem.201002054.

[23] a) Dean, R. T.; Padgett, H. C.; Rapoport, H. A high yield regiospecific preparation of iminium salts. *J. Am. Chem. Soc.* **1976**, *98*, 7448–7449, DOI: 10.1021/ja00439a069; b) Vedejs, E.; Arco, M. J.; Powell, D. W.; Renga, J. M.; Singer, S. P. Ring expansion of 2-vinyl derivatives of thiane, N-benzylpiperidine, and thiepane by [2,3] sigmatropic shift. *J. Org. Chem.* **1978**, *43*, 4831–4837, DOI: 10.1021/jo00419a040.

[24] Xing, X.; O'Connor, N. R.; Stoltz, B. M. Palladium(II)-Catalyzed Allylic C–H Oxidation of Hindered Substrates Featuring Tunable Selectivity Over Extent of Oxidation. *Angew. Chem. Int. Ed.* **2015**, *54*, 11186–11190, DOI: 10.1002/anie.201504007.

[25] Griffiths, R. J.; Burley, G. A.; Talbot, E. P. A. Transition-Metal-Free Amine Oxidation: A Chemoselective Strategy for the Late-Stage Formation of Lactams. *Org. Lett.* **2017**, *19*, 870–873, DOI: 10.1021/acs.orglett.7b00021.

[26] Schumacher, C.; Hernández, J. G.; Bolm, C. Electro-Mechanochemical Atom Transfer Radical Cyclizations using Piezoelectric $BaTiO_3$. *Angew. Chem. Int. Ed.* **2020**, *59*, 16357–16360, DOI: 10.1002/anie.202003565.

[27] Spiegel, J.; Cromm, P. M.; Itzen, A.; Goody, R. S.; Grossmann, T. N.; Waldmann, H. Direct Targeting of Rab-GTPase–Effector Interactions. *Angew. Chem. Int. Ed.* **2014**, *53*, 2498–2503, 10.1002/anie.201308568.

[28] Yao, Y.; Li, R.; Liu, X.; Yang, F.; Yang, Y.; Li, X.; Shi, X.; Yuan, T.; Fang, L.; Du, G.; Jiao, X.; Xie, P. Discovery of Novel N-Substituted Prolinamido Indazoles as Potent Rho Kinase Inhibitors and Vasorelaxation Agents. *Molecules* **2017**, *22*, 1766, DOI: 10.3390/molecules22101766.

[29] Mukherjee, P.; Widenhoefer, R. A. Gold(I)-Catalyzed Intramolecular Amination of Allylic Alcohols With Alkylamines. *Org. Lett.* **2011**, *13*, 1334–1337, DOI: 10.1021/ol103175w.

6 Copy of NMR Spectra

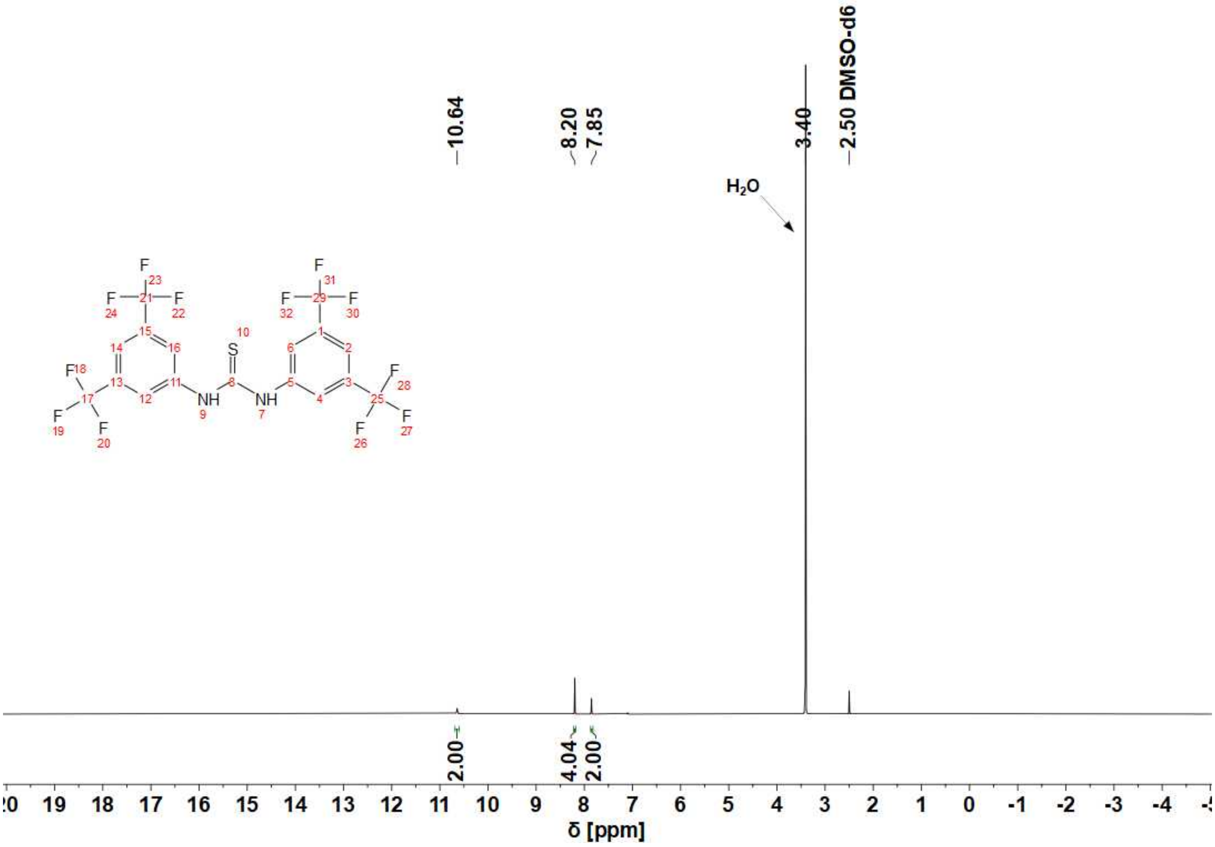

Figure S1: ^{1}H NMR spectrum (DMSO-d$_6$, 600 MHz) of Schreiner's thiourea catalyst.

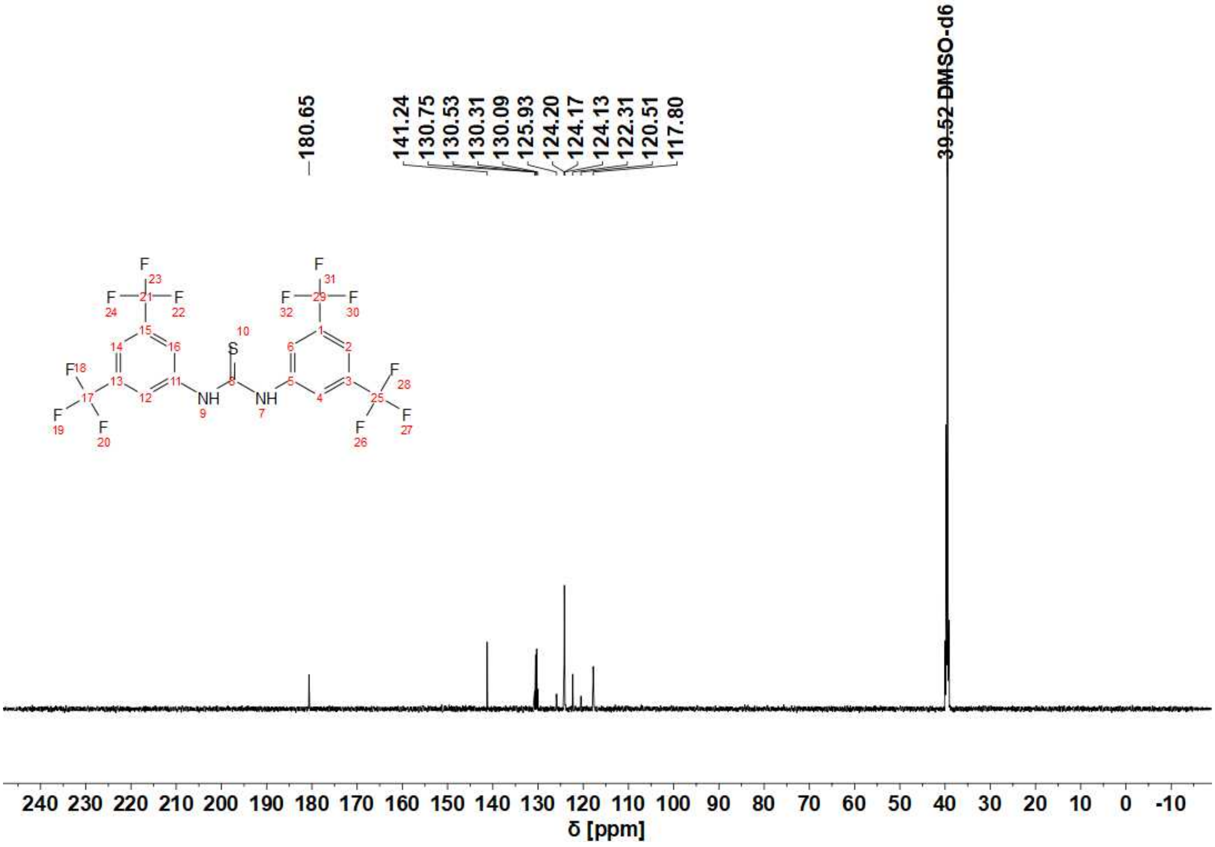

Figure S2: $^{13}C\{^{1}H\}$ NMR spectrum (DMSO-d_6, 151 MHz) of Schreiner's thiourea catalyst.

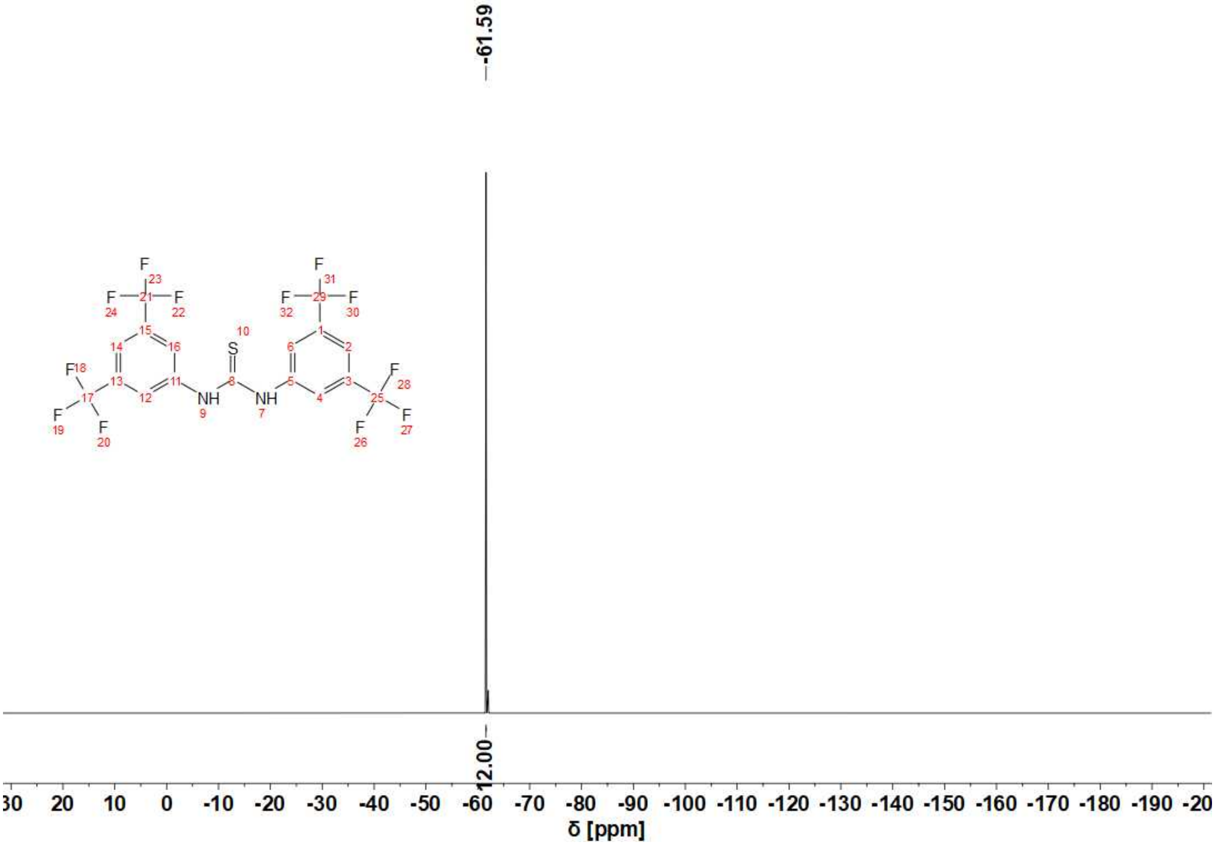

Figure S3: ^{19}F NMR spectrum (DMSO-d_6, 564 MHz) of Schreiner's thiourea catalyst.

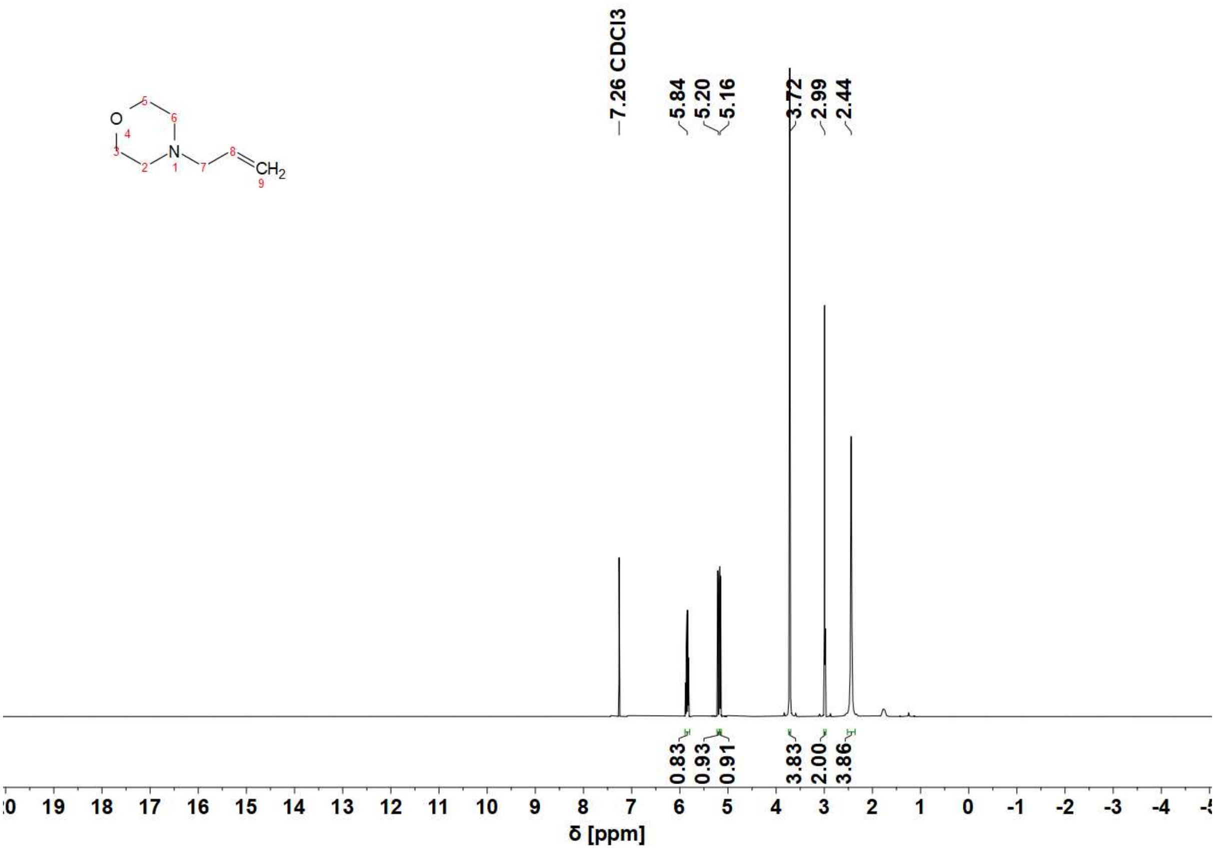

Figure S4: ^{1}H NMR spectrum ($CDCl_3$, 600 MHz) of *N*-allylmorpholine (**2a**).

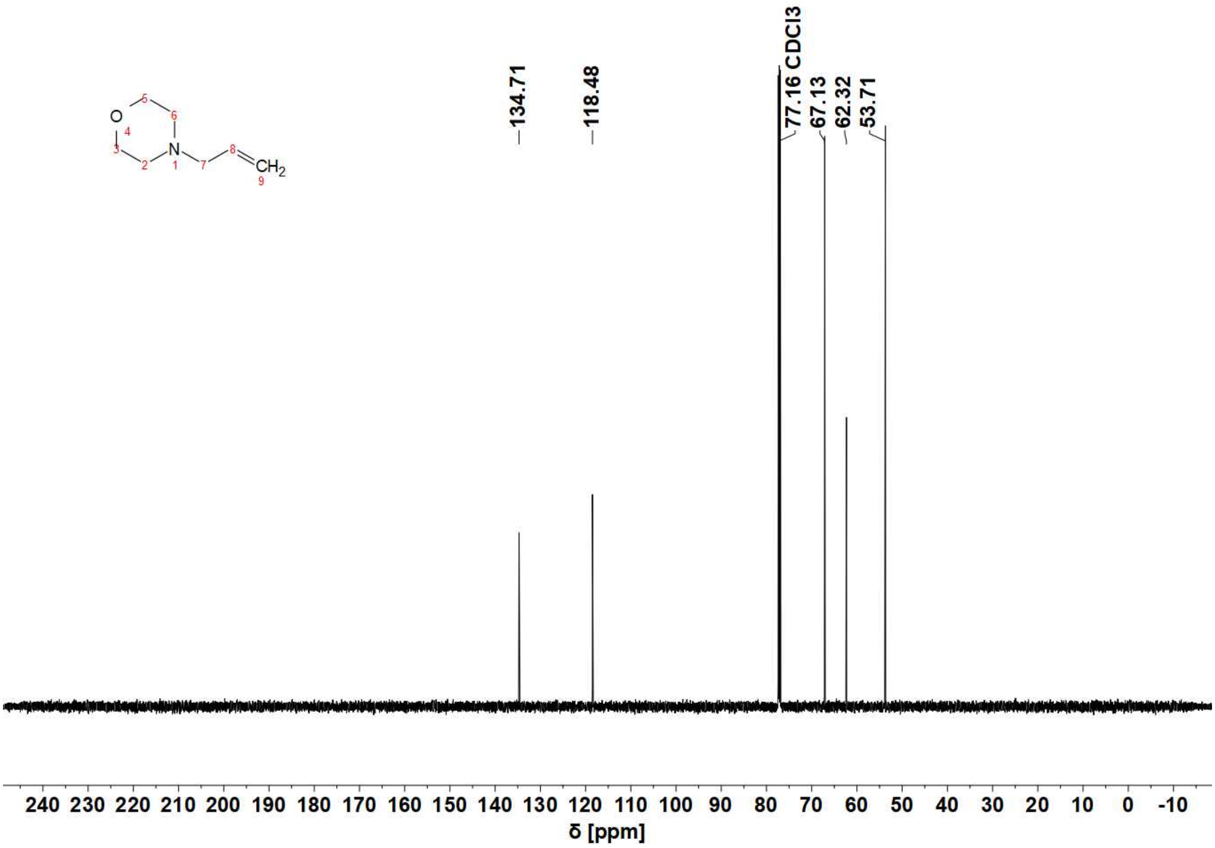

Figure S5: $^{13}C\{^{1}H\}$ NMR spectrum ($CDCl_3$, 151 MHz) of *N*-allylmorpholine (**2a**).

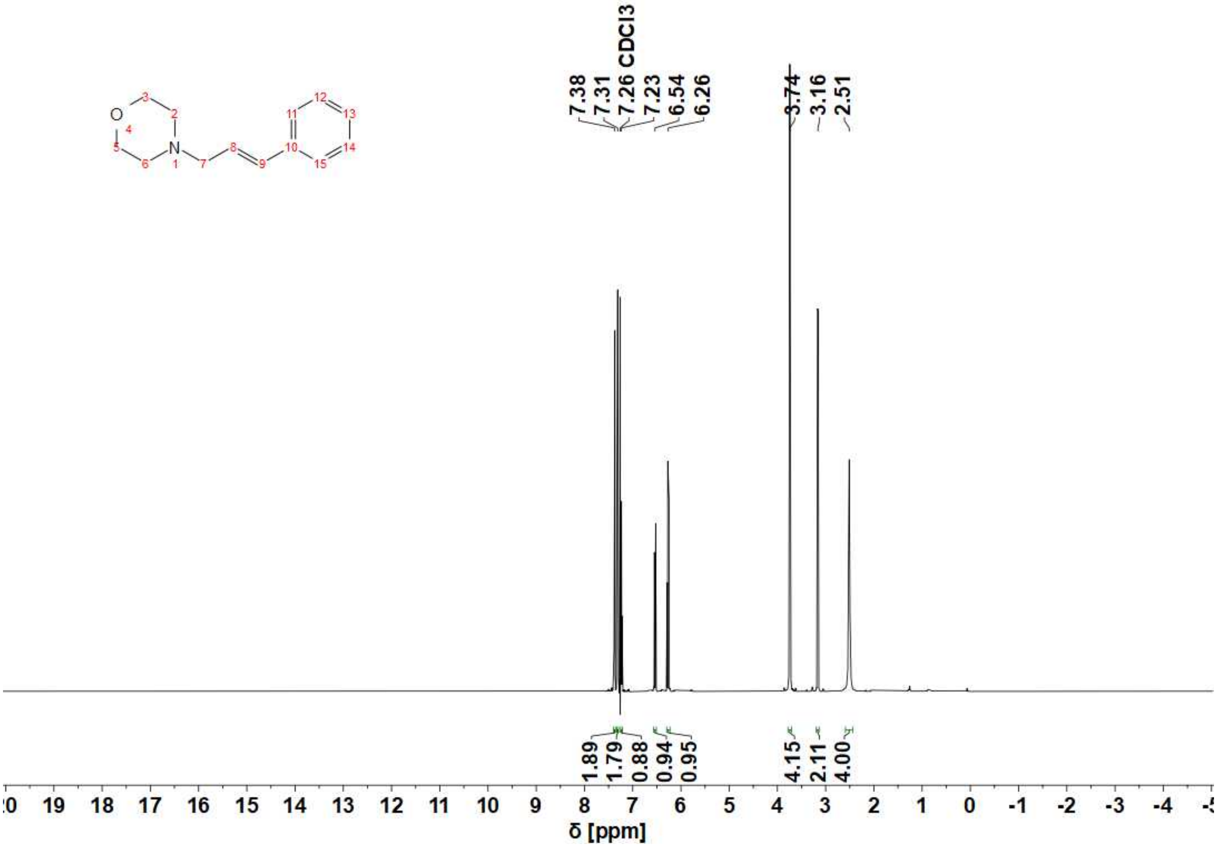

Figure S6: ^{1}H NMR spectrum ($CDCl_3$, 600 MHz) of *N*-(cinnamyl)morpholine (**2b**).

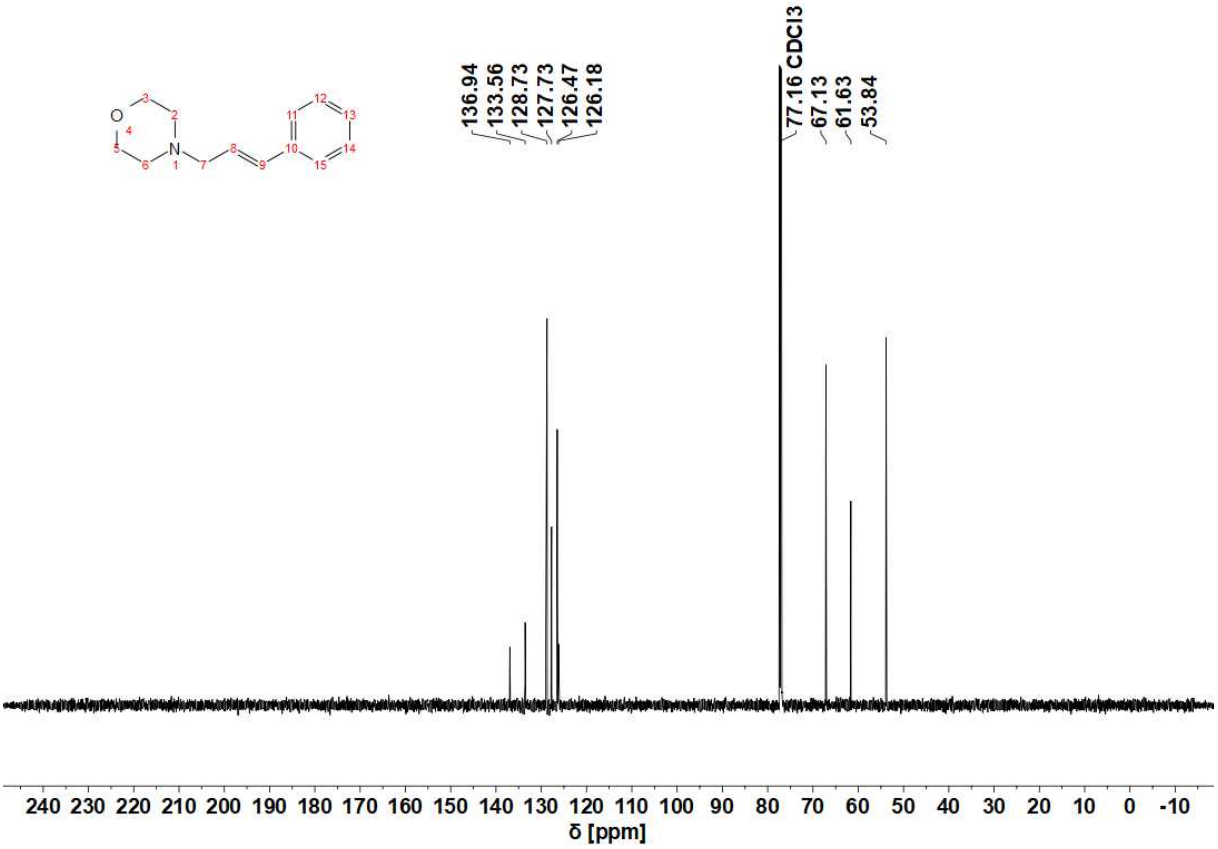

Figure S7: $^{13}C\{^{1}H\}$ NMR spectrum ($CDCl_3$, 151 MHz) of *N*-(cinnamyl)morpholine (**2b**).

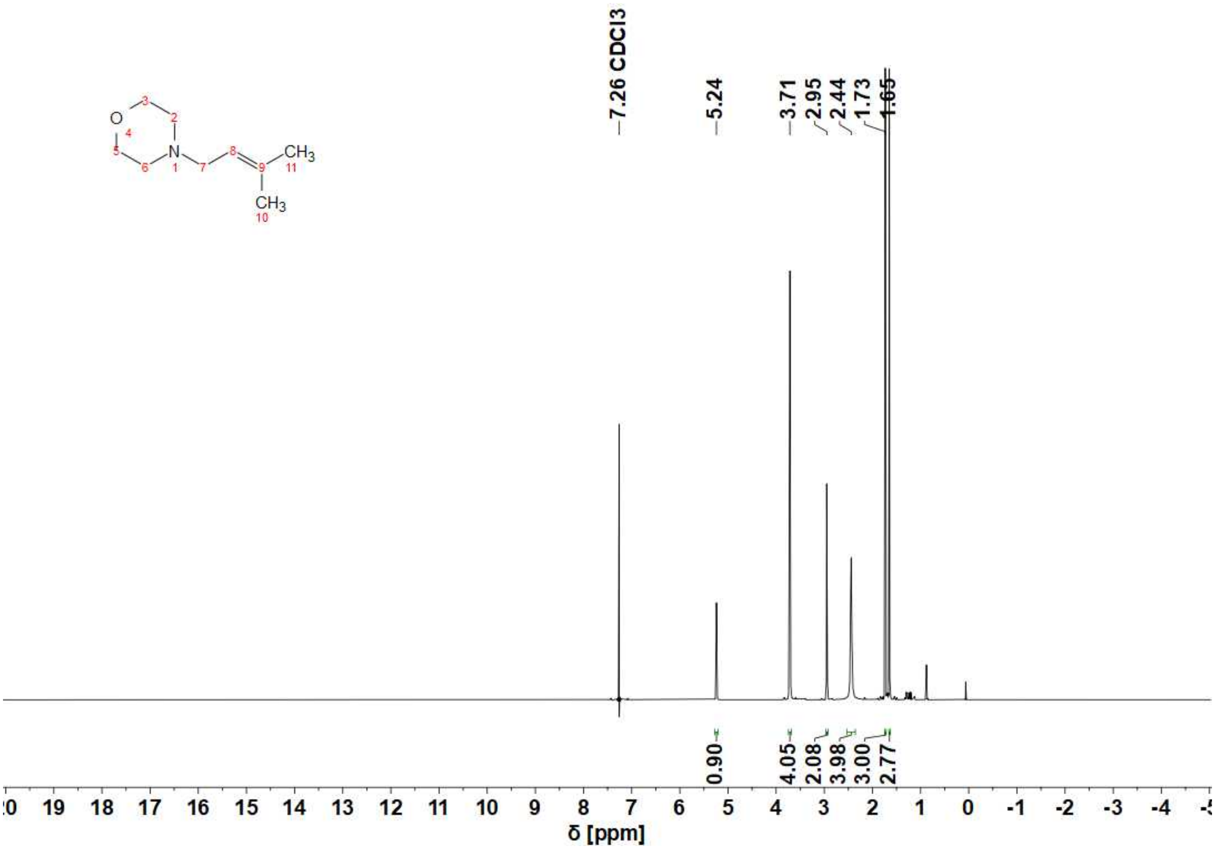

Figure S8: ^{1}H NMR spectrum ($CDCl_3$, 600 MHz) of *N*-prenylmorpholine (**2c**).

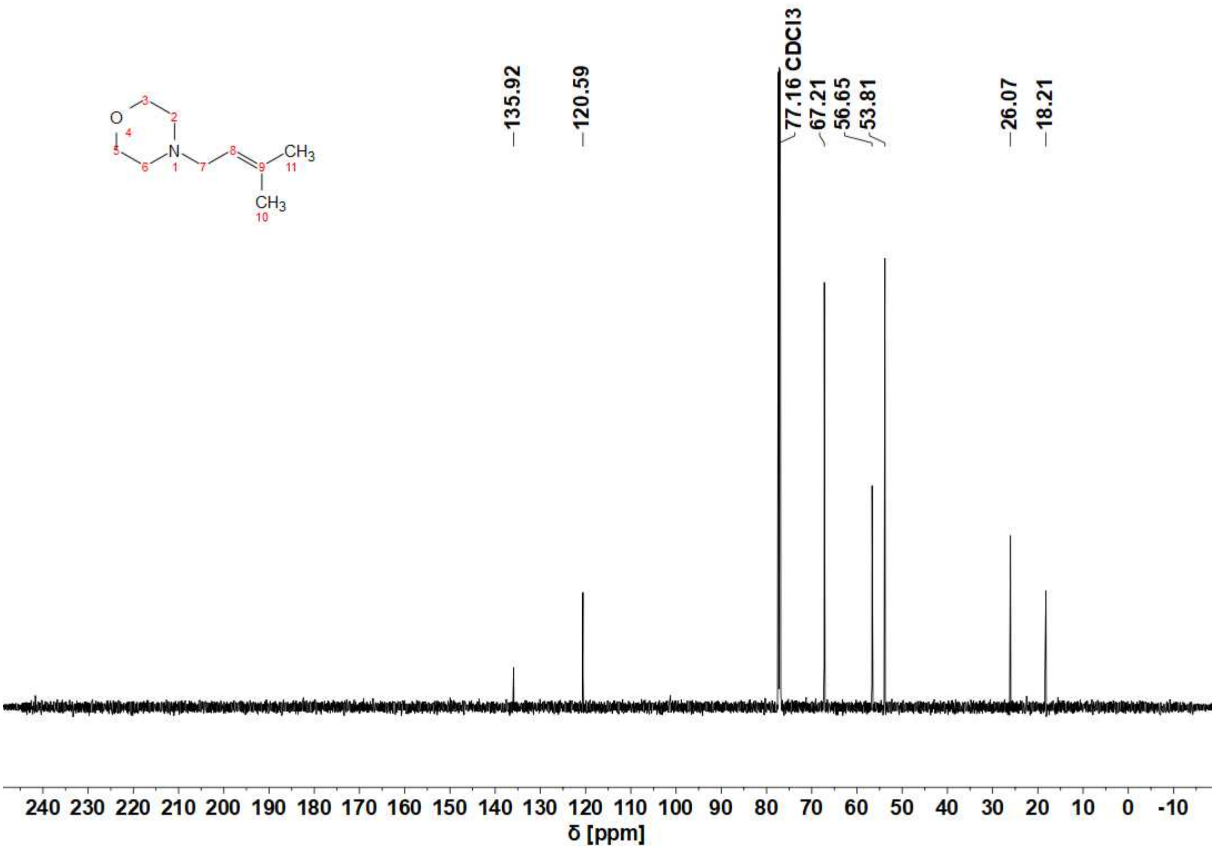

Figure S9: $^{13}C\{^{1}H\}$ NMR spectrum ($CDCl_3$, 151 MHz) of *N*-prenylmorpholine (**2c**).

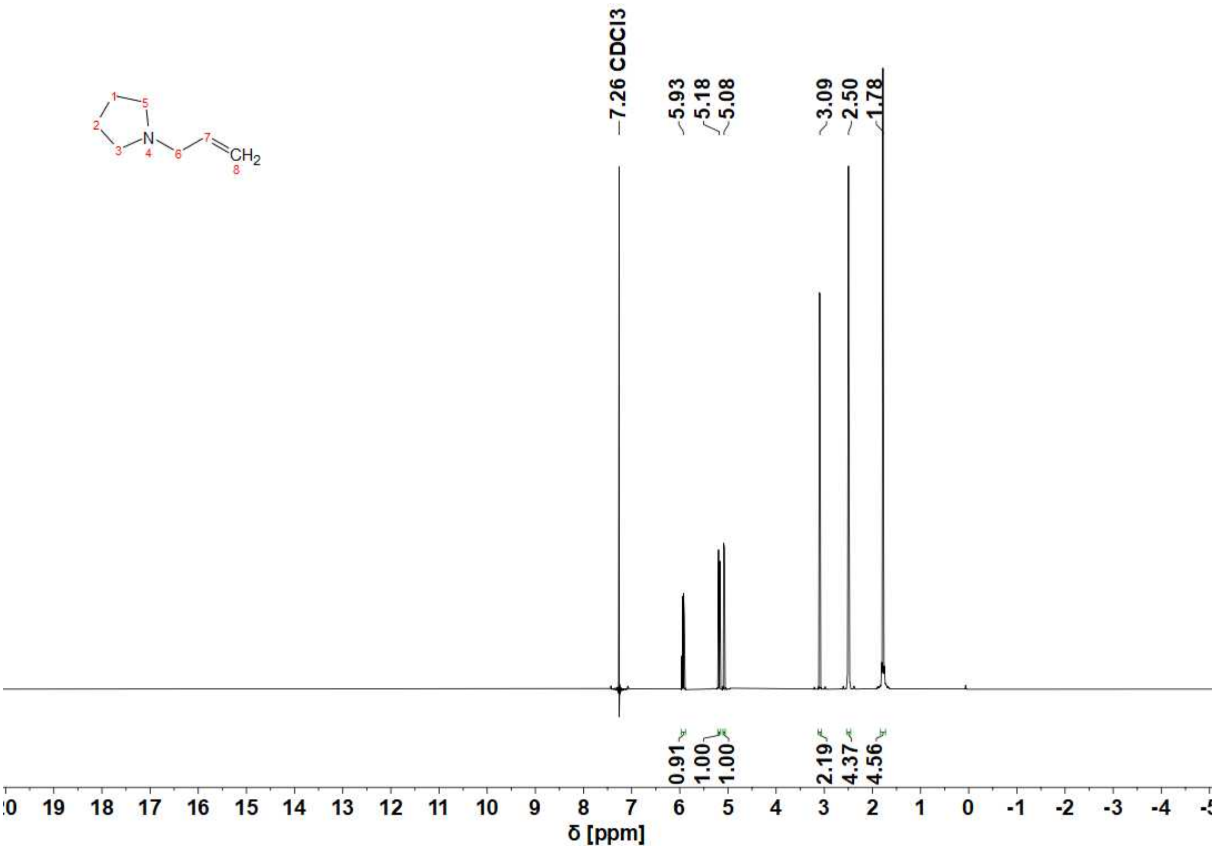

Figure S10: ^{1}H NMR spectrum ($CDCl_3$, 600 MHz) of *N*-allylpyrrolidine (**2d**).

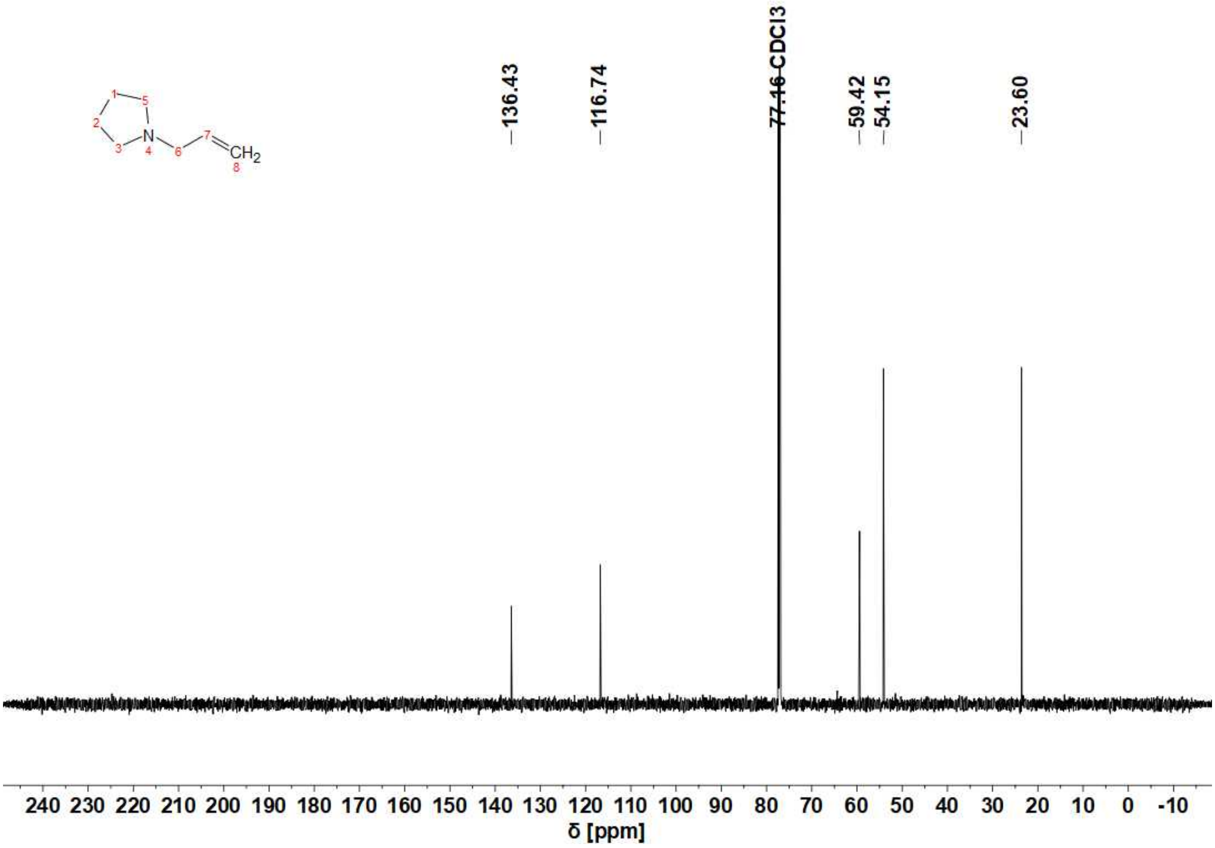

Figure S11: $^{13}C\{^{1}H\}$ NMR spectrum ($CDCl_3$, 151 MHz) of *N*-allylpyrrolidine (**2d**).

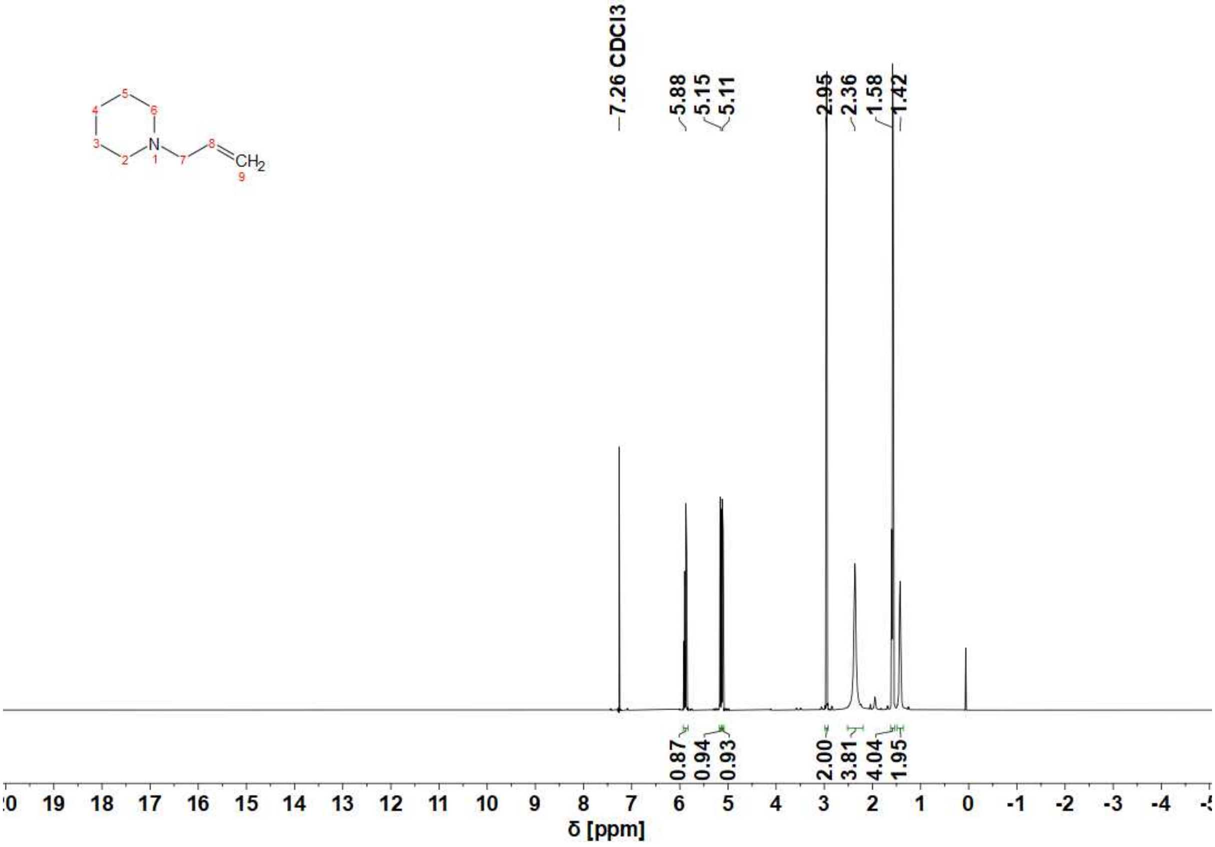

Figure S12: ^{1}H NMR spectrum ($CDCl_3$, 600 MHz) of *N*-allylpiperidine (**2e**).

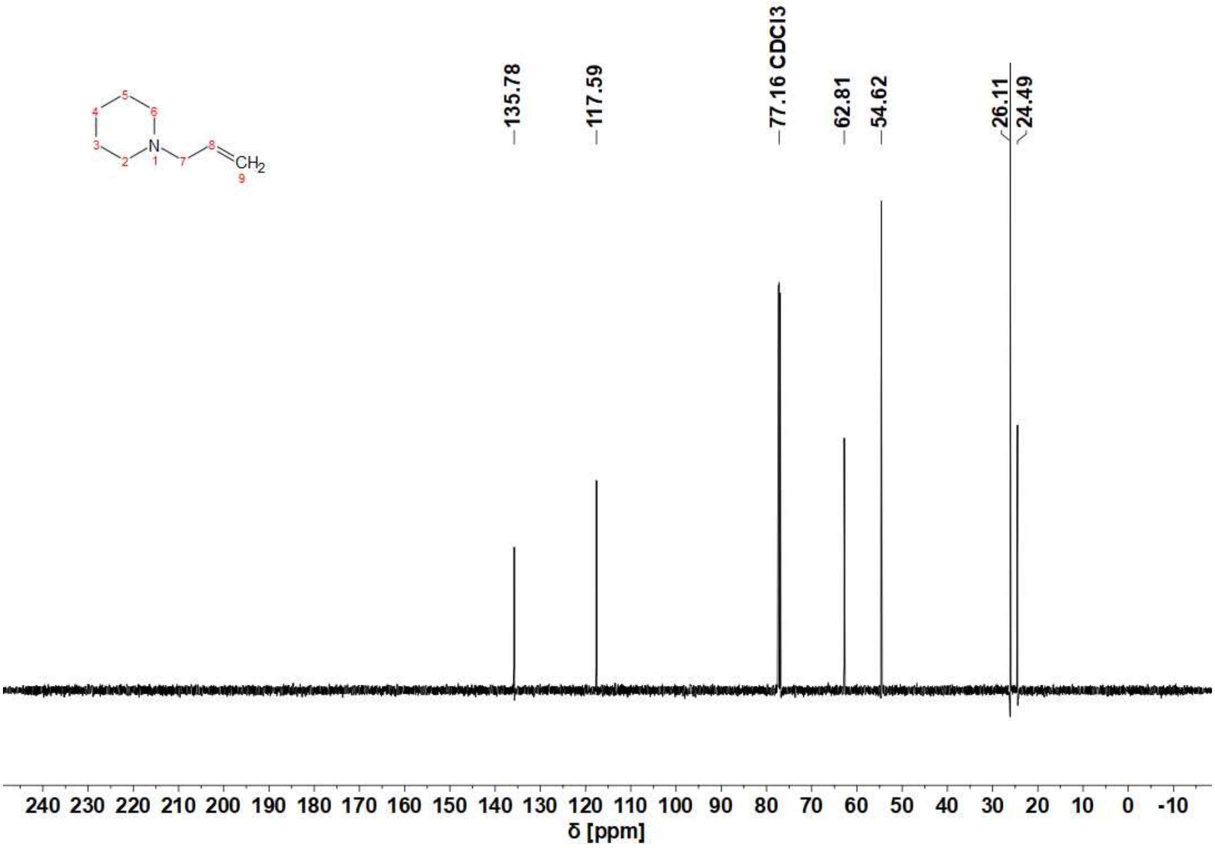

Figure S13: $^{13}C\{^1H\}$ NMR spectrum ($CDCl_3$, 151 MHz) of *N*-allylpiperidine (**2e**).

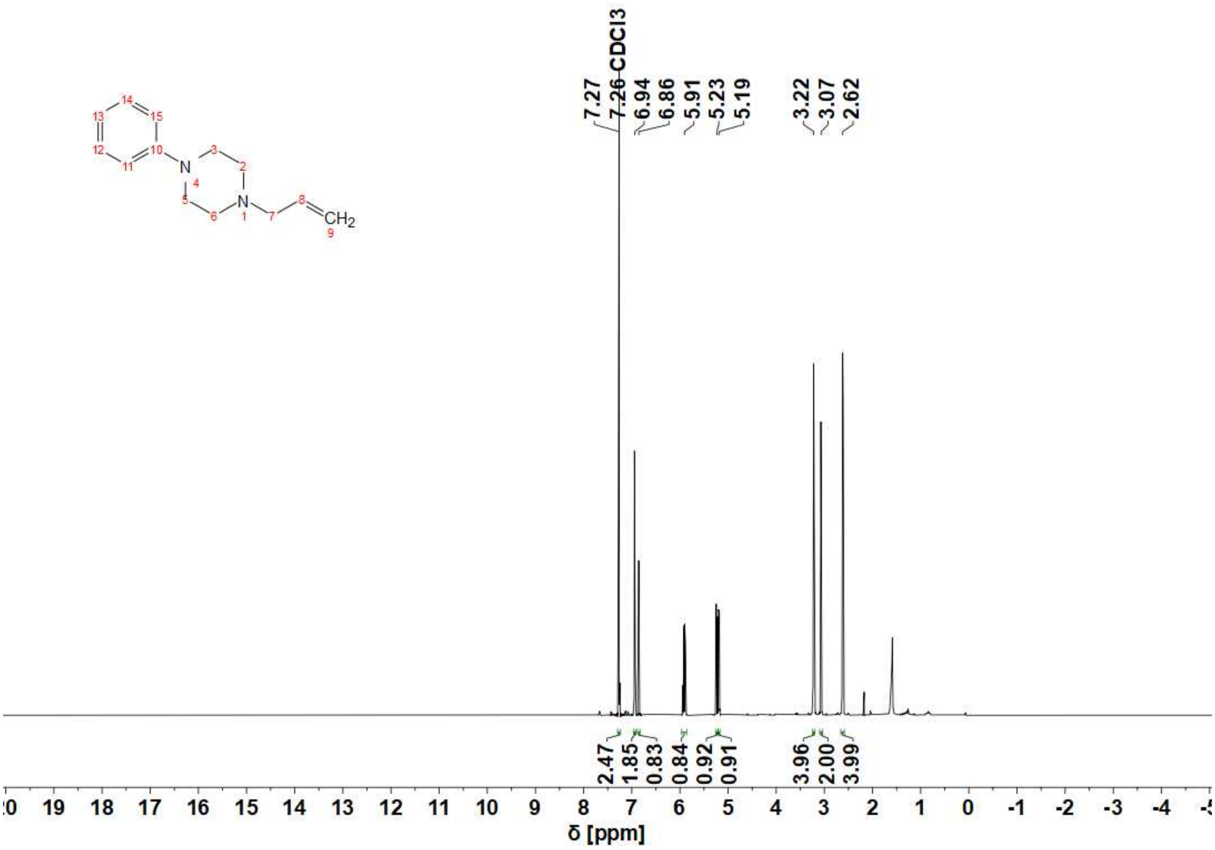

Figure S14: ^{1}H NMR spectrum ($CDCl_3$, 600 MHz) of 1-allyl-4-phenylpiperazine (**2f**).

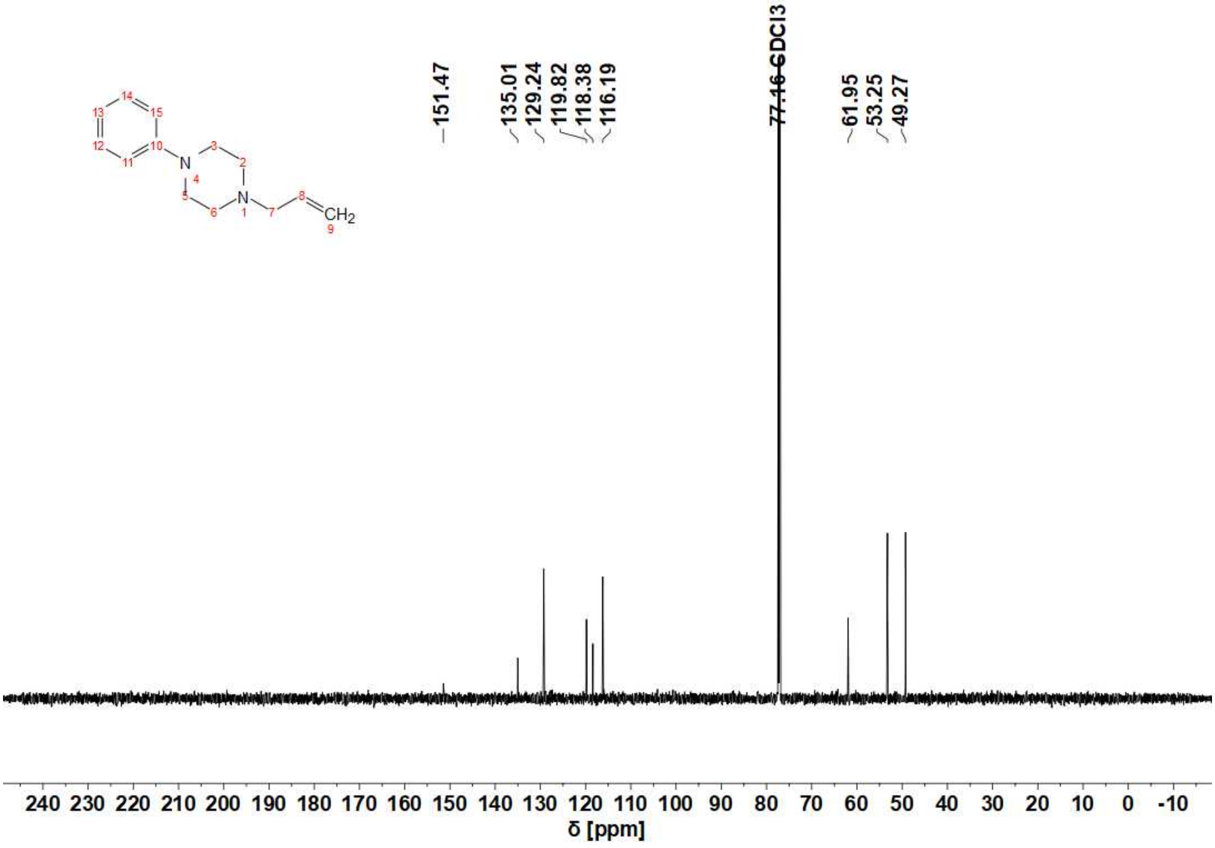

Figure S15: $^{13}C\{^{1}H\}$ NMR spectrum ($CDCl_3$, 151 MHz) of 1-allyl-4-phenylpiperazine (**2f**).

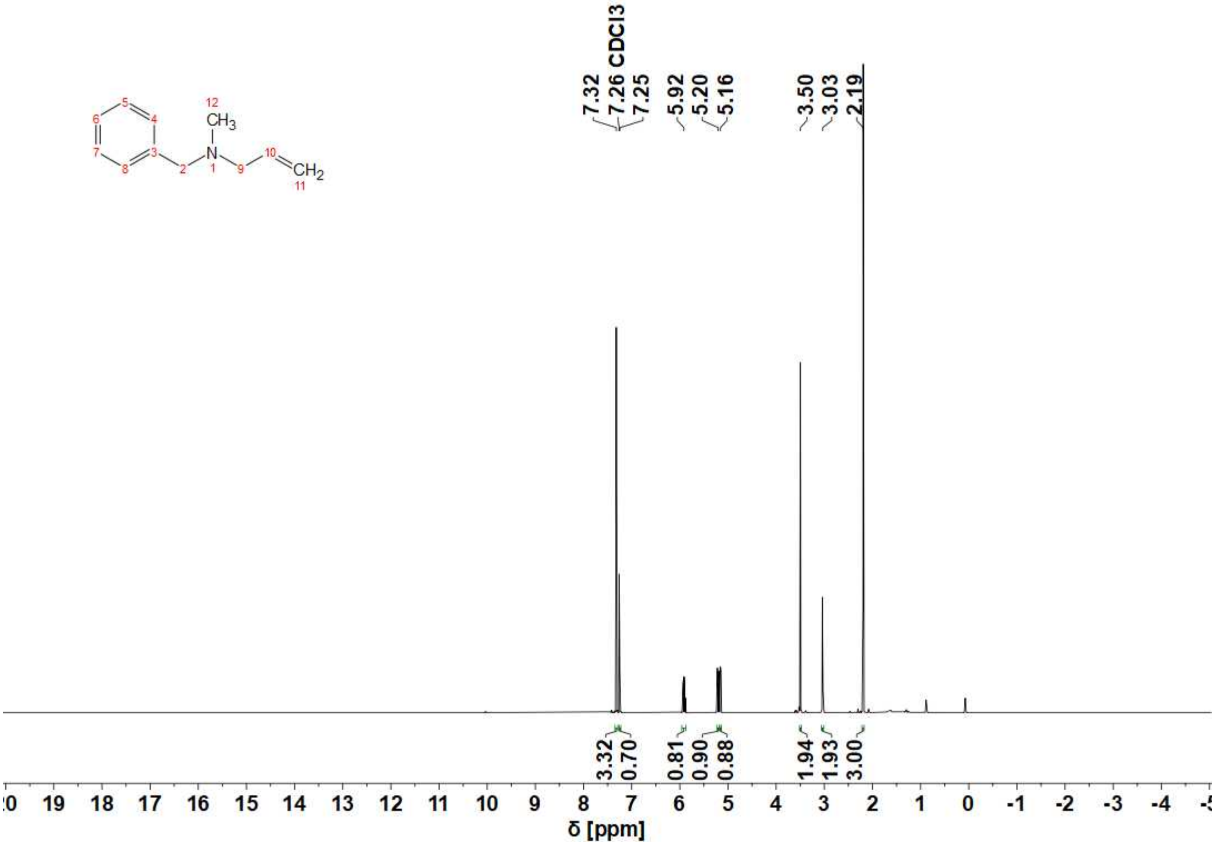

Figure S16: ^{1}H NMR spectrum ($CDCl_3$, 600 MHz) of *N*-benzyl-*N*-methylprop-2-en-1-amine (**2g**).

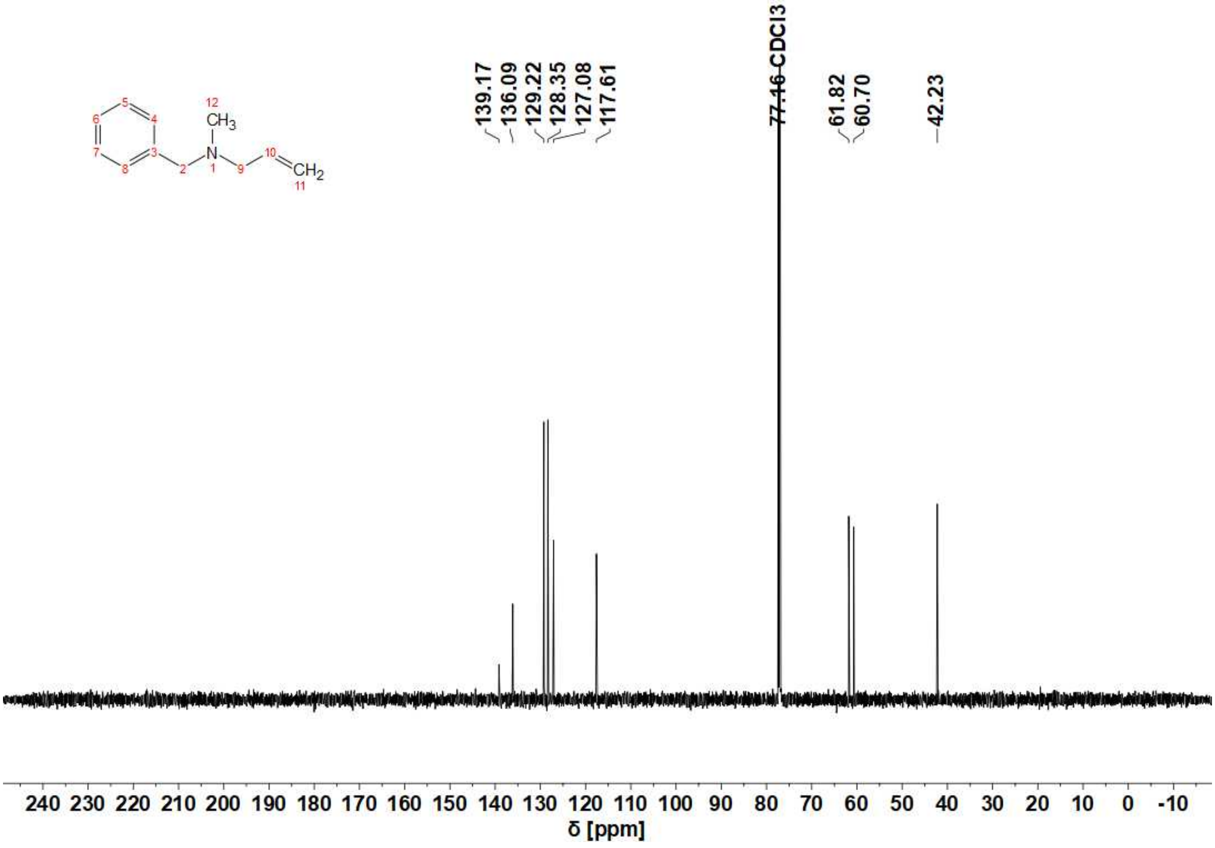

Figure S17: $^{13}C\{^{1}H\}$ NMR spectrum ($CDCl_3$, 151 MHz) of *N*-benzyl-*N*-methylprop-2-en-1-amine (**2g**).

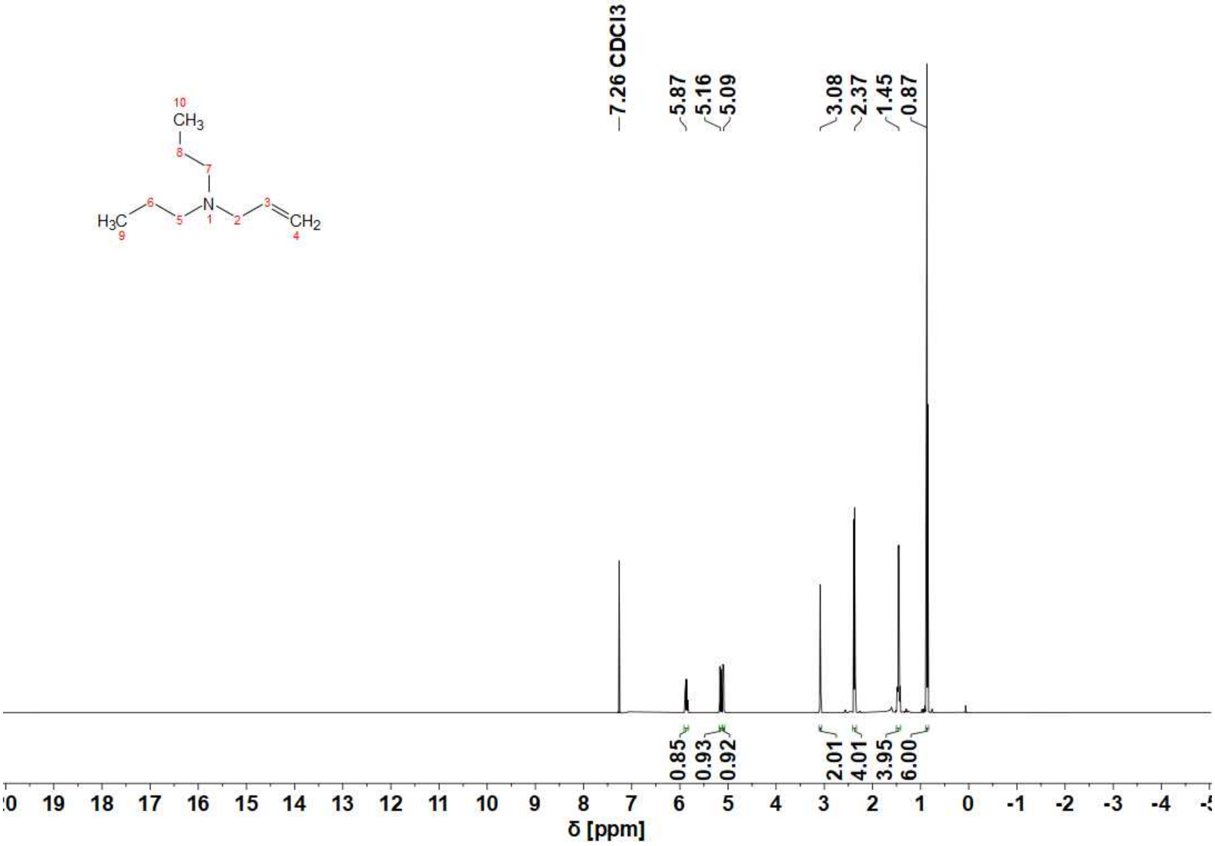

Figure S18: ^{1}H NMR spectrum ($CDCl_3$, 600 MHz) of *N,N*-dipropylprop-2-en-1-amine (**2h**).

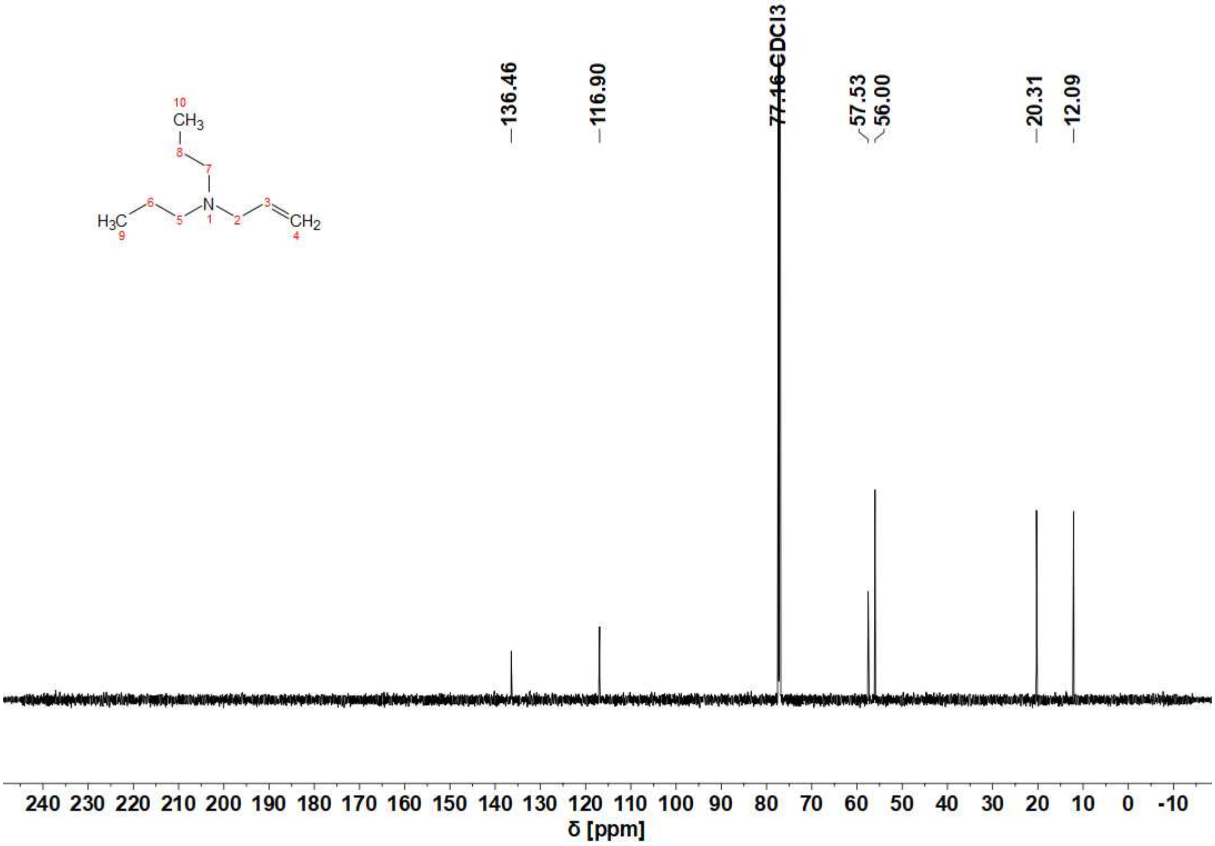

Figure S19: $^{13}C\{^{1}H\}$ NMR spectrum ($CDCl_3$, 151 MHz) of *N,N*-dipropylprop-2-en-1-amine (**2h**).

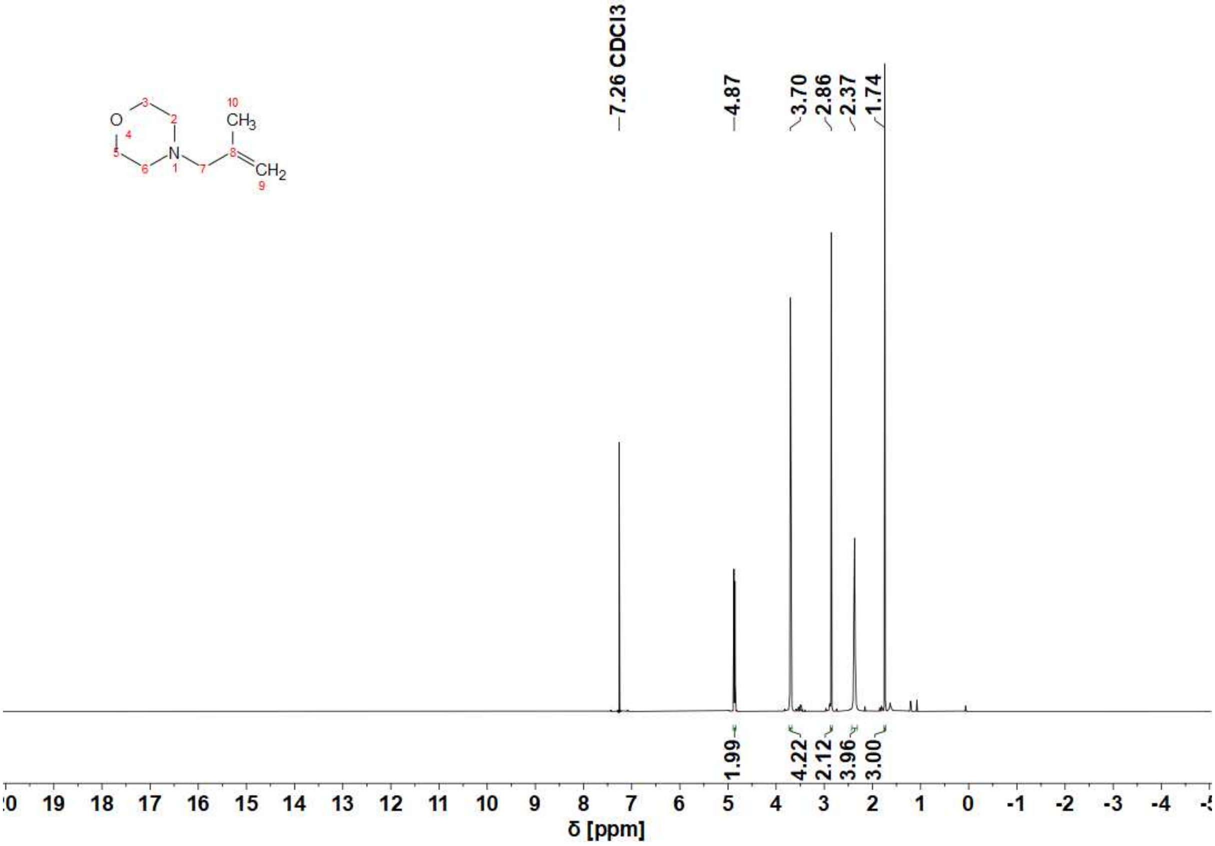

Figure S20: ^{1}H NMR spectrum ($CDCl_3$, 600 MHz) of *N*-(2-methylallyl)morpholine (**2i**).

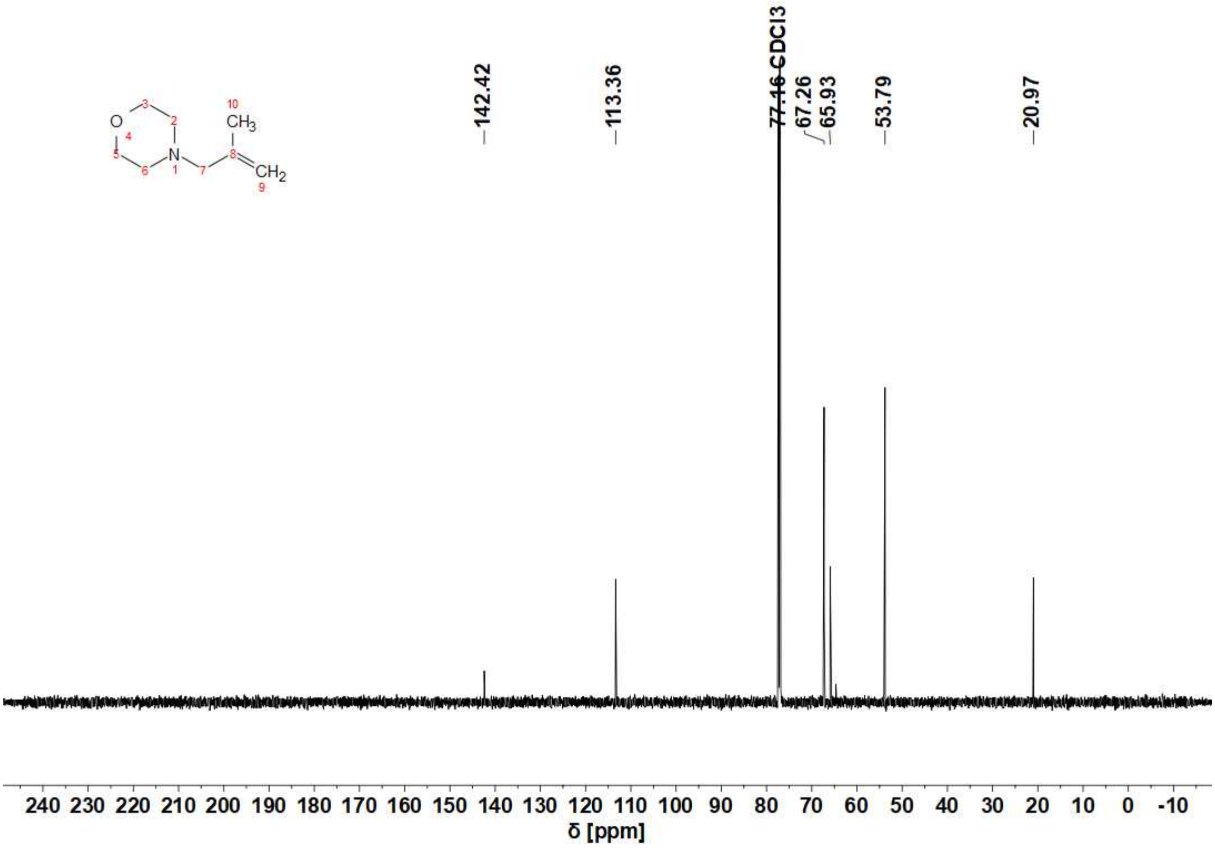

Figure S21: $^{13}C\{^{1}H\}$ NMR spectrum ($CDCl_3$, 151 MHz) of *N*-(2-methylallyl)morpholine (**2i**).

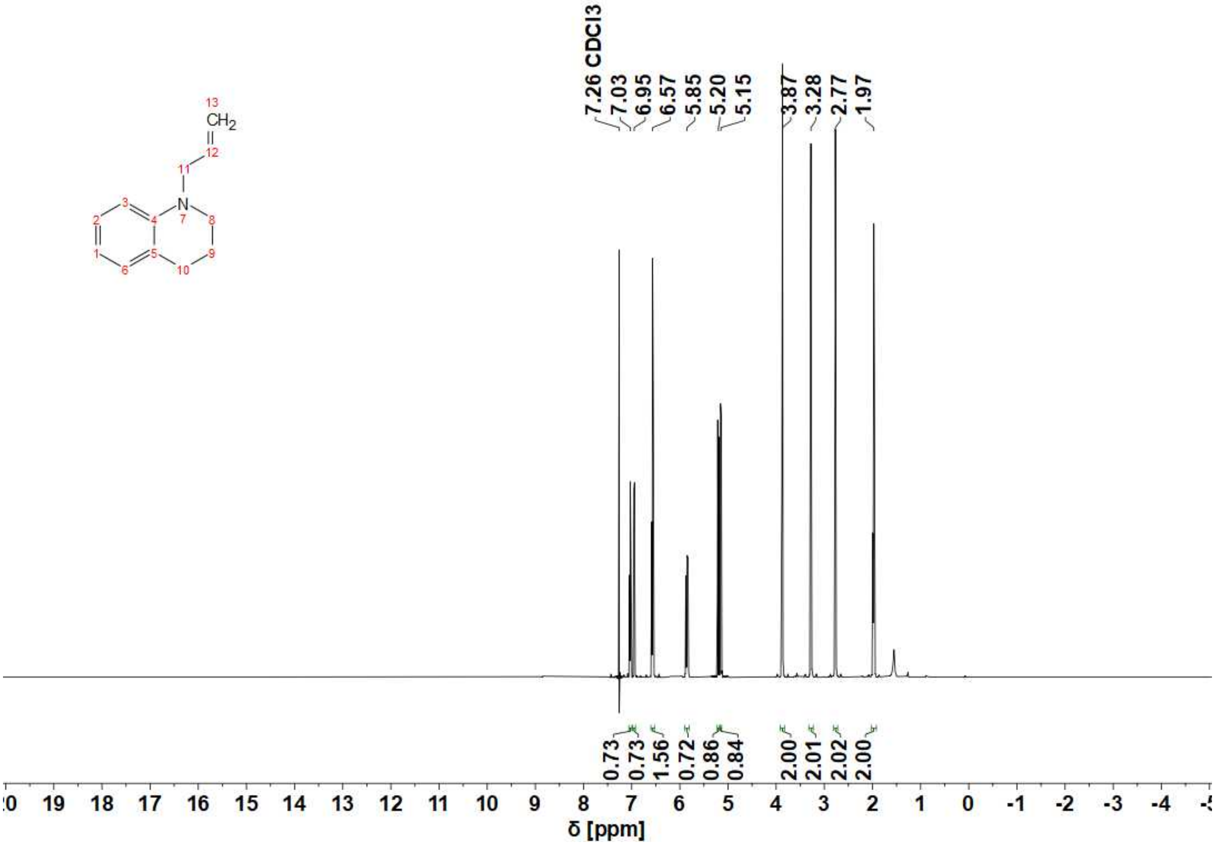

Figure S22: ^{1}H NMR spectrum ($CDCl_3$, 600 MHz) of *N*-allyl-1,2,3,4-tetrahydroquinoline (**2j**).

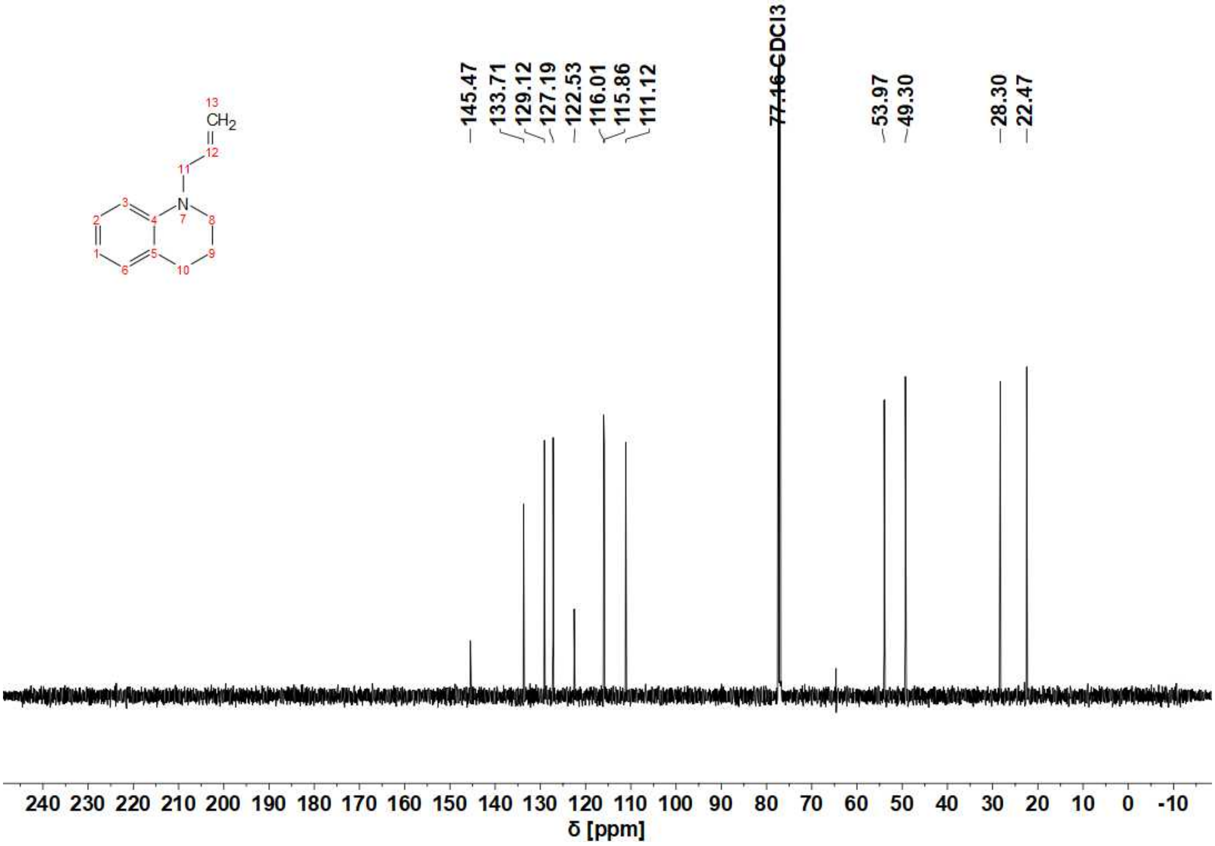

Figure S23: $^{13}C\{^{1}H\}$ NMR spectrum ($CDCl_3$, 151 MHz) of *N*-allyl-1,2,3,4-tetrahydroquinoline (**2j**).

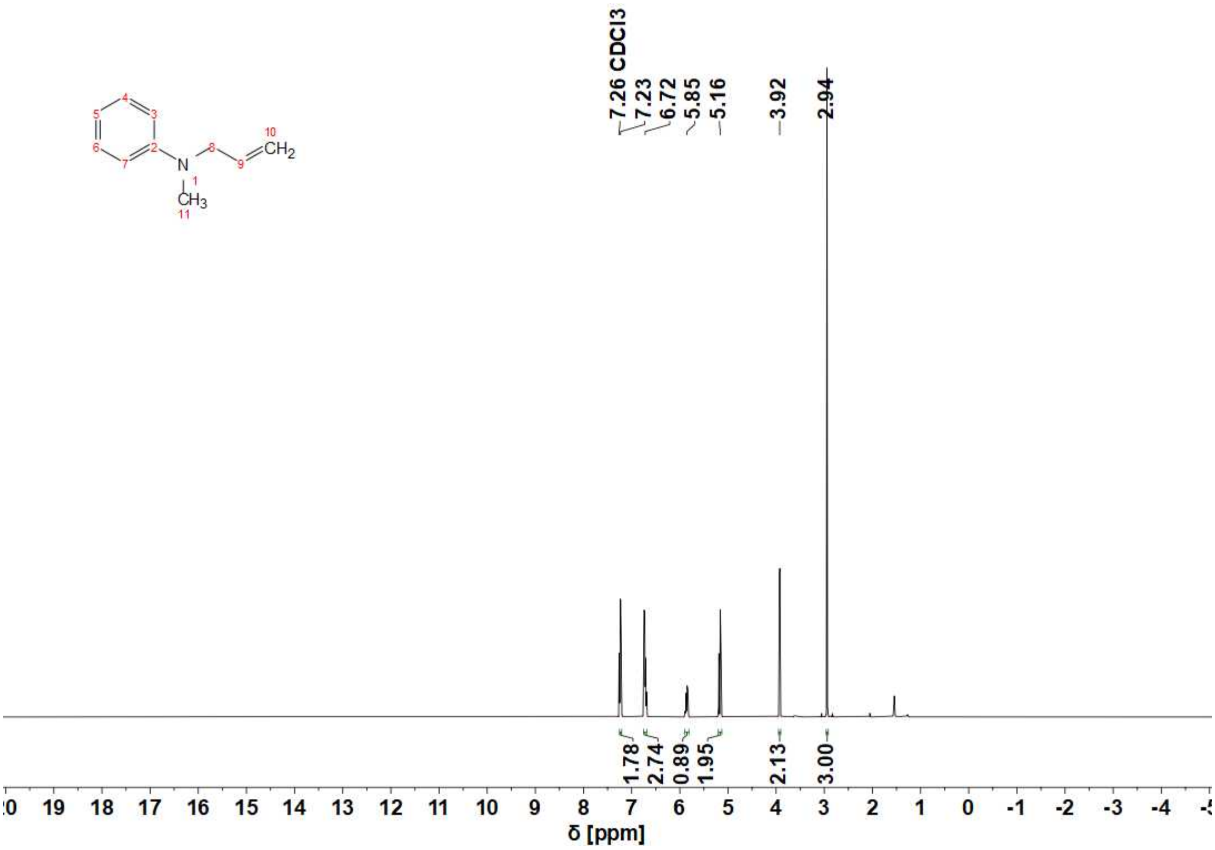

Figure S24: ^{1}H NMR spectrum ($CDCl_3$, 600 MHz) of *N*-allyl-*N*-methylaniline (**2k**).

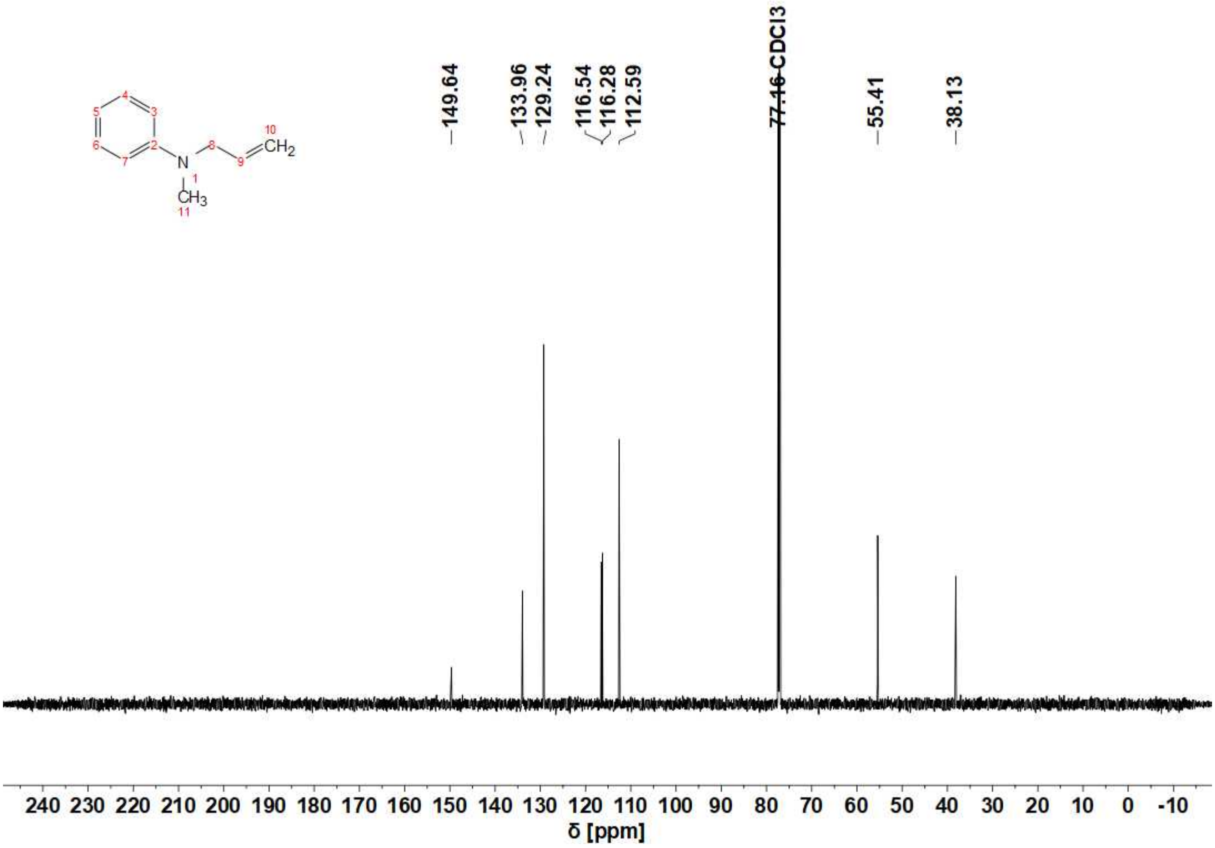

Figure S25: $^{13}C\{^{1}H\}$ NMR spectrum ($CDCl_3$, 151 MHz) of *N*-allyl-*N*-methylaniline (**2k**).

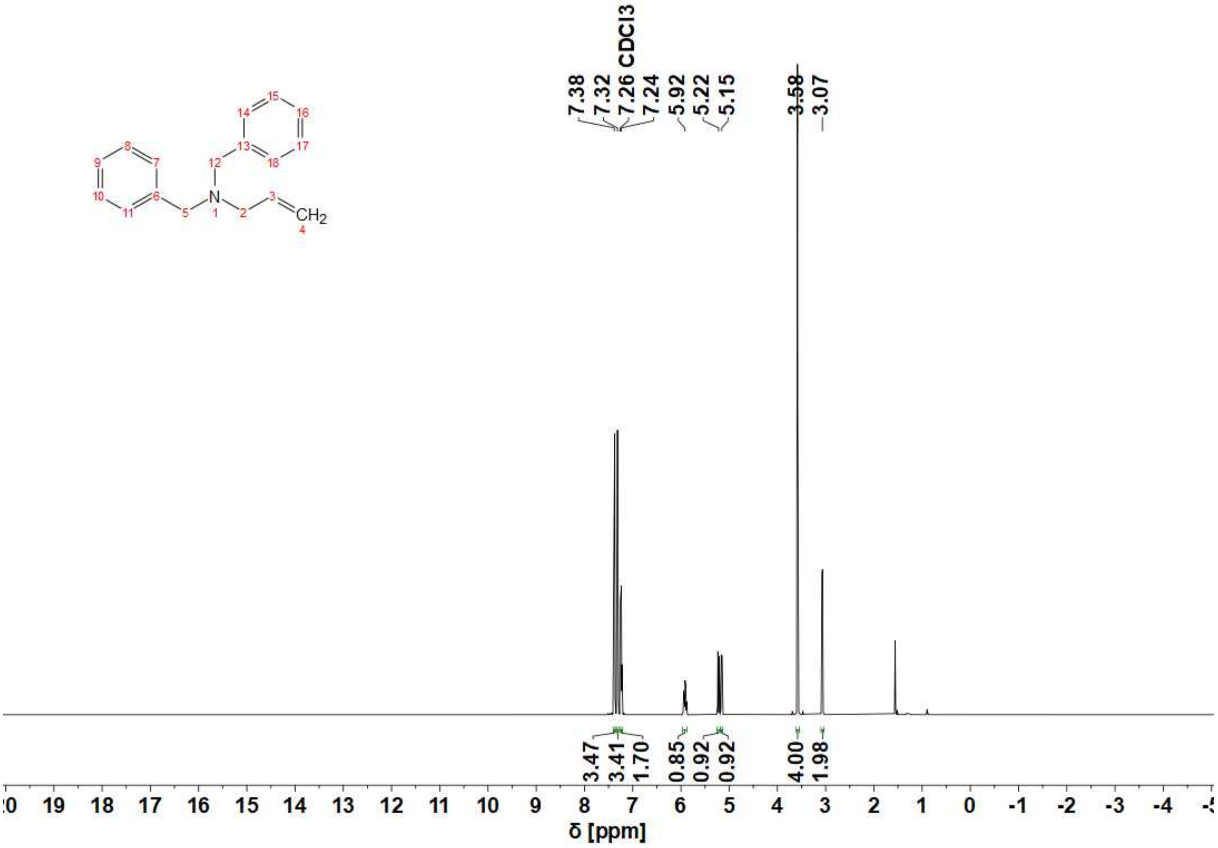

Figure S26: ^{1}H NMR spectrum ($CDCl_3$, 600 MHz) of *N*,*N*-dibenzylprop-2-en-1-amine (**2l**).

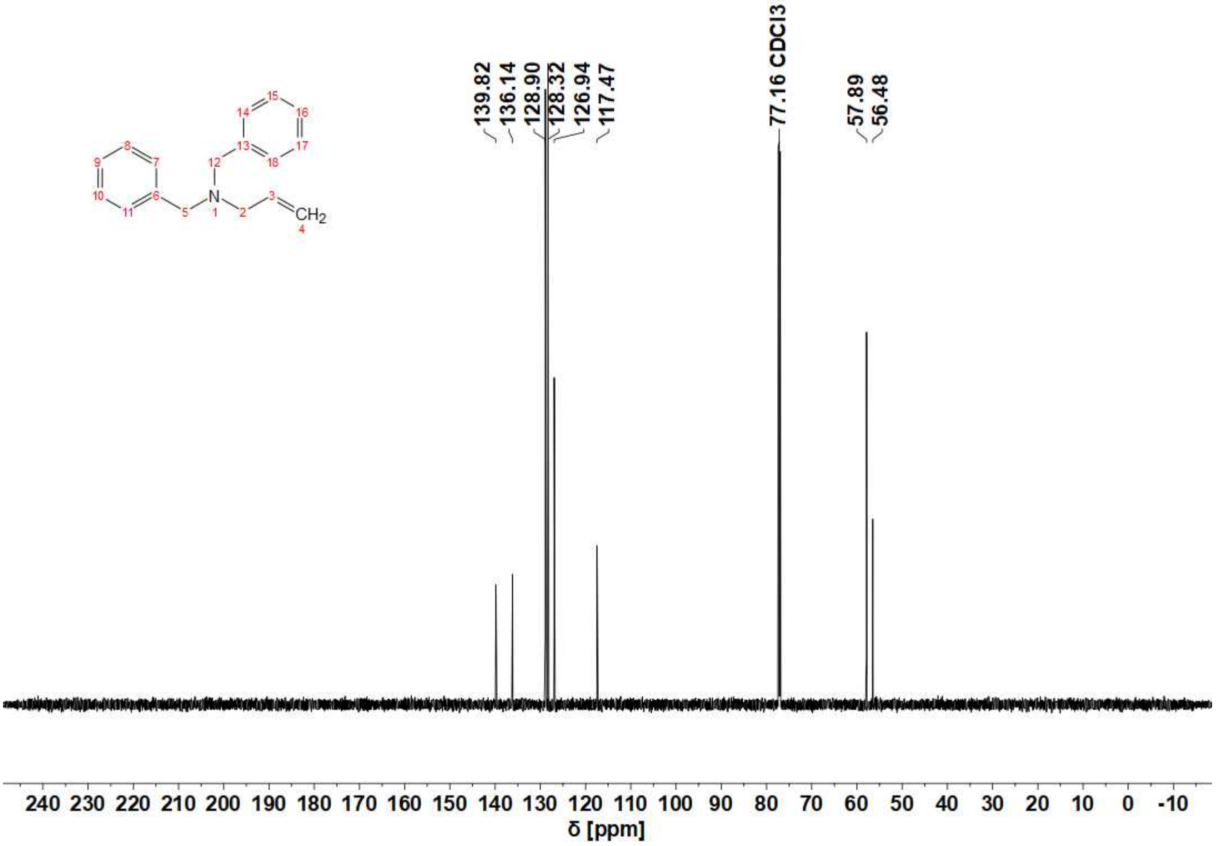

Figure S27: $^{13}C\{^{1}H\}$ NMR spectrum ($CDCl_3$, 151 MHz) of *N*,*N*-dibenzylprop-2-en-1-amine (**2l**).

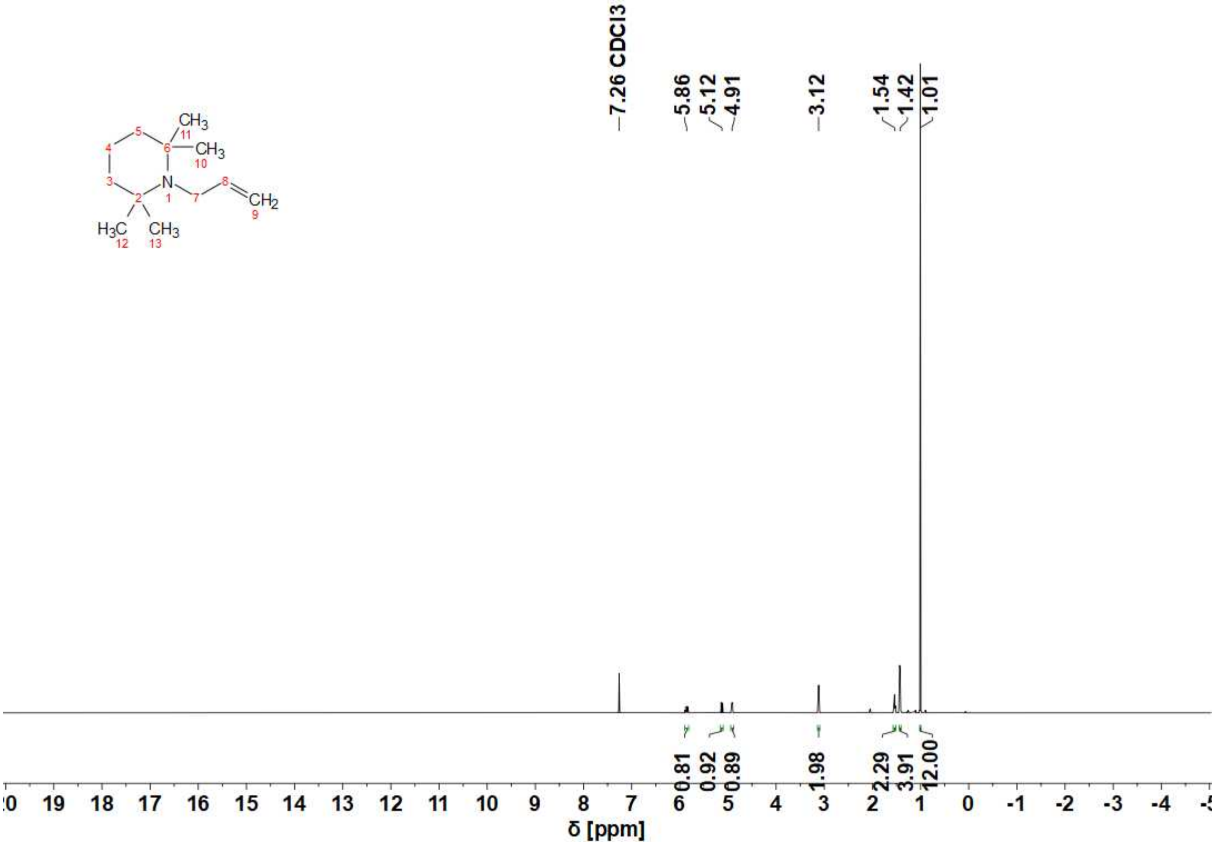

Figure S28: ^{1}H NMR spectrum ($CDCl_3$, 600 MHz) of 1-allyl-2,2,6,6-tetramethylpiperidine (**2m**).

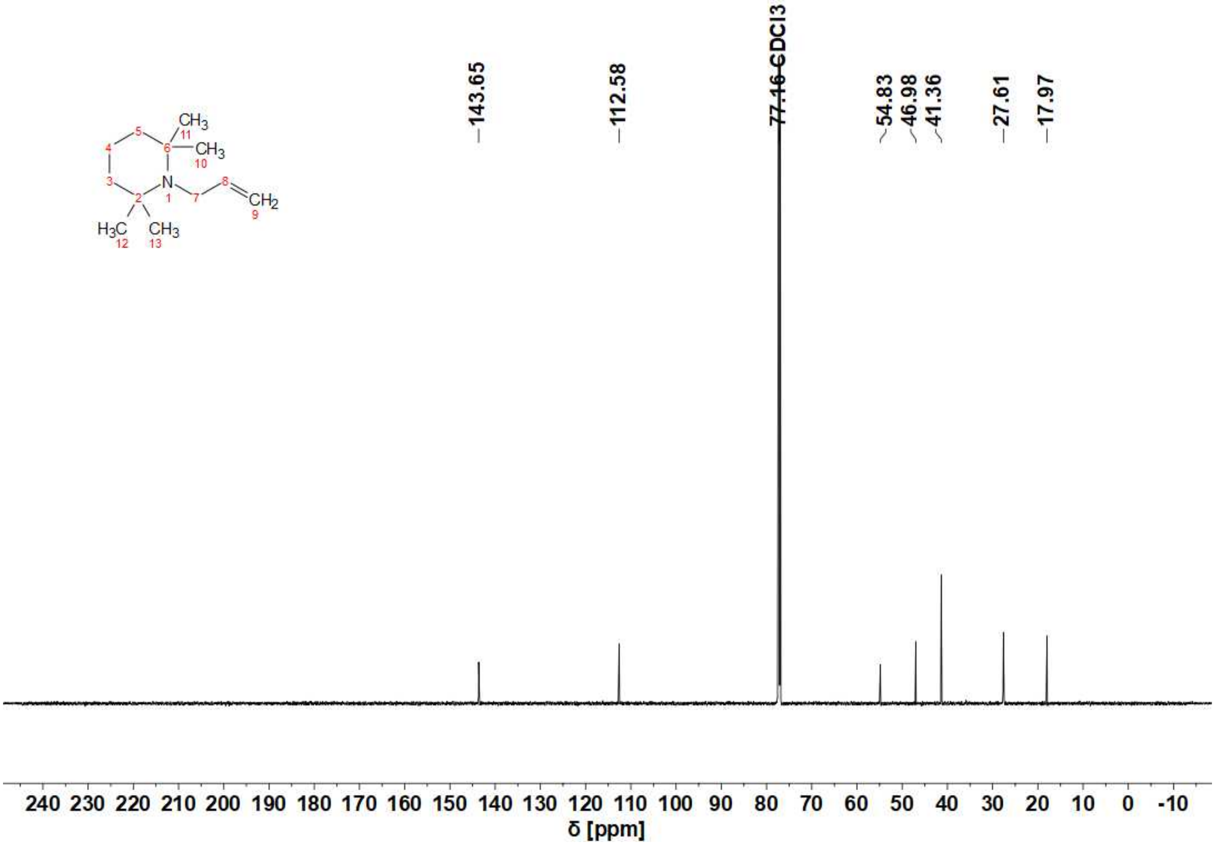

Figure S29: $^{13}C\{^{1}H\}$ NMR spectrum ($CDCl_3$, 151 MHz) of 1-allyl-2,2,6,6-tetramethylpiperidine (**2m**).

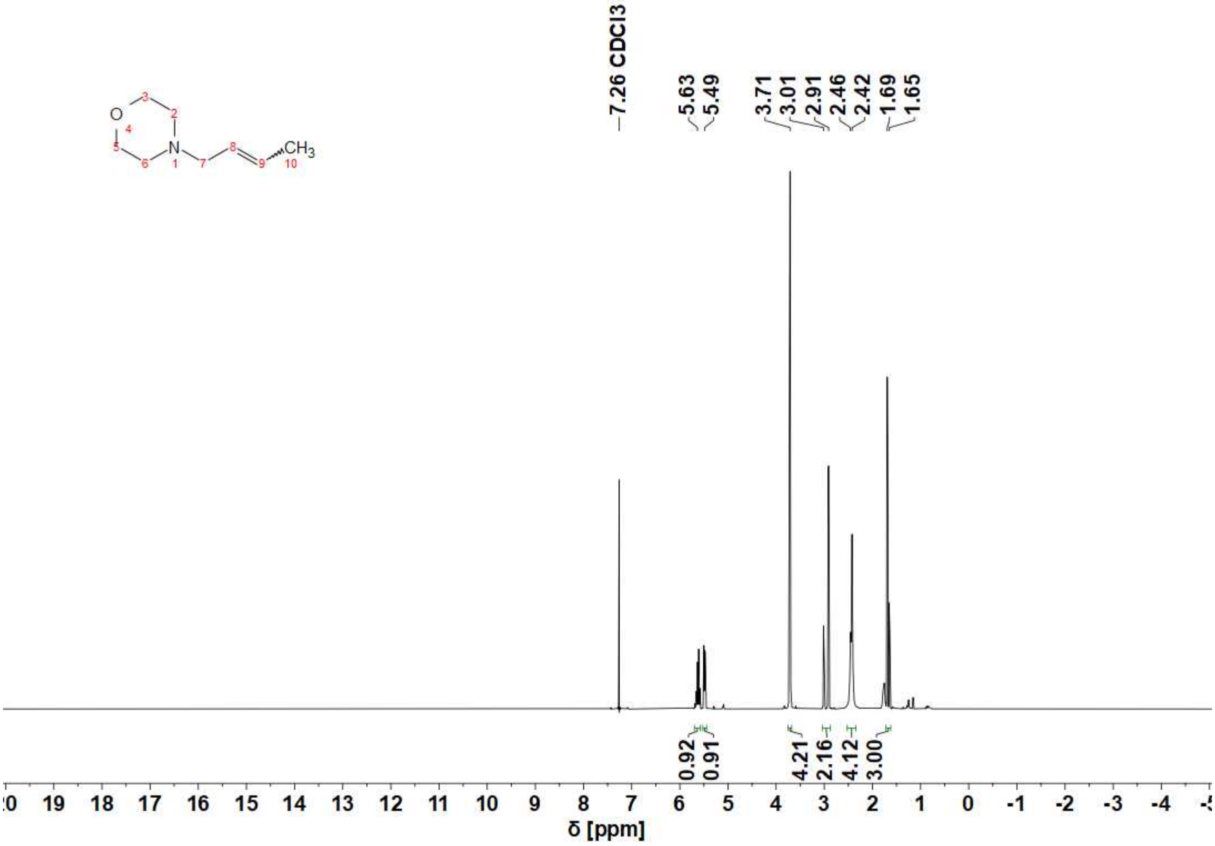

Figure S30: ^{1}H NMR spectrum ($CDCl_3$, 600 MHz) of *N*-crotylmorpholine (**2n**).

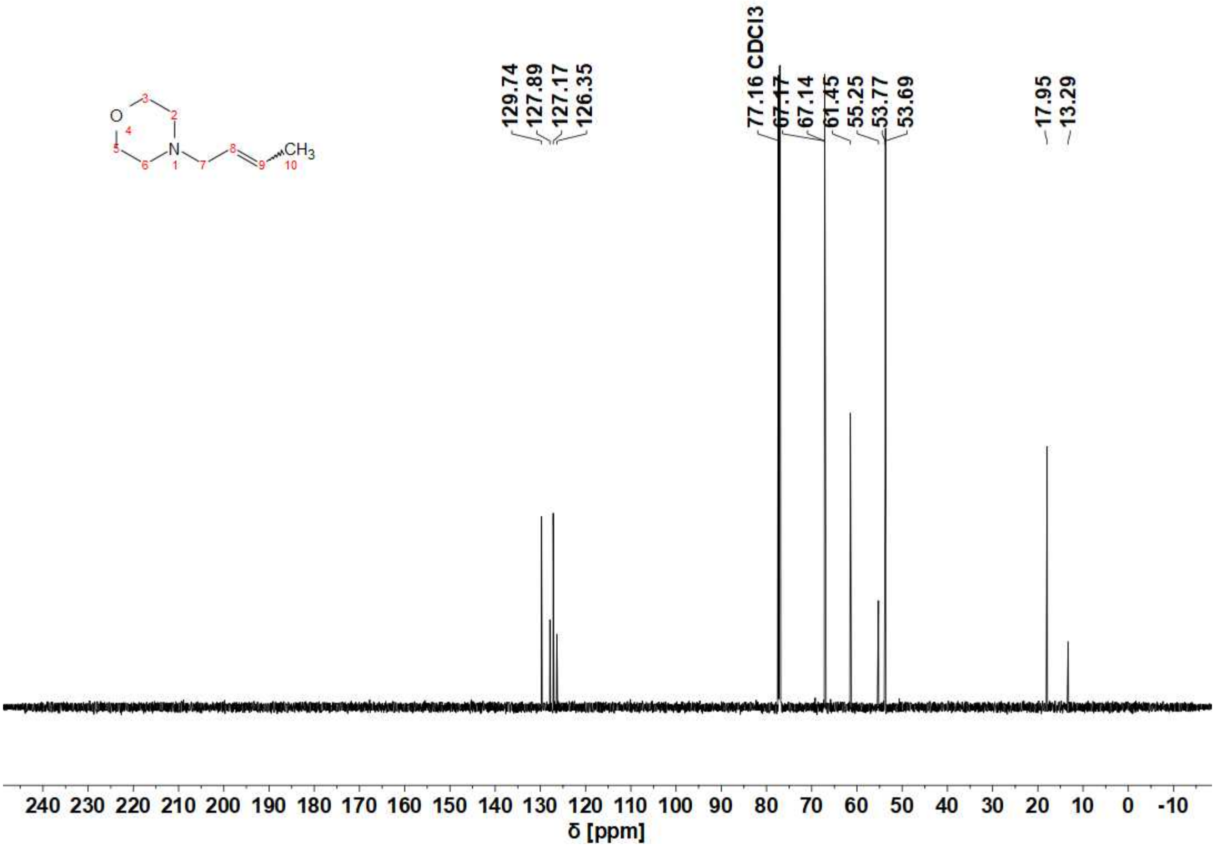

Figure S31: $^{13}C\{^{1}H\}$ NMR spectrum ($CDCl_3$, 151 MHz) of *N*-crotylmorpholine (**2n**).

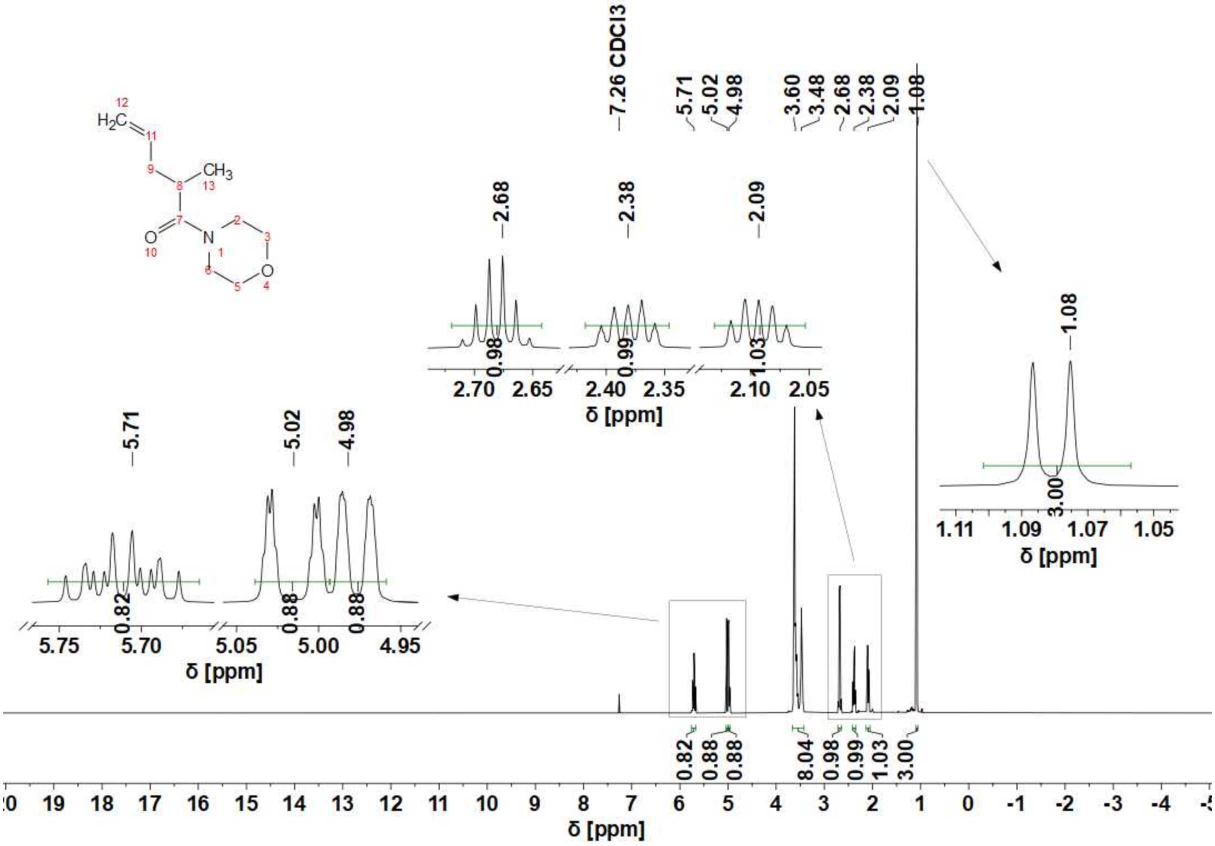

Figure S32: ^{1}H NMR spectrum ($CDCl_3$, 600 MHz) of 2-methyl-1-morpholinopent-4-en-1-one (**3aa**).

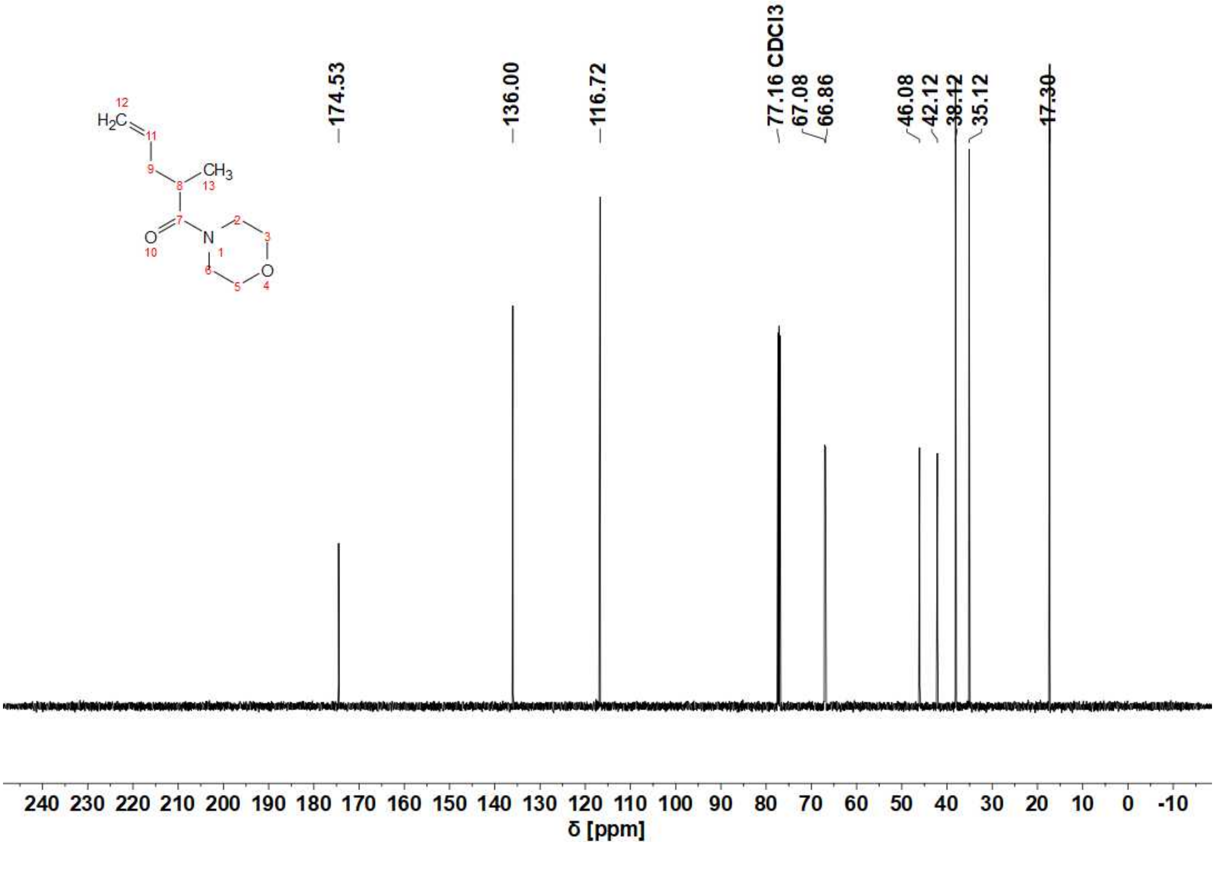

Figure S33: $^{13}C\{^{1}H\}$ NMR spectrum ($CDCl_3$, 151 MHz) of 2-methyl-1-morpholinopent-4-en-1-one (**3aa**).

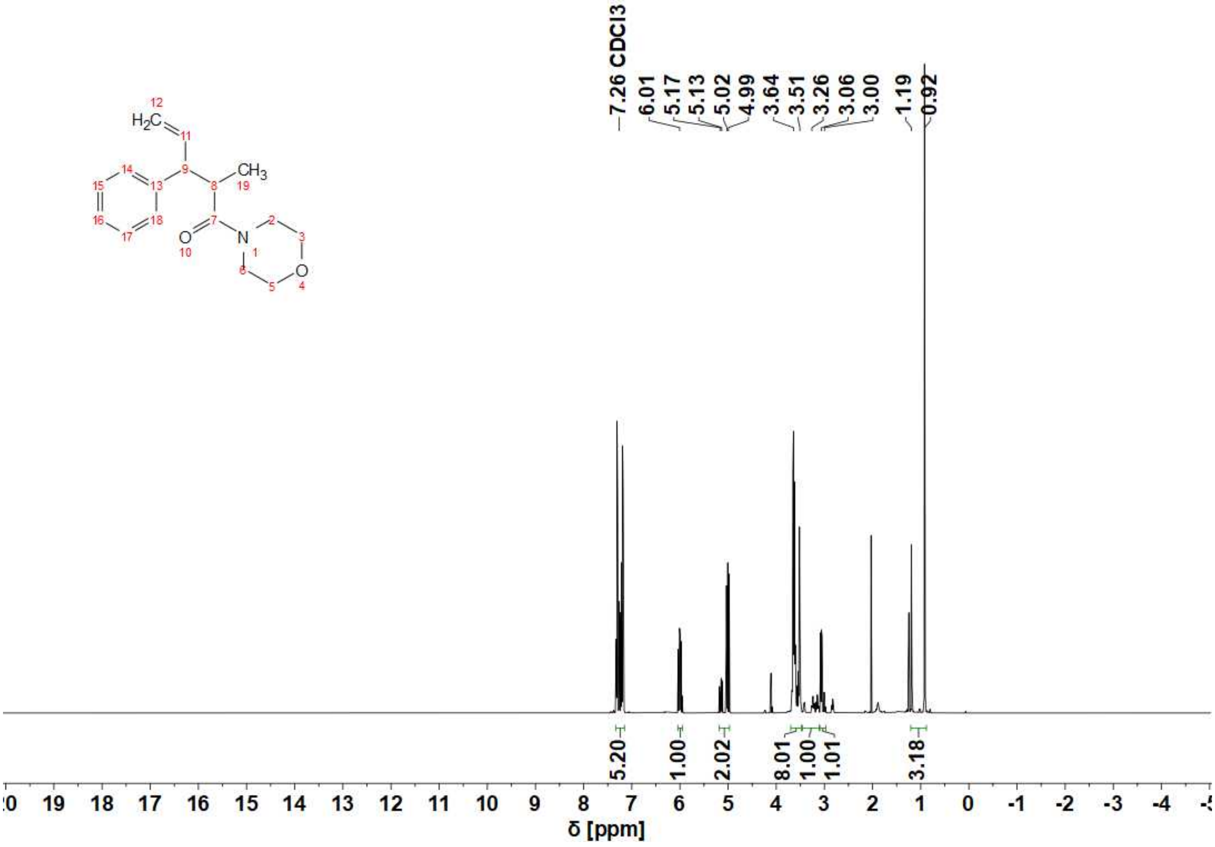

Figure S34: ^{1}H NMR spectrum ($CDCl_3$, 600 MHz) of 2-methyl-1-morpholino-3-phenylpent-4-en-1-one (**3ab**).

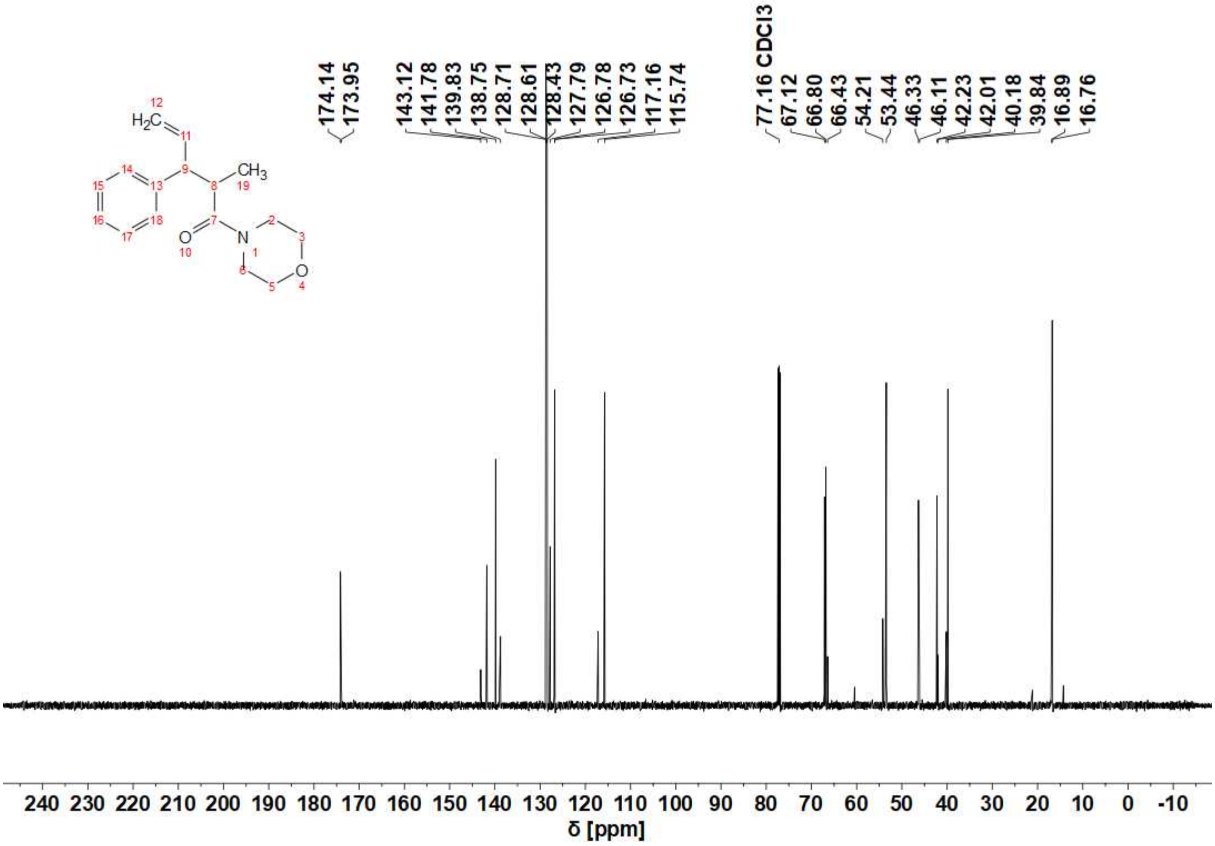

Figure S35: $^{13}C\{^{1}H\}$ NMR spectrum ($CDCl_3$, 151 MHz) of 2-methyl-1-morpholino-3-phenylpent-4-en-1-one (**3ab**).

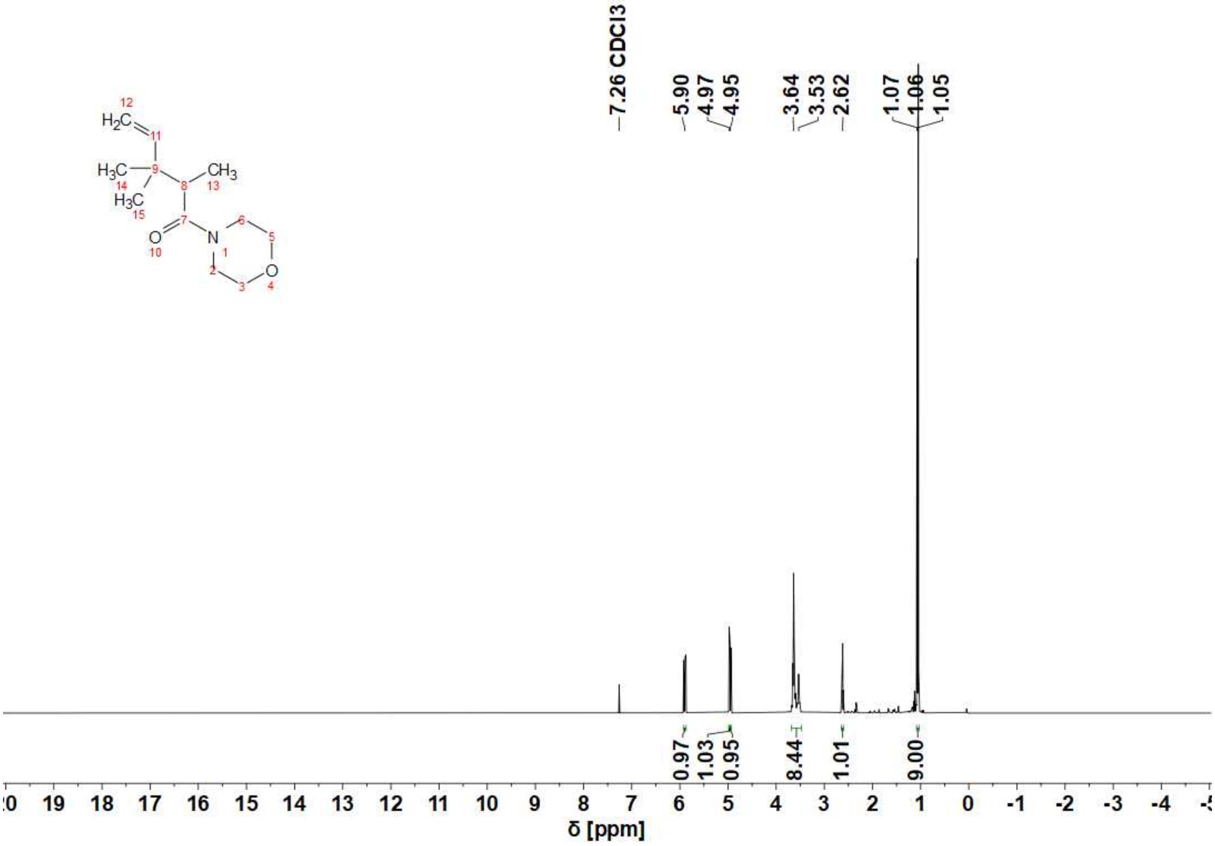

Figure S36: ^{1}H NMR spectrum ($CDCl_3$, 600 MHz) of 2,3,3-trimethyl-1-morpholinopent-4-en-1-one (**3ac**).

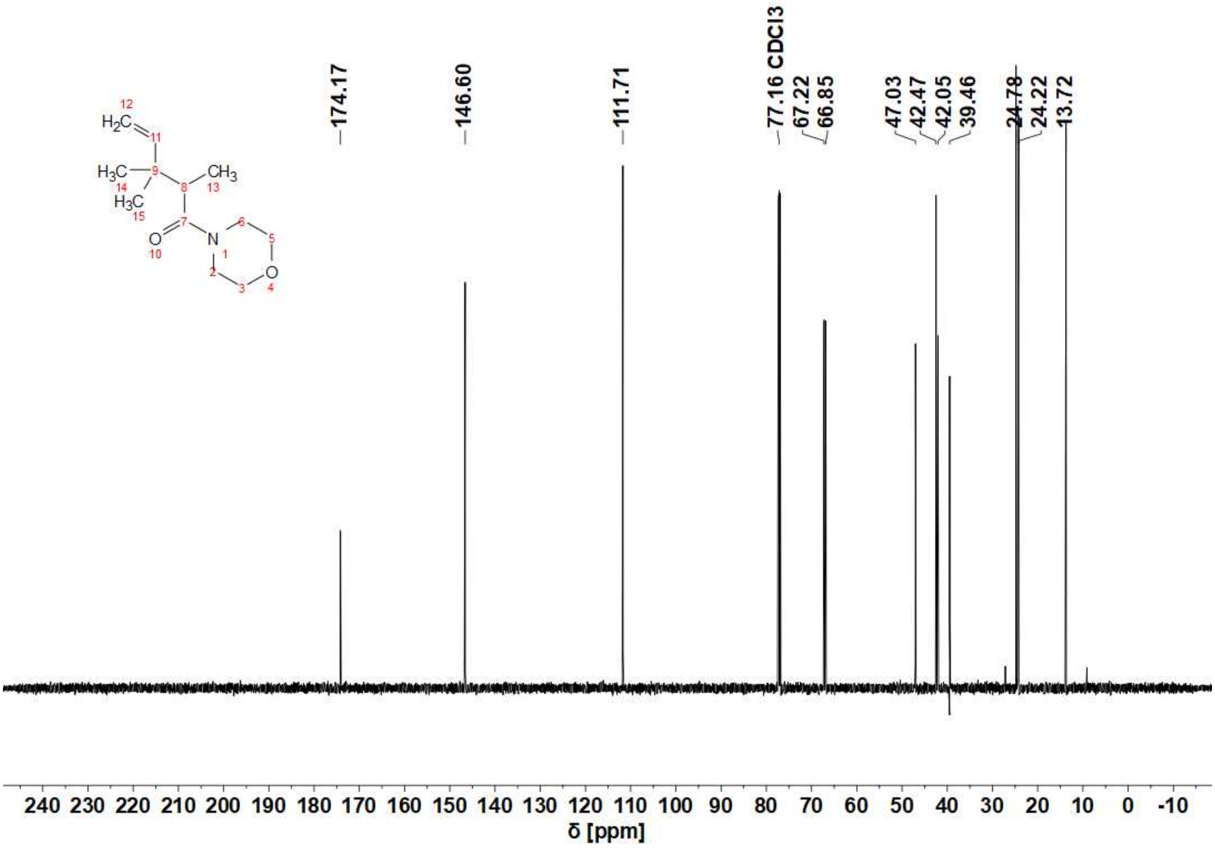

Figure S37: $^{13}C\{^{1}H\}$ NMR spectrum ($CDCl_3$, 151 MHz) of 2,3,3-trimethyl-1-morpholinopent-4-en-1-one (**3ac**).

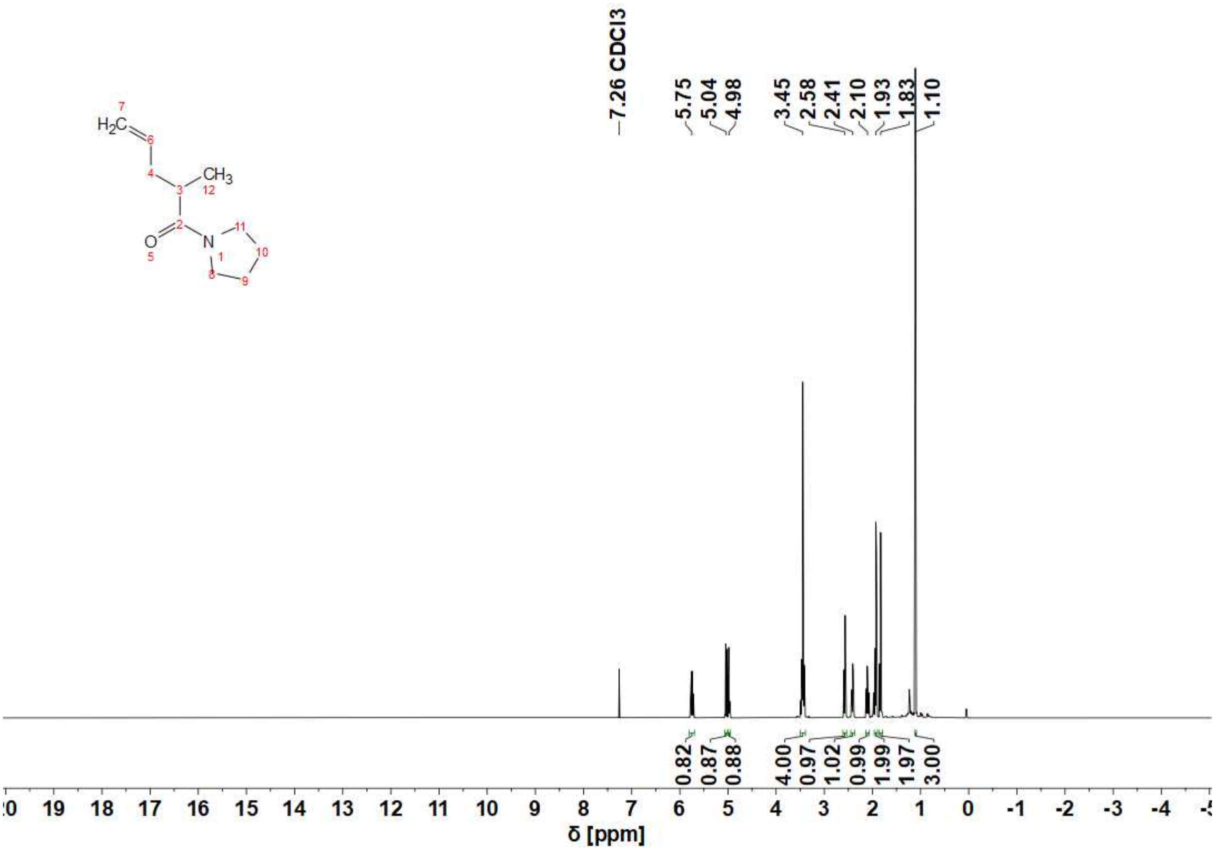

Figure S38: ^{1}H NMR spectrum ($CDCl_3$, 600 MHz) of 2-methyl-1-(pyrrolidin-1-yl)pent-4-en-1-one (**3ad**).

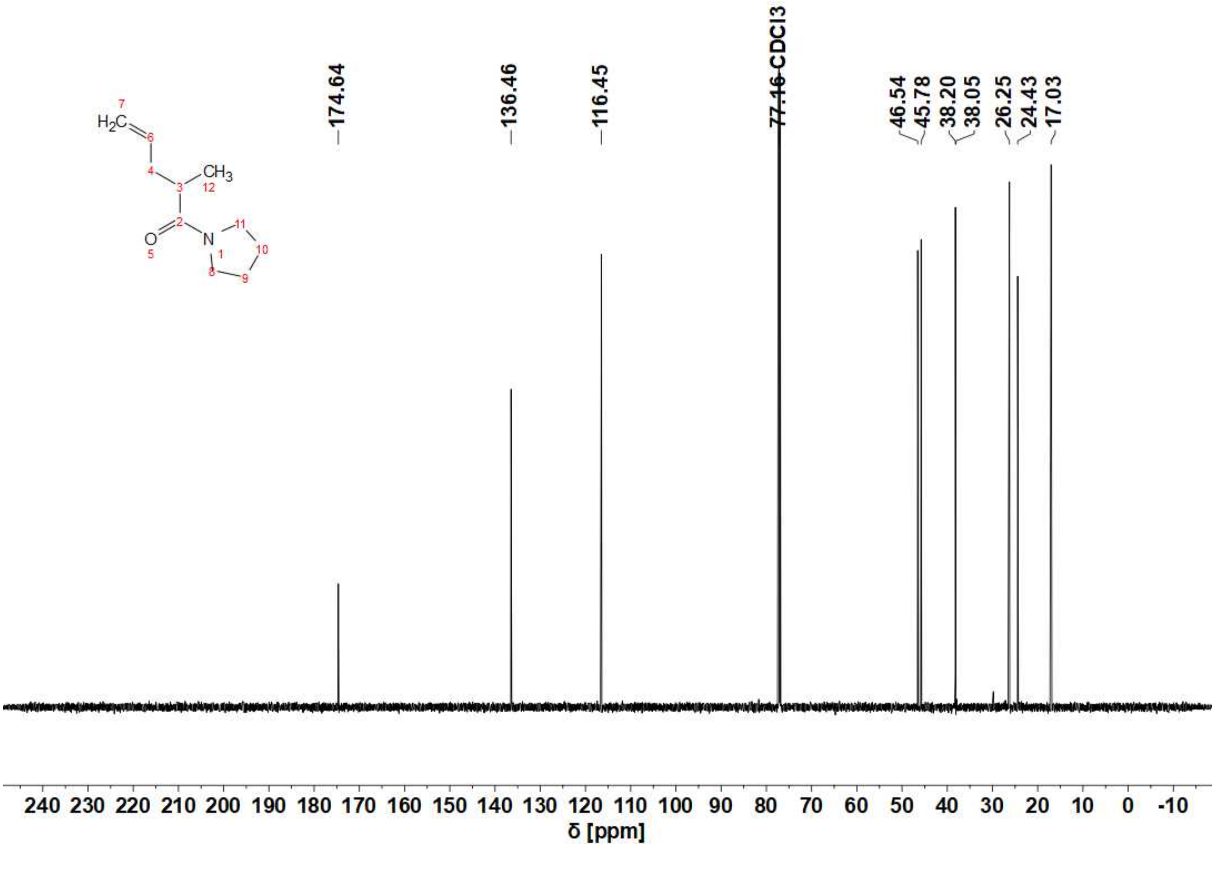

Figure S39: $^{13}C\{^1H\}$ NMR spectrum ($CDCl_3$, 151 MHz) of 2-methyl-1-(pyrrolidin-1-yl)pent-4-en-1-one (**3ad**).

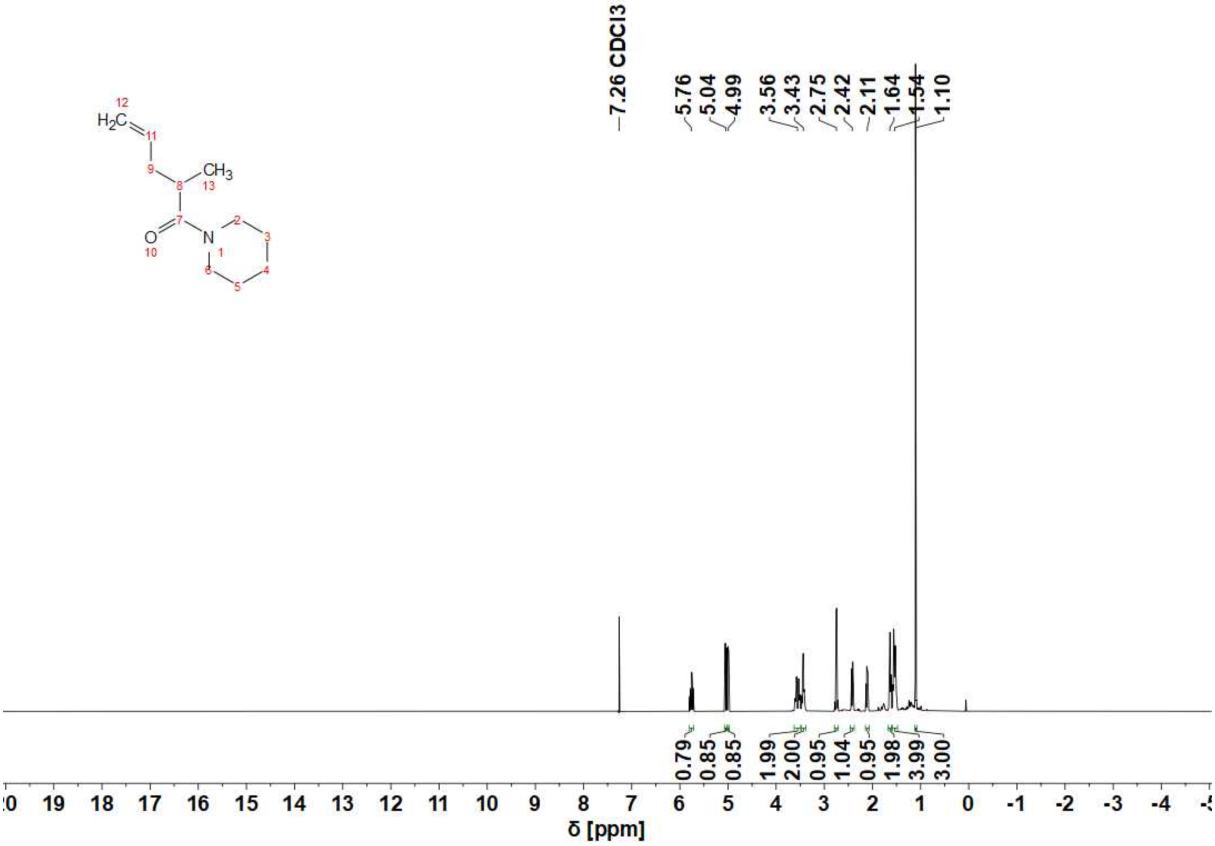

Figure S40: ^{1}H NMR spectrum ($CDCl_3$, 600 MHz) of 2-methyl-1-(piperidin-1-yl)pent-4-en-1-one (**3ae**).

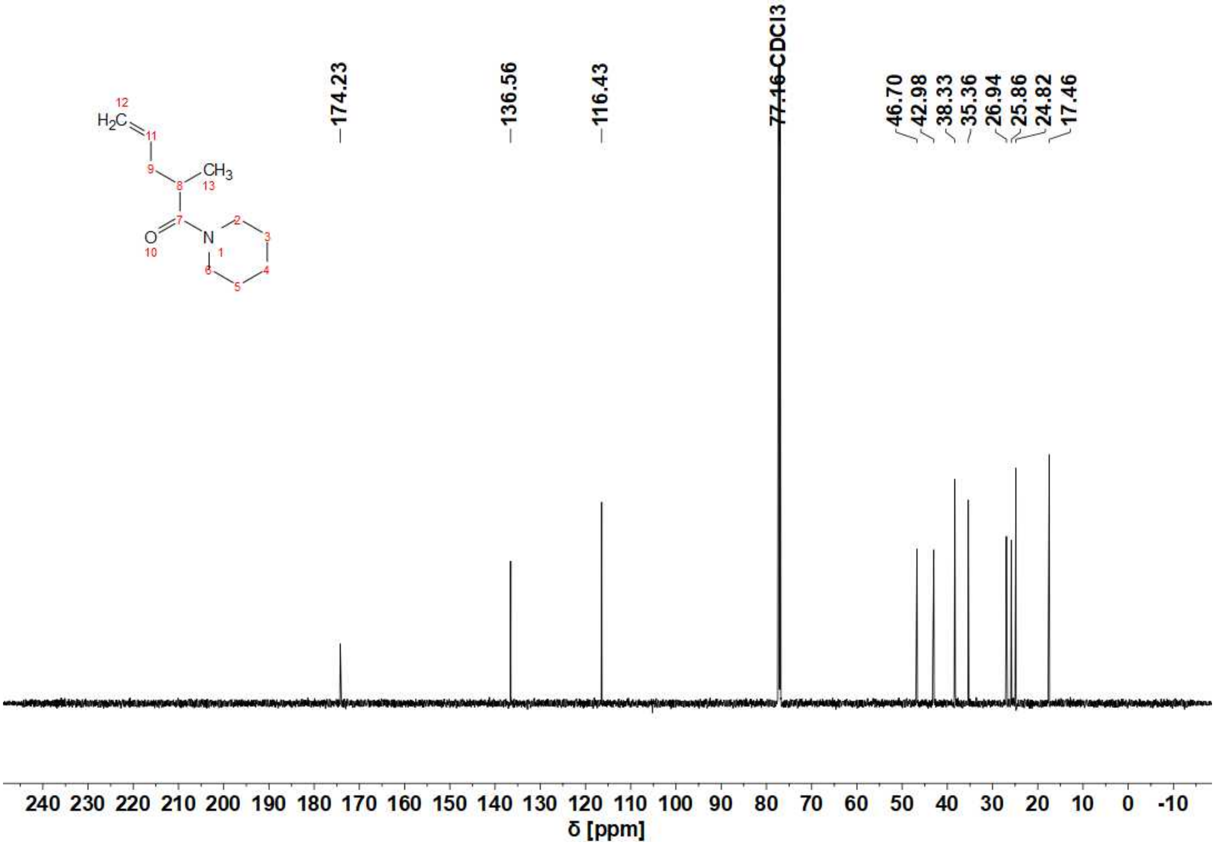

Figure S41: $^{13}C\{^1H\}$ NMR spectrum ($CDCl_3$, 151 MHz) of 2-methyl-1-(piperidin-1-yl)pent-4-en-1-one (**3ae**).

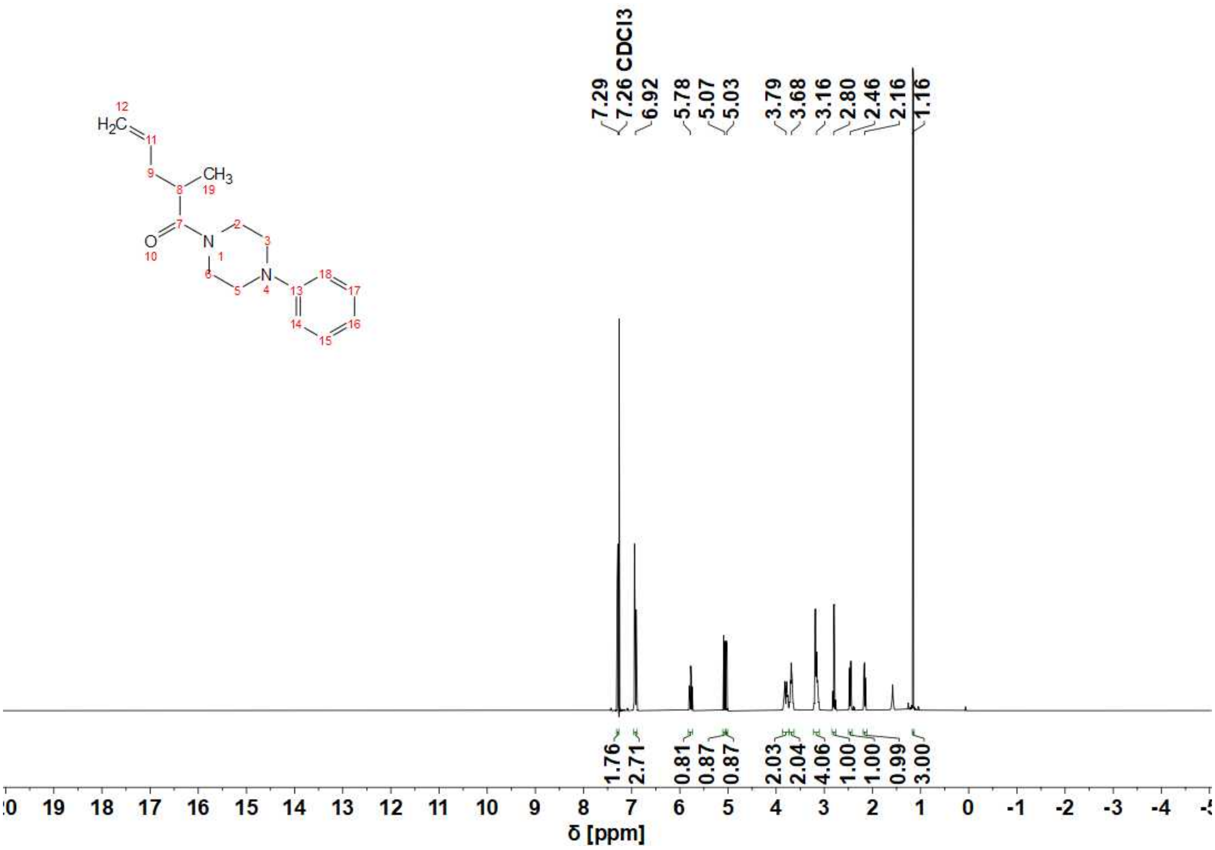

Figure S42: ^{1}H NMR spectrum ($CDCl_3$, 600 MHz) of 2-methyl-1-(4-phenylpiperazin-1-yl)pent-4-en-1-one (**3af**).

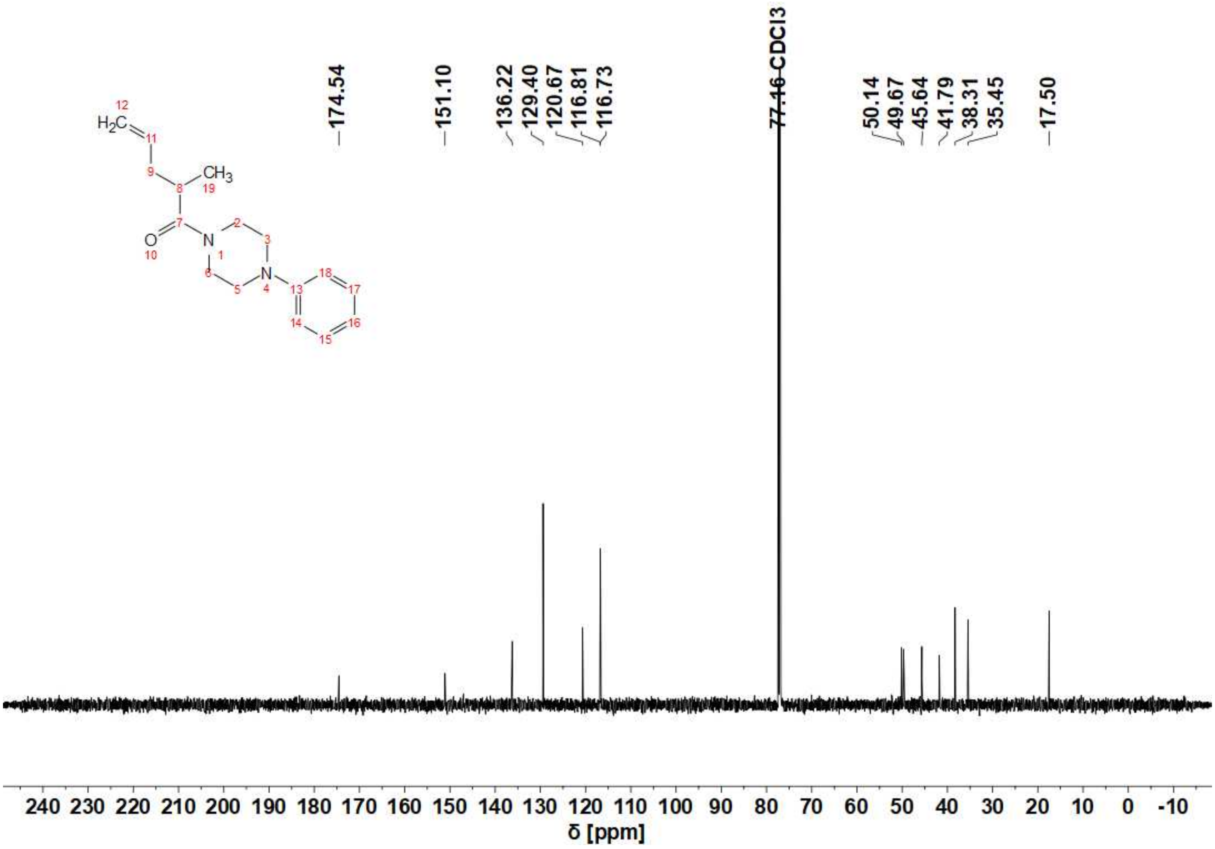

Figure S43: $^{13}C\{^{1}H\}$ NMR spectrum ($CDCl_3$, 151 MHz) of 2-methyl-1-(4-phenylpiperazin-1-yl)pent-4-en-1-one (**3af**).

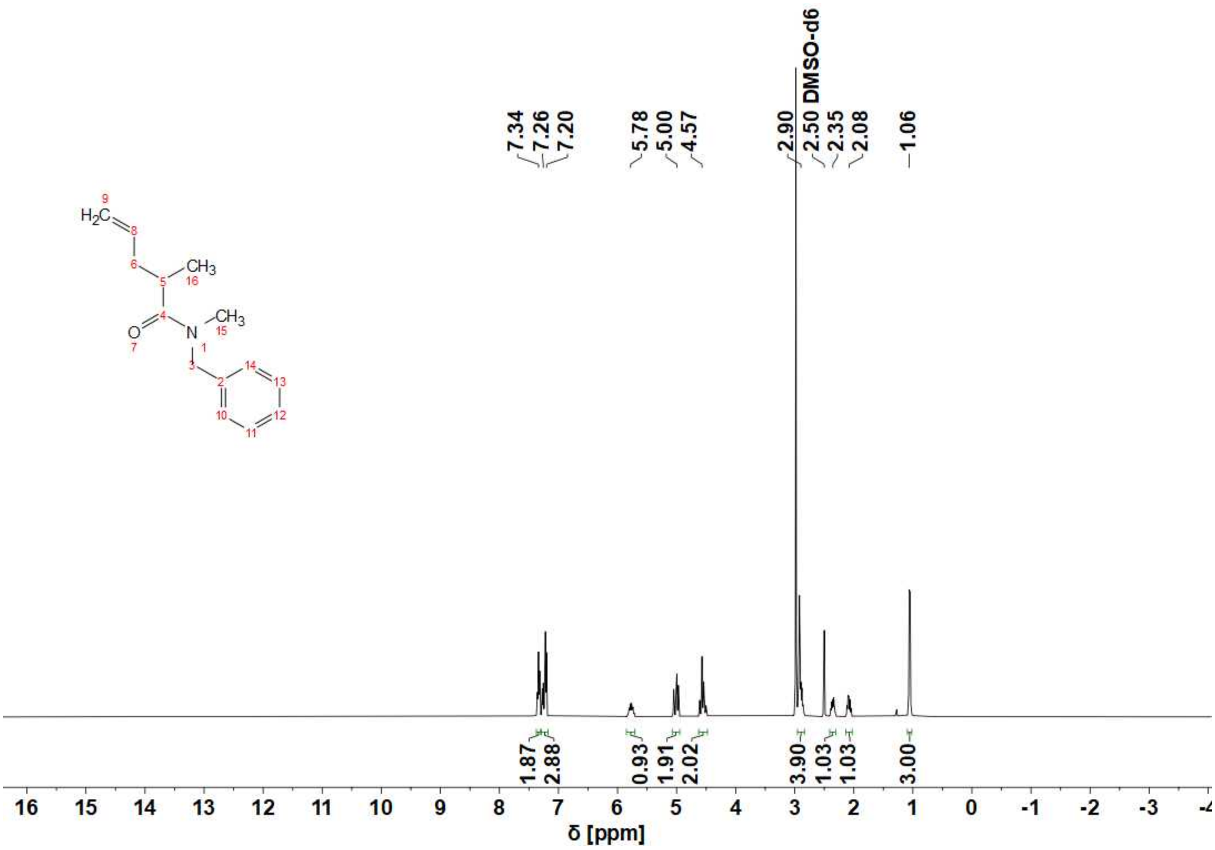

Figure S44: ^{1}H NMR spectrum (100 °C, DMSO-d_6, 400 MHz) of *N*-benzyl-*N*,2-dimethylpent-4-enamide (**3ag**).

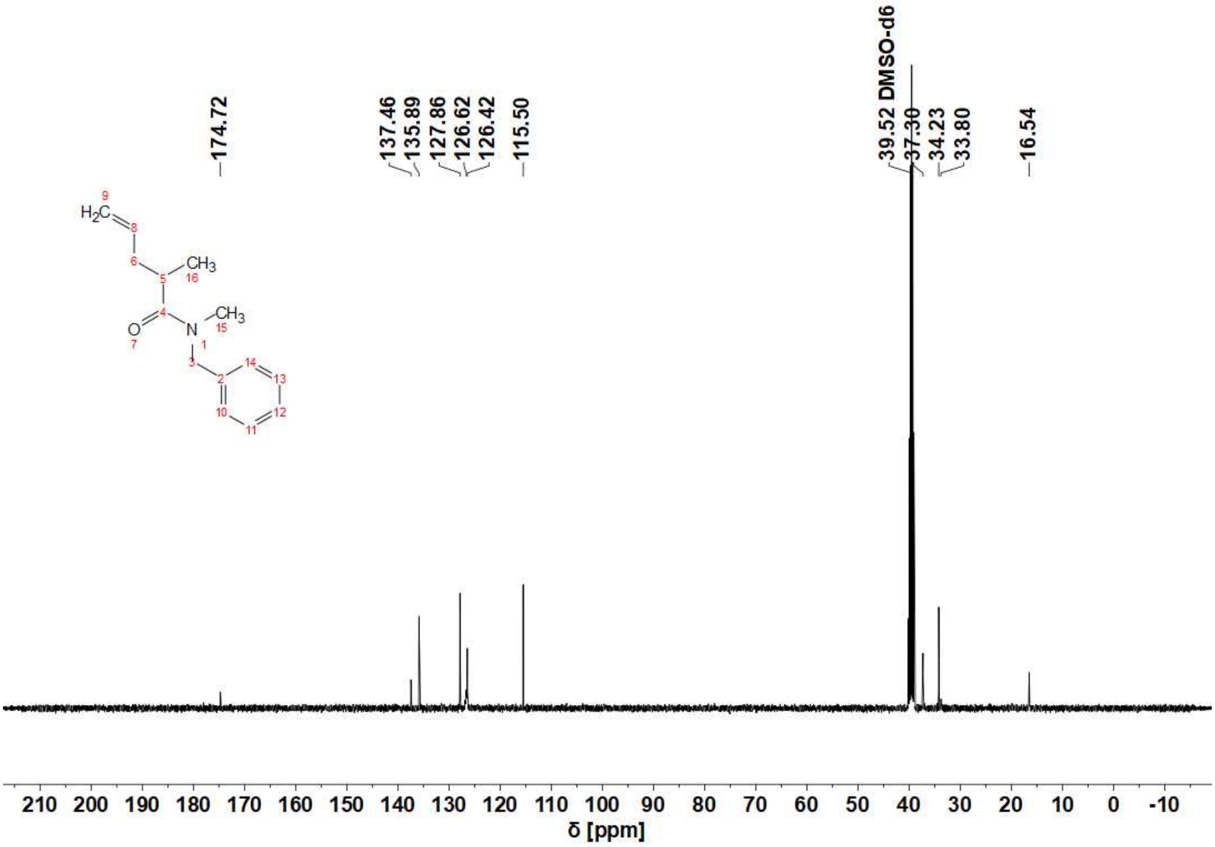

Figure S45: $^{13}C\{^{1}H\}$ NMR spectrum (100 °C, DMSO-d_6, 101 MHz) of *N*-benzyl-*N*,2-dimethylpent-4-enamide (**3ag**).

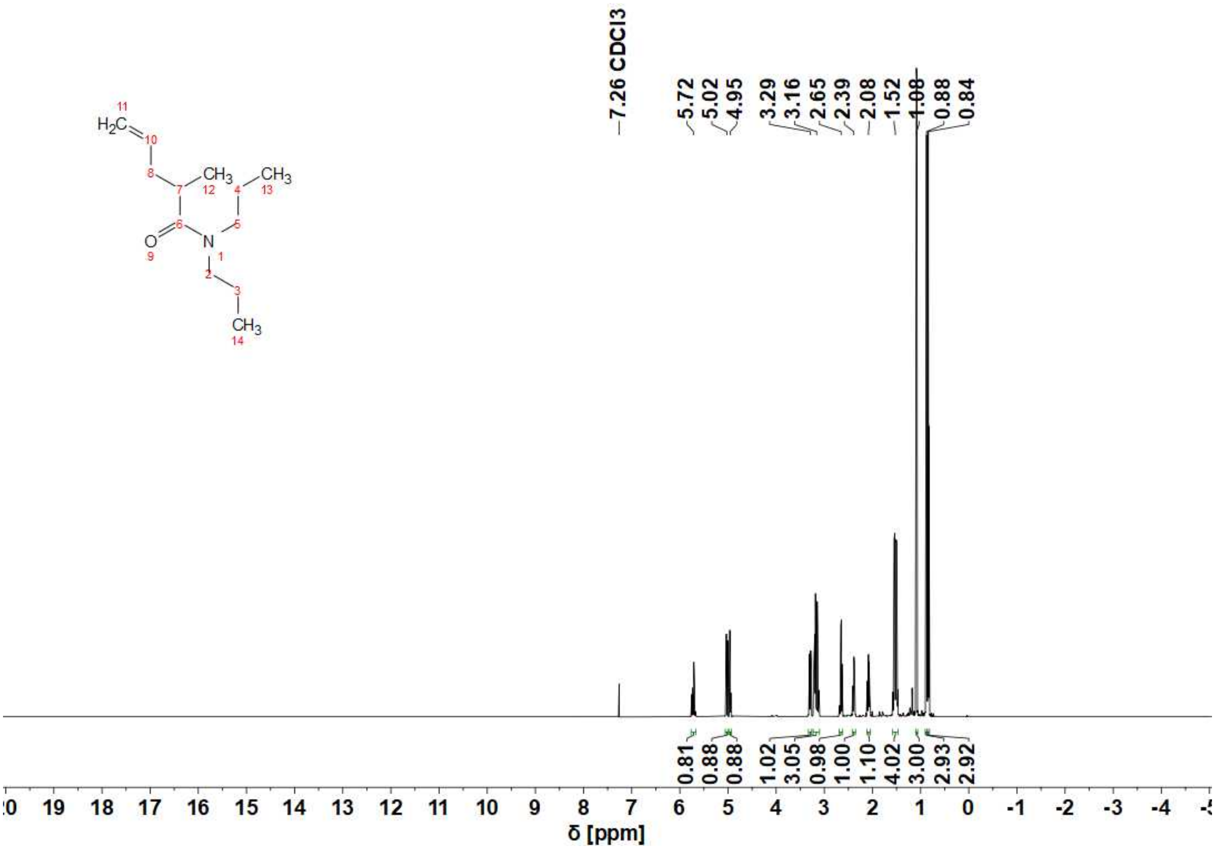

Figure S46: ^{1}H NMR spectrum ($CDCl_3$, 600 MHz) of 2-methyl-*N*,*N*-dipropylpent-4-enamide (**3ah**).

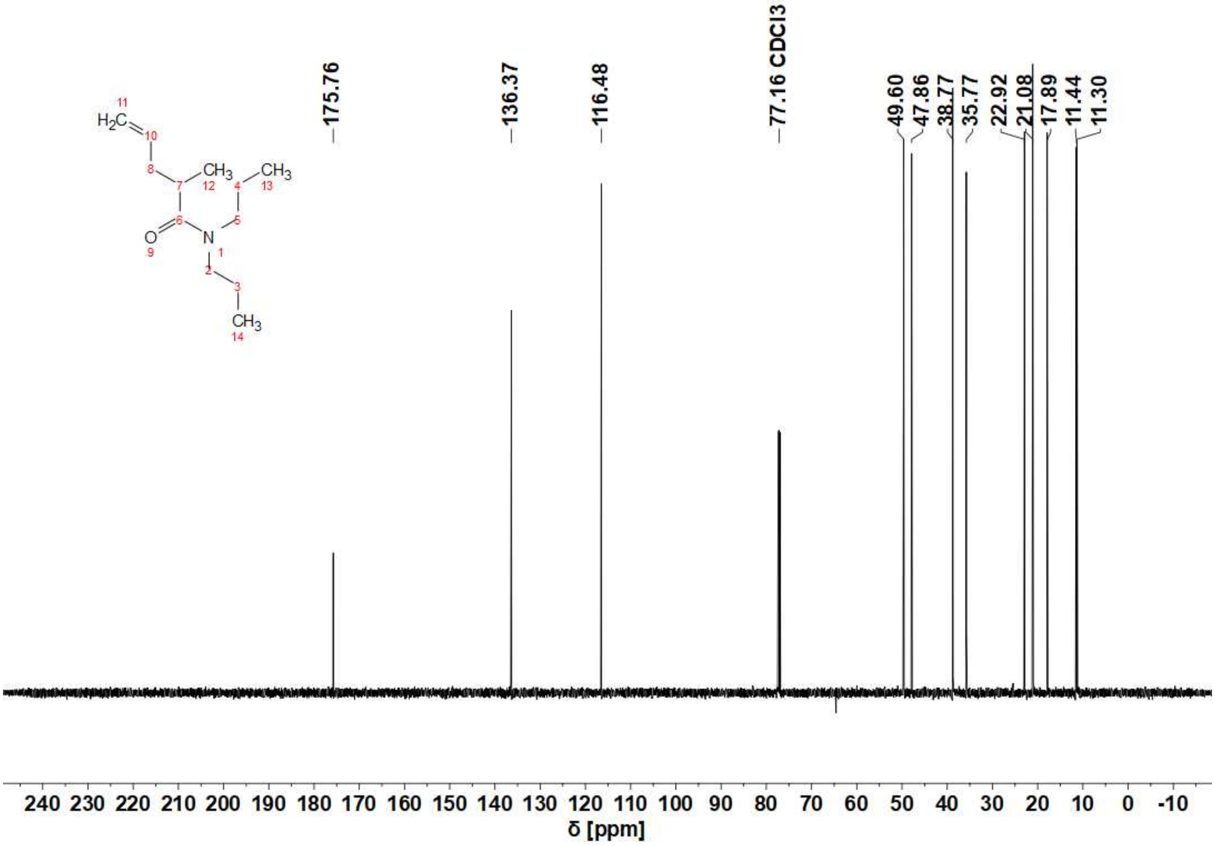

Figure S47: $^{13}C\{^{1}H\}$ NMR spectrum ($CDCl_3$, 151 MHz) of 2-methyl-*N*,*N*-dipropylpent-4-enamide (**3ah**).

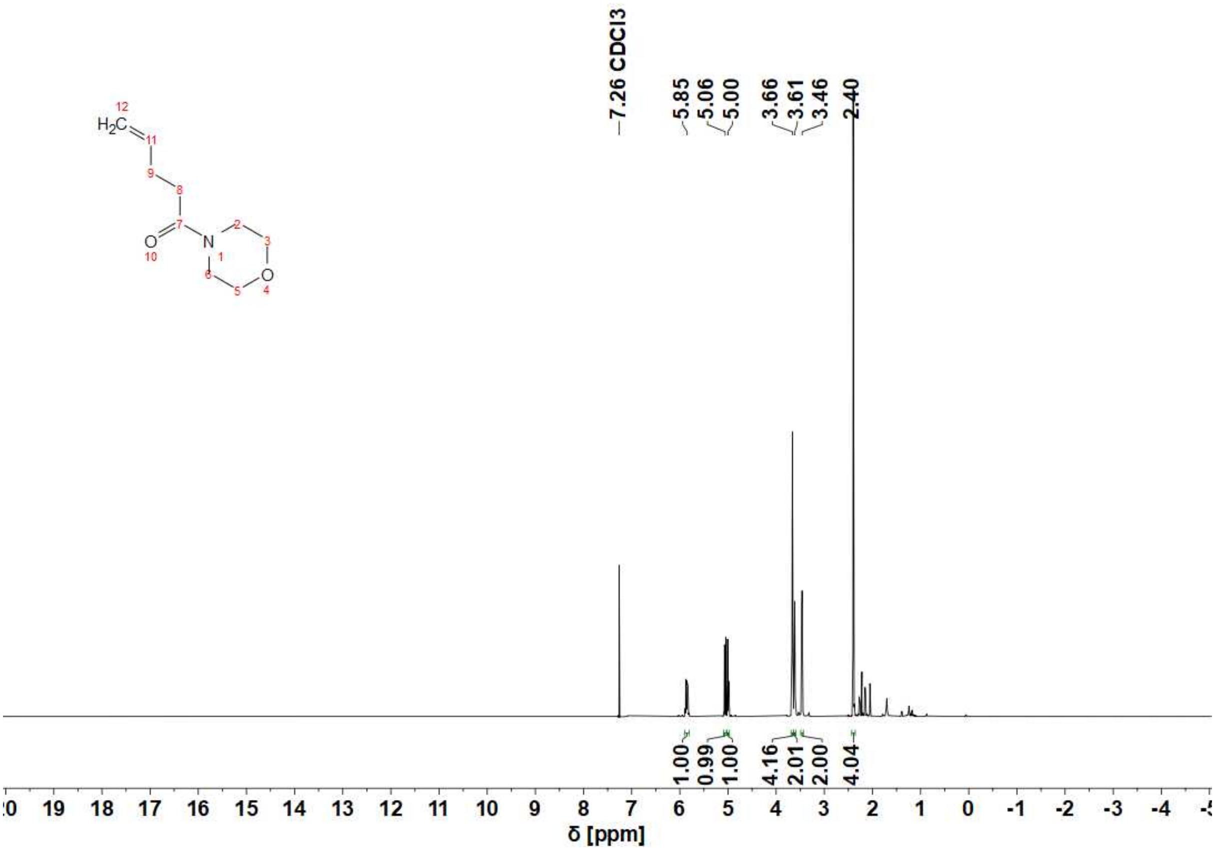

Figure S48: ^{1}H NMR spectrum ($CDCl_3$, 600 MHz) of 1-morpholinopent-4-en-1-one (**3ba**).

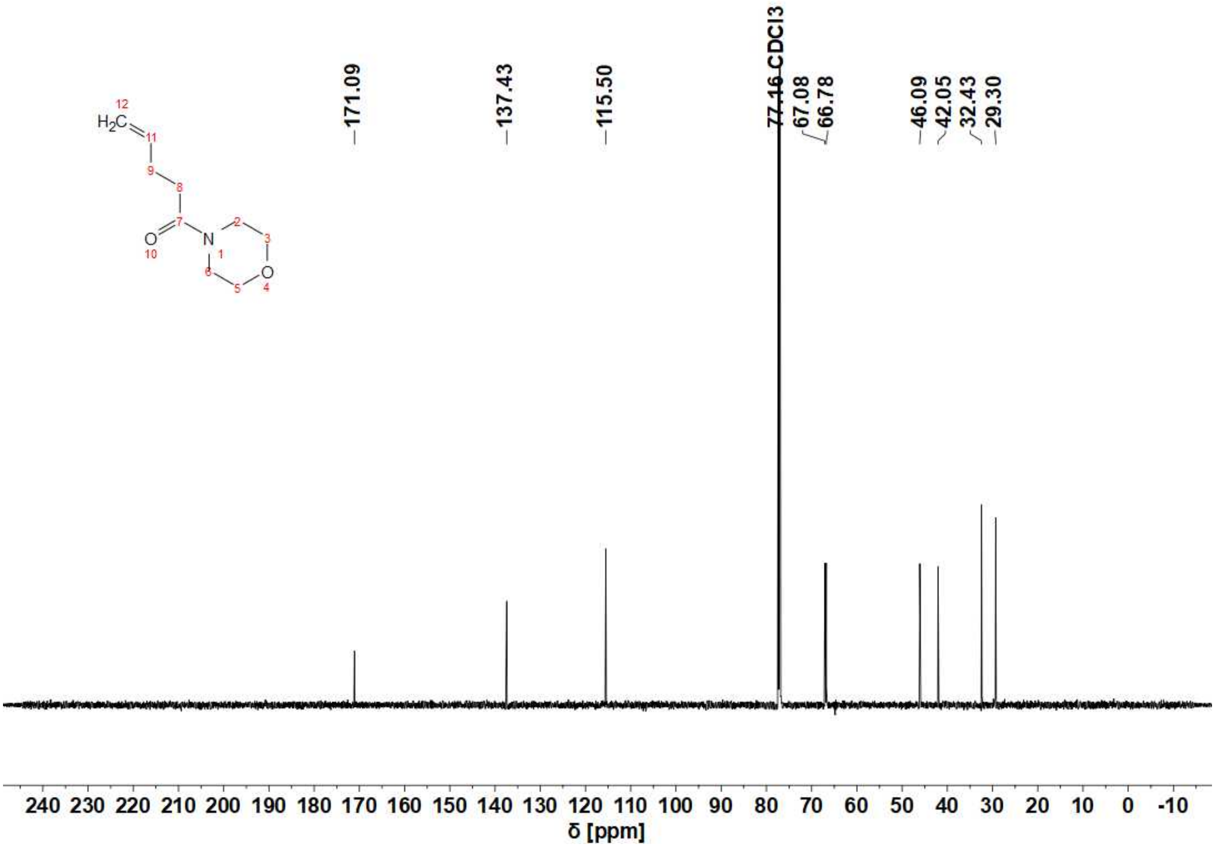

Figure S49: $^{13}C\{^{1}H\}$ NMR spectrum ($CDCl_3$, 151 MHz) of 1-morpholinopent-4-en-1-one (**3ba**).

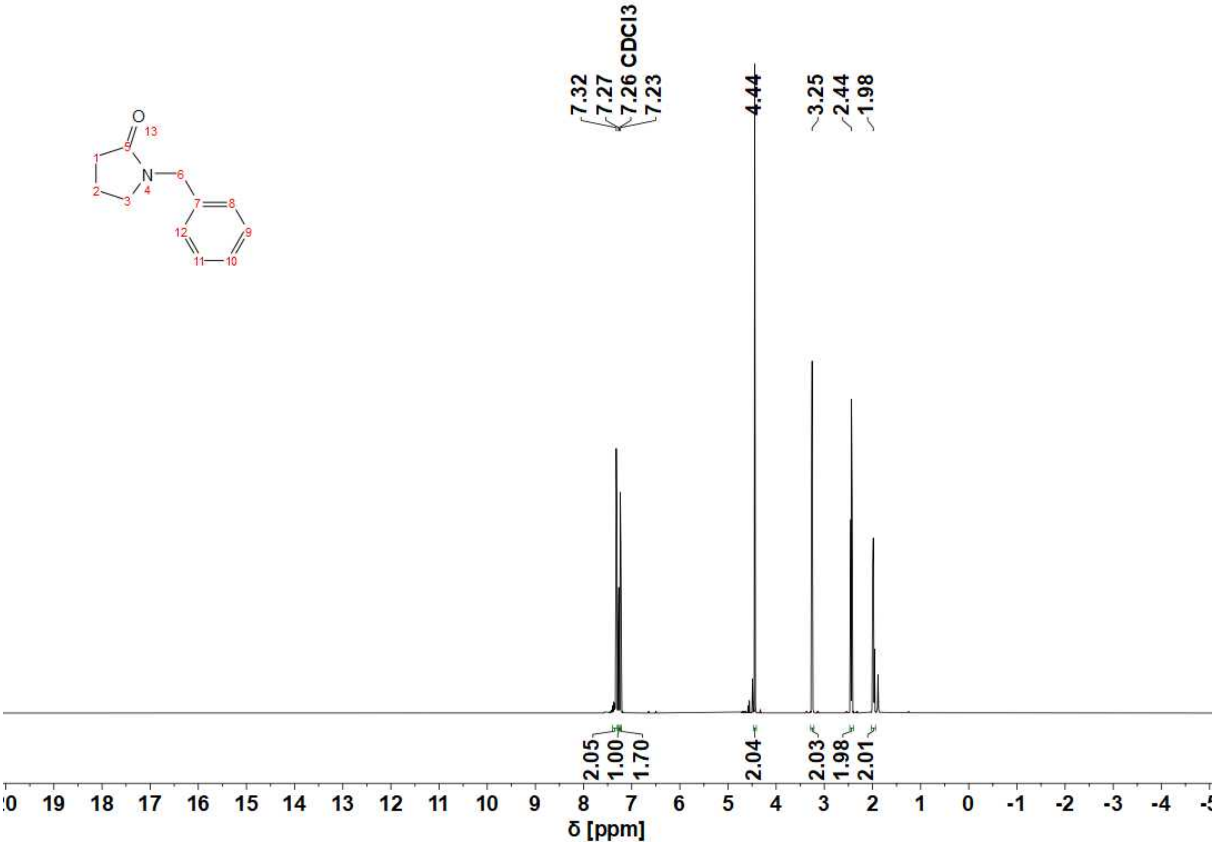

Figure S50: ^{1}H NMR spectrum ($CDCl_3$, 600 MHz) of *N*-benzylpyrrolidone (**S1**).

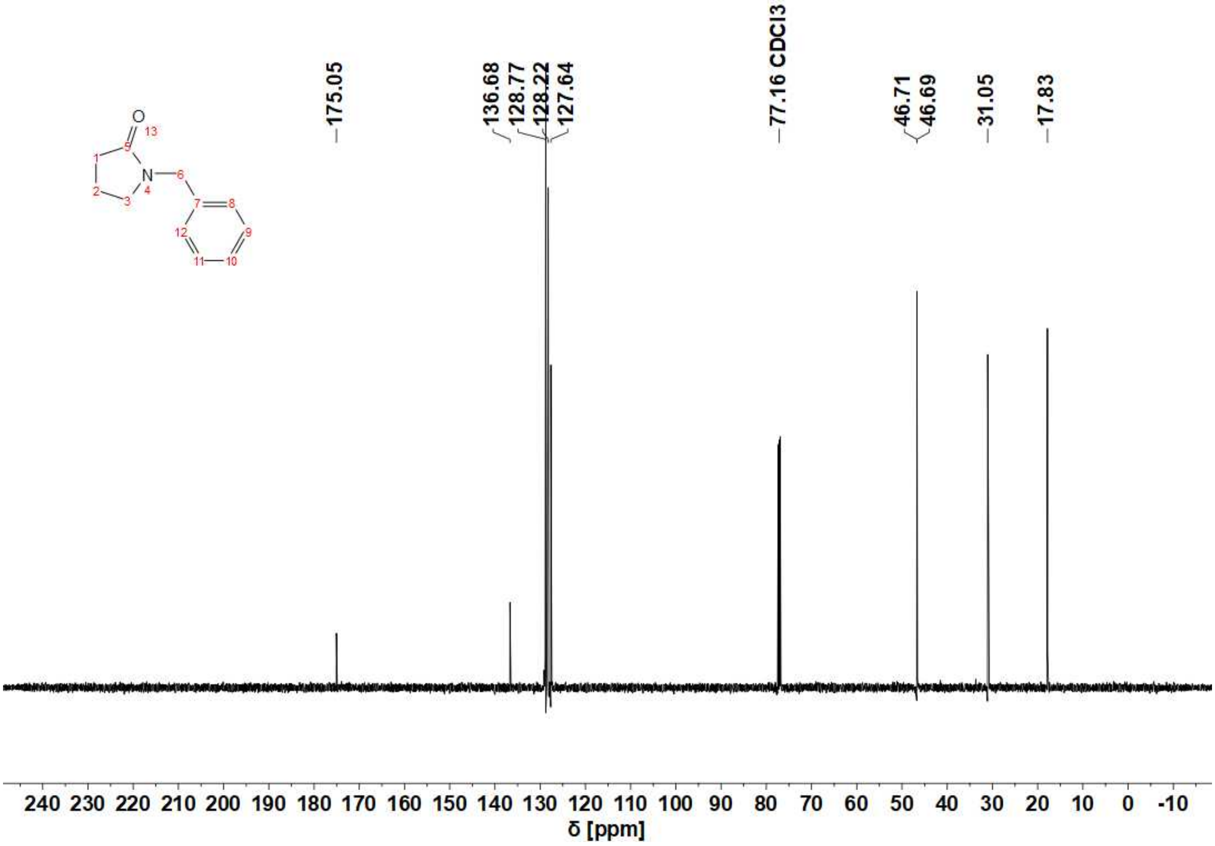

Figure S51: $^{13}C\{^{1}H\}$ NMR spectrum ($CDCl_3$, 151 MHz) of *N*-benzylpyrrolidone (**S1**).

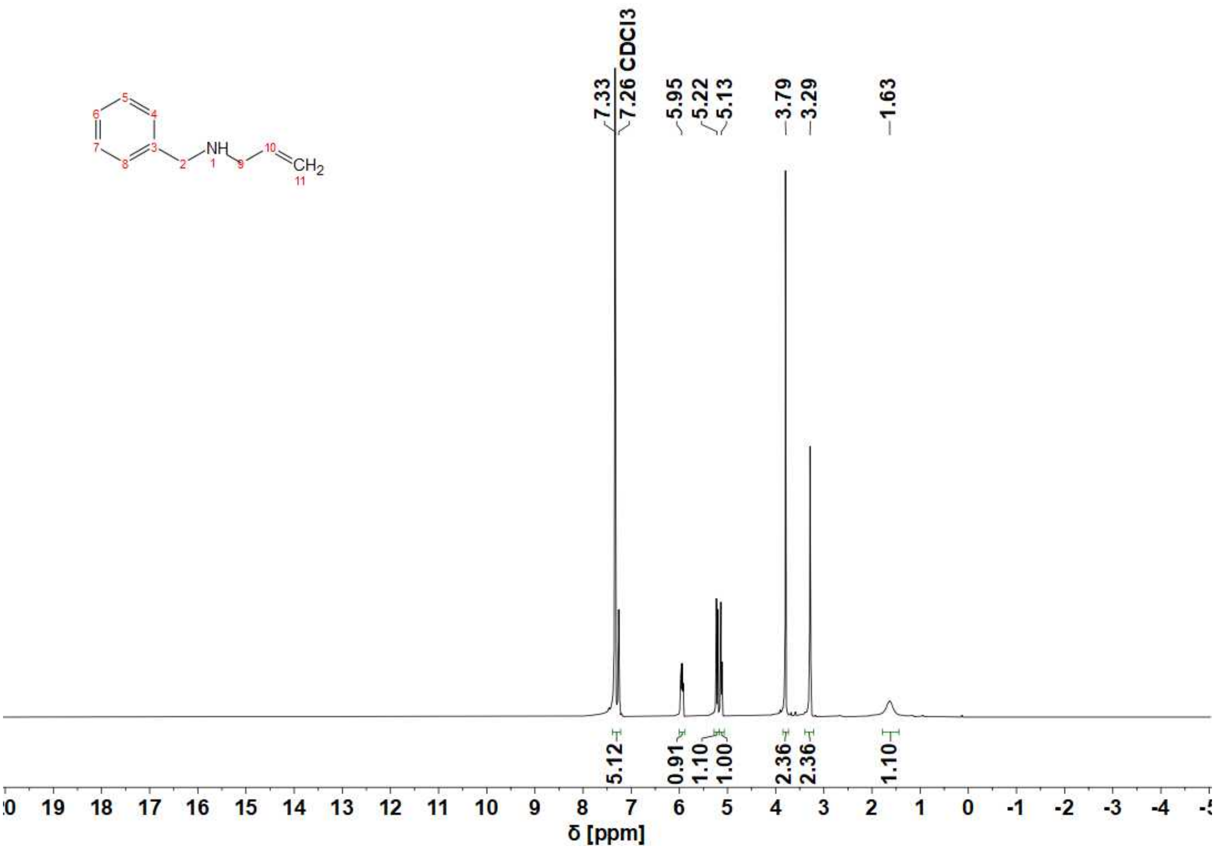

Figure S52: ^{1}H NMR spectrum ($CDCl_3$, 600 MHz) of *N*-benzylprop-2-en-1-amine (**S2**).

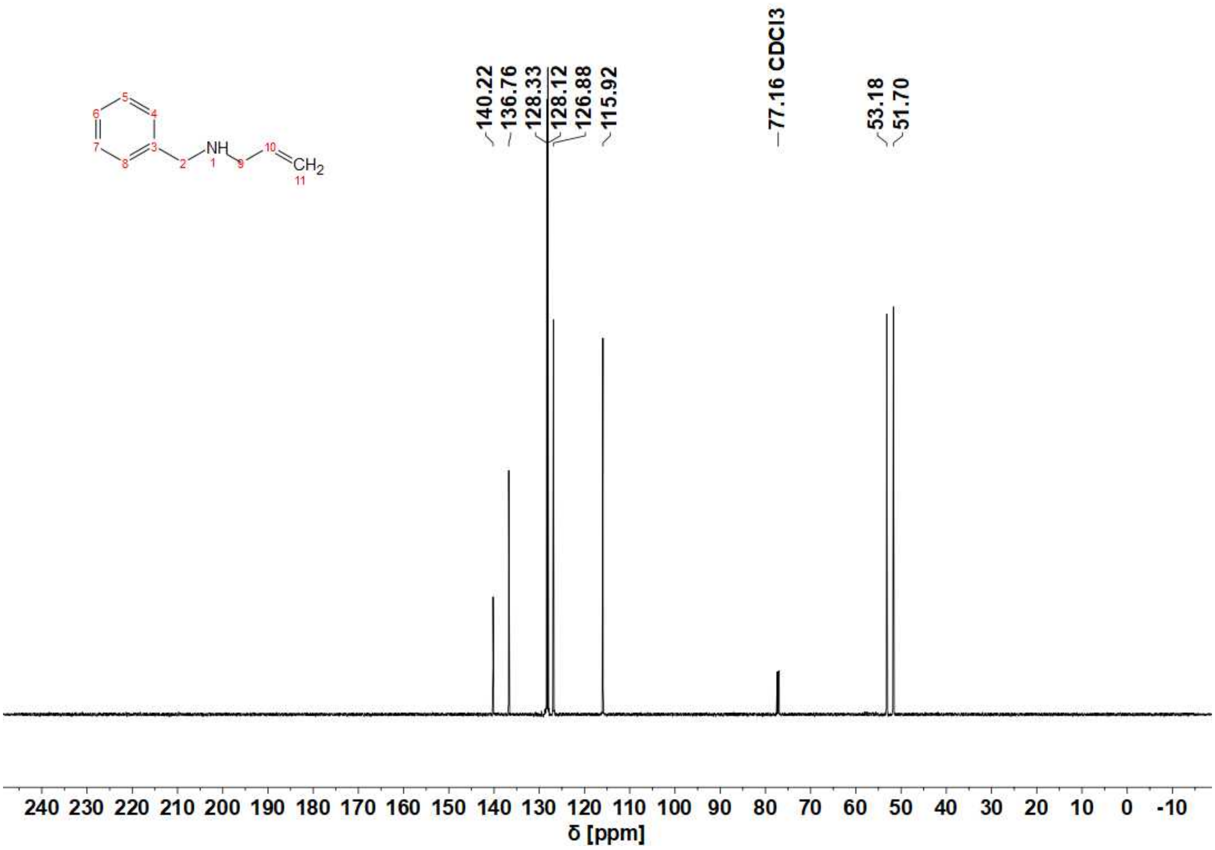

Figure S53: $^{13}C\{^{1}H\}$ NMR spectrum ($CDCl_3$, 151 MHz) of *N*-benzylprop-2-en-1-amine (**S2**).

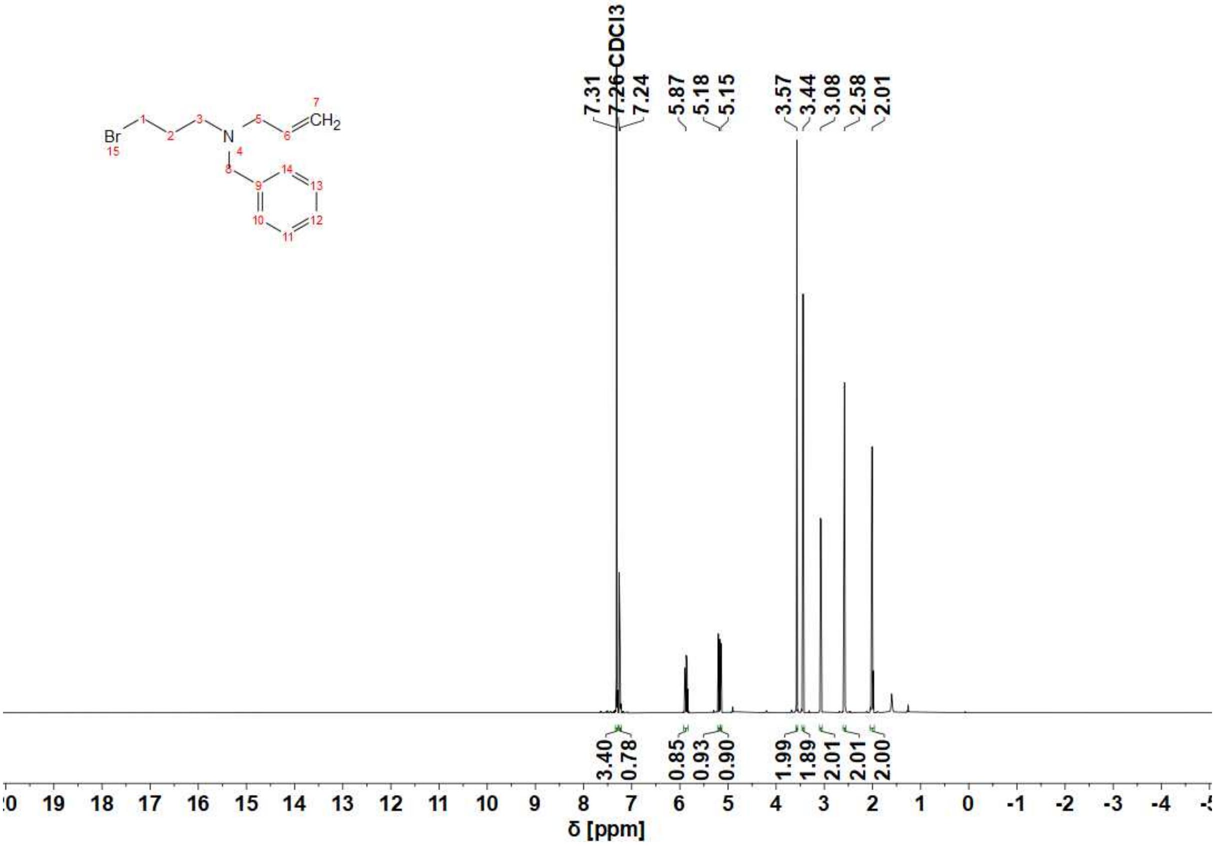

Figure S54: ^{1}H NMR spectrum ($CDCl_3$, 600 MHz) of *N*-benzyl-*N*-(3-bromopropyl)prop-2-en-1-amine (**S3a**).

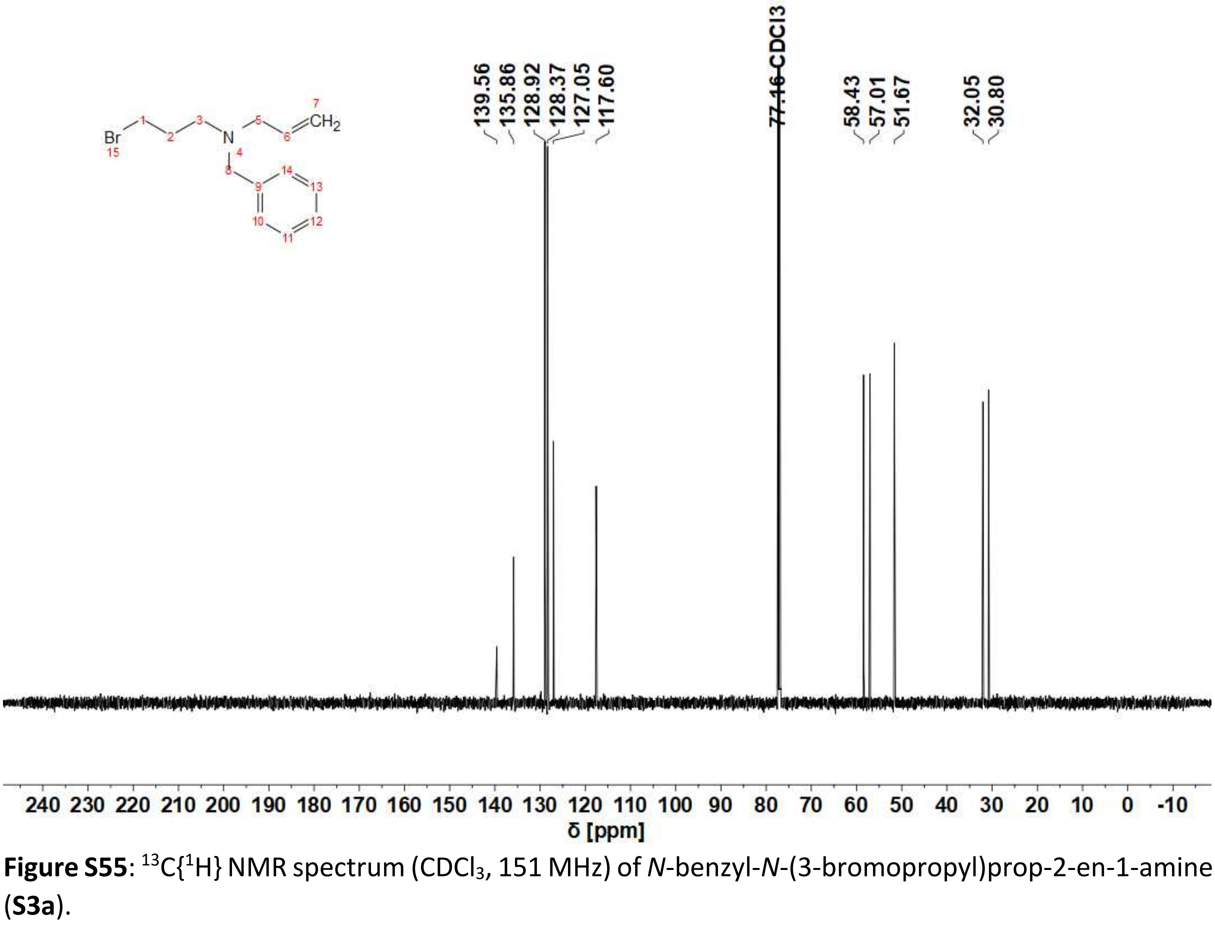

Figure S55: $^{13}C\{^{1}H\}$ NMR spectrum ($CDCl_3$, 151 MHz) of *N*-benzyl-*N*-(3-bromopropyl)prop-2-en-1-amine (**S3a**).

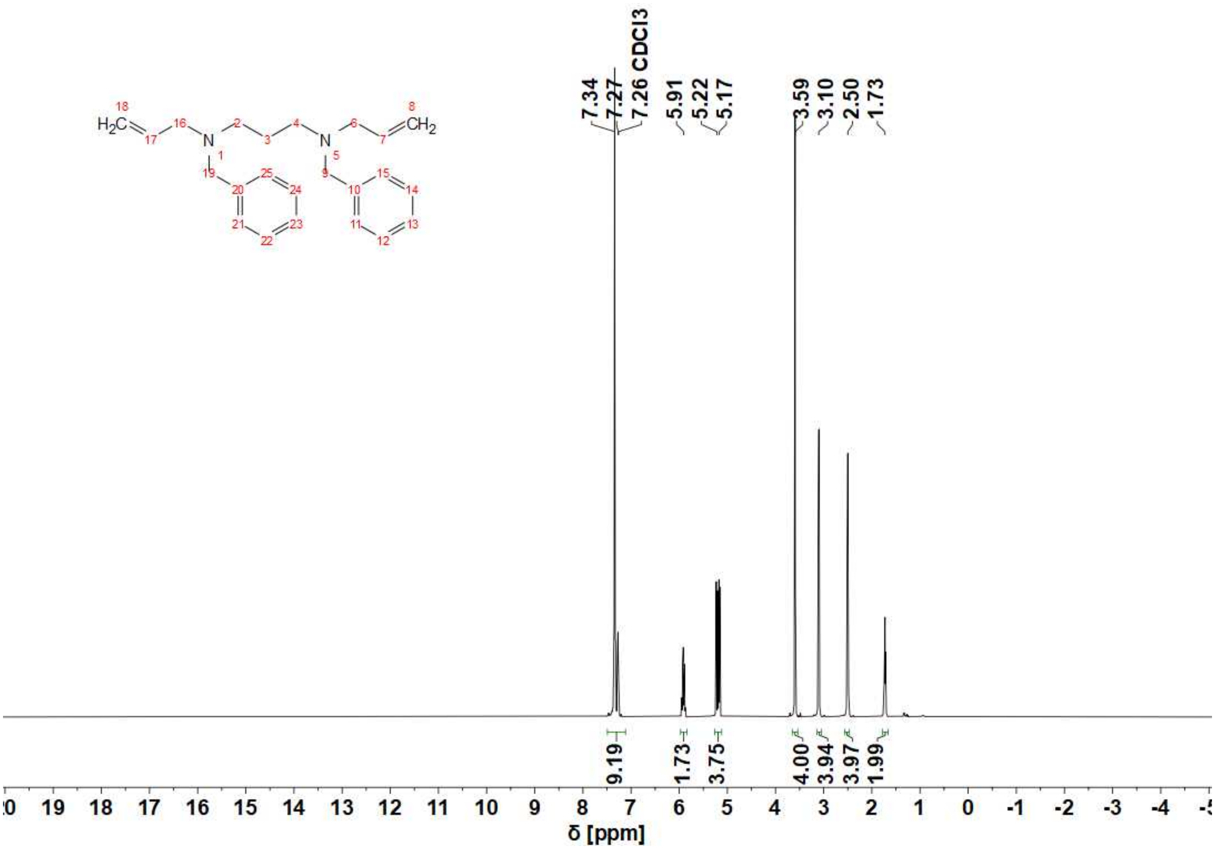

Figure S56: ^{1}H NMR spectrum ($CDCl_3$, 600 MHz) of *N*[1],*N*[3]-diallyl-*N*[1],*N*[3]-dibenzylpropane-1,3-diamine (**S3b**).

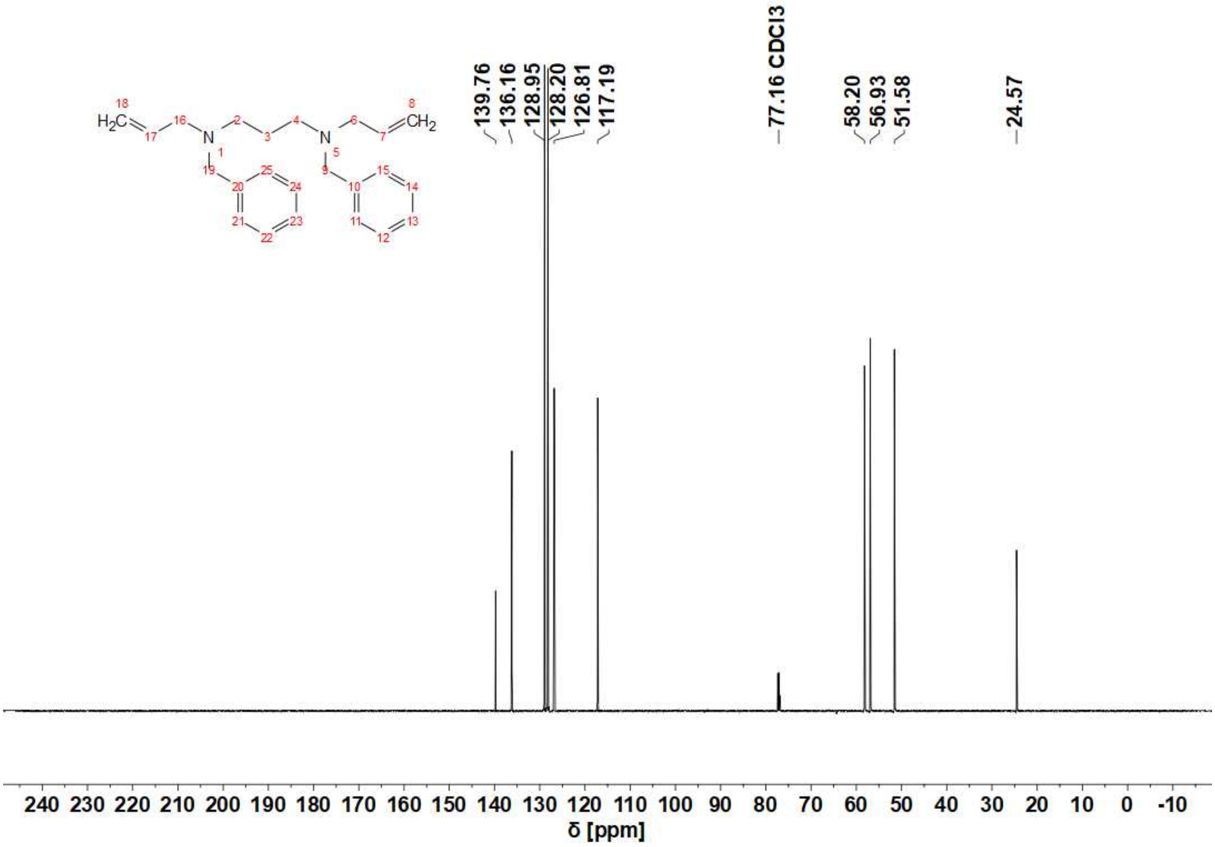

Figure S57: ^{13}C{^{1}H} NMR spectrum ($CDCl_3$, 151 MHz) of *N*[1],*N*[3]-diallyl-*N*[1],*N*[3]-dibenzylpropane-1,3-diamine (**S3b**).

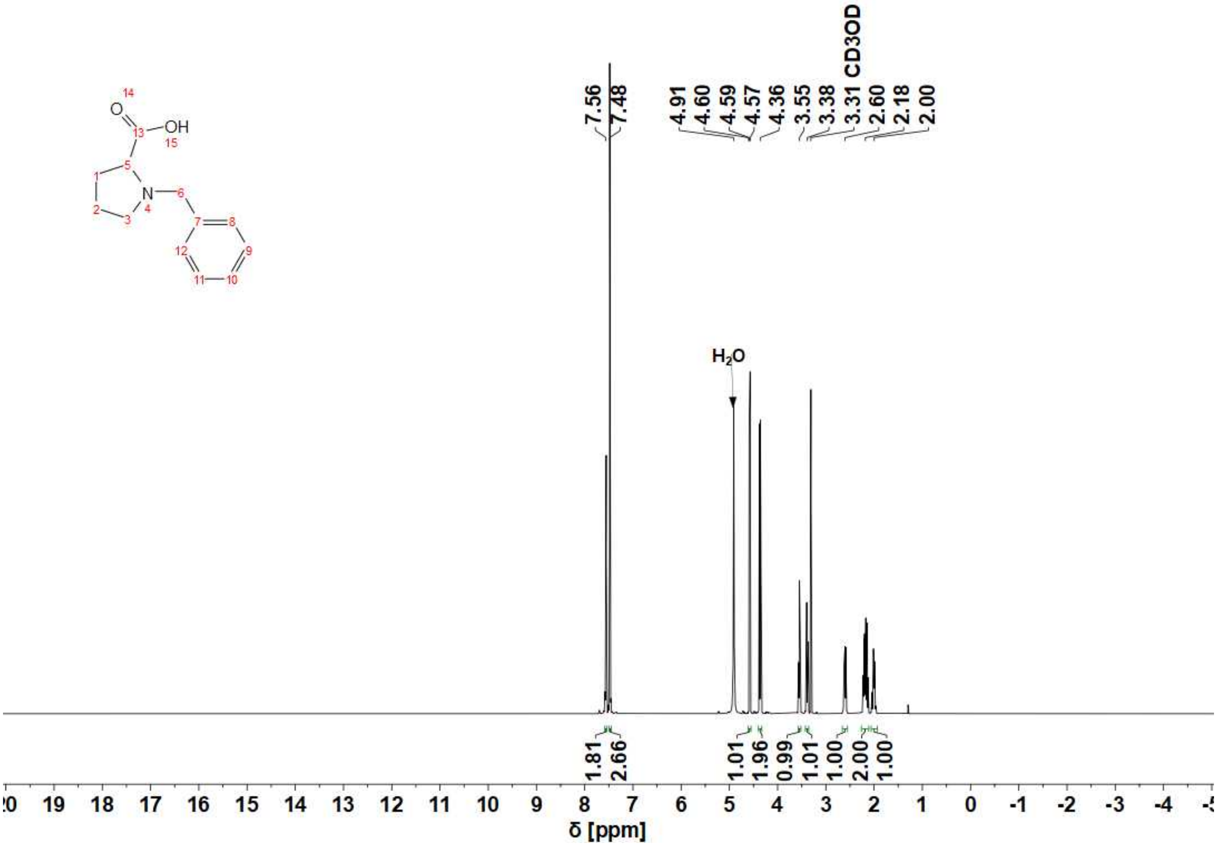

Figure S58: ^{1}H NMR spectrum (CD_3OD, 600 MHz) of *N*-benzylproline (**S4a**).

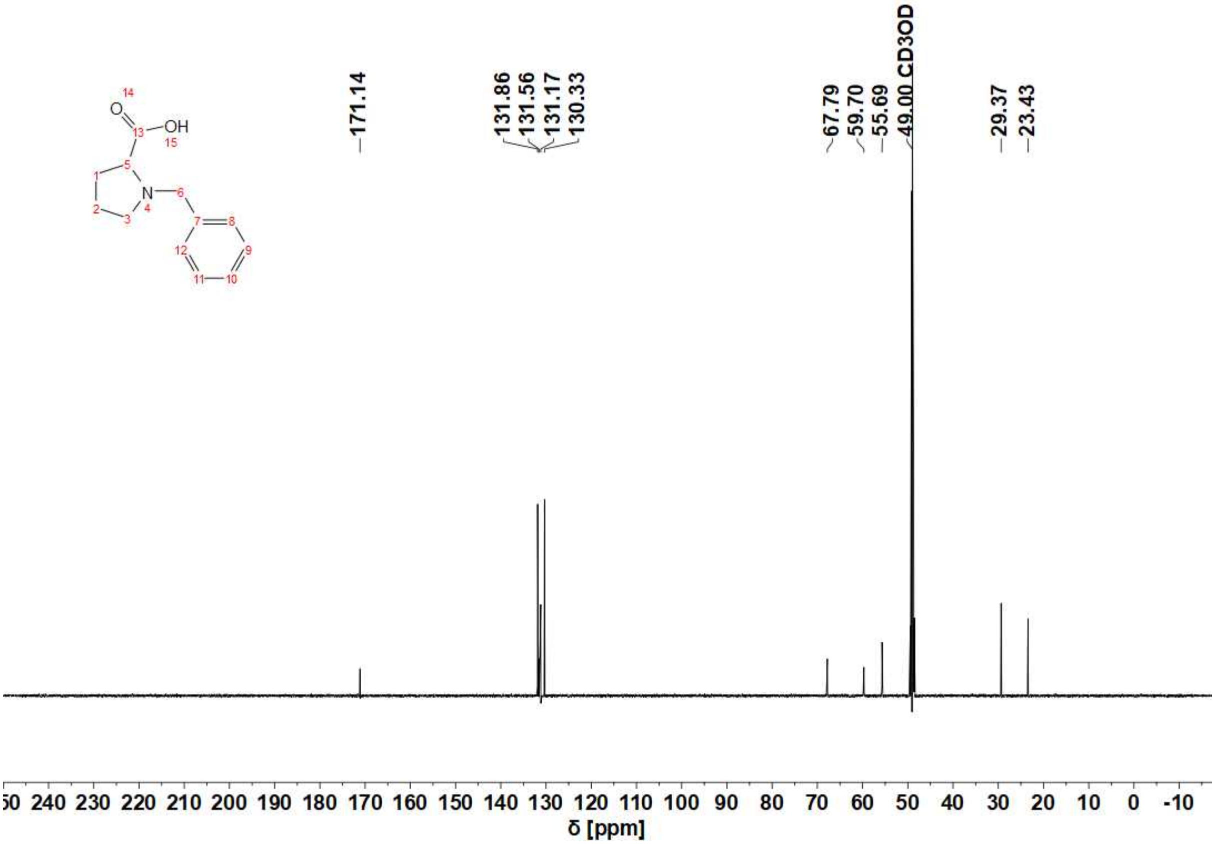

Figure S59: $^{13}C\{^{1}H\}$ NMR spectrum (CD_3OD, 151 MHz) of *N*-benzylproline (**S4a**).

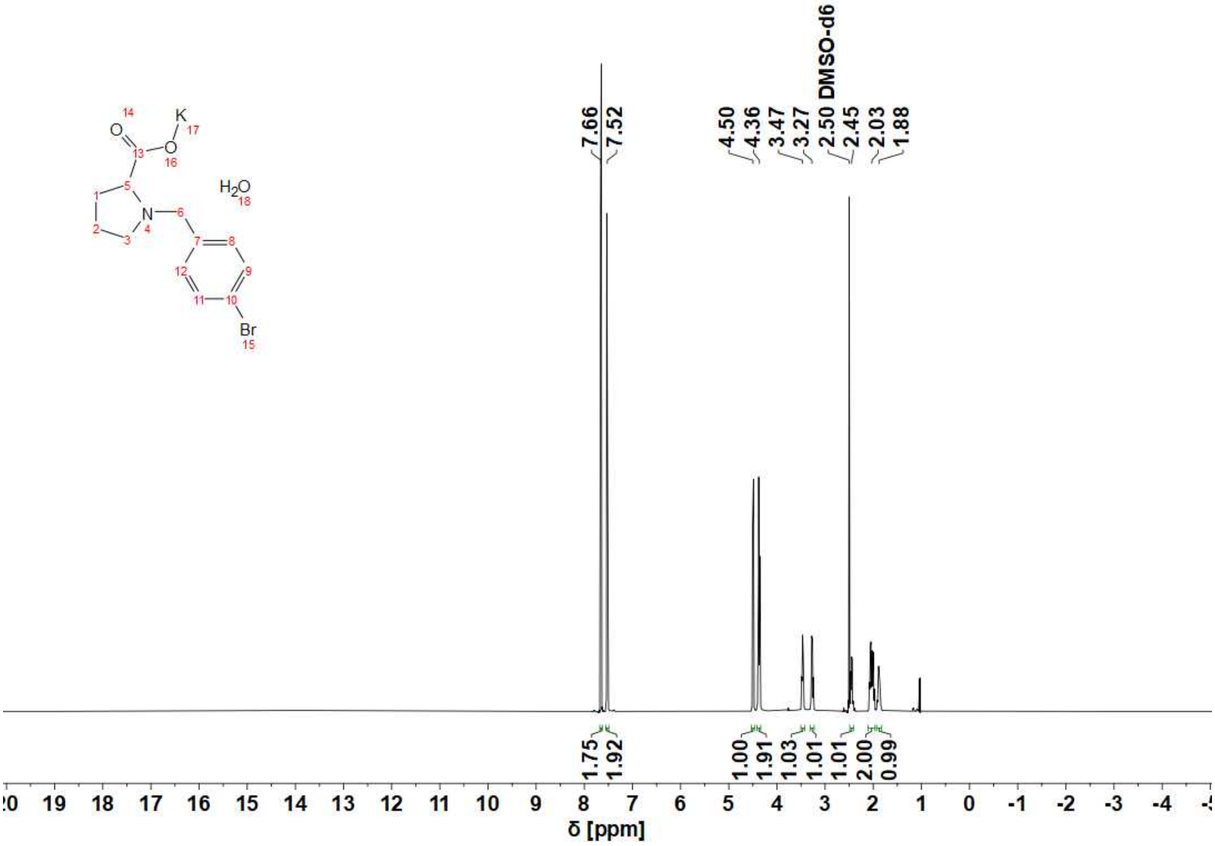

Figure S60: ^{1}H NMR spectrum (DMSO-d_6, 600 MHz) of potassium *N*-(4-bromobenzyl)prolinate monohydrate (**S4b**).

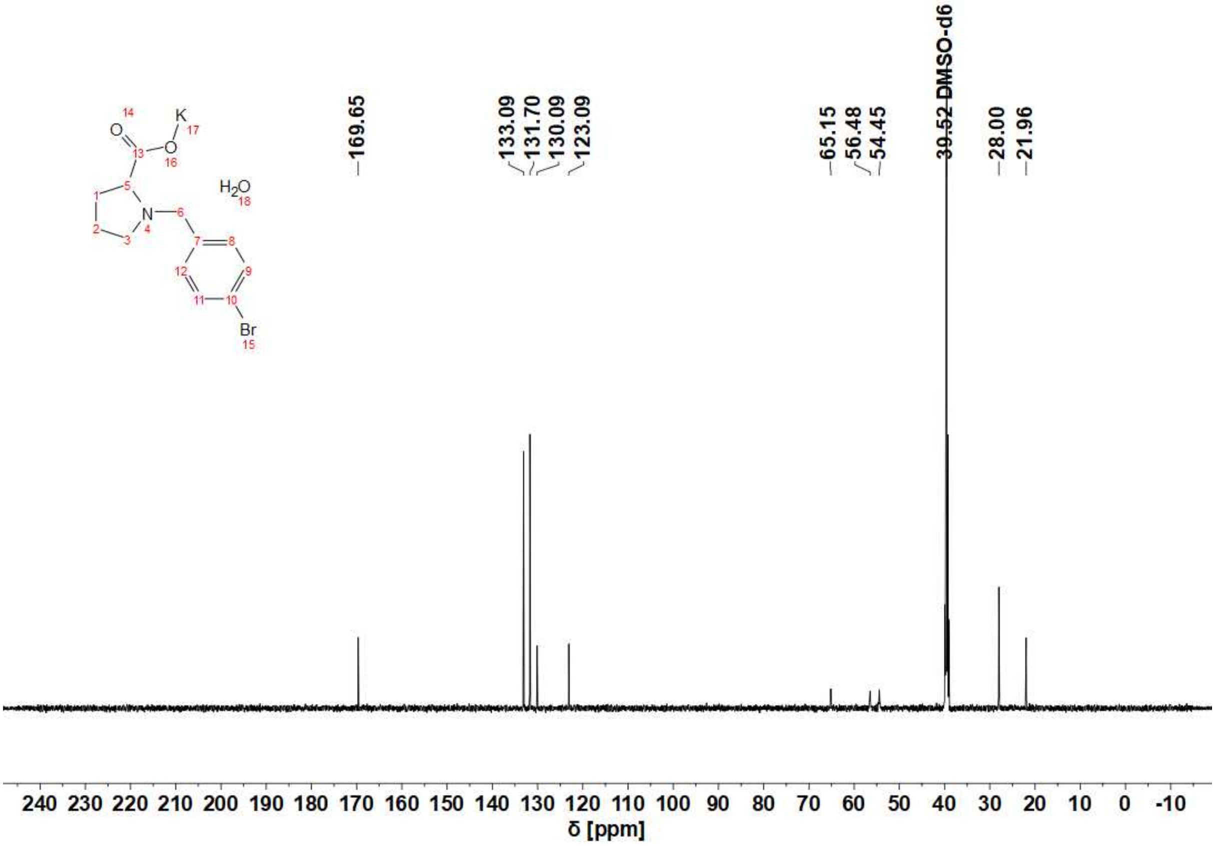

Figure S61: $^{13}C\{^{1}H\}$ NMR spectrum (DMSO-d_6, 151 MHz) of potassium *N*-(4-bromobenzyl)prolinate monohydrate (**S4b**).

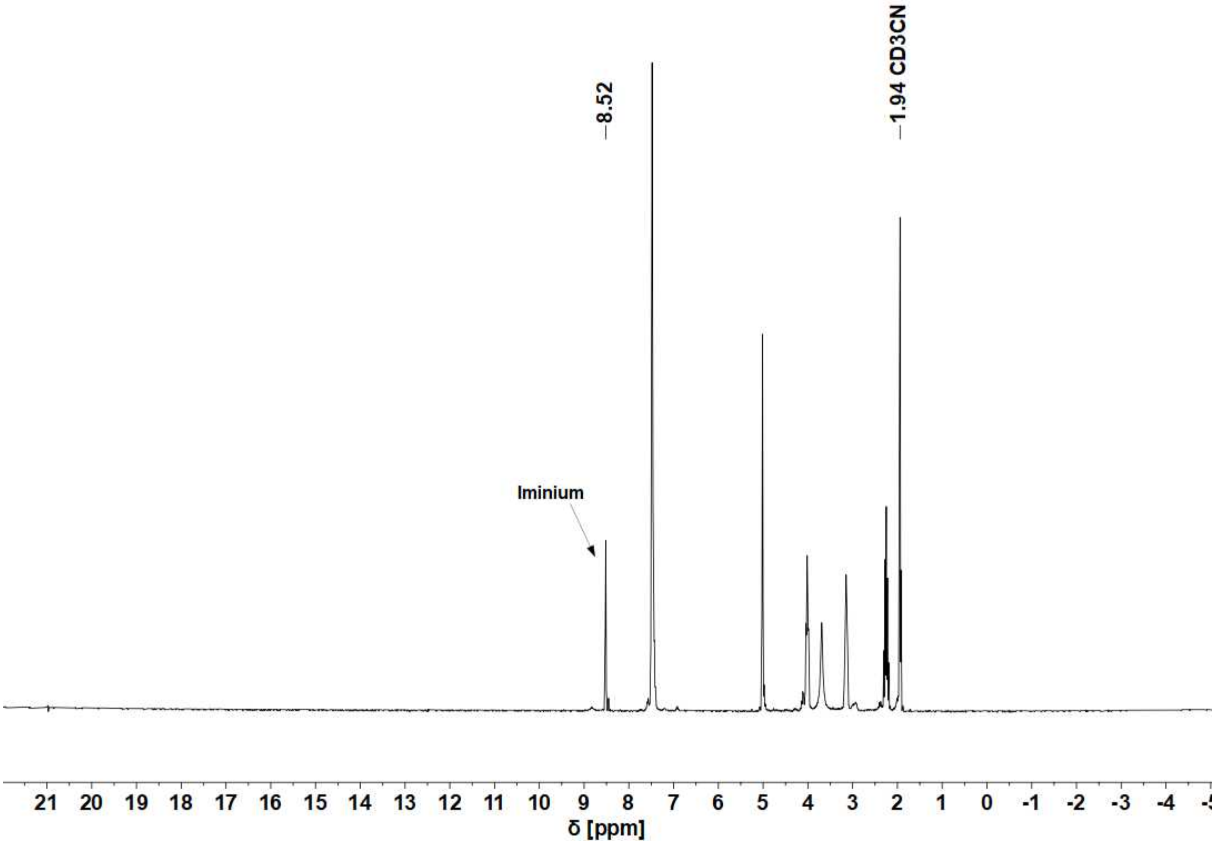

Figure S62: ^{1}H NMR spectrum (CD_3CN, 300 MHz) of the observed iminium intermediate during the synthesis of starting material **4**.

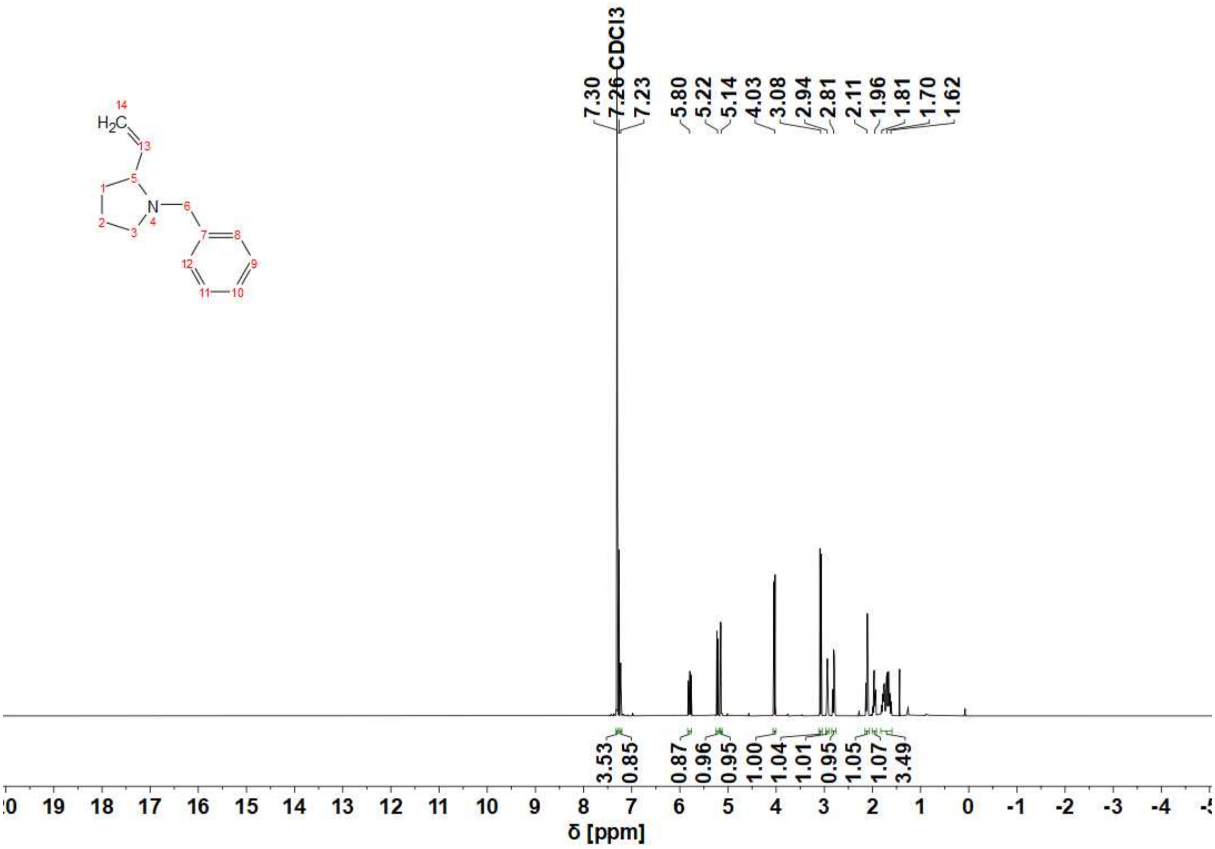

Figure S63: ^{1}H NMR spectrum ($CDCl_3$, 600 MHz) of *N*-benzyl-2-vinylpyrrolidine (**4**).

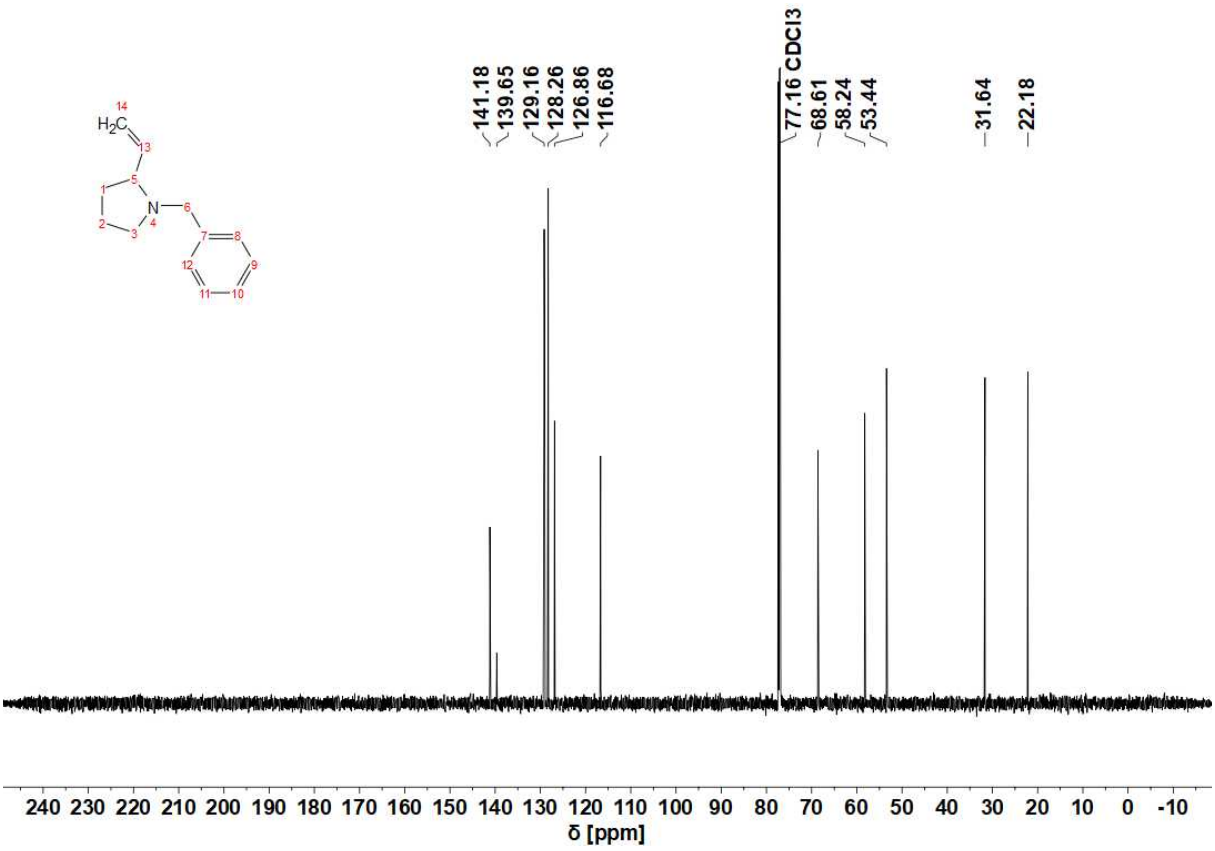

Figure S64: $^{13}C\{^{1}H\}$ NMR spectrum ($CDCl_3$, 151 MHz) of *N*-benzyl-2-vinylpyrrolidine (**4**).

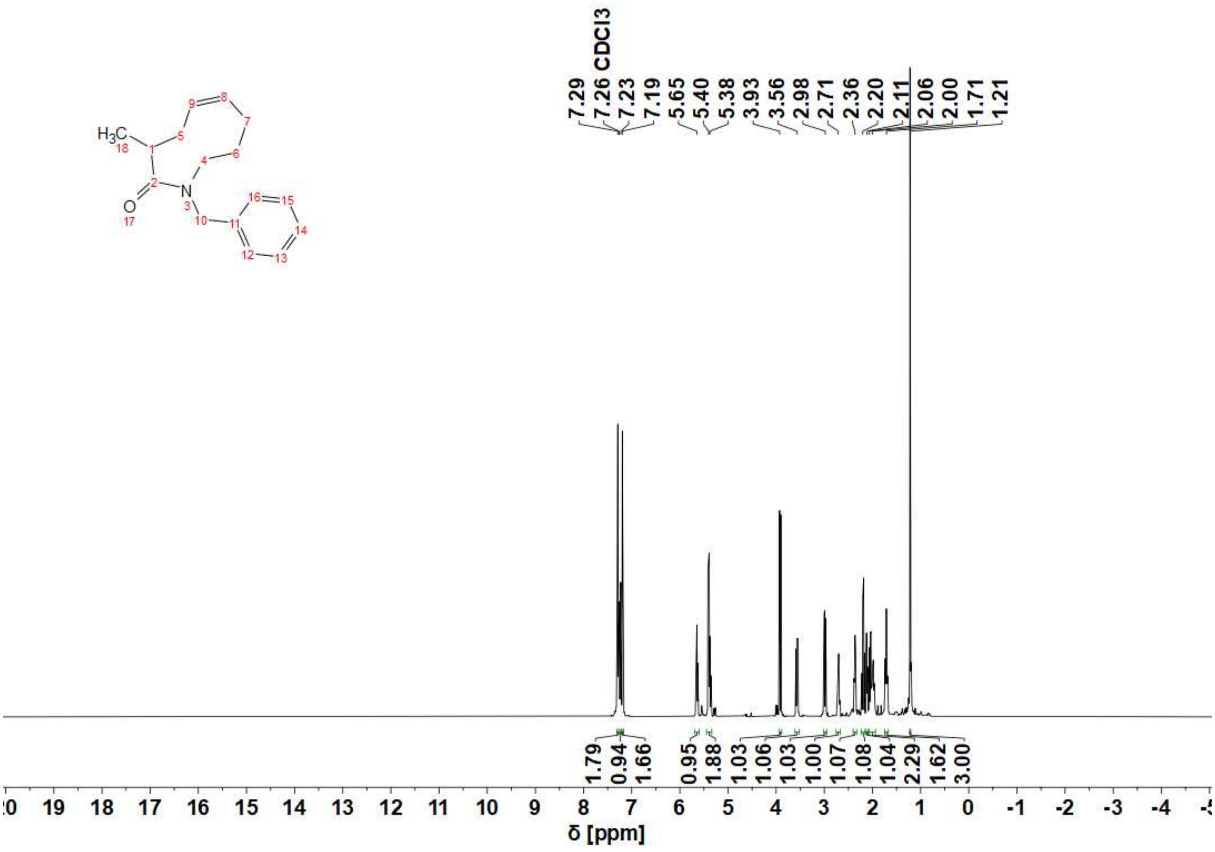

Figure S65: ^{1}H NMR spectrum ($CDCl_3$, 600 MHz) of 1-benzyl-3-methyl-1,3,4,7,8,9-hexahydro-2*H*-azonin-2-one (**5**).

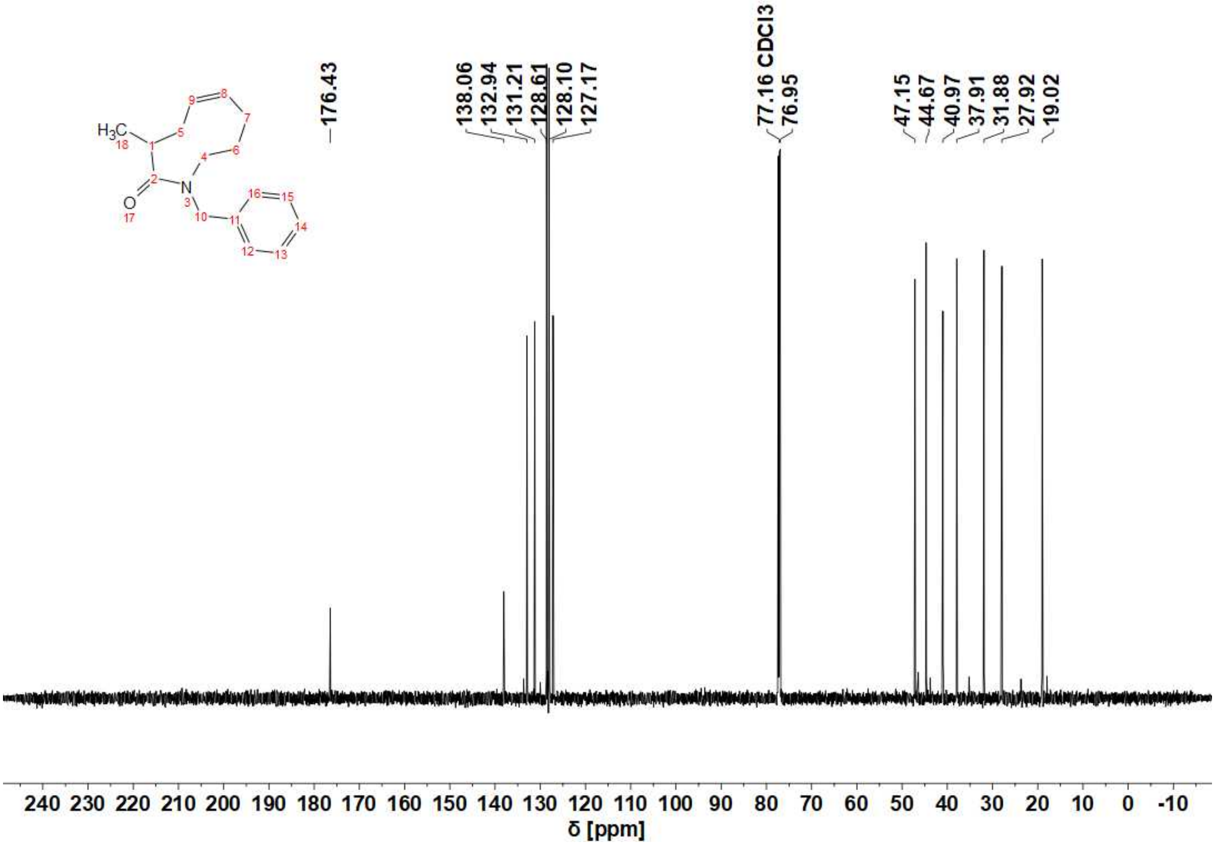

Figure S66: $^{13}C\{^{1}H\}$ NMR spectrum ($CDCl_3$, 151 MHz) of 1-benzyl-3-methyl-1,3,4,7,8,9-hexahydro-2*H*-azonin-2-one (**5**).
